# Synthesis of spiro-1,2-dihydropyridine derivatives via cycloisomerization of 3-aza-1,5-enynes catalyzed by gold(I) complex/polyoxometalate hybrids

**DOI:** 10.3762/bjoc.22.106

**Published:** 2026-09-28

**Authors:** Titien Simon, Fabian Schlimpen, Pierre de Frémont, Stefan Chassaing, Aurélien Blanc

**Affiliations:** 1 Institut de Chimie, UMR 7177-CNRS, Université de Strasbourg, 4 rue Blaise Pascal, CS 90032, 67081 Strasbourg, France; 2 Eco-Efficient Products and Processes Laboratory (IRL3464 CNRS/Syensqo), 3966 Jin Du Rd., Xin Zhuang Industrial Zone, Shanghai, China

**Keywords:** 3-aza-1,5-enynes, cycloisomerization, 1,2-dihydropyridines, gold, polyoxometalates

## Abstract

This study reports an optimized method for the synthesis of spiro-1,2-dihydropyridines via the cycloisomerization of 3-aza-1,5-enynes, catalyzed by heterogeneous gold(I)–polyoxometalate (POM) hybrids. By replacing traditional homogeneous catalysts with these hybrid systems, we achieved high yields (up to 82% isolated) in short reaction times, while minimizing product degradation. The reaction scope was demonstrated across a broad range of substrates, including aryl, alkyl, and heterocyclic derivatives, as well as variations in ring size and nitrogen substituents using 5 mol % of [Au^I^(IPr)(MeCN)][H_5_P_2_W_18_O_64_] as catalyst.

## Introduction

**Scheme 1:**
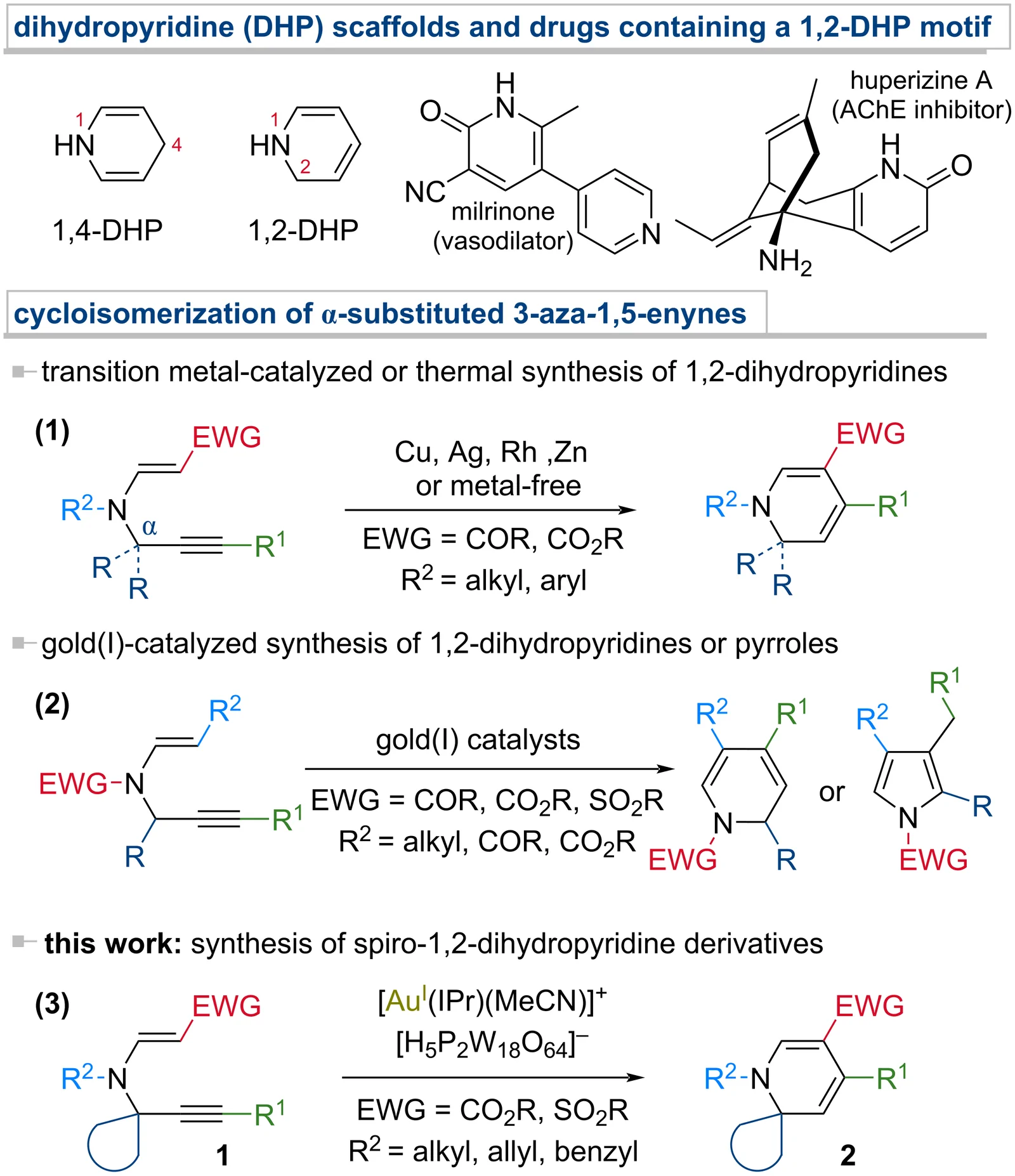
Bioactive 1,2-dihydropyridine derivatives and synthetic access from 3-aza-1,5-enynes by cycloisomerization.

Dihydropyridines (DHPs) are valuable compounds in organic synthesis and exhibit a wide variety of biological activities [1]. Among the different isomeric structures, 1,2- and 1,4-dihydropyridines are by far the most represented in the literature [2]. While 1,4-DHPs are well-established as core structures in blockbuster drugs [3], 1,2-DHPs have received comparatively less attention in medicinal chemistry [4]. Nevertheless, both synthetic or natural drugs, such as milrinone, a pulmonary vasodilator, and, huperzine A, a reversible acetylcholinesterase inhibitor used in the treatment of Alzheimer disease, highlight the therapeutic potential of this scaffold [4] (Scheme 1, top). In addition,1,2-DHPs represent useful intermediates in the total synthesis of natural products [5–7]. However, examples of 1,2-DHPs featuring a tertiary, spiro carbon at the C2 position are rare, but representing a promising yet largely untapped area for the development of bioactive compounds [8].

Among the synthetic approaches of 1,2-DHPs [9], the cycloisomerization of 3-aza-1,5-enynes stands out as a powerful and versatile strategy (Scheme 1, reaction 1). This transformation can be catalyzed by various transition metals, including copper [10–11], silver [12], rhodium [13], or zinc [14]. 3-Aza-1,5-enyne derivatives can also undergo 6-*endo*-dig cyclization upon thermal or electrophilic activation [15]. Alternatively, spiro-1,2-DHP derivatives can be synthesized from propargyl vinyl ethers in the presence of primary amines under microwave-assisted heating [16].

Gold(I) catalysts, renowned for their efficiency in cycloisomerization reactions [17–19], also promote the rearrangement of 3-aza-1,5-enynes bearing an electron-withdrawing group on the nitrogen atom (Scheme 1, reaction 2). However, the reactivity of these substrates depends on their substitution pattern, the gold catalyst employed, and sometimes the additives used [20–21]. Indeed, controlling the regioselectivity of the enamine addition enables the formation of either 1,2-DHPs [22–23] or pyrrole derivatives [24–25], the latter arising via Claisen rearrangement and 5-*exo*-dig cyclization.

Despite these advances, α-tertiary 1,2-DHP derivatives, particularly spirocyclic 1,2-DHPs, remain underexplored due to challenges in reactivity and selectivity. Existing methods [18–19,21,23,25–26] often suffer from prolonged reaction times, moderate yields, or limited substrate scope, and none employ gold(I) catalysis, prompting the need for an efficient and versatile catalytic system.

In this study, we report the efficient synthesis of spiro-1,2-dihydropyridines **2** via the cycloisomerization of *N*-alkyl α-tertiary 3-aza-1,5-enynes **1**, derived from propargylamines, using heterogeneous gold(I)/polyoxometalate (POM) hybrids catalysts (Scheme 1, reaction 3).

## Results and Discussion

### Optimization

The propargylamine precursors of 3-aza-1,5-enynes **1** are readily accessible through the reported ketone–amine–alkyne coupling reaction (KA^2^) with primary amines under Cu^I^-zeolite catalysis, offering different points of diversification and thus enabling the exploration of structural diversity [26]. We began our optimization studies with the 1,5-enyne **1a**, which was subjected to cycloisomerization in dichloroethane (DCE) in the presence of 5 mol % of classical phosphine or carbene gold(I) catalysts (Table 1, entries 1–4). Under these homogeneous conditions, we were pleased to observe promising time conversions (2–5 h) and yields (43–48%).

**Table 1:** Optimization of azaspiro-1,5-enyne **1a** cycloisomerization into spiro-1,2-dihydropyridine **2a**.

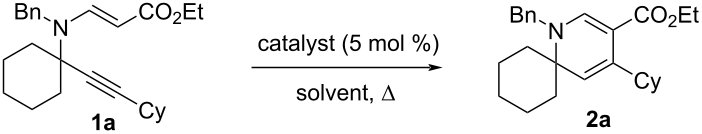					

Entry	Catalyst (5 mol %)	*T* (°C)	Solvent	Time (h)	Yield^a^ (%)

**1**	Au^I^(PPh_3_)NTf_2_	100	DCE	2	45
**2**	Au^I^(JohnPhos)NTf_2_	100	DCE	5	43
**3**	Au^I^(IPr)NTf_2_	100	DCE	2	44
**4**	Au^I^(IPr)(MeCN)SbF_6_	100	DCE	2	48
**5**	[Au^I^(PPh_3_)(MeCN)][H_2_PW_12_O_40_] (**A**)	100	DCE	0.5	47
**6**	[Au^I^(PPh_3_)(MeCN)][H_5_P_2_W_18_O_64_] (**B**)	100	DCE	0.5	41
**7**	[Au^I^(IPr)(MeCN)][H_2_PW_12_O_40_] (**C**)	100	DCE	0.5	50
**8**	[Au^I^(IPr)(MeCN)][H_5_P_2_W_18_O_64_] (**D**)	100	DCE	0.5	53
**9**	**A**	60	DCE	2	79
**10**	**C**	60	DCE	3	54
**11**	**D**	60	DCE	6	75
**12**	**A**	60	MeCN	2	90 (73)^b^
**13**	**D**	60	MeCN	2	**94** (82)^b^
**14**	Au^I^(IPr)(MeCN)SbF_6_	60	MeCN	2	73 (63)^b^
**15**	H_3_PW_12_O_40_ or H_6_P_2_W_18_O_64_	60	MeCN	24	–^c^

^a^Calculated yield by ^1^H NMR integration relative to an internal standard (MeNO_2_); ^b^Isolated yield; ^c^Degradation occurs.

We next turned our attention to heterogeneous gold(I)/POM hybrids as potential catalysts [27–30], which have demonstrated strong stability, efficiency and polyvalence in gold(I) catalysis, sometimes surpassing traditional homogeneous gold catalysts. Two cationic gold(I) complexes, i.e., Au^I^(PPh_3_)^+^ and Au^I^(IPr)^+^ (IPr = 1,3-bis(2,6-diisopropylphenyl)imidazol-2-ylidene), were combined with two anionic heteropolyacids (HPA, i.e., conjugate acids of POMs), H_2_PW_12_O_40_^−^ or H_5_P_2_W_18_O_64_^−^. The resulting four catalysts (**A**–**D**) were evaluated under the same reaction conditions (Table 1, entries 5–8), affording **2a** in slightly improved yields (47–53% for **A**, **C**, and **D**), together with markedly enhanced reaction kinetics, as full conversion was achieved within only 30 min.

During all these experiments, a significant amount of degradation products was observed in the ¹H NMR spectra of the crude reaction mixtures. Consequently, the reaction temperature was lowered to 60 °C, resulting in the formation of spiro-1,2-dihydropyridine **2a** in good yields (75–79%) using catalysts [Au^I^(PPh_3_)(MeCN)][H_2_PW_12_O_40_] (**A**) and [Au^I^(IPr)(MeCN)][H_5_P_2_W_18_O_64_] (**D**) (Table 1, entries 9–11), while catalyst **C** proved less efficient, yielding only 54%. A longer reaction time was required with the less electrophilic cationic Au^I^(IPr)^+^ hybrid compared to Au^I^(PPh_3_)^+^ (6 h vs 2 h, entries 11 vs 9). Various solvents were then screened under the latter conditions using catalyst **A** (see Table S1 in Supporting Information File 1), and we found that performing the reaction in acetonitrile afforded a better yield of 90% for **2a** in 2 h (Table 1, entry 12, 73% isolated). We also tested catalyst **D** in acetonitrile, and we were pleased to obtain a slightly improved yield of 94% (Table 1, entry 13, 82% isolated). Under the same conditions, reducing the catalyst loading to 2 mol % resulted in low conversion (<30% after 24 h). The reaction was also performed under optimized conditions using the homogeneous Au(IPr)SbF₆ catalyst (Table 1, entry 14), achieving 73% conversion, confirming the superiority of our gold-POM hybrids. Finally, control experiments with H_3_PW_12_O_40_ or H_6_P_2_W_18_O_64_ were conducted (Table 1, entry 15) and, whatever the HPAs used, only degradation products were observed by ^1^H NMR.

### Scope

**Scheme 2:**
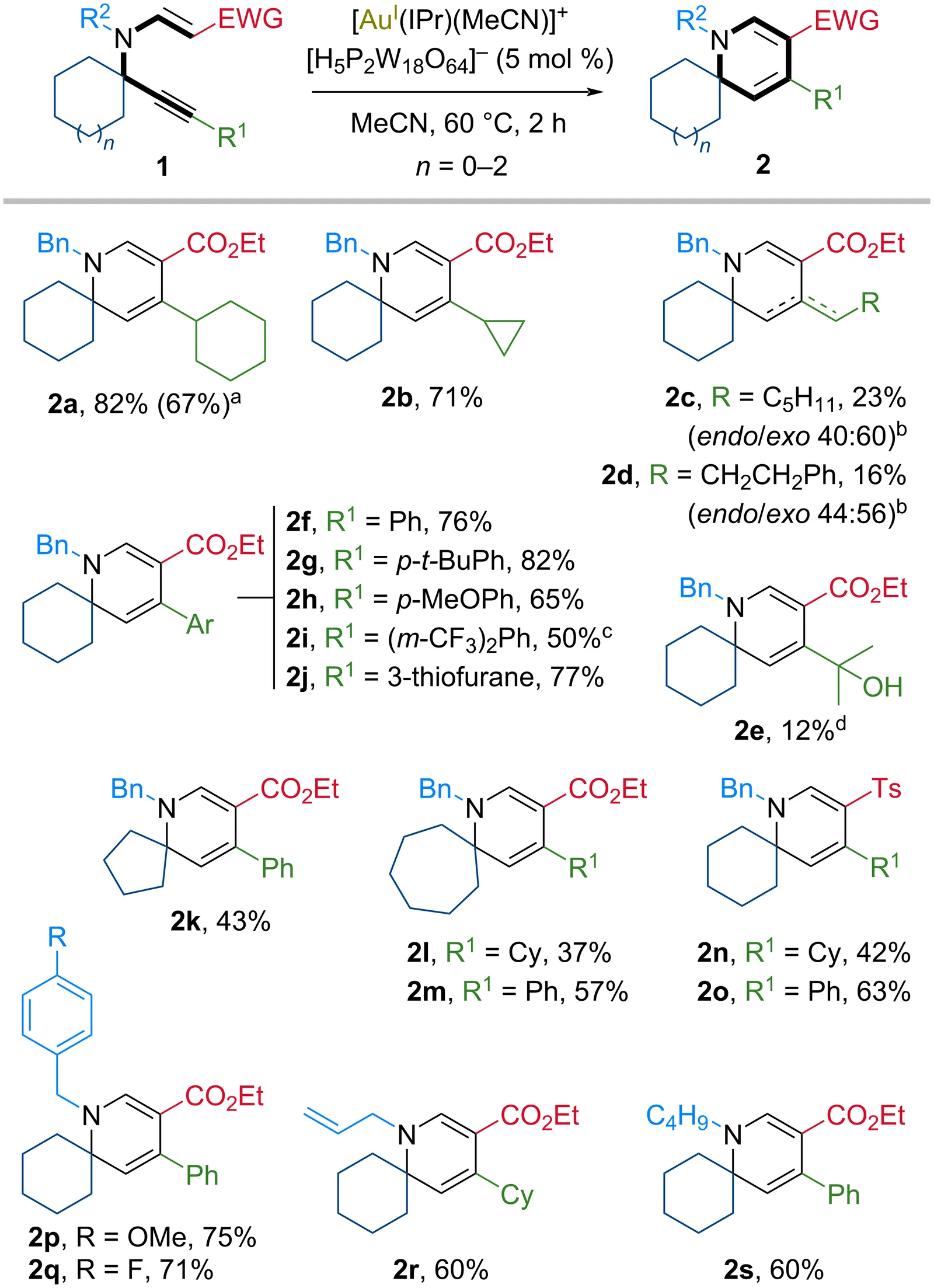
Scope of the cycloisomerization of *N*-alkyl α-tertiary 3-aza-1,5-enynes **1** into spiro-1,2-dihydropyridines **2**. ^a^Gram-scale. ^b^Calculated ratio determined by ^1^H NMR integration. ^c^Run at 80 °C. ^d^35% of conversion after 24 h of heating.

Having established the optimal conditions for the cycloisomerization reaction using Au^I^-POM hybrid **D** as catalyst (Table 1, entry 13), we first carried out the rearrangement of **1a** on a gram scale, affording **2a** in a slightly reduced isolated yield of 67% (Scheme 2).

We then investigated the scope of the reaction, focusing on the influence of the R^1^ substituent on the alkynyl moiety. The cyclopropyl-substituted substrate **1b** underwent smooth cyclization, yielding the spiro derivative **2b** in good yield. However, substrates **1c**,**d**, bearing methylene substituents at the propargylic position, provided the dihydropyridine derivatives **2c**,**d** in low yields (<25%) as *endo*/*exo* mixtures, likely due to isomerization of the endocyclic double bond. A tertiary alcohol in R^1^ strongly limited the conversion (35%) even after prolonged reaction time, presumably due to steric effects perturbating alkyne activation by gold. Consequently, spiro derivative **2e** was isolated in only 12% yield.

Introducing aryl or thiofuran substituents at the propargylic position (substrates **1f**–**j**) restored the reactivity, affording spiro-1,2-dihydropyridines **2f**–**j** in good yields (50–82%). Notably, electron-withdrawing groups on the aryl ring required more elevated temperatures (80 °C) to achieve good conversion (**2i** vs **2f**–**h**), though the yield remained moderate (50%).

We next examined the influence of the ring size in the starting material. Five- and seven-membered rings were investigated with compounds **1k**–**m** and, in both cases, the corresponding 1,2-dihydropyridine derivatives **2k**–**m** were obtained in moderate but lower yields compared to the six-membered ring analog (43% for **2k** and 57% for **2m** vs 76% for **2f**).

The ester group (EWG) on the enamine moiety could be replaced by a *para*-tolylsulfonate substituent, enabling access to *C*-tosylated derivatives **2n**–**o**. Finally, the nitrogen substituent (R^2^) was successfully modulated, as demonstrated by the efficient synthesis of *N*-*para*-methoxybenzyl, *N*-*para*-fluorobenzyl, *N*-allyl and *N*-alkyl 1,2-dihydropyridine derivatives **2p**–**s** (60–75%).

### Catalyst recycling

**Figure 1:**
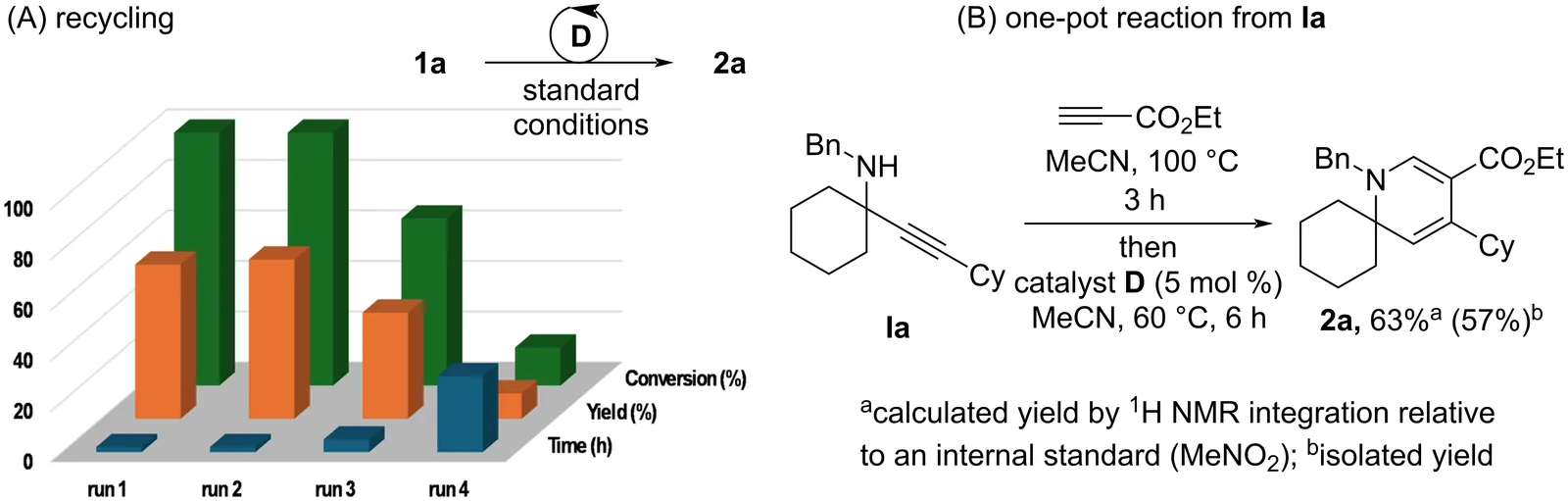
(A) Recycling of catalyst **D** in the cycloisomerization of **1a** and (B) one-pot formation of **2a** from propargylamine **Ia**.

Given that heterogeneous gold(I)–POM hybrids can be recovered by precipitation [25–26], the recyclability of the catalyst **D** was next investigated to assess its robustness in the cycloisomerization (Figure 1A). After completion of the reaction, the Au(I)–POM hybrid catalyst was readily recovered after precipitation upon addition of diethyl ether, followed by centrifugation and then reused in subsequent runs under the optimized reaction conditions. Notably, the catalyst could be recycled up to two consecutive times without significant loss of activity, maintaining consistent conversions and comparable isolated yields across the cycles. However, a sharp decline in catalytic activity was observed in the fourth run, with only 15% conversion after 30 h of reaction. As previously reported by our group, this decrease in activity may be attributed to a loss of catalysts during the washing phase of the recycling step [26]. Indeed, upon heating, the catalyst may undergo successive in situ H^+^/gold(I)^+^ exchanges around POM units, leading to a more soluble complex in which the POM unit charge is fully counterbalanced by NHC–gold complex cations.

We then explored the feasibility of a one-pot reaction starting from propargylic amine precursor **Ia** (Figure 1B). Compound **Ia** was thus condensed on ethyl propiolate in acetonitrile at 100 °C for 3 h. Upon completion of the reaction to form derivative **1a**, the temperature was reduced at 60 °C and 5 mol % of [Au^I^(IPr)(MeCN)][H_5_P_2_W_18_O_64_] (**D**) was then added to the reaction mixture. While the formation of 1,2-dihydropyridine **2a** was confirmed, the yield was lower (42%) compared to the overall yield obtained via the sequential two-step process (81%).

## Conclusion

In this study, we have demonstrated the catalytic potential of heterogeneous gold(I)–POM hybrid materials for the cycloisomerization of 1,6-enynes, providing rapid access to spiro-1,2-dihydropyridine derivatives (19 examples). Optimization efforts identified Au(I)–POM hybrid as the most effective catalyst, enabling modest to high yields (12–82%) under mild conditions. The substrate scope proved broad, accommodating various aryl, alkyl, and heterocyclic substituents, as well as modifications in ring size and nitrogen substituents. The recyclability of the catalyst was confirmed for two consecutive cycles, though diminished activity in subsequent runs suggests in situ POM/gold(I) exchanges as a limiting factor. Additionally, a one-pot synthetic approach was developed, offering a practical alternative to sequential methods. This work highlights the potential of heterogeneous gold catalysts in complex organic transformations and opens avenues for further exploration in catalyst design and sustainable synthetic methodologies.

## Experimental

General procedure for the preparation of spiro-1,2-dihydropyridine **2** derivatives: [Au^I^(IPr)(MeCN)][H_5_P_2_W_18_O_62_] (5 mol %) was added to a solution of enyne **1** in MeCN (1 M) in a screw cap tube (3 mL) equipped with a stirring bar. The reaction flask was flushed with argon before sealing. The mixture was placed into a pre-heated aluminum block at 60 °C and stirred until completion. The catalyst was filtered off through a short pad of Celite^®^ and the residue was washed with dichloromethane (3 × 20 mL). The filtrate was concentrated under reduced pressure, and the crude product was purified by column chromatography over silica gel (cyclohexane/EtOAc) to afford the product **2**.

## Supporting Information

File 1 Experimental procedures, characterization data and copies of spectra.

## Data Availability

Data generated and analyzed during this study is openly available in nmrXiv at https://doi.org/10.57992/nmrxiv.p165
