# Supplementary material for: Synthesis of spiro-1,2-dihydropyridine derivatives via cycloisomerization of 3-aza-1,5-enynes catalyzed by gold(I) complex/polyoxometalate hybrids
Source: Beilstein J Org Chem. 2026 Sep 28;22:1316–22. doi: 10.3762/bjoc.22.106 (PMC13635902; doi:10.3762/bjoc.22.106)
Supplement: Supplementary file 1 [file Beilstein_J_Org_Chem-22-1316-s001.pdf]

BEILSTEIN JOURNAL OF ORGANIC CHEMISTRY

Supporting Information

for

Synthesis of spiro-1,2-dihydropyridine derivatives via cycloisomerization of 3-aza-1,5-enynes catalyzed by gold(I) complex/polyoxometalate hybrids

Titien Simon, Fabian Schlimpen, Pierre de Frémont, Stefan Chassaing
and Aurélien Blanc

Beilstein J. Org. Chem. **2026,** *22,* 1316–1322. doi:10.3762/bjoc.22.106

Experimental procedures, characterization data and copies of spectra

License and Terms: This is a supporting information file under the terms of the Creative Commons Attribution License (https://creativecommons.org/licenses/by/4.0). Please note that the reuse, redistribution and reproduction in particular requires that the author(s) and source are credited and that individual graphics may be subject to special legal provisions.
The license is subject to the *Beilstein Journal of Organic Chemistry* terms and conditions: (https://www.beilstein-journals.org/bjoc/terms)

Table of contents

1. Organic synthesis *S2*

1.1 General information *S2*

1.2 Optimization of the reaction conditions (Table S1) *S2*

*1.3 Preparation of propargylamines (**I**)* *S3*

*1.4 Preparation of 3-aza-1,5-enynes (**1**)* *S7*

*1.5 Synthesis of 1,2-dihydropyridines (**2**)* *S11*

2. ^{1}H and ^{13}C NMR spectra *S17*

1. *Organic synthesis*

1.1 General information

All chemicals were of the best commercially available grade and used without further purification. Reactions taking place in screw cap tubes were flushed with argon before sealing. Reactions were monitored by thin layer chromatography carried out on silica (silica gel 60 F254, Merck) plates using UV-light, $KMnO_4$, vanillin (especially for carbonyls) and ninhydrine (for amines and imines) stains for visualization. Column chromatographies were performed on silica gel 60 (40–63 µm, Merck). Further abbreviations were used to consider solvents: EtOAc for ethyl acetate and DCM for dichloromethane. IR spectra were recorded neat on a Bruker Alpha II Fourier transform spectrometer. NMR spectra were recorded on a Burker Avance III spectrometer operating at specified power (Burker Corporation, Billerica, MA, USA). ^{1}H and ^{13}C NMR spectra were recorded at 500 MHz for ^{1}H and 126 MHz for ^{13}C NMR experiments at 298 K. Chemical shifts (δ) and coupling constants (*J*) are given in parts per million (ppm) and Hertz (Hz), respectively. Chemical shifts (δ) are reported relative to the residual solvent as an internal standard ($CDCl_3$: δ 7.26 ppm for ^{1}H and δ 77.16 ppm for ^{13}C). Assignments were determined based on chemical shifts, multiplicities and coupling constants. COSY, HSQC and HMBC experiments were sometimes used to interpret spectra for related compounds. Low-(MS) and high-resolution mass spectra (HRMS) data were recorded on a MicroTOF spectrometer (Broker Corporation, Billerica, MA, USA) equipped with an orthogonal electro spray interface (ESI) or with atmospheric pressure chemical ionisation (APCI). The parent ions $[M + H]^+$ or $[M + Na]^+$ are indicated. Mass spectra were obtained from the « Service de Spectroscopie de Masse » of the Fédération de Chimie « Le Bel » (FR2010). Gold(I)-polyoxometalate catalysts were synthesized following described procedures.[1]

1.2 Optimization of the reaction conditions (Table S1)

Table S1. Solvents Optimization of Azaspiro-1,5-enyne **1a** Cycloisomerization into Dihydropyridine **2a** using 5 mol% of catalyst **A**.

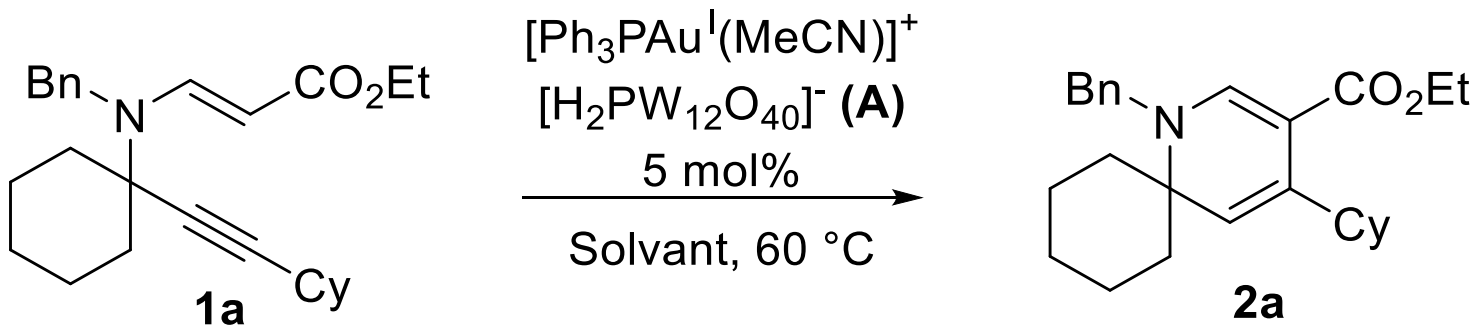

Entry	Solvent	Time (h)	Yield^a (%)
**1**	DCE	2	79
**2**	Toluene	24	71
**3**	HFIP	2	68
**4**	EtOH	8	61
**5**	2-MeTHF	4	73
**6**	AcOEt	24	77
**7**	MeCN	2	90

^{a}Calculated yield by ^{1}H NMR integration relative to an internal standard ($MeNO_2$).

[1] For the synthesis of NHC-gold(I) polyoxometalate catalysts, see: F. Sirindil *et al*. *Chem. Eur. J*. **2018**, *24*, 12630. For the synthesis of phosphines-gold(I) polyoxometalate catalysts, see: D. Hueber *et al. Organometallics* **2010**, *34*, 5065.

1.3 Preparation of propargylamines (**I**)

General procedure A: synthesis of progargylamines (I) via KA² reaction

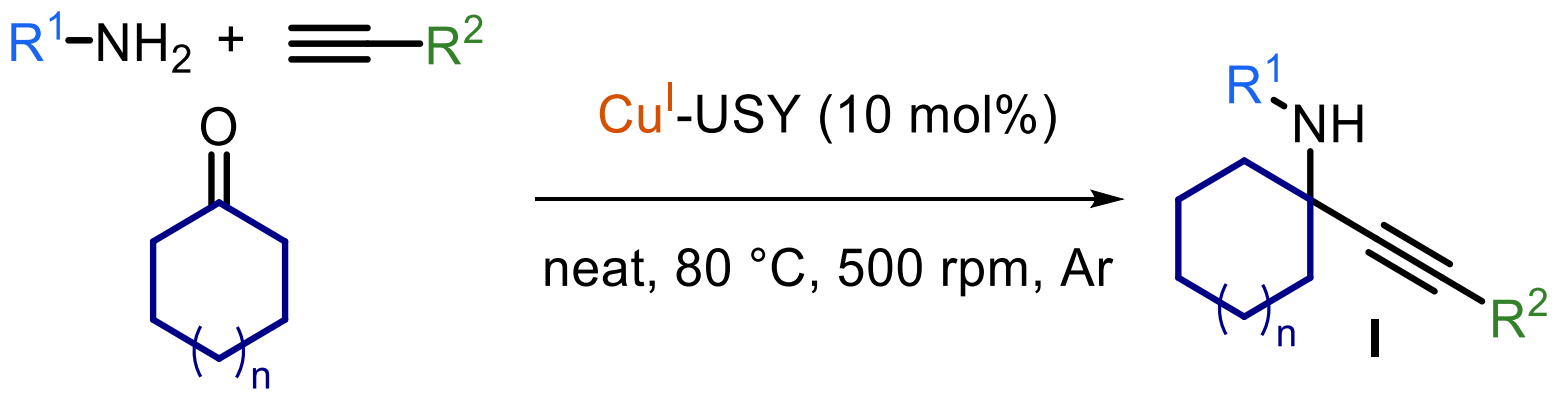

Ketone (1.0 mmol, 1 equiv), alkyne (1.2 mmol, 1.2 equiv) and Cu^I-USY (36 mg, 10 mol %) were successively added to a screw cap tube (10 mL) and stirred at room temperature and 500 rpm for 15 min. Next, amine (1.0 mmol, 1.0 equiv) was added, and the mixture was progressively heated to 80 °C and left to stir for 48 h. Once cooled to room temperature, the reaction mixture was diluted with DCM (2 mL), filtered over Celite and washed with DCM (3 x 20 mL). The filtrate was concentrated under reduced pressure and the crude product was purified by flash chromatography over silica gel (cyclohexane/EtOAc).

***N*-Benzyl-1-(cyclohexylethynyl)cyclohexan-1-amine (Ia).**

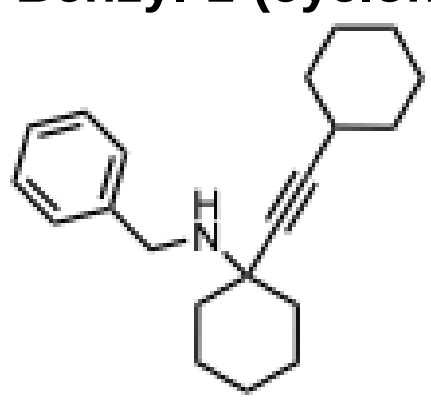

Ia
Chemical Formula: $C_{21}H_{29}N$
Molecular Weight: 295,47

Following the **General Procedure A** starting from cyclohexanone (207 µL, 2.0 mmol), cyclohexylacetylene (265 µL, 2.4 mmol), Cu^I-USY (72 mg) and benzylamine (218 µL, 2.0 mmol), the title compound **Ia** was obtained (272 mg, 0.92 mmol, 46% yield) as a yellow oil after purification by column chromatography of the crude residue (cyclohexane/EtOAc 98/2 to 94/6). **Rf** = 0.55 (SiO_2, cyclohexane/EtOAc 8/2). **ATR FTIR** ν = 2926, 2851, 1604, 1495, 1447, 1345, 1115, 734, 697 cm^{-1}. **^{1}H NMR** (500 MHz, $CDCl_3$) δ 7.37-7.34 (m, 2H), 7.33-7.29 (m, 2H), 7.25-7.21 (m, 1H), 3.89 (s, 2H), 2.48 (m, 1H), 1.88-1.13 (m, 20H) ppm. **^{13}C NMR** (126 MHz, $CDCl_3$) δ 141.5, 128.7, 128.5, 126.9, 89.2, 83.9, 55.0, 48.1, 38.7, 33.4, 29.1, 26.2, 26.2, 24.9, 23.3 ppm. **HRMS:** 296.2370 $[M+H]^+$, calcd 296.2373.

***N*-Benzyl-1-(cyclopropylethynyl)cyclohexan-1-amine (Ib).**

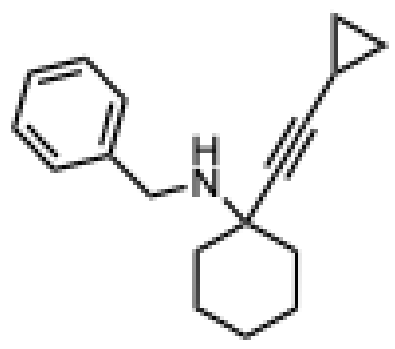

Ib
Chemical Formula: $C_{18}H_{23}N$
Molecular Weight: 253,39

Following the **General Procedure A** starting from cyclohexanone (207 µL, 2.0 mmol), cyclopropylacetylene (203 µL, 2.4 mmol), Cu^I-USY (72 mg) and benzylamine (218 µL, 2.0 mmol), the title compound **Ib** was obtained (178 mg, 0.70 mmol, 35% yield) as a yellow oil after purification by column chromatography of the crude residue (cyclohexane/EtOAc 98/2 to 94/6). **Rf** = 0.48 (SiO_2, cyclohexane/EtOAc 8/2). **ATR FTIR** ν = 2928, 2852, 1450, 1112, 1027, 911, 734 cm^{-1}. **^{1}H NMR** (500 MHz, $CDCl_3$) δ 7.37-7.34 (m, 2H), 7.33-7.29 (m, 2H), 7.25-7.21 (m, 1H), 3.86 (s, 2H), 1.84-1.77 (m, 2H), 1.67-1.52 (m, 5H), 1.44-1.36 (m, 2H), 1.30 (tt, J = 8.2, 5.0 Hz, 1H), 1.24-1.17 (m, 1H), 0,78 (dd, J = 8.3, 2.6 Hz, 2H), 0.66 (dd, J = 8.2, 2.6 Hz, 2H) ppm. **^{13}C NMR** (126 MHz, $CDCl_3$) δ 141.4, 128.6, 128.5, 126.9, 87.9, 79.2, 54.9, 48.0, 38.5, 26.1, 23.1, 8.7, 0.3 ppm. **HRMS:** 254.1902 $[M+H]^+$, calcd 254.1903.

***N*-Benzyl-1-(oct-1-yn-1-yl)cyclohexan-1-amine (Ic).**

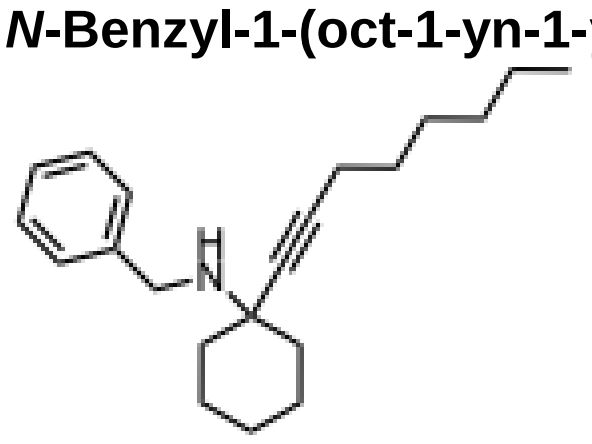

Ic
Chemical Formula: $C_{21}H_{31}N$
Molecular Weight: 297,49

Following the **General Procedure A** starting from cyclohexanone (207 µL, 2.0 mmol), 1-octyne (354 µL, 2.4 mmol), Cu^I-USY (72 mg) and benzylamine (218 µL, 2.0 mmol), the title compound **Ic** was obtained (263 mg, 0.88 mmol, 44% yield) as a pale-yellow oil after purification by column chromatography of the crude residue (cyclohexane/EtOAc 98/2 to 94/6). **Rf** = 0.59 (SiO_2, cyclohexane/EtOAc 8/2). **ATR FTIR** ν = 2927, 2854, 1462, 1448, 1116, 833, 735, 694 cm^{-1}. **^{1}H NMR** (500 MHz, $CDCl_3$) δ 7.39-7.35 (m, 2H), 7.34-7.30 (m, 2H), 7.26-7.22 (m, 1H), 3.89 (s, 2H), 2.27 (t, J = 7.0 Hz, 2H), 1.87-1.81 (m, 2H), 1.69-1.58 (m, 5H), 1.58-1.52 (m, 2H), 1.48-1.38 (m, 4H), 1.35-1.30 (m, 5H), 1.24-1.18 (m, 1H), 0.93-0.88 (m, 3H) ppm. **^{13}C NMR** (126 MHz, $CDCl_3$) δ 141.5, 128.6, 128.5, 126.9, 84.7, 84.0, 55.0, 48.1, 38.6, 31.5, 29.4, 28.7, 26.1, 23.1, 22.8, 18.9, 14.2 ppm. **HRMS:** 298.2542 $[M+H]^+$, calcd 298.2529.

N-Benzyl-1-(5-phenylpent-1-yn-1-yl)cyclohexan-1-amine (Id).

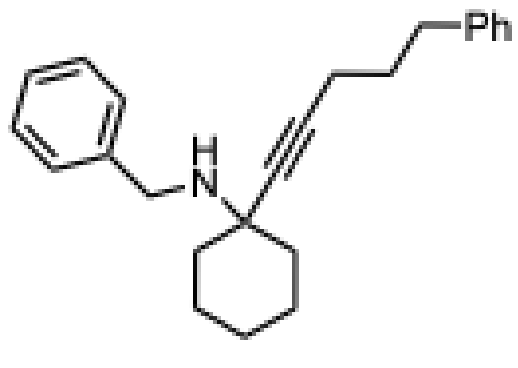

Id
Chemical Formula: $C_{24}H_{29}N$
Molecular Weight: 331,50

Following the **General Procedure A** starting from cyclohexanone (207 µL, 2.0 mmol), 5-phenyl-1-pentyne (364 µL, 2.4 mmol), Cu^{I}-USY (72 mg) and benzylamine (218 µL, 2.0 mmol), the title compound **Id** was obtained (234 mg, 0.70 mmol, 35% yield) as a pale-yellow oil after purification by column chromatography of the crude residue (cyclohexane/EtOAc 98/2 to 94/6). **Rf** = 0.46 (SiO_2, cyclohexane/EtOAc 8/2). **ATR FTIR** ν = 2928, 2852, 1495, 1451, 1113, 738, 694 cm^{-1}. **^{1}H NMR** (500 MHz, $CDCl_3$) δ 7.39-7.37 (m, 2H), 7.34-7.28 (m, 4H), 7.26-7.19 (m, 4H), 3.92 (s, 2H), 2.80-2.77 (m, 2H), 2.30 (t, *J* = 6.9 Hz, 2H), 1.91-1.84 (m, 4H), 1.72-1.50 (m, 5H), 1.49-1.42 (m, 2H), 1.29-1.20 (m, 1H) ppm. **^{13}C NMR** (126 MHz, $CDCl_3$) δ 142.0, 141.4, 128.7, 128.6, 128.5, 128.5, 126.9, 126.0, 84.6, 84.2, 55.0, 48.1, 38.6, 35.1, 31.1, 26.1, 23.2, 18.4 ppm. **HRMS:** 332.2384 $[M+H]^+$, calcd 332.2373.

4-(1-(Benzylamino)cyclohexyl)-2-methylbut-3-yn-2-ol (Ie).

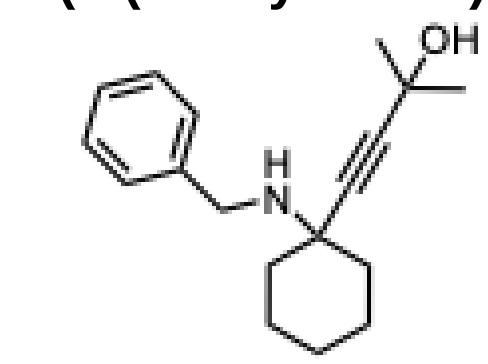

Ie
Chemical Formula: $C_{18}H_{25}NO$
Molecular Weight: 271,40

Following de **General Procedure A** starting from cyclohexanone (207 µL, 2.0 mmol), 2-methyl-3-butyn-2-ol (233 µL, 2.4 mmol), Cu^{I}-USY (72 mg) and benzylamine (218 µL, 2.0 mmol), the title compound **Ie** was obtained (48 mg, 0.18 mmol, 8% yield) as a yellow oil after purification by column chromatography of the crude residue (cyclohexane/EtOAc 98/2 to 94/6). **Rf** = 0.08 (SiO_2, cyclohexane/EtOAc 8/2). **ATR FTIR** ν = 2980, 2931, 2856, 1452, 1273, 1163, 1112, 733, 702 cm^{-1}. **^{1}H NMR** (500 MHz, $CDCl_3$) δ 7.38-7.29 (m, 4H), 7.26-7.22 (m, 1H), 3.87 (s, 2H), 1.89-1.82 (m, 2H), 1.71-1.57 (m, 6H), 1.56 (s, 6H), 1.47-1.40 (m, 2H) ppm. **^{13}C NMR** (126 MHz, $CDCl_3$) δ 141.2, 128.6, 128.6, 127.0, 89.7, 86.0, 65.6, 54.7, 48.1, 38.3, 32.1, 26.0, 23.1 ppm. **HRMS:** 272.2022 $[M+H]^+$, calcd 272.2009.

N-Benzyl-1-(phenylethynyl)cyclohexan-1-amine (If).

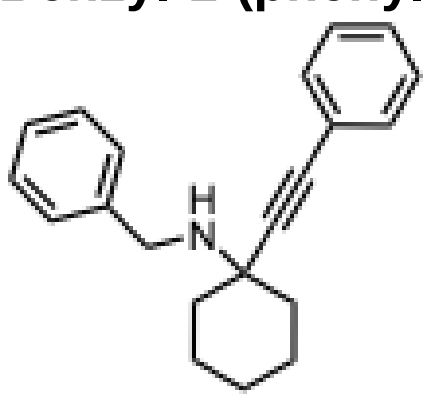

If
Chemical Formula: $C_{21}H_{23}N$
Molecular Weight: 289,42

Following the **General Procedure A** starting from cyclohexanone (207 µL, 2.0 mmol), phenylacetylene (264 µL, 2.4 mmol), Cu^{I}-USY (72 mg) and benzylamine (218 µL, 2.0 mmol), the title compound **If** was obtained (477 mg, 1.65 mmol, 83% yield) as a yellow oil after purification by column chromatography of the crude residue (cyclohexane/EtOAc 98/2 to 94/6). **Rf** = 0.57 (SiO_2, cyclohexane/EtOAc 8/2). **ATR FTIR** ν = 2936, 2917, 2856, 1596, 1489, 1443, 1285, 1116, 734, 696 cm^{-1}. **^{1}H NMR** (500 MHz, $CDCl_3$) δ 7.61-7.45 (m, 2H), 7.44-7.39 (m, 2H), 7.37-7.30 (m, 5H), 7.29-7.23 (m, 1H), 4.00 (s, 2H), 2.05-1.95 (m, 2H), 1.79-1.63 (m, 5H), 1.61-1.49 (m, 2H), 1.36-1.22 (m, 1H) ppm. **^{13}C NMR** (126 MHz, $CDCl_3$) δ 141.2, 131.8, 128.6, 128.6, 128.4, 127.9, 123.8, 93.7, 84.9, 55.5, 48.2, 38.4, 26.1, 23.2 ppm. **HRMS:** 290.1895 $[M+H]^+$, calcd 290.1903.

N-Benzyl-1-((4-(*tert*-butyl)phenyl)ethynyl)cyclohexan-1-amine (Ig).

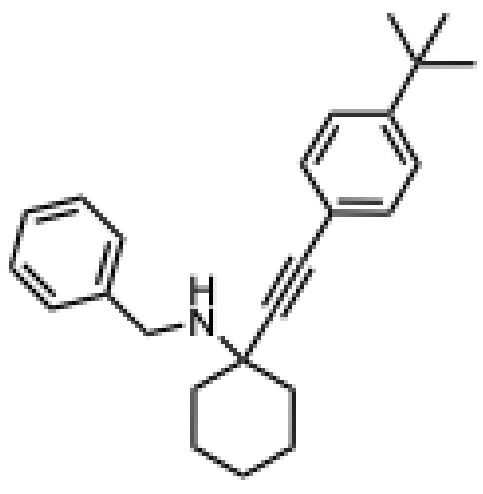

Ig
Chemical Formula: $C_{25}H_{31}N$
Molecular Weight: 345,53

Following the **General Procedure A** starting from cyclohexanone (207 µL, 2.0 mmol), 4-*tert*-butylphenylacetylene (433 µL, 2.4 mmol), Cu^{I}-USY (72 mg) and benzylamine (218 µL, 2.0 mmol), the title compound **Ig** was obtained (325 mg, 0.94 mmol, 47% yield) as a white solid after purification by column chromatography of the crude residue (cyclohexane/EtOAc 98/2 to 94/6). **Rf** = 0.48 (SiO_2, cyclohexane/EtOAc 8/2). **ATR FTIR** ν = 2932, 2854, 1464, 1268, 1117, 833, 768, 736, 696, 564 cm^{-1}. **^{1}H NMR** (500 MHz, $CDCl_3$) δ 7.42-7.37 (m, 4H), 7.35-7.29 (m, 4H), 7.25-7.21 (m, 1H), 3.97 (s, 2H), 2.01-1.93 (m, 2H), 1.75-1.62 (m, 5H), 1.57-1.47 (m, 2H), 1.32 (s, 9H), 0.89-0.77 (m, 1H) ppm. **^{13}C NMR** (126 MHz, $CDCl_3$) δ 151.2, 141.3, 131.5, 128.7, 128.6, 127.0, 125.4, 121.8, 93.0, 84.9, 55.5, 48.2, 38.4, 34.9, 31.4, 26.1, 23.2 ppm. **HRMS:** 346.2523 $[M+H]^+$, calcd 346.2529.

N-Benzyl-1-((4-methoxyphenyl)ethynyl)cyclohexan-1-amine (Ih).

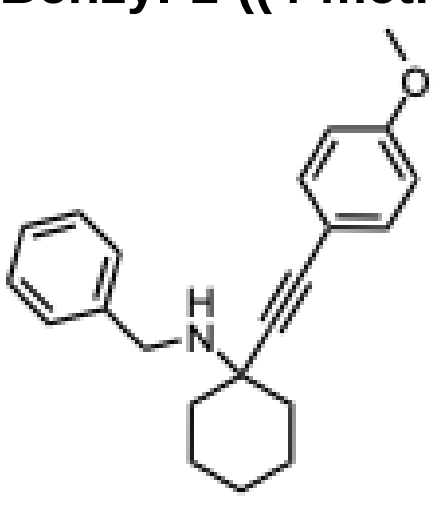

Ih
Chemical Formula: $C_{22}H_{25}NO$
Molecular Weight: 319,45

Following the **General Procedure A** starting from cyclohexanone (207 µL, 2.0 mmol), 4-methoxyphenylacetylene (311 µL, 2.4 mmol), Cu^{I}-USY (72 mg) and benzylamine (218 µL, 2.0 mmol), the title compound **Ih** was obtained (211 mg, 0.66 mmol, 33% yield) as a pale-yellow oil after purification by column chromatography of the crude residue (cyclohexane/EtOAc 98/2 to 94/6). **Rf** = 0.43 (SiO_2, cyclohexane/EtOAc 8/2). **ATR FTIR** ν = 2929, 2851, 1605, 1507, 1441, 1284, 1245, 1209, 830, 699 cm^{-1}. **^{1}H NMR** (500 MHz, $CDCl_3$) δ 7.42-7.37 (m, 4H), 7.36-7.29 (m, 2H), 7.27-7.21 (m, 1H), 6.88-6.82 (m, 2H), 3.97 (s, 2H), 3.82 (s, 3H), 2.01-1.92 (m, 2H), 1.77-1.60 (m, 7H), 1.34-1.19 (m, 1H) ppm. **^{13}C NMR** (126 MHz, $CDCl_3$) δ 159.4, 141.3, 133.2, 128.6, 128.6, 127.0, 116.0, 114.0, 92.1, 84.6, 55.5, 55.5, 48.2, 38.4, 26.1, 23.2 ppm. **HRMS:** 320.2018 $[M+H]^+$, calcd 320.2009.

_N_-Benzyl-1-((3,5-bis(trifluoromethyl)phenyl)ethynyl)cyclohexan-1-amine (Ii).

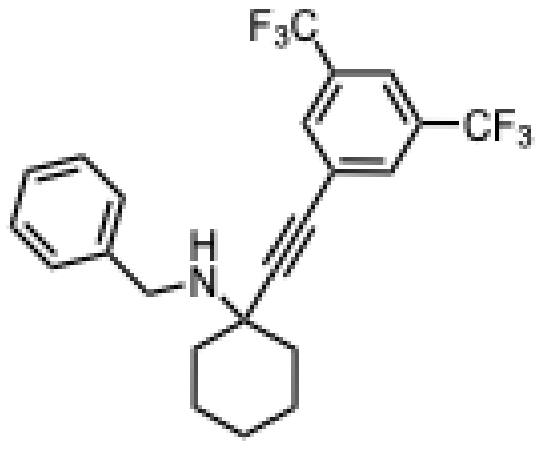

Ii
Chemical Formula: $C_{23}H_{21}F_6N$
Molecular Weight: 425,42

Following the **General Procedure A** starting from cyclohexanone (207 µL, 2.0 mmol), 1-ethynyl-3,5-bis(trifluoromethyl)benzene (425 µL, 2.4 mmol), Cu^{I}-USY (72 mg) and benzylamine (218 µL, 2.0 mmol), the title compound **Ii** was obtained (462 mg, 1.08 mmol, 54% yield) as a yellow solid after purification by column chromatography of the crude residue (cyclohexane/EtOAc 98/2 to 94/6). **Rf** = 0.54 (SiO_2, cyclohexane/EtOAc 8/2). **ATR FTIR** ν = 2934, 2855, 1614, 1461, 1379, 1276, 1131, 896, 683 cm^{-1}. **^{1}H NMR** (500 MHz, $CDCl_3$) δ 7.85-7.82 (bs, 2H) 7.80-7.77 (bs, 1H), 7.40-7.38 (m, 2H), 7.35-7.32 (m, 2H), 7.27-7.24 (m, 1H), 3.96 (s, 2H), 2.02-1.98 (m, 2H), 1.79-1.74 (m, 2H), 1.71-1.53 (m, 5H), 1.35-1.26 (m, 1H) ppm. **^{13}C NMR** (126 MHz, $CDCl_3$) δ 140.9, 132.0 (q, *J* = 33.6 Hz), 131.7 (d, *J* = 3.93 Hz), 128.6, 128.5, 127.2, 126.1, 121.6 (m), 97.9, 82.1, 55.4, 48.2, 38.1, 27.1, 25.6, 23.0 ppm. **^{19}F NMR** (282 MHz, $CDCl_3$) δ -63.1 ppm. **HRMS:** 426.1670 $[M+H]^+$, calcd 426.1651.

_N_-Benzyl-1-(thiophen-3-ylethynyl)cyclohexan-1-amine (Ij).

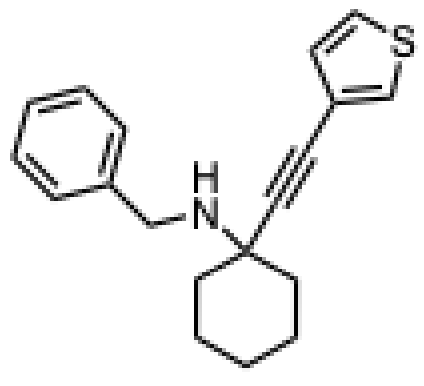

Ij
Chemical Formula: $C_{19}H_{21}NS$
Molecular Weight: 295,44

Following the **General Procedure A** starting from cyclohexanone (207 µL, 2.0 mmol), 3-ethynylthiophene (269 µL, 2.4 mmol), Cu^{I}-USY (72 mg) and benzylamine (218 µL, 2.0 mmol), the title compound **Ij** was obtained (501 mg, 1.70 mmol, 85% yield) as a pale-yellow oil after purification by column chromatography of the crude residue (cyclohexane/EtOAc 98/2 to 94/6). **Rf** = 0.36 (SiO_2, cyclohexane/EtOAc 8/2). **ATR FTIR** ν = 3313, 3085, 2928, 2851, 1449, 1335, 1114, 815, 715, 626 cm^{-1}. **^{1}H NMR** (500 MHz, $CDCl_3$) δ 7.43-7.37 (m, 3H), 7.35-7.30 (dd, *J* = 8.3, 6.9 Hz, 2H), 7.29-7.22 (m, 2H), 7.13 (dd, *J* = 5.0, 1.2 Hz, 1H), 3.96 (s, 2H), 2.01-1.93 (m, 2H), 1.76-1.62 (m, 5H), 1.53 (td, 11.9, 4.0 Hz, 2H), 1.32-1.29 (m, 1H) ppm. **^{13}C NMR** (126 MHz, $CDCl_3$) δ 141.2, 130.3, 128.6, 128.6, 128.0, 127.0, 125.3, 122.8, 93.2, 79.7, 55.5, 48.2, 38.2, 26.1, 23.1 ppm. **HRMS:** 296.1470 $[M+H]^+$, calcd 296.1467.

_N_-Benzyl-1-(phenylethynyl)cyclopentan-1-amine (Ik).

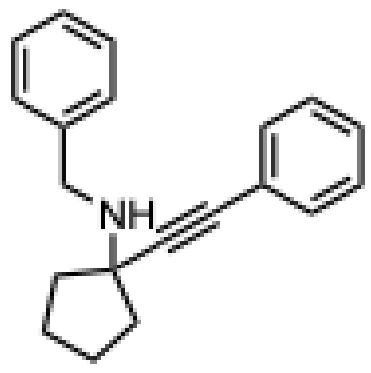

Ik
Chemical Formula: $C_{20}H_{21}N$
Molecular Weight: 275,39

Following de **general procedure A** starting from cyclopentanone (177 µL, 2.0 mmol), cyclohexylacetylene (265 µL, 2.4 mmol), Cu^{I}-USY (72 mg) and benzylamine (218 µL, 2.0 mmol), the title compound **Ik** was obtained (36 mg, 65.4 µmol, 7% yield) as a yellow oil after purification by column chromatography of the crude residue (cyclohexane/EtOAc 98/2 to 94/6). **Rf** = 0.52 (SiO_2, cyclohexane/EtOAc 8/2). **ATR FTIR** ν = 2958, 2867, 1597, 1489, 1452, 1196, 1069, 754, 691 cm^{-1}. **^{1}H NMR** (500 MHz, $CDCl_3$) δ 7.47-7.43 (m, 2H), 7.41-7.38 (m, 2H), 7.36-7.29 (m, 5H), 7.27-7.23 (m, 1H), 3.96 (s, 2H), 2.11-2.01 (m, 2H), 1.92-1.79 ppm. **^{13}C NMR** (126 MHz, $CDCl_3$) δ 141.1, 131.8, 128.6, 128.5, 128.4, 127.9, 127.0, 123.8, 94.6, 83.1, 61.4, 50.0, 40.9, 23.9 ppm. **HRMS:** 276.1734 $[M+H]^+$, calcd 276.1747.

_N_-Benzyl-1-(cyclohexylethynyl)cycloheptan-1-amine (Il).

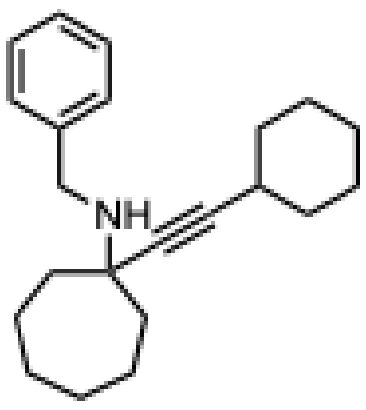

Il
Chemical Formula: $C_{22}H_{31}N$
Molecular Weight: 309,50

Cycloheptanone (117 µL, 1.0 mmol), benzylamine (109 µL, 1 mmol), dry DCM (0.5 mL) and molecular sieves 4 Å (72 mg) were successively added to a round bottom flask and stirred at room temperature. After completion, the mixture was filtrated over Celite and dried with $MgSO_4$. The filtrate was concentrated under reduced pressure and dry THF was added to the crude product. $BF_3{\cdot}OEt_2$ (209 µL, 2 mmol) was added to the solution of imine (0.1 M) and cooled down to −78 °C for 10 min. In another round bottom flask, *n*-BuLi (1.8 mL, 3.2 mmol, 1.6 M in hexane) was added to a solution of cyclohexylacetylene (331 µL, 3 mmol) in dry THF (0.5 M) at −78 °C. The solution of lithium acetylide is then added to the solution of activated imine and after 30 min, the mixture is progressively heated to room temperature for 2 h. The reaction mixture is then quenched with NH_4Cl (20 mL) and THF was removed under reduced pressure. The organic phase is extracted with DCM (3 × 20 mL) and then concentrated under reduced pressure. The title compound **Il** was obtained (61.7 mg, 0.20 mmol, 20% yield) as a yellow oil after purification by column chromatography of the crude residue (cyclohexane/EtOAc 98/2 to 94/6). **Rf** = 0.60 (SiO_2, cyclohexane/EtOAc 8/2). **ATR FTIR** ν = 2924, 2851, 1708, 1495, 1447, 1360, 1203, 1071, 1028, 728, 697 cm^{-1}. **^{1}H NMR** (500 MHz, $CDCl_3$) δ 7.38-7.35 (m, 2H), 7.33-7.29 (m, 2H), 7.25-7.22 (m, 1H), 3.86 (s, 2H), 2.51-2.44 (m, 1H), 1.91-1.78 (m, 5H), 1.77-1.70 (m, 2H), 1.70-1.60 (m, 9H), 1.58-1.44 (m, 6H), 1.40-1.33 (m, 2H) ppm. **^{13}C NMR** (126 MHz, $CDCl_3$) δ 141.5, 128.7, 128.5, 126.9, 88.3, 84.9, 58.1, 48.7, 41.2, 33.3, 29.1, 28.2, 26.2, 24.9, 22.8 ppm. **HRMS:** 310.2521 [M+H]+, calcd 310.2529.

***N*-Benzyl-1-(phenylethynyl)cycloheptan-1-amine (Im).**

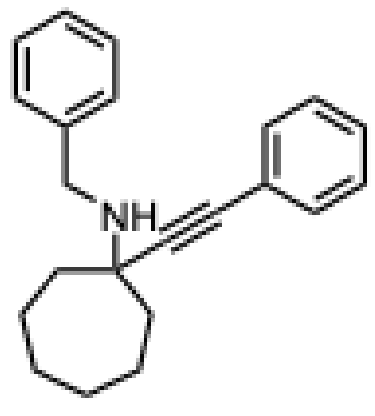

Im
Chemical Formula: $C_{22}H_{25}N$
Molecular Weight: 303,45

Following de **General Procedure A** starting from cycloheptanone (235 µL, 2.0 mmol), cyclohexylacetylene (265 µL, 2.4 mmol), Cu^{I}-USY (72 mg) and benzylamine (218 µL, 2.0 mmol), the title compound **Im** was obtained (248.2 mg, 0.82 mmol, 67% yield) as a yellow oil after purification by column chromatography of the crude residue (cyclohexane/EtOAc 98/2 to 94/6). **Rf** = 0.56 (SiO_2, cyclohexane/EtOAc 8/2). **ATR FTIR** ν = 2922, 2853, 1597, 1489, 1453, 1287, 1028, 754, 691 cm^{-1}. **^{1}H NMR** (500 MHz, $CDCl_3$) δ 7.48-7.44 (m, 2H), 7.42-7.38 (m, 2H), 7.35-7.29 (m, 5H), 7.27-7.22 (m, 1H), 3.96 (s, 2H), 2.07-1.99 (m, 2H), 1.841.56 (m, 10H) ppm. **^{13}C NMR** (126 MHz, $CDCl_3$) δ 141.3, 131.8, 128.6, 128.6, 128.4, 127.9, 127.0, 123.9, 94.9, 83.9, 58.5, 49.8, 40.9, 28.4, 22.8 ppm. **HRMS:** 304.2046 $[M+H]^+$, calcd 304.2060.

***N*-(4-Methoxybenzyl)-1-(phenylethynyl)cyclohexan-1-amine (Ip).**

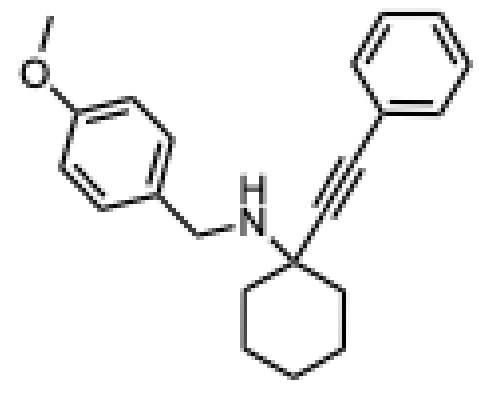

Ip
Chemical Formula: $C_{22}H_{25}NO$
Molecular Weight: 319,45

Following de **General Procedure A** starting from cyclohexanone (207 µL, 2.0 mmol), cyclohexylacetylene (265 µL, 2.4 mmol), Cu^{I}-USY (72 mg) and 4-methoxybenzylamine (261 µL, 2.0 mmol), the title compound **Ip** was obtained (493.4 mg, 1.5 mmol, 77% yield) as a yellow oil after purification by column chromatography of the crude residue (cyclohexane/EtOAc 98/2 to 94/6). **Rf** = 0. 33 (SiO_2, cyclohexane/EtOAc 8/2). **ATR FTIR** ν = 2996, 2929, 2852, 2834, 1611, 1598, 1585, 1510, 1442, 1172, 1034, 822 cm^{-1}. **^{1}H NMR** (500 MHz, $CDCl_3$) δ 7.48-7.45 (m, 2H), 7.34-7.29 (m, 5H), 6.88-6.84 (m, 2H), 3.92 (s, 2H), 3.80 (s, 3H), 2.01-1.93 (m, 2H), 1.77-1.62 (m, 5H), 1.52 (ddd, *J* = 12.6, 10.9, 4.4 Hz, 2H), 1.34-1.21 (m, 1H) ppm. **^{13}C NMR** (126 MHz, $CDCl_3$) δ 158.7, 133.4, 131.8, 129.8, 128.4, 127.9, 123.9, 114.0, 93.9, 84.8, 55.4, 47.6, 38.4, 26.1, 23.2 ppm. **HRMS:** 320.2005 $[M+H]^+$, calcd 320.2009.

1-(Cyclohexylethynyl)-*N*-(4-fluorobenzyl)cyclohexan-1-amine (Iq).

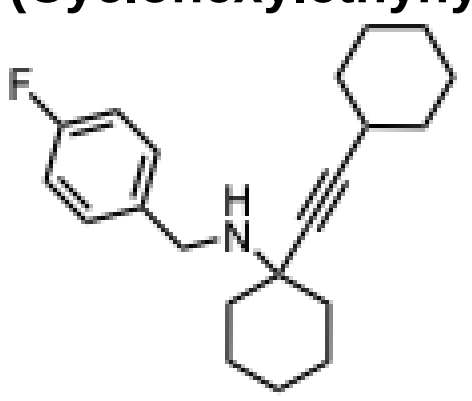

Iq
Chemical Formula: $C_{21}H_{28}FN$
Molecular Weight: 313,46

Following the **General Procedure A** starting from cyclohexanone (207 µL, 2.0 mmol), cyclohexylacetylene (265 µL, 2.4 mmol), Cu^{I}-USY (72 mg) and 4-fluorobenzylamine (229 µL, 2.0 mmol), the title compound **Iq** was obtained (124 mg, 0.40 mmol, 19% yield) as a yellow oil after purification by column chromatography of the crude residue (cyclohexane/EtOAc 98/2 to 94/6). **Rf** = 0.71 (SiO_2, cyclohexane/EtOAc 8/2). **ATR FTIR** ν = 2926, 2852, 1602, 1608, 1446, 1220, 1113, 824, 739 cm^{-1}. **^{1}H NMR** (500 MHz, $CDCl_3$) δ 7.38-7.35 (m, 2H), 6.99 (t, *J* = 8.7 Hz, 2H), 3.85 (s, 2H), 2.47 (tt, *J* = 8.6, 3.7, 1H), 1.88-1.13 (m, 20H) ppm. **^{13}C NMR** (126 MHz, $CDCl_3$) δ 162.0 (d, *J* = 244.3 Hz), 137.2 (d, *J* = 3.1 Hz), 130.2 (d, *J* = 7.9 Hz), 115.3 (d, *J* = 21.2 Hz), 89.3, 83.8, 55.0, 47.4, 38.7, 33.3, 29.1, 26.1, 24.9, 23.2 ppm. **^{19}F NMR** (282 MHz, $CDCl_3$) δ -116.5 ppm. **HRMS:** 314.2271 $[M+H]^+$, calcd 314.2279.

***N*-Allyl-1-(cyclohexylethynyl)cyclohexan-1-amine (Ir).**

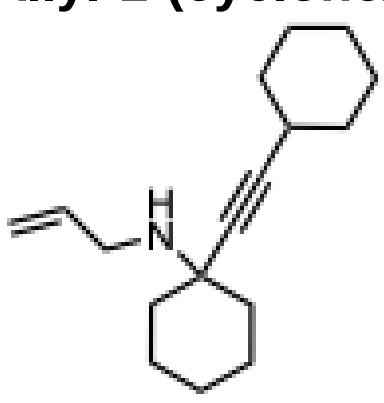

Ir
Chemical Formula: $C_{17}H_{27}N$
Molecular Weight: 245,41

Following the **General Procedure A** starting from cyclohexanone (207 µL, 2.0 mmol), cyclohexylacetylene (265 µL, 2.4 mmol), Cu^{I}-USY (72 mg) and benzylamine (218 µL, 2.0 mmol), the title compound **Ir** was obtained (239 mg, 0.97 mmol, 24% yield) as a pale-yellow oil after purification by column chromatography of the crude residue (cyclohexane/EtOAc 98/2 to 94/6). **Rf** = 0.45 (SiO_2, cyclohexane/EtOAc 8/2). **ATR FTIR** ν = 2932, 2852, 1643, 1447, 1346, 1282, 1117, 994, 747 cm^{-1}. **^{1}H NMR** (500 MHz, $CDCl_3$) δ 5.97 (ddt, *J* = 17.2, 10.2, 6.1 Hz, 1H), 5.19 (dq, *J* = 17.1, 1.7 Hz, 1H), 5.06 (dq, *J* = 10.2, 1.4 Hz, 1H), 3.36 (dt, *J* = 6.1, 1.5 Hz, 2H), 2.42 (dt, *J* = 6.6, 4.7 Hz, 1H), 1.82-1.73 (m, 4H), 1.72-1.66 (m, 2H), 1.65-1.58 (m, 4H), 1.51-1.40 (m, 3H), 1.37-1.28 (m, 5H), 1.19-1.11 (m, 1H) ppm. **^{13}C NMR** (126 MHz, $CDCl_3$) δ 137.7, 115.6, 89.3, 83.4, 54.7, 46.4, 38.7, 33.2, 29.1, 26.1, 26.1, 24.8, 23.3 ppm. **HRMS:** 246.2204 $[M+H]^+$, calcd 246.2216.

***N*-Butyl-1-(phenylethynyl)cyclohexan-1-amine (Is).**

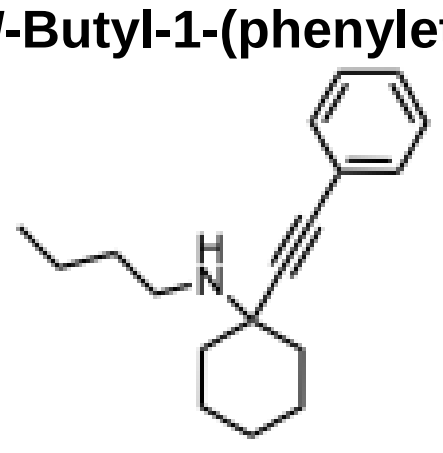

Is
Chemical Formula: $C_{18}H_{25}N$
Molecular Weight: 255,40

Following de **General Procedure A** starting from cyclohexanone (207 µL, 2.0 mmol), cyclohexylacetylene (265 µL, 2.4 mmol), Cu^{I}-USY (72 mg) and butylamine (199 µL, 2.0 mmol), the title compound **Is** was obtained (439 mg, 1.72 mmol, 86% yield) as a yellow oil after purification by column chromatography of the crude residue (cyclohexane/EtOAc 98/2 to 94/6). **Rf** = 0.29 (SiO_2, cyclohexane/EtOAc 8/2). **ATR FTIR** ν = 2928, 2853, 1597, 1488, 1459, 1443, 1289, 1126, 1069, 753, 690 cm^{-1}. **^{1}H NMR** (500 MHz, $CDCl_3$) δ 7.44-7.40 (m, 2H), 7.32-7.26 (m, 3H), 2.80 (t, *J* = 7.3 Hz, 2H), 1.98-1.89 (m, 2H), 1.75-1.60 (m, 5H), 1.55-1.36 (m, 6H), 1.29-1.20 (m, 1H), 0.94 (t, *J* = 7.3 Hz, 3H) ppm. **^{13}C NMR** (126 MHz, $CDCl_3$) δ 131.7, 128.3, 127.8, 123.9, 93.9, 84.5, 55.1, 43.1, 38.4, 33.0, 26.1, 23.2, 20.8, 14.2 ppm. **HRMS:** 256.2068 $[M+H]^+$, calcd 256.2060.

1.4 Preparation of 3-aza-1,5-enynes (**1**)

General procedure B: synthesis of aza-1,5-enynes (1)

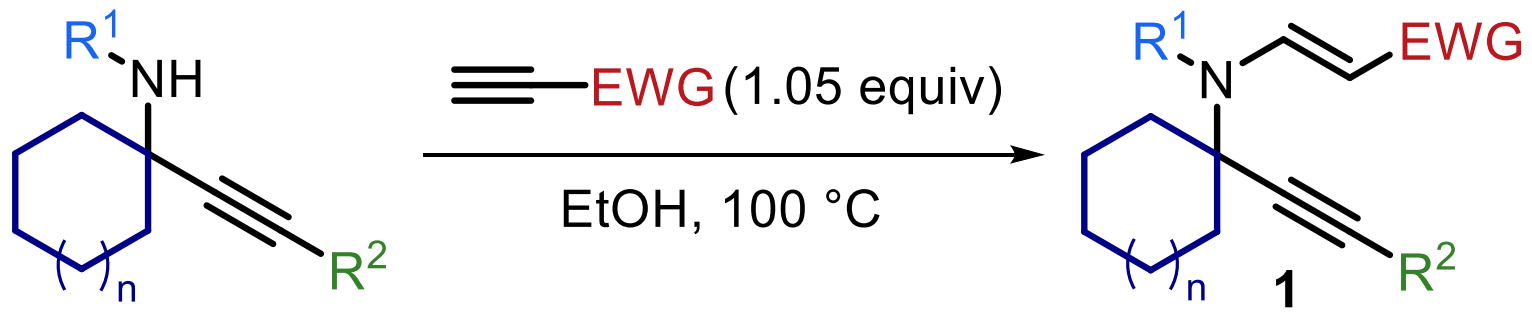

Activated alkyl propiolate (1.05 equiv) was added to a solution of propargylamine **(I)** (1.0 equiv) in EtOH (1 M) in a screw cap tube (3 mL) equipped with a stirring bar. The reaction flask was flushed with argon before sealing. Next, the mixture was placed into a pre-heated aluminium block at 100 °C and stirred until completion. All volatiles were removed under reduced pressure and the crude product was used without further purification unless otherwise stated.

Ethyl (*E*)-3(benzyl-(1-(cyclohexylethynyl)cyclohexyl)amino)acrylate (1a).

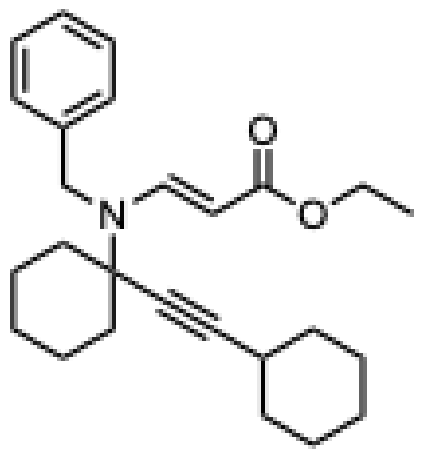

1a
Chemical Formula: $C_{26}H_{35}NO_2$
Molecular Weight: 393,57

Following **General Procedure B** starting from **Ia** (295.5 mg, 1 mmol), the title compound **1a** was obtained in quantitative yield as a pale-yellow solid. **Rf** = 0.46 (SiO_2, cyclohexane/EtOAc 8/2). **ATR FTIR** ν = 2928, 2853, 1686, 1604, 1448, 1328, 1120, 1105, 726 cm^{-1}. **^{1}H NMR** (500 MHz, $CDCl_3$) δ 8.17 (d, *J* = 12.9 Hz, 1H), 7.30-7.26 (m, 2H), 7.21-7.17 (m, 3H), 4.51 (s, 2H), 4.47 (d, *J* = 12.9 Hz, 1H), 4.04 (q, *J* = 7.1 Hz, 2H), 2.37 (m, 1H), 1.91 (d, 2H), 1.77-1.59 (m, 10H), 1.53-1.25 (m, 6H), 1.17 (t, *J* = 7.1 Hz, 3H), 1.16-1.05 (m, 1H), 0.86-0.78 (m, 1H) ppm. **^{13}C NMR** (126 MHz, $CDCl_3$) δ 170.0, 148.1, 137.6, 128.5, 126.8, 126.4, 93.3, 87.2, 79.9, 62.8, 59.0, 50.2, 37.8, 32.6, 29.1, 26.0, 25,3, 24.8, 24.0, 14,7 ppm. **HRMS:** 394.2748 [M+H]$^+$, calcd 394.2741.

Ethyl (*E*)-3-(benzyl(1-(cyclopropylethynyl)cyclohexyl)amino)acrylate (1b).

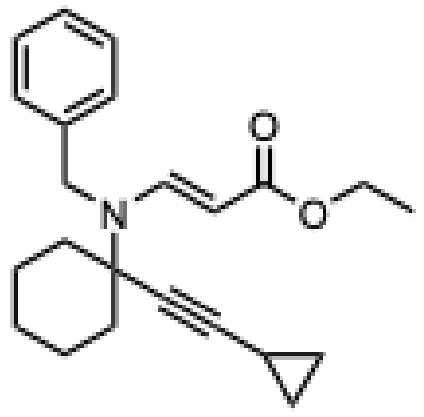

1b
Chemical Formula: $C_{23}H_{29}NO_2$
Molecular Weight: 351,49

Following **General Procedure B** starting from **Ib** (90.7 mg, 0.36 mmol), the title compound **1b** was obtained in quantitative yield as a pale-yellow solid. **Rf** = 0.44 (SiO_2, cyclohexane/EtOAc 8/2). **ATR FTIR** ν = 2932, 1683, 1626, 1443, 1327, 1290, 1138, 973, 793 cm^{-1}. **^{1}H NMR** (500 MHz, $CDCl_3$) δ 8.16 (d, *J* = 12.9, 1H), 7.31-7.26 (m, 2H) , 7.22-7.15 (m, 3H), 4.52 (s, 2H), 4.49 (d, *J* = 12.9 Hz, 1H), 4.08 (q, *J* = 7.1 Hz, 2H), 1.96-1.90 (m, 2H), 1.75-1.58 (m, 8H), 1.21 (t, *J* = 7.1 Hz, 3H), 1.13-1.05 (m, 1H), 0.74-0.69 (m, 2H), 0.56-0.52 (m, 2H) ppm. **^{13}C NMR** (126 MHz, $CDCl_3$) δ 170.0, 148.0, 137.6, 128.5, 128.6, 126.3, 92.0, 87.3, 75.0, 62.7, 59.1, 50.2, 37.7, 25.2, 23.9, 14.7, 8.4, 0.4 ppm. **HRMS:** 374.2091 [M+Na]$^+$, calcd 374.2091.

Ethyl (E)-3-(benzyl(1-oct-1-yn-1-yl)cyclohexyl)amino)acrylate (1c).

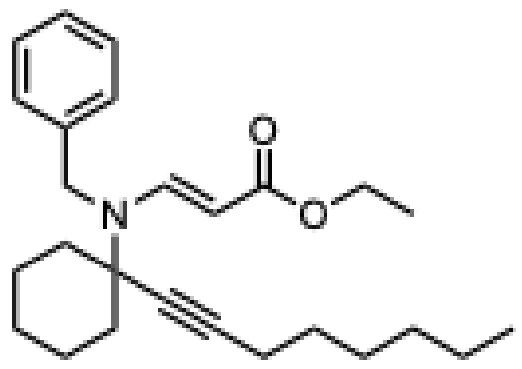

1c
Chemical Formula: $C_{26}H_{37}NO_2$
Molecular Weight: 395,59

Following **General Procedure B** starting from **Ic** (298 mg, 1 mmol), the title compound **1c** was obtained in quantitative yield as a pale-yellow oil. **Rf** = 0.58 (SiO_2, cyclohexane/EtOAc 8/2). **ATR FTIR** ν = 2930, 2857, 1687, 1559, 1328, 1139, 1106, 726 cm^{-1}. **^{1}H NMR** (500 MHz, $CDCl_3$) δ 8.18 (d, *J* = 13.0 Hz, 2H), 7.30-7.26 (m, 2H), 7.22-7.17 (m, 3H), 4.55 (s, 2H), 4.51 (d, *J* = 12.9 Hz, 2H), 4.08 (q, *J* = 7.1 Hz, 2H), 2.19 (t, *J* = 7.0 Hz, 2H), 1.99-1.93 (m, 2H), 1.75-1.59 (m, 7H), 1.37-1.24 (m, 6H), 1.21 (t, *J* = 7.1 Hz, 3H), 1.15-1.06 (m, 1H), 0.88 (t, *J* = 7.0 Hz, 3H) ppm. **^{13}C NMR** (126 MHz, $CDCl_3$) δ 170.0, 148.0, 137.6, 128.5, 126.8, 126.3, 88.9, 87.3, 80.0, 62.7, 59.0, 37.7, 31.4, 28.7, 28.7, 25.2, 23.9, 22.7, 18.8, 14.7, 14.2 ppm. **HRMS:** 418.2696 [M+Na]$^+$, calcd 418.2717.

Ethyl (*E*)-3-(benzyl(1-(5-phenylpent-1-yn-1-yl)cyclohexyl)amino)acrylate (1d).

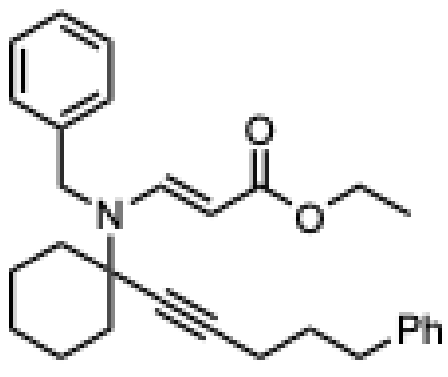

1d
Chemical Formula: $C_{29}H_{35}NO_2$
Molecular Weight: 429,60

Following **General Procedure B** starting from **Id** (88 mg, 0.26 mmol), the title compound **1d** was obtained in quantitative yield as a pale-yellow oil. **Rf** = 0.48 (SiO_2, cyclohexane/EtOAc 8/2). **ATR FTIR** ν = 2931, 2857, 1686, 1590, 1452, 1328, 1142, 1107, 728, 698 cm^{-1}. **^{1}H NMR** (500 MHz, $CDCl_3$) δ 8.20 (d, *J* = 12.9 Hz, 1H), 7.30-7.26 (m, 4H), 7.22-7.17 (m, 4H), 7.16-7.12 (m, 2H), 4.56 (s, 2H), 4.52 (d, *J* = 12.9 Hz, 1H), 2.67 (dd, *J* = 8.4 ; 6.8 Hz, 2H), 2.22 (t, *J* = 7.0 Hz, 2H), 2.02-1.95 (m, 2H), 1.80-1.62 (m, 9H), 1.21 (t, *J* = 7.1 Hz, 3H), 1.16-1.08 (m, 1H) ppm. **^{13}C NMR** (126 MHz, $CDCl_3$) δ 170.0, 148.0, 141.6, 137.6, 128.6, 128.6, 128.6, 126.9, 126.3, 126.1, 88.3, 87.5, 80.6, 62.7, 59.1, 50.3, 37.7, 35.0, 30.5, 25.2, 23.9, 18.3, 14.7 ppm. **HRMS:** 452.2541 [M+Na]$^+$, calcd 452.2560.

Ethyl (*E*)-3-(benzyl(1-(3-hydroxy-3-methylbut-1-yn-1-yl)cyclohexyl)amino)acrylate (1e).

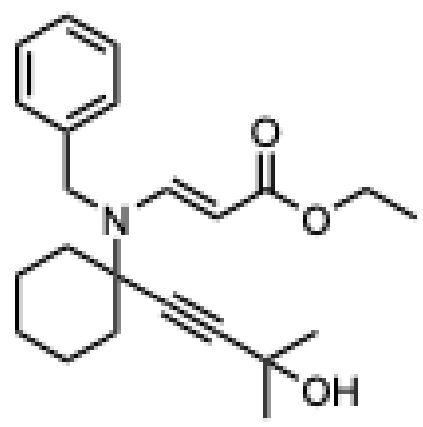

1e
Chemical Formula: $C_{23}H_{31}NO_3$
Molecular Weight: 369,50

Following **General Procedure B** starting from **Ie** (50.2 mg, 0.19 mmol), the title compound **1e** was obtained in quantitative yield as a pale-yellow solid. **Rf** = 0.10 (SiO_2, cyclohexane/EtOAc 8/2). **ATR FTIR** ν = 3377, 2979, 2933, 2858, 1669, 1594, 1452, 1329, 1261, 1147, 1106, 794, 728, 695 cm^{-1}. **^{1}H NMR** (500 MHz, $CDCl_3$) δ 8.15 (d, *J* = 13.0 Hz, 1H), 7.32-7.28 (m, 2H), 7.23-7.17 (m, 3H), 4.54 (d, J = 13.0 Hz, 1H), 4.54 (s, 2H), 4.08 (q, J = 7.1 Hz, 2H), 2.03-1.98 (m, 2H), 1.77-1.63 (m, 8H), 1.42 (s, 6H), 1.21 (t, *J* = 7.1 Hz, 3H) ppm. **^{13}C NMR** (126 MHz, $CDCl_3$) δ 170.0, 147.7, 137.4, 128.6, 127.0, 126.2, 93.1, 87.6, 81.9, 65.2, 62.4, 59.2, 50.3, 37.4, 31.4, 25.2, 23.9, 14.7 ppm. **HRMS:** 392.2174 [M+Na]$^+$, calcd 392.2196.

Ethyl (*E*)-3-(benzyl(1-(phenylethynyl)cyclohexyl)amino)acrylate (1f).

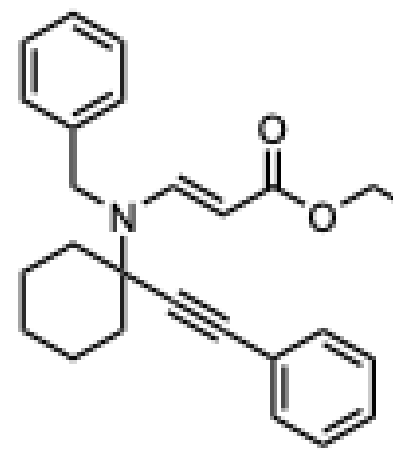

1f
Chemical Formula: $C_{26}H_{29}NO_2$
Molecular Weight: 387,52

Following **General Procedure B** starting from **If** (290.7 mg, 1 mmol), the title compound **1f** was obtained in quantitative yield as a pale-brown solid. **Rf** = 0.39 (SiO_2, cyclohexane/EtOAc 8/2). **ATR FTIR** ν = 2933, 2855, 1679, 1626, 1451, 1329, 1288, 1138, 654 cm^{-1}. **^{1}H NMR** (500 MHz, $CDCl_3$) δ 8.22 (d, *J* = 12.9 Hz, 1H), 7.33-7.26 (m, 7H), 7.25-7.20 (m, 3H), 4.63 (s, 2H), 4.58 (d, *J* = 12.9 Hz, 1H), 4.09 (q, *J* = 7.1 Hz, 2H), 2.16-2.10 (m, 2H), 1.85-1.69 (m, 8H), 1.22 (t, *J* = 7.2, 3H) ppm. **^{13}C NMR** (126 MHz, $CDCl_3$) δ 169.9, 147.7, 137.5, 131.7, 128.6, 128.6, 128.4, 126.9, 126.4, 122.6, 89.2, 88.2, 87.9, 63.0, 59.1, 50.5, 37.7, 25.2, 23.9, 14.7 ppm. **HRMS:** 410.2107 [M+Na]$^+$, calcd 410.2090.

Ethyl (*E*)-3-(benzyl(1-((4-(*tert*-butyl)phenyl)ethynyl)cyclohexyl)amino)acrylate (1g).

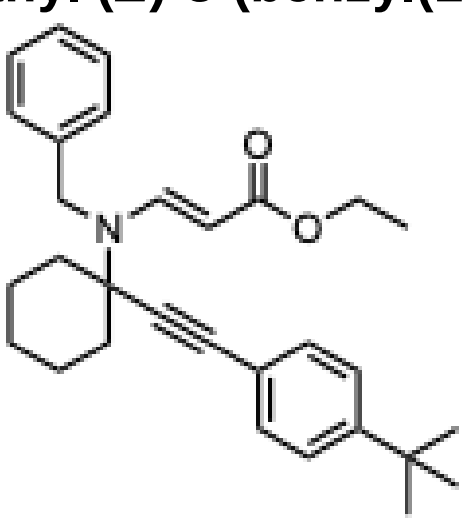

1g
Chemical Formula: $C_{30}H_{37}NO_2$
Molecular Weight: 443,63

Following **General Procedure B** starting from **Ig** (236 mg, 0.68 mmol), the title compound **1g** was obtained in quantitative yield as a pale-yellow oil. **Rf** = 0.42 (SiO_2, cyclohexane/EtOAc 8/2). **ATR FTIR** ν = 2934, 2860, 1684, 1626, 1328, 1208, 1106, 730 cm^{-1}. **^{1}H NMR** (500 MHz, $CDCl_3$) δ 8.21 (d, *J* = 13.0 Hz, 1H), 7.32-7.28 (m, 4H), 7.27-7.21 (m, 5H), 4.63 (s, 2H), 4.57 (d, *J* = 13.0 Hz, 1H), 4.09 (q, *J* = 7.1 Hz, 2H), 2.13 (m, 2H), 1.82-1.68 (m, 8H), 1.30 (s, 9H), 1.22 (t, *J* = 7.1 Hz, 3H) ppm. **^{13}C NMR** (126 MHz, $CDCl_3$) δ 169.9, 151.9, 147.7, 137.5, 131.5, 128.6, 126.9, 126.4, 125.4, 119.6, 88.5, 88.3, 87.8, 63.1, 59.1, 50.6, 37.7, 34.9, 31.3, 25.2, 23.9, 14.7 ppm. **HRMS:** 466.2704 [M+Na]$^+$, calcd 466.2717.

Ethyl (*E*)-3-(benzyl(1-((4-methoxyphenyl)ethynyl)cyclohexyl)amino)acrylate (1h).

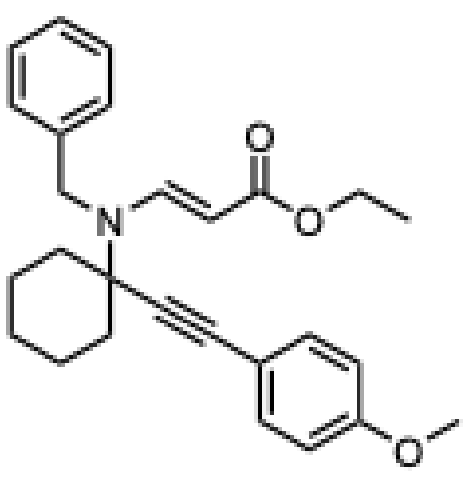

1h
Chemical Formula: $C_{27}H_{31}NO_3$
Molecular Weight: 417,55

Following **General Procedure B** starting from **Ih** (90.5 mg, 0.28 mmol), the title compound **1h** was obtained in quantitative yield as a pale-yellow oil. **Rf** = 0. (SiO_2, cyclohexane/EtOAc 8/2). **ATR FTIR** ν = 2933, 2857, 1683, 1626, 1509, 1452, 1226, 1106, 731 cm^{-1}. **^{1}H NMR** (500 MHz, $CDCl_3$) δ 8.23 (d, *J* = 13.0 Hz, 1H), 7.33-7.28 (m, 2H), 7.26-7.21 (m, 5H), 6.80 (d, *J* = 8.8 Hz, 2H), 4.62 (s, 2H), 4.57 (d, *J* = 12.9 Hz, 1H), 4.09 (q, *J* = 7.1 Hz, 2H), 3.80 (s, 3H), 2.14-2.08 (m, 2H), 1.87-1.71 (m, 7H), 1.22 (t, *J* = 7.2 Hz, 3H), 1.19-1.13 (m, 1H) ppm. **^{13}C NMR** (126 MHz, $CDCl_3$) δ 169.9, 159.8, 147.8, 137.5, 133.1, 128.6, 126.0, 126.4, 114.7, 114.0, 88.1, 87.7, 87.6, 63.1, 59.1, 55.4, 50.5, 37.7, 25.2, 23.9, 14.7 ppm. **HRMS:** 418.2366 [M+H]$^+$, calcd 418.2377.

Ethyl (*E*)-3-(enzyl(1-((3,5-bis(trifluoromethyl)phenyl)ethynyl)cyclohexyl)amino)acrylate (1i).

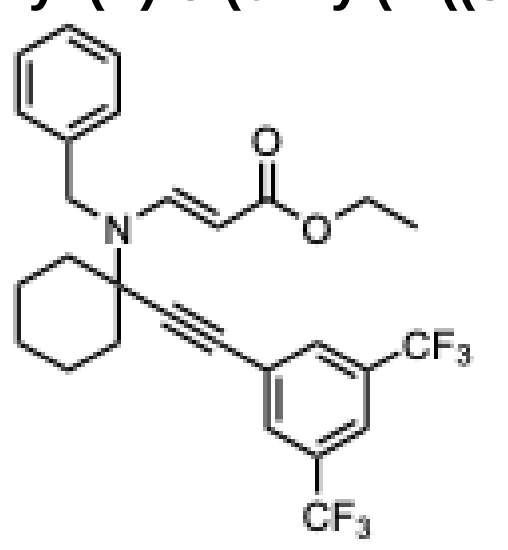

1i
Chemical Formula: $C_{28}H_{27}F_6NO_2$
Molecular Weight: 523,52

Following **General Procedure B** starting from **Ii** (302 mg, 0.70 mmol), the title compound **1i** was obtained in quantitative yield as a pale-yellow oil. **Rf** = 0.42 (SiO_2, cyclohexane/EtOAc 8/2). **ATR FTIR** ν = 2937, 1687, 1600, 1380, 1268, 1110, 987, 682 cm^{-1}. ^{1}**H NMR** (500 MHz, $CDCl_3$) δ 8.14 (d, *J* = 13.0 Hz, 1H), 7.79-7.75 (bs, 1H), 7.57-7.55 (m, 2H), 7.34-7.29 (bs, 2H), 7.26-7.20 (m, 3H), 4.63 (d, *J* = 13.0 Hz, 1H), 4.62 (s, 2H), 4.10 (q, *J* = 7.1 Hz, 2H), 2.23-2.17 (m, 1H), 1.89-1.68 (m, 7H), 1.12 (t, *J* = 7.1 HZ, 3H), 1.20-1.16 (m, 1H) ppm. ^{13}**C NMR** (126 MHz, $CDCl_3$) δ 169.8, 147.1, 137.2, 132.0 (q, *J* = 33.8 Hz), 131.6 (d, *J* = 3.2 Hz), 128.7, 127.2, 126.2, 124.8, 124.1, 122.0 (m), 93.1, 88.5, 84.7, 62.6, 59.3, 50.7, 37.4, 25.1, 23.9, 14.7 ppm. ^{19}**F NMR** (282 MHz, $CDCl_3$) δ -63.1 ppm **HRMS:** 546.1833 $[M+Na]^+$, calcd 546.1838.

Ethyl (*E*)-3-(benzyl(1-(thiophen-3-ylethynyl)cyclohexyl)amino)acrylate (1j).

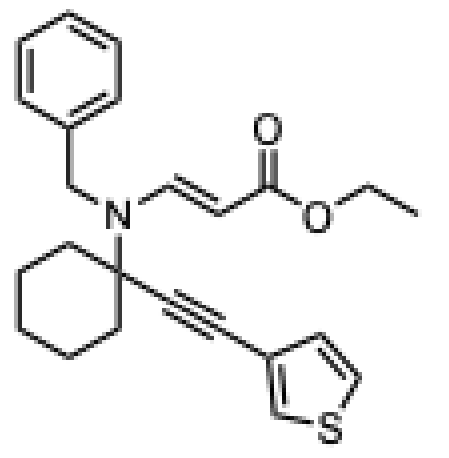

1j
Chemical Formula: $C_{24}H_{27}NO_2S$
Molecular Weight: 393,55

Following **General Procedure B** starting from **Ij** (178 mg, 0.60 mmol), the title compound **1j** was obtained in quantitative yield as a pale-brown oil. **Rf** = 0.43 (SiO_2, cyclohexane/EtOAc 8/2). **ATR FTIR** ν = 2976, 2932, 2857, 1683, 1596, 1451, 1327, 1241, 1139, 963, 782, 694 cm^{-1}. ^{1}**H NMR** (500 MHz, $CDCl_3$) δ 8.20 (d, *J* = 13.0 Hz, 1H), 7.32-7.28 (m, 3H), 7.24-7.20 (m, 4H), 6.98 (dd, *J* = 5.0, 1.2 Hz, 1H), 4.59 (s, 2H), 4.58 (d, *J* = 13.0 Hz, 1H), 4.09 (q, *J* = 7.2 Hz, 2H), 2.15-2.08 (m, 2H), 1.83-1.68 (m, 7H), 1.21 (t, *J* = 7.1 Hz, 3H), 1.19-1.12 (m, 1H) ppm. ^{13}**C NMR** (126 MHz, $CDCl_3$) δ 170.0, 147.7, 137.4, 129.9, 129.0, 128.6, 126.9, 126.3, 125.5, 121.5, 88.7, 87.7, 83.1, 63.0, 59.1, 50.5, 37.6, 25.2, 23.9, 14.7 ppm. **HRMS:** 394.1836 $[M+H]^+$, calcd 394.1835.

Ethyl (*E*)-3-(benzyl(1-(phenylethynyl)cyclopentyl)amino)acrylate (1k).

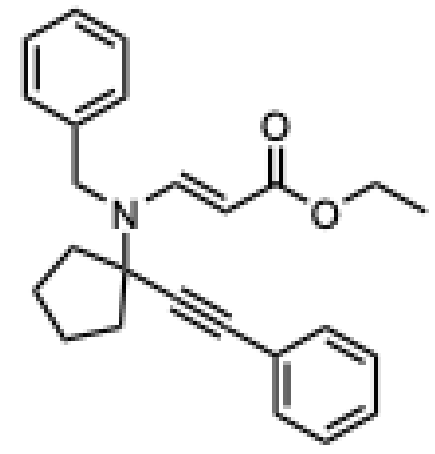

1k
Chemical Formula: $C_{25}H_{27}NO_2$
Molecular Weight: 373,50

Following **General Procedure B** starting from **Ik** (34.3 mg, 0.12 mmol), the title compound **1k** was obtained in quantitative yield as an orange oil. **Rf** = 0.42 (SiO_2, cyclohexane/EtOAc 8/2). **ATR FTIR** ν = 2950, 2928, 2861, 1687, 1601, 1490, 1452, 1244, 1145, 1043, 756, 728, 692 cm^{-1}. ^{1}**H NMR** (500 MHz, $CDCl_3$) δ 8.02 (d, *J* = 13.1 Hz, 1H), 7.36-7.33 (m, 2H), 7.32-7.28 (m, 5H), 7.25-7.21 (m, 3H), 4.62 (d, *J* = 13.1 Hz, 1H), 4.56 (s, 2H), 4.10 (q, *J* = 7.1 Hz, 2H), 2.32-2.26 (m, 2H), 2.11-2.04 (m, 2H),1.97-1.91 (m, 2H), 1.87-1.81 (m, 2H), 1.22 (t, *J* = 7.1 Hz, 3H) ppm. ^{13}**C NMR** (126 MHz, $CDCl_3$) δ 169.7, 149.5, 137.4, 131.8, 128.7, 128.5, 128.4, 127.1, 126.3, 122.8, 91.2, 88.8, 84.4, 67.9, 59.2, 52.1, 40.0, 22.8, 14.7 ppm. **HRMS:** 396.1931 $[M+Na]^+$, calcd 396.1934.

Ethyl (*E*)-3-(benzyl(1-(cyclohexylethynyl)cycloheptyl)amino)acrylate (1l).

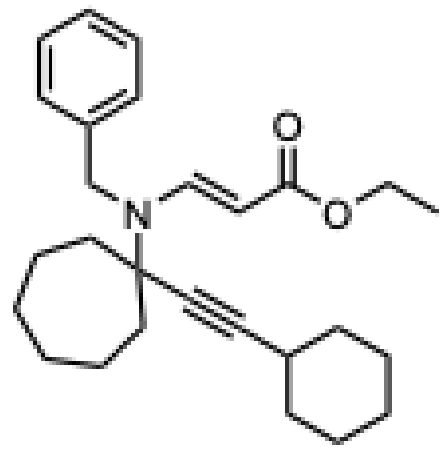

1l
Chemical Formula: $C_{27}H_{37}NO_2$
Molecular Weight: 407,60

Following **General Procedure B** starting from **Il** (48.8 mg, 0.16 mmol), the title compound **1l** was obtained in quantitative yield as a pale-yellow oil. **Rf** = 0.35 (SiO_2, cyclohexane/EtOAc 8/2). **ATR FTIR** ν = 2927, 2853, 1689, 1601, 1449, 1330, 1241, 1146, 1096, 712 cm^{-1}. ^{1}**H NMR** (500 MHz, $CDCl_3$) δ 8.20 (d, *J* = 13.0 Hz, 1H), 7.30-7.26 (m, 2H), 7.21-7.18 (m, 3H), 4.53 (s, 2H), 4.46 (d, *J* = 13.0 Hz, 1H), 4.07 (q, *J* = 7.1 Hz, 2H), 2.42-2.35 (m, 1H), 2.05-1.98 (m, 2H), 1.93 (ddd, *J* = 13.5, 10.2, 3.0 Hz, 2H), 1.65-1.58 (m, 10H), 1.57-1.50 (m, 2H), 1.38-1.25 (m, 6H), 1.22 (t, *J* = 7.1 Hz, 3H) ppm. ^{13}**C NMR** (126 MHz, $CDCl_3$) δ 169.9, 148.4, 137.6, 128.5, 126.8, 126.3, 3.0, 87.5, 80.5, 66.1, 59.0, 50.7, 40.9, 32.5, 29.1, 27.2, 26.0, 24.8, 23.4, 14.7 ppm. **HRMS:** 408.2885 $[M+H]^+$, calcd 408.2897.

Ethyl (*E*)-3-(benzyl(1-(phenylethynyl)cycloheptyl)amino)acrylate (1m).

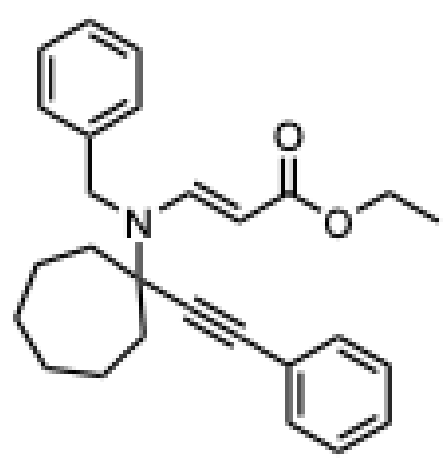

1m
Chemical Formula: $C_{27}H_{31}NO_2$
Molecular Weight: 401,55

Following **General Procedure B** starting from **Im** (303.5 mg, 1 mmol), the title compound **1m** was obtained in quantitative yield as an orange oil **Rf** = 0.57 (SiO_2, cyclohexane/EtOAc 8/2). **ATR FTIR** ν = 2931, 2859, 1678, 1595, 1444, 1334, 1292, 1152, 1095, 965, 756, 724, 693 cm^{-1}. ^{1}**H NMR** (500 MHz, $CDCl_3$) δ 8.21 (d, *J* = 12.9 Hz, 1H), 7.33-7.26 (m, 4H), 7.26-7.20 (m, 6H), 4.61 (s, 2H), 4.55 (d, *J* = 13.0 Hz, 1H), 4.09 (q, *J* = 7.1 Hz, 2H), 2.19 (ddt, *J* = 14.0, 6.9, 1.7 Hz, 2H), 2.06 (ddd, *J* = 13.7, 10.3, 3.0 Hz, 2H), 1.87-1.68 (m, 6H), 1.63-1.55 (m, 2H), 1.22 (t, *J* = 7.1 Hz, 3H) ppm. ^{13}**C NMR** (126 MHz, $CDCl_3$) δ 169.9, 148.1, 137.5, 131.6, 128.6, 128.6, 128.4, 126.9, 126.3, 122.6, 89.9, 88.1, 87.8, 66.3, 59.1, 51.2, 40.8, 27.4, 23.4, 14.7 ppm. **HRMS:** 402.2438 $[M+H]^+$, calcd 402.2428.

(*E*)-*N*-Benzyl-1-(cyclohexylethynyl)-*N*-(2-tosylvinyl)cyclohexan-1-amine (1n).

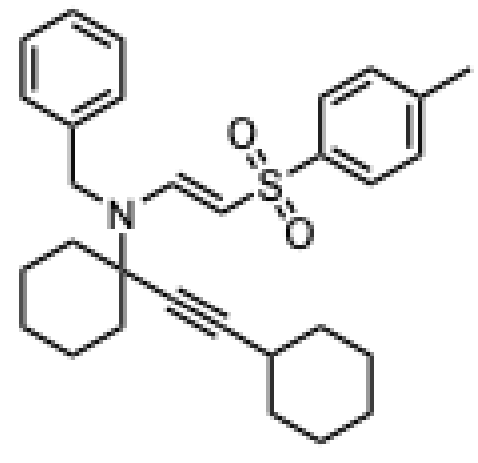

1n
Chemical Formula: $C_{30}H_{37}NO_2S$
Molecular Weight: 475,69

Following **General Procedure B** starting from **Ia** (113.3 mg, 0.38 mmol), the title compound **1n** was obtained in quantitative yield as a pale-yellow solid. **Rf** = 0.32 (SiO_2, cyclohexane/EtOAc 8/2). **ATR FTIR** ν = 3080, 3060, 2928, 2853, 1600, 1448, 1326, 1283, 1133, 1082, 876, 733, 707, 660, 539, 559 cm^{-1}. **^{1}H NMR** (500 MHz, $CDCl_3$) δ 8.06 (d, *J* = 12.7 Hz, 1H), 7.57 (d, *J* = 8.3 Hz, 2H), 7.27-7.22 (m, 2H), 7.21-7.11 (m, 5H), 4.82 (d, *J* = 12.7 Hz, 1H), 4.51 (s, 2H), 2.42 (td, *J* = 6.1, 5.2, 3.3 Hz, 1H), 2.37 (s, 3H), 1.99-1.93 (m, 2H), 1.78-1.59 (m, 12H), 1.42-1.36 (m, 2H), 1.33-1.29 (m, 2H), 1.17-1.06 (m, 1H) ppm. **^{13}C NMR** (126 MHz, $CDCl_3$) δ 146.3, 142.5, 142.0, 136.6, 129.4, 128.6, 127.1, 126.3, 126.1, 95.4, 93.8, 79.5, 63.1, 53.6, 50.4, 37.7, 32.6, 29.0, 26.0, 25.2, 23.9, 21.6 ppm. **HRMS:** 476.2607 $[M+H]^+$, calcd 476.2682.

(*E*)-*N*-Benzyl-1-(phenylethynyl)-*N*-(2-tosylvinyl)cyclohexan-1-amine (1o).

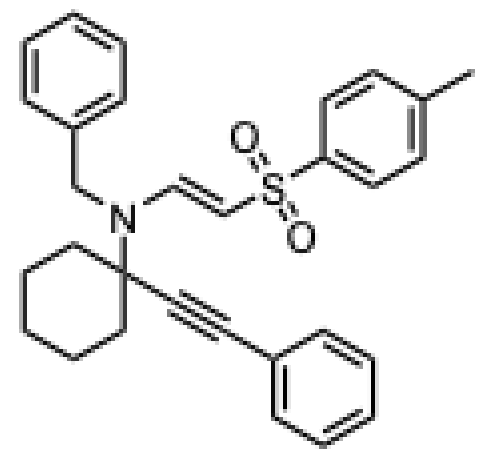

1o
Chemical Formula: $C_{30}H_{31}NO_2S$
Molecular Weight: 469,64

Following **General Procedure B** starting from **If** (289.4 mg, 1 mmol), the title compound **1o** was obtained (372.5 mg, 7.9 mmol, 79% yield) as a white solid after purification by column chromatography of the crude residue (cyclohexane/EtOAc 98/2 to 94/6). **Rf** = 0.27 (SiO_2, cyclohexane/EtOAc 8/2). **ATR FTIR** ν = 3060, 3029, 2930, 2857, 1598, 1491, 1451, 1327, 1280, 1131, 1080, 874, 691, 655, 556, 536 cm^{-1}. **^{1}H NMR** (500 MHz, $CDCl_3$) δ 8.09 (d, *J* = 12.6 Hz, 1H), 7.59 (d, *J* = 8.3 Hz, 2H), 7.36-7.24 (m, 7H), 7.23-7.15 (m, 5H), 4.91 (d, *J* = 12.6 Hz, 1H), 4.60 (s, 2H), 2.37 (s, 3H), 2.19-2.11 (m, 2H), 1.84-1.72 (m, 7H), 1.21-1.19 (m, 1H) ppm. **^{13}C NMR** (126 MHz, $CDCl_3$) δ 145.9, 142.2, 142.1, 136.5, 131.7, 129.4, 128.7, 128.7, 128.5, 127.2, 126.3, 126.2, 122.3, 96.1, 88.6, 88.6, 63.3, 50.8, 37.6, 25.1, 23.9, 21.6 ppm. **HRMS:** 470.2138 $[M+H]^+$, calcd 470.2148.

Ethyl (*E*)-3-((4-methoxybenzyl)(1-(phenylethynyl)cyclohexyl)amino)acrylate (1p).

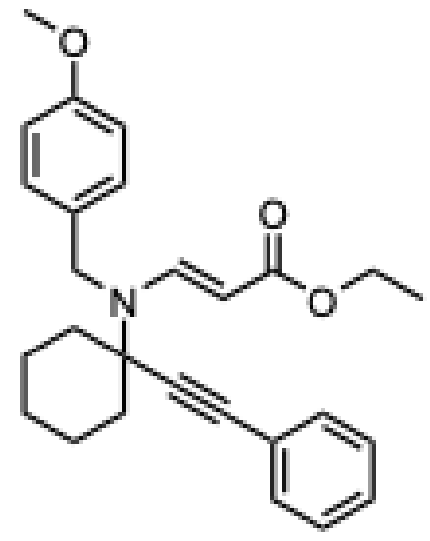

1p
Chemical Formula: $C_{27}H_{31}NO_3$
Molecular Weight: 417,55

Following **General Procedure B** starting from **Ip** (303.5 mg, 1 mmol), the title compound **1p** was obtained in quantitative yield as an orange oil. **Rf** = 0.40 (SiO_2, cyclohexane/EtOAc 8/2). **ATR FTIR** ν = 3059, 2933, 2858, 2836, 1685, 1597, 1328, 1139, 1099, 755, 691 cm^{-1}. **^{1}H NMR** (500 MHz, $CDCl_3$) δ 8.20 (d, *J* = 13.0 Hz, 1H), 7.36-7.33 (m, 2H), 7.32-7.28 (m, 3H), 7.18-7.14 (m, 2H), 6.86-6.82 (m, 2H), 4.60 (d, *J* = 13.0 Hz, 1H), 4.57 (s, 2H), 4.10 (q, *J* = 7.1 Hz, 2H), 3.79 (s, 3H), 2.15-2.08 (m, 2H), 1.89-1.63 (m, 7H), 1.22 (t, J = 7.1 Hz, 3H), 1.20-1.13 (m, 1H) ppm. **^{13}C NMR** (126 MHz, $CDCl_3$) δ 170.0, 158.6, 147.7, 131.7, 129.5, 128.6, 128.4, 127.5, 122.6, 114.1, 89.2, 88.2, 87.8, 63.0, 59.1, 55.4, 50.0, 37.7, 25.2, 23.9, 14.7 ppm. **HRMS:** 440.2193 $[M+Na]^+$, calcd 440.2196.

Ethyl (*E*)-3-((1-(cyclohexylethynyl)cyclohexyl)(4-fluorobenzyl)amino)acrylate (1q).

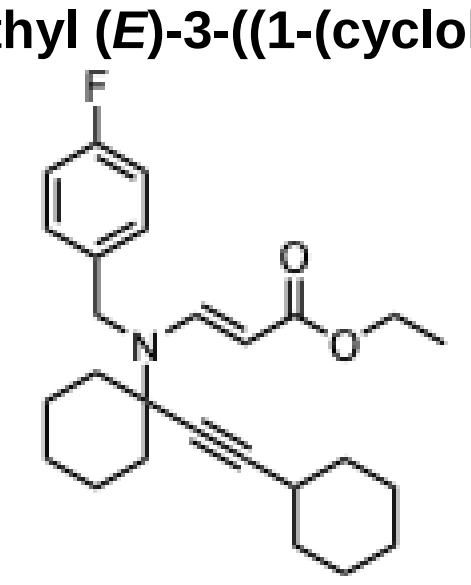

1q
Chemical Formula: $C_{26}H_{34}FNO_2$
Molecular Weight: 411,56

Following **General Procedure B** starting from **Iq** (345 mg, 1.10 mmol), the title compound **1q** was obtained in quantitative yield as a pale-yellow solid. **Rf** = 0.46 (SiO_2, cyclohexane/EtOAc 8/2). **ATR FTIR** ν = 2929, 2854, 1683, 1608, 1508, 1328, 1222, 1107, 1093, 732 cm^{-1}. **^{1}H NMR** (500 MHz, $CDCl_3$) δ 8.16 (d, *J* = 13.0 Hz, 1H), 7.18-7.13 (m, 2H), 6.97 (t, *J* = 8.7 Hz, 2H), 4.51 (s, 2H), 4.47 (d, *J* = 13.0 Hz, 1H), 4.08 (q, *J* = 7.1 Hz, 2H), 2.44-2.37 (m, 1H), 1.96-1.91 (m, 2H), 1.76-1.57 (m, 12H), 1.42-1.25 (m, 5H), 1.21 (t, *J* = 7.1 Hz, 1H), 1.15-1.06 (m, 1H) ppm. **^{13}C NMR** (126 MHz, $CDCl_3$) δ 169.9, 161.8 (d, *J* = 244.5 Hz), 141.7, 133.2 (d, *J* = 3.1 Hz), 127.9 (d, *J* = 7.9 Hz), 115.4 (d, *J* = 21.5 Hz), 93.3, 87.5, 79.8, 62.8, 59.1, 49.6, 37.8, 32.6, 29.0, 26.0, 25.2, 24.8, 23.9, 14.7 ppm. **^{19}F NMR** (282 MHz, $CDCl_3$) δ -116.5 ppm. **HRMS:** 434.2454 $[M+Na]^+$, calcd 434.2466.

Ethyl (*E*)-3-(allyl(1-(cyclohexylethynyl)cyclohexyl)amino)acrylate (1r).

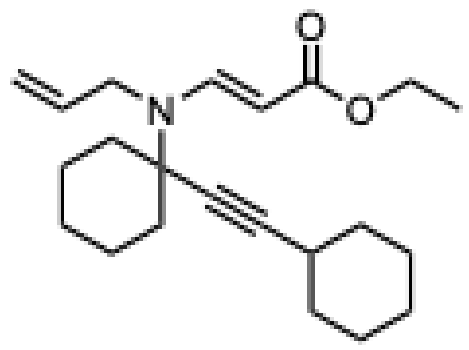

1r
Chemical Formula: $C_{22}H_{33}NO_2$
Molecular Weight: 343,51

Following **general procedure B** starting from **Ir** (128 mg, 0.50 mmol), the title compound **1r** was obtained in quantitative yield as a pale-brown oil. **Rf** = 0.50 (SiO_2, cyclohexane/EtOAc 8/2). **ATR FTIR** ν = 2928, 2854, 1729, 1688, 1598, 1447, 1238, 1141, 966, 790 cm^{-1}. **^{1}H NMR** (500 MHz, $CDCl_3$) δ 8.07 (d, *J* = 13.0 Hz, 1H), 5.74 (ddt, *J* = 17.2, 10.2, 5.0 Hz, 1H), 5.16-5.13 (m, 2H), 4.60 (d, *J* = 13.0 Hz, 1H), 4.11 (q, *J* = 7.1 Hz, 2H), 3.91 (dt, *J* = 5.0, 1.9 Hz, 2H), 2.45 (dt, *J* = 8.9, 4.8 Hz, 1H), 1.92-1.87 (m, 2H), 1.82-1.75 (m, 2H), 1.74-1.62 (m, 8H), 1.62-1.54 (m, 2H), 1.51-1.43 (m, 3H), 1.35-1.31 (m, 2H), 1.24 (t, J = 7.1 Hz, 3H), 1.15-1.04 (m, 1H) ppm. **^{13}C NMR** (126 MHz, $CDCl_3$) δ 170.1, 147.6, 133.4, 116.6, 99.0, 86.0, 79.8, 62.3, 59.0, 48.6, 37.6, 32.8, 29.1, 26.0, 25.2, 24.8, 23.9, 14.8 ppm. **HRMS:** 344.2603 $[M+H]^+$, calcd 344.2584.

Ethyl (*E*)-3-(butyl(1-(phenylethynyl)cyclohexyl)amino)acrylate (1s).

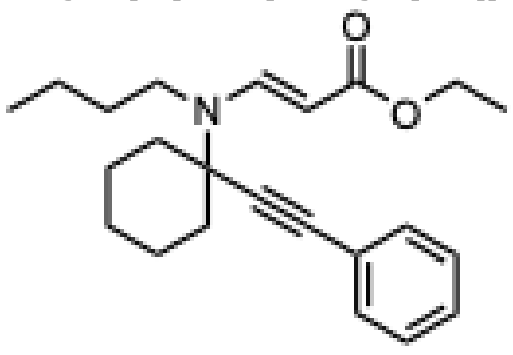

1s
Chemical Formula: $C_{23}H_{31}NO_2$
Molecular Weight: 353,51

Following **general procedure B** starting from I**s** (303.5 mg, 1 mmol), the title compound **1s** was obtained in quantitative yield as an orange oil. **Rf** = 0.45 (SiO_2, cyclohexane/EtOAc 8/2). **ATR FTIR** ν = 3057, 2933, 2860, 1686, 1591, 1330, 1236, 1123, 1075, 755, 691 cm^{-1}. **^{1}H NMR** (500 MHz, $CDCl_3$) δ 8.02 (d, *J* = 13.0 Hz, 1H), 7.46-7.41 (m, 2H), 7.34-7.30 (m, 3H), 4.65 (d, *J* = 13.0 Hz, 1H), 4.15 (q, *J* = 7.1 Hz, 2H), 3.33-3.27 (m, 2H), 2.11-2.02 (m, 2H), 1.83-1.75 (m, 4H), 1.75-1.60 (m, 5H), 1.38-1.30 (m, 2H), 1.27 (t, *J* = 7.1 Hz, 3H), 1.23-1.14 (m, 1H), 0.94 (t, *J* = 7.4 Hz, 3H) ppm. **^{13}C NMR** (126 MHz, $CDCl_3$) δ 170.2, 147.2, 131.7, 128.6, 128.5, 122.8, 89.3, 87.8, 85.3, 62.3, 59.0, 46.8, 37.6, 29.8, 25.2, 23.9, 20.6, 14.9, 14.0 ppm. **HRMS:** 354.2439 $[M+H]^+$, calcd 354.2428.

1.5 Synthesis of 1,2-dihydropyridines (**2**)

General procedure C: synthesis of 1,2-dihydropyridines 2 from 1

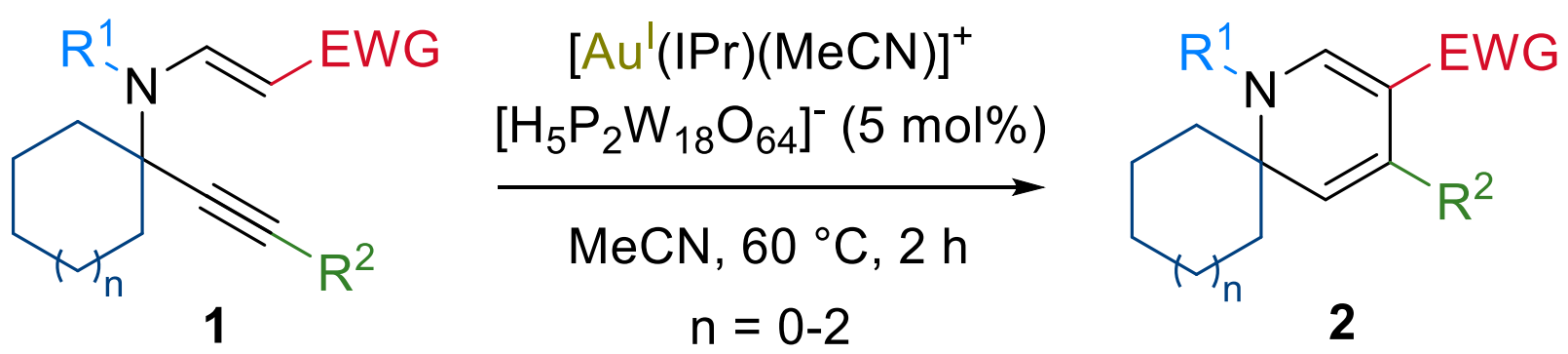

$[Au^I(IPr)(MeCN)][H_5P_2W_{18}O_{62}]$ (5 mol %) was added to a solution of enyne (**1**) in MeCN (1 M) in a screw cap tube (3 mL) equipped with a stirring bar. The reaction flask was flushed with argon before sealing. The mixture was placed into a pre-heated aluminum block at 60 °C and stirred until completion. The catalyst was filtered off through a short pad of Celite and the residue was washed with Et_2O (3 × 20 mL). The filtrate was concentrated under reduced pressure and the crude product was purified by column chromatography over silica gel (cyclohexane/EtOAc) to afford the product (**2**).

Recycling procedure of gold(I)-polyoxometallate complex catalysts

After completion, the reaction mixture is diluted in Et_2O (2 mL) and after centrifugation and decantation, the supernatant was taken out. This operation was repeated 3 times. The decanted catalyst was dried under reduced pressure and directly engaged for the next cycle. This procedure was repeated up to 3 consecutive times.

Ethyl 1-benzyl-4-cyclohexyl-1-azaspiro[5.5]undeca-2,4-diene-3-carboxylate (2a).

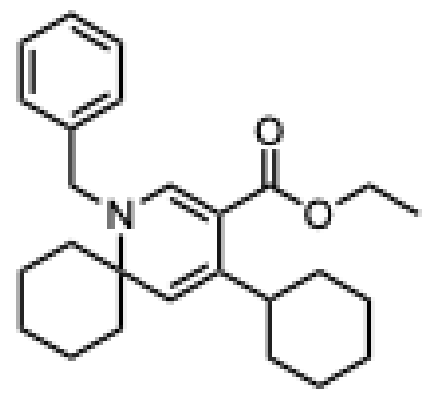

2a
Chemical Formula: $C_{26}H_{35}NO_2$
Molecular Weight: 393,57

Following **General Procedure C** starting from **1a** (50 mg, 0.13 mmol), the title compound **2a** was obtained (42.1 mg, 1.07 mmol, 82% yield) as a pale-yellow oil after purification by column chromatography of the crude residue (cyclohexane/EtOAc 95/5). **Rf** = 0.60 (SiO_2, cyclohexane/EtOAc 8/2). **ATR FTIR** ν = 2925, 2851, 1674, 1553, 1450, 1287, 1218, 1134, 1071, 907, 750 cm^{-1}. **^{1}H NMR** (500 MHz, $CDCl_3$) δ 7.50 (s, 1H), 7.34-7.29 (m, 2H), 7.25-7.19 (m, 3H) 5.00 (d, *J* = 1.2 Hz, 1H), 4.49 (s, 2H), 4.13 (q, *J* = 7.1 Hz, 2H), 3.04 (m, 1H), 1.91-1.04 (m, 19H), 1.26 (t, *J* = 7.1 Hz, 3H), 0.92-0.78 (m, 1H) ppm. **^{13}C NMR** (126 MHz, $CDCl_3$) δ 167.0, 150.5, 140.1, 139.7, 128.8, 127.4, 126.6, 110.8, 99.5, 59,9, 59.0, 53.4, 38.9, 34.7, 33.8, 27.3, 26.9, 25.7, 21.7, 14.7 ppm. **HRMS:** 393.2657 $[M]^+$, calcd 393.2662.

2a was also obtained at gram-scale (665 mg, 1.7 mmol, 67%) from **1a** (1 g, 2.54 mmol)

2a can be obtained via one-pot process from **Ia**. Activated alkyl propiolate (1.05 equiv) was added to a solution of propargylamine **Ia** (1.0 equiv) in EtOH (1 M) in a screw cap tube (3 mL) equipped with a stirring bar. The reaction flask was flushed with argon before sealing. Next, the mixture was placed into a pre-heated aluminium block at 100 °C and stirred until completion. The temperature is reduced to 60 °C and [Au^I(IPr)(MeCN)][$H_5P_2W_{18}O_{62}$] (5 mol %) was then added to the reaction mixture. The reaction flask was flushed with argon before sealing and stirred until completion. The catalyst was filtered off through a short pad of Celite and the residue was washed with Et_2O (3 × 20 mL). The filtrate was concentrated under reduced pressure and the crude product was purified by column chromatography over silica gel (cyclohexane/EtOAc 8/2) to afford the product **2a** (28.5 mg, 74.1 µmol, 57% yield).

Following the **Recycling procedure**, **2a** (50 mg, 0.13 mmol) with 5 mol % of catalyst [Au^I(IPr)(MeCN)][$H_5P_2W_{18}O_{62}$] (25.8 mg) with was obtained with 66% yield (33 mg) after RUN 1 (2 h), 63% yield (31.5 mg) after RUN 2 (2 h), 42% yield (21 mg) after RUN 3 (5 h) and 10% yield (5 mg) after RUN 4 (30 h). [Au^I(IPr)(MeCN)][$H_5P_2W_{18}O_{62}$] was recovered up to 95% (RUN 1, m_{cat} = 22.4mg), 82% (RUN 2, m_{cat} = 21.0 mg) and 77% (RUN 3, m_{cat} = 18.3 mg). It was not recovered after RUN 4 since it was not effective anymore.

Ethyl 1-benzyl-4-cyclopropyl-1-azaspiro[5.5]undeca-2,4-diene-3-carboxylate (2b).

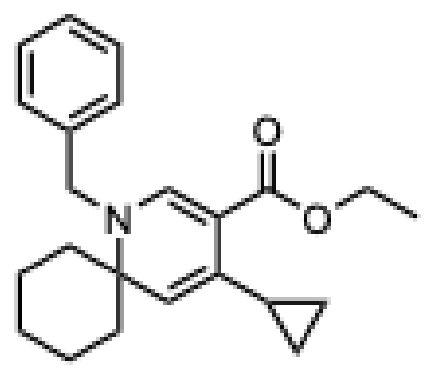

2b
Chemical Formula: $C_{23}H_{29}NO_2$
Molecular Weight: 351,49

Following **General Procedure C** starting from **1b** (45.7 mg, 0.13 mmol), the title compound **2b** was obtained (32.4 mg, 92.3 µmol, 71% yield) as a pale-yellow oil after purification by column chromatography of the crude residue (cyclohexane/EtOAc 95/5). **Rf** = 0.52 (SiO_2, cyclohexane/EtOAc 8/2). **ATR FTIR** ν = 2928, 1677, 1626, 1554, 1291, 1207, 1130, 1070, 654 cm^{-1}. **^{1}H NMR** (500 MHz, $CDCl_3$) δ 7.50 (s, 1H), 7.36-7.32 (m, 2H), 7.29-7.21 (m, 3H), 4.90 (m, 1H), 4.50 (s, 2H), 4.18 (q, J = 7.1 Hz, 2H), 2.16-2.10 (m, 1H), 1.86-1.80 (m, 2H), 1.65-1.59 (m, 1H), 1.52-1.38 (m, 6H), 1.28 (t, *J* = 7.1 Hz, 3H), 1.10-1.05 (m, 1H), 0.77-0.72 (m, 2H), 0.51-0.47 (td, *J* = 6.0, 4.0, 2H) ppm. **^{13}C NMR** (126 MHz, $CDCl_3$) δ 167.1, 149.8, 139.4, 135.9, 128.8, 127.4, 126.6, 110.2, 100.2, 60.0, 59.0, 53.3, 34.8, 25.5, 21.5, 14.7, 14.3, 7.0 ppm. **HRMS:** 374.2085 [M+Na]$^+$, calcd 374,2091.

Ethyl 1-benzyl-4-hexyl-1-azaspiro[5.5]undeca-2,4-diene-3-carboxylate (2c) and Ethyl (*Z*)-1-benzyl-4-hexylidene-1-azaspiro[5.5]undec-2-ene-3-carboxylate (2c').

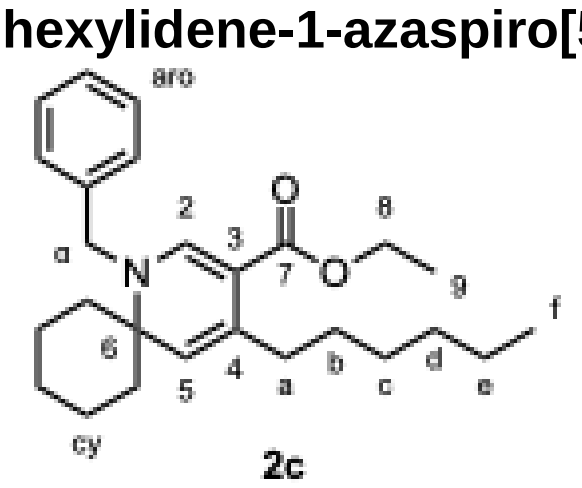

2c
Chemical Formula: $C_{26}H_{37}NO_2$
Molecular Weight: 395,59

+

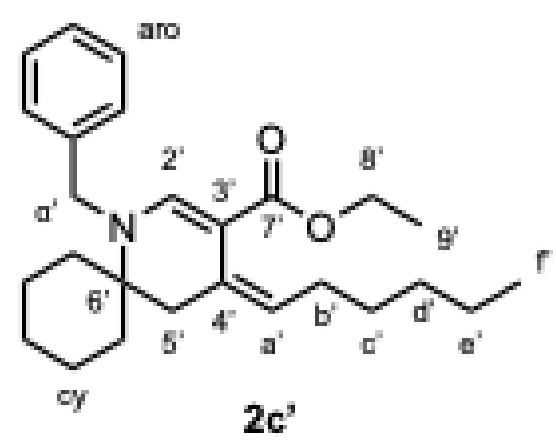

2c'
Chemical Formula: $C_{26}H_{37}NO_2$
Molecular Weight: 395,59

Following **General Procedure C** starting from **1c** (50.0 mg, 0.13 mmol), the title compound **2c** was obtained (12 mg, 30.3 µmol, 23% yield, **2c:2c'** 40:60) as a pale-yellow oil after purification by column chromatography of the crude residue (cyclohexane/EtOAc 95/5). **Rf** = 0.60 (SiO_2, cyclohexane/EtOAc 8/2). **ATR FTIR** ν = 2925, 2854, 1679, 1581, 1452, 1363, 1162, 1069, 730 cm^{-1}. **^{1}H NMR** (500 MHz, $CDCl_3$) δ 7.49 (s, 1H, H_2), 7.42 (s, 1H, $H_{2'}$), 7.37-7.29 (m, 3H x2, H_{aro}), 7.28-7.18 (m, 2H x2, H_{aro}), 6.32 (t, *J* = 7.3 Hz, 1H, $H_{a'}$), 4.98 (s, 1H, H_5), 4.49 (s, 2H, H_α), 4.46 (s, 2H, $H_{\alpha'}$), 4.14 (q, *J* = 7.1 Hz, 2H x2, H_7 and $H_{7'}$), 2.55 (s, 2H, $H_{5'}$), 2.50 (t, *J* = 7.5 Hz, 2H, H_a), 2.17 (q, J = 7.3 Hz, 2H, $H_{b'}$), 1.87-1.78 (m, 2H, H_{cy}), 1.71-1.29 (m, 15H x2, H_{cy} and H^n_{hex}), 1.26 (t, J = 7.1 Hz, 3H x2, H_8 and $H_{8'}$), 1.16-1.08 (m, 1H x2, H_{cy}), 0.90-0.86 (m, 3H x2, H_f and $H_{f'}$) ppm. **^{13}C NMR** (126 MHz, $CDCl_3$) δ 167.55, 166.99, 150.43, 147.80, 139.84, 139.66, 134.67, 128.79, 127.41, 127.37, 126.64, 126.56, 125.00, 123.96, 115.07, 113.75, 99.30, 98.36, 60.21, 59.10, 59.00, 58.96, 53.36, 53.24, 34.92, 34.41, 32.95, 32.25, 32.08, 31.92, 30.24, 29.89, 29.28, 28.11, 25.70, 25.64, 22.95, 22.84, 22.74, 21.66, 14.75, 14.72, 14.30, 14.25 ppm. **HRMS:** 396.2894 [M+H]$^+$, calcd 396.2897.

Ethyl 1-benzyl-4-(3-phenylpropyl)-1-azaspiro[5.5]undeca-2,4-diene-3-carboxylate (2d) and Ethyl (*Z*)-1-benzyl-4-(3-phenylpropylidene)-1-azaspiro[5.5]undec-2-ene-3-carboxylate (2d').

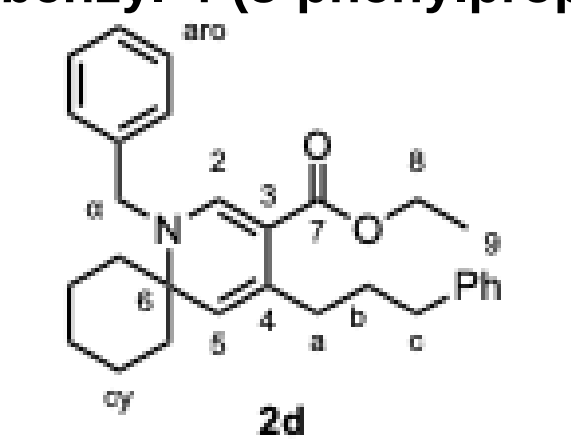

2d
Chemical Formula: $C_{29}H_{35}NO_2$
Molecular Weight: 429,60

+

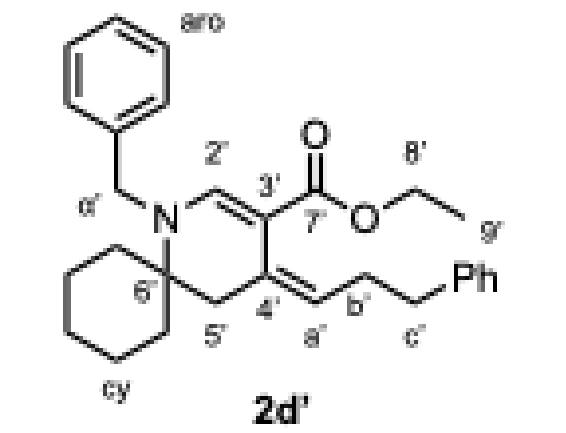

2d'
Chemical Formula: $C_{29}H_{35}NO_2$
Molecular Weight: 429,60

Following **General Procedure C** starting from **1d** (71.2 mg, 0.17 mmol), the title compound **2d** was obtained (16.8 mg, 39.1 μmol, 16% yield, **2d:2d'** 43:57) as a pale-yellow oil after purification by column chromatography of the crude residue (cyclohexane/EtOAc 95/5). **Rf** = 0.54 (SiO_2, cyclohexane/EtOAc 8/2). **ATR FTIR** ν = 3026, 2928, 2856, 1679, 1581, 1557, 1452, 1288, 1222, 1131, 1067, 1028, 729, 697 cm^{-1}. ^{1}**H NMR** (500 MHz, $CDCl_3$) δ 7.51 (s, 1H, H_2), 7.44 (s, 1H, $H_{2'}$), 7.36-7.26 (m, 5H x 2, H_{aro}), 7.25-7.15 (m, 5H x 2, H_{aro}), 6.40 (t, *J* = 7.2 Hz, 1H, $H_{a'}$), 4.98 (s, 1H), 4.50 (s, 2H), 4.45 (s, 2H), 4.14 (q, *J* = 7.1 Hz, 2H x 2, H_7 and $H_{7'}$), 2.77-2.73 (m, 2H, $H_{c'}$), 2.69-2.64 (m, 2H, H_c), 2.62-2.57 (m, 2H, H_a), 2.52-2.46 (m, 2H, $H_{b'}$), 2.51 (s, 2H, $H_{5'}$), 1.86-1.82 (m, 2H, H_{cy}), 1.79 (td, *J* = 7.3, 7.0, 3.4 Hz, 2H, H_b), 1.65-1.61 (m, 2H, H_{cy}), 1.58-1.30 (m, 7H x 2, H_{cy}), 1.06 (dt, *J* = 12.9, 3.8 Hz, 1H x 2, H_{cy}) ppm. ^{13}**C NMR** (126 MHz, $CDCl_3$) δ 167.5, 166.9, 150.6, 148.0, 143.3, 142.8, 139.7, 139.6, 134.2, 128.8, 128.8, 128.7, 128.7, 128.4, 128.3, 127.5, 127.4, 126.6, 126.5, 125.9, 125.8, 125.6, 122.6, 114.1, 99.1, 98.2, 60.3, 59.2, 59.0, 59.0, 53.4, 53.3, 37.0, 35.9, 34.9, 34.1, 32.9, 32.2, 31.8, 30.7, 25.6, 25.6, 22.7, 21.6, 14.8, 14.8 ppm. **HRMS:** 430.2731 $[M+H]^+$, calcd 430.274.

Ethyl 1-benzyl-4-(2-hydroxypropan-2-yl)-1-azaspiro[5.5]undeca-2,4-diene-3-carboxylate (2e).

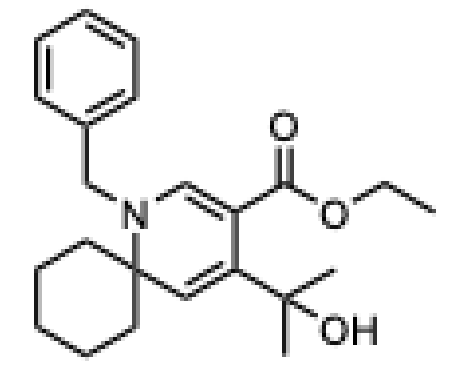

2e
Chemical Formula: $C_{23}H_{31}NO_3$
Molecular Weight: 369,50

Following **General Procedure C** starting from **1e** (36.3 mg, 98.2 μmol), the title compound **2e** was obtained (4.5 mg, 12.2 μmol, 12% yield) as a pale-yellow oil after purification by column chromatography of the crude residue (cyclohexane/EtOAc 95/5). **Rf** = 0.17 (SiO_2, cyclohexane/EtOAc 8/2). **ATR FTIR** ν = 3312, 2977, 2929, 2856, 1646, 1628, 1556, 1411, 1347, 1287, 1223, 1167, 1135, 1074, 1054, 715 cm^{-1}. ^{1}**H NMR** (500 MHz, $CDCl_3$) δ 7.67 (s, 1H), 7.38-7.27 (m, 3H), 7.24-7.17 (m, 2H), 7.11 (s, 1H), 5.27 (s, 1H), 4.52 (s, 2H), 4.14 (q, *J* = 7.1 Hz, 2H), 1.92-1.82 (m, 2H), 1.70-1.47 (m, 8H), 1.45 (s, 6H), 1.24 (t, *J* = 7.1 Hz, 3H) ppm. ^{13}**C NMR** (126 MHz, $CDCl_3$) δ 168.5, 152.8, 139.4, 138.7, 128.8, 127.6, 126.5, 112.2, 97.6, 70.4, 59.9, 59.8, 53.4, 34.6, 29.5, 25.4, 21.6, 14.5 ppm. **HRMS:** 370.2370 $[M+H]^+$, calcd 370.2377.

Ethyl 1-benzyl-4-phenyl-1-azaspiro[5.5]undeca-2,4-diene-3-carboxylate (2f).

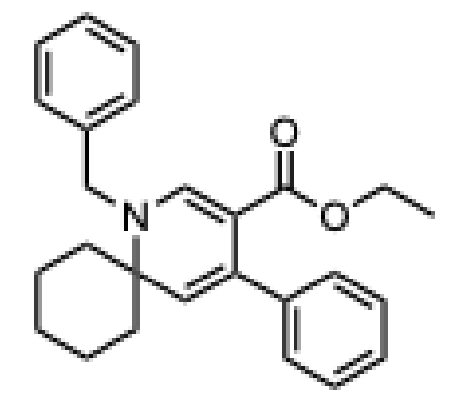

2f
Chemical Formula: $C_{26}H_{29}NO_2$
Molecular Weight: 387,52

Following **General Procedure C** starting from **1f** (50.4 mg, 0.13 mmol), the title compound **2f** was obtained (38.4 mg, 99.1 μmol, 76% yield) as a pale-yellow oil after purification by column chromatography of the crude residue (cyclohexane/EtOAc 95/5). **Rf** = 0.39 (SiO_2, cyclohexane/EtOAc 8/2). **ATR FTIR** ν = 2929, 1676, 1615, 1550, 1451, 1291, 1207, 1138, 1070, 907, 654 cm^{-1}. ^{1}**H NMR** (500 MHz, $CDCl_3$) δ 7.63 (s, 1H), 7.35-7.23 (m, 10H), 5.10 (s, 1H), 4.57 (s, 2H), 3.96 (q, *J* = 7.1 Hz, 2H), 2.00-1.96 (m, 2H), 1.68-1.63 (m, 1H), 1.57-1.52 (m, 4H), 1.49-1.43 (m, 2H), 1.15-1.05 (m, 1H), 0.95 (t, *J* = 7.1 Hz, 3H) ppm. ^{13}**C NMR** (126 MHz, $CDCl_3$) δ 166.7, 150.3, 142.0, 139.3, 136.1, 128.9, 127.9, 127.6, 127.5, 126.7, 126.6, 116.5, 100.1, 60.8, 59.1, 53.6, 34.4, 25.6, 21.8, 14.2 ppm. **HRMS:** 388.2295 $[M+H]^+$, calcd 388.2271.

Ethyl 1-benzyl-4-(4-(*tert*-butyl)phenyl)-1-azaspiro[5.5]undeca-2,4-diene-3-carboxylate (2g).

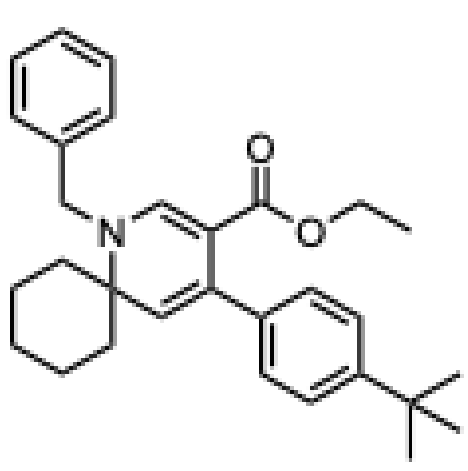

2g
Chemical Formula: $C_{30}H_{37}NO_2$
Molecular Weight: 443,63

Following **General Procedure C** starting from **1g** (62.5 mg, 0.14 mmol), the title compound **2g** was obtained (50.9 mg, 0.11 mmol, 82% yield) as a pale-yellow solid after purification by column chromatography of the crude residue (cyclohexane/EtOAc 95/5). **Rf** = 0.43 (SiO_2, cyclohexane/EtOAc 8/2). **ATR FTIR** ν = 2933, 2852, 1676, 1617, 1551, 1293, 1221, 1133, 1066, 919, 729 cm^{-1}. ^{1}**H NMR** (500 MHz, $CDCl_3$) δ 7.63 (s, 1H), 7.34-7.30 (m, 4H), 7.27-7.24 (m, 3H), 7.22-7.1 (m, 2H), 5.10 (s, 1H), 4.57 (s, 2H), 3.96 (q, *J* = 7.1 Hz, 2H), 1.99-1.93 (m, 2H), 1.68-1.62 (m, 1H), 1.59-1.50 (m, 4H), 1.45 (td, *J* = 12.2, 4.9 Hz, 2H), 1.33 (s, 9H), 1.15-1.04 (m, 1H), 0.92 (t, *J* = 7.1 Hz, 3H) ppm. ^{13}**C NMR** (126 MHz, $CDCl_3$) δ 166.7, 150.2, 149.4, 139.2, 138.8, 135.7, 128.7, 127.4, 127.4, 126.5, 124.3, 116.1, 100.0, 60.7, 59.9, 34.5, 34.2, 31.5, 25.5, 21.7, 13.9 ppm. **HRMS:** 444.2886 $[M+H]^+$, calcd 444.2897.

Ethyl 1-benzyl-4-(4-methoxyphenyl)-1-azaspiro[5.5]undeca-2,4-diene-3-carboxylate (2h).

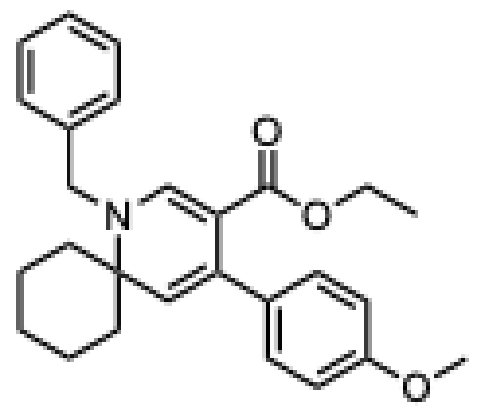

2h
Chemical Formula: $C_{27}H_{31}NO_3$
Molecular Weight: 417,55

Following **General Procedure C** starting from **1h** (50.0 mg, 0.12 mmol), the title compound **2h** was obtained (32.6 mg, 78.0 μmol, 65% yield) as a pale-yellow solid after purification by column chromatography of the crude residue (cyclohexane/EtOAc 95/5). **Rf** = 0.32 (SiO_2, cyclohexane/EtOAc 8/2). **ATR FTIR** ***ν*** = 2930, 2855, 1676, 1610, 1551, 1502, 1290, 1206, 1173, 1029, 728 cm^{-1}. **^{1}H NMR** (500 MHz, $CDCl_3$) δ 7.62 (s, 1H), 7.35-7.31 (m, 2H), 7.28-7.24 (m, 3H), 7.22-7.18 (m, 2H), 6.86-6.83 (m, 2H), 5.05 (s, 1H), 4.56 (s, 2H), 3.99 (q, *J* = 7.1 Hz, 2H), 3.82 (s, 3H), 1.99-1.94 (m, 2H), 1.68-1.62 (m, 1H), 1.56-1.51 (m, 4H), 1.49-1.41 (m, 2H), 1.15-1.06 (m, 1H), 1.02 (t, *J* = 7.1 Hz, 3H) ppm. **^{13}C NMR** (126 MHz, $CDCl_3$) δ 166.7, 156.7, 150.3, 139.2, 135.5, 134.4, 129.0, 128.9, 127.6, 126.7, 115.8, 113.0, 100.0, 60.8, 59.1, 55.4, 53.9, 34.4, 25.6, 21.8, 14.4 ppm. **HRMS:** 418.2365 $[M+H]^+$, calcd 418.2377.

Ethyl 1-benzyl-4-(3,5-bis(trifluoromethyl)phenyl)-1-azaspiro[5.5]undeca-2,4-diene-3-carboxylate (2i).

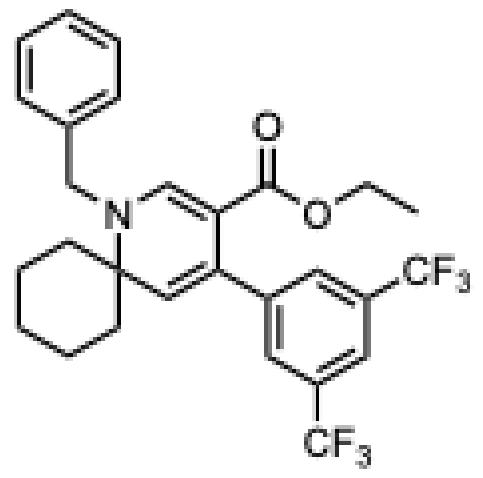

2i
Chemical Formula: $C_{28}H_{27}F_6NO_2$
Molecular Weight: 523,52

Following **General Procedure C** starting from **1i** (67.3 mg, 0.13 mmol) heated at 80 °C, the title compound **2i** was obtained (33.4 mg, 63.8 μmol, 50% yield) as a pale-yellow oil after purification by column chromatography of the crude residue (cyclohexane/EtOAc 95/5). **Rf** = 0.46 (SiO_2, cyclohexane/EtOAc 8/2). **ATR FTIR** ***ν*** = 2980, 2934, 2859, 1679, 1612, 1553, 1364, 1275, 1209, 1171, 1128, 907, 724 cm^{-1}. **^{1}H NMR** (500 MHz, $CDCl_3$) δ 7.81-7.76 (bs, 1H), 7.71 (s, 1H), 7.70-7.68 (bs, 2H), 7.42-7.30 (m, 4H), 5.11 (s, 1H), 4.61 (s, 2H), 3.96 (q, *J* = 7.1 Hz, 2H), 2.08-1.95 (m, 2H), 1.78-1.67 (m, 1H), 1.66-1.50 (m, 6H), 1.20-1.07 (m, 1H), 0.94 (t, *J* = 7.1 Hz, 3H) ppm. **^{13}C NMR** (126 MHz, $CDCl_3$) δ 166.1, 151.1, 144.2, 138.7, 134.3, 130.7 (q, *J* = 33.1 Hz), 129.0, 128.2, 127.8, 126.8, 124.8, 122.6, 120.4 (m), 117.8, 98.9, 61.2, 59.3, 53.8, 34.4, 25.4, 21.7, 14.0 ppm. **^{19}F NMR** (282 MHz, $CDCl_3$) δ -62.6 ppm. **HRMS:** 524.2005 $[M+H]^+$, calcd 524.2019.

Ethyl 1-benzyl-4-(thiophen-3-yl)-1-azaspiro[5.5]undeca-2,4-diene-3-carboxylate (2j).

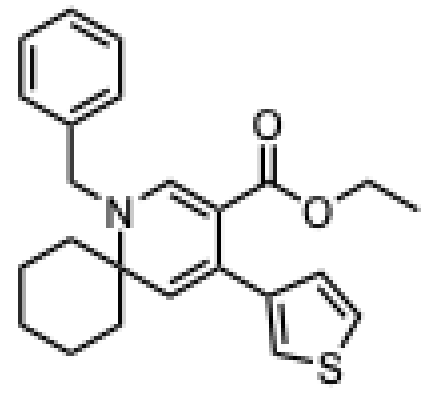

2j
Chemical Formula: $C_{24}H_{27}NO_2S$
Molecular Weight: 393,55

Following **General Procedure C** starting from **1j** (51.2 mg, 0.13 mmol), the title compound **2j** was obtained (39.4 mg, 0.10 mmol, 77% yield) as a pale-yellow oil after purification by column chromatography of the crude residue (cyclohexane/EtOAc 95/5). **Rf** = 0.43 (SiO_2, cyclohexane/EtOAc 8/2). **ATR FTIR** ***ν*** = 2927, 2854, 1675, 1612, 1551, 1495, 1451, 1361, 1289, 1210, 1136, 1070, 732, 697 cm^{-1}. **^{1}H NMR** (500 MHz, $CDCl_3$) δ 7.61 (s, 1H), 7.35-7.32 (m, 2H), 7.28-7.24 (m, 3H), 7.21 (dd, *J* = 4.9, 3.0 Hz, 1H), 7.15 (dd, *J* = 3.0, 1.3 Hz, 1H), 7.01 (dd, *J* = 5.0, 1.3 Hz, 1H), 5.20 (s, 1H), 4.56 (s, 2H), 4.02 (q, *J* = 7.1 Hz, 2H), 1.98-1.95 (m, 2H), 1.69-1.62 (m, 2H), 1.58-1.52 (m, 6H), 1.06 (t, *J* = 7.1 Hz, 3H) ppm. **^{13}C NMR** (126 MHz, $CDCl_3$) δ 166.6, 150.2, 142.5, 139.2, 130.8, 128.9, 128.7, 127.6, 126.7, 123.7, 121.0, 116.0, 99.9, 60.6, 59.2, 53.7, 34.3, 25.8, 21.8, 14.3 ppm. **HRMS:** 394.1824 $[M+H]^+$, calcd 394.1835.

Ethyl 1-benzyl-4-phenyl-1-azaspiro[4.5]dodeca-2,4-diene-3-carboxylate (2k).

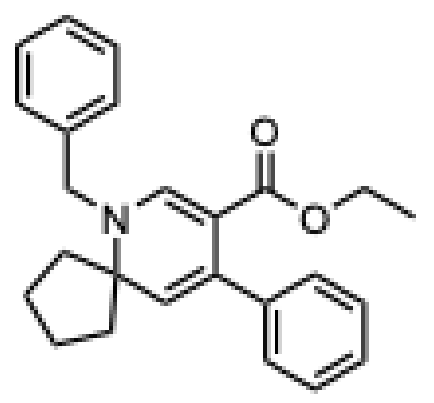

2k
Chemical Formula: $C_{25}H_{27}NO_2$
Molecular Weight: 373,50

Following **General Procedure C** starting from **1k** (44.8 mg, 0.12 mmol), the title compound **2k** was obtained (19.3 mg, 51.6 μmol, 43% yield) as a pale-yellow oil after purification by column chromatography of the crude residue (cyclohexane/EtOAc 95/5). **Rf** = 0.42 (SiO_2, cyclohexane/EtOAc 8/2). **ATR FTIR** ***ν*** = 2955, 2901, 2872, 1679, 1613, 1549, 1442, 1288, 1219, 1145, 1084, 1032, 754, 730, 697 cm^{-1}. **^{1}H NMR** (500 MHz, $CDCl_3$) δ 7.56 (s, 1H), 7.39-7.33 (m, 2H), 7.32-7.24 (m, 8H), 4.90 (s, 1H), 4.52 (s, 2H), 3.93 (q, *J* = 7.1 Hz, 2H), 1.94-1.80 (m, 4H), 1.73-1.63 (m, 4H), 0.93 (t, *J* = 7.1 Hz, 3H) ppm. **^{13}C NMR** (126 MHz, $CDCl_3$) δ 166.6, 150.1, 141.8, 138.9, 134.4, 129.0, 127.8, 127.7, 127.5, 126.6, 126.5, 121.0, 99.4, 68.3, 59.0, 53.9, 39.5, 23.2, 13.2 ppm. **HRMS:** 374.2091 $[M+H]^+$, calcd 374.2115.

Ethyl 1-benzyl-4-cyclohexyl-1-azaspiro[5.6]dodeca-2,4-diene-3-carboxylate (2l).

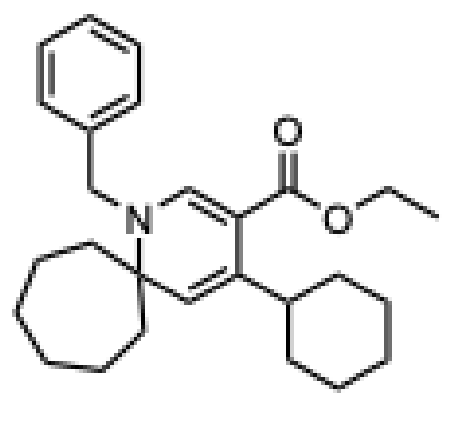

2l
Chemical Formula: $C_{27}H_{37}NO_2$
Molecular Weight: 407,60

Following **General Procedure C** starting from **1l** (52.9 mg, 0.13 mmol), the title compound **2l** was obtained (19.8 mg, 48.6 µmol, 37% yield) as a pale-yellow oil after purification by column chromatography of the crude residue (cyclohexane/EtOAc 95/5). **Rf** = 0.60 (SiO_2, cyclohexane/EtOAc 8/2). **ATR FTIR** *ν* = 2921, 2849, 1677, 1630, 1555, 1450, 1284, 1217, 1140, 1065, 729 cm^{-1}. **^{1}H NMR** (500 MHz, $CDCl_3$) δ 7.40 (s, 1H), 7.35-7.31 (m, 2H), 7.28-7.24 (m, 1H), 7.22-7.19 (m, 2H), 4.84 (s, 1H), 4.51 (s, 2H), 4.10 (q, *J* = 7.1 Hz, 2H), 3.03 (ttd, *J* = 11.6, 3.0, 1.1 Hz, 1H), 1.83 (q, *J* = 5.2, 5.0 Hz, 4H), 1.80-1.74 (m, 2H), 1.74-1.68 (m, 1H), 1.55-1.45 (m, 9H), 1.44-1.34 (m, 3H), 1.28 (t, J = 7.1 Hz, 3H), 1.15-1.06 (m, 3H) ppm. **^{13}C NMR** (126 MHz, $CDCl_3$) δ 166.9, 149.5, 139.4, 139.4, 128.9, 127.5, 126.8, 114.4, 99.2, 62.9, 58.9, 53.5, 39.4, 38.6, 33.8, 29.7, 27.3, 26.9, 22.5, 14.7 ppm. **HRMS:** 408.2887 $[M+H]^+$, calcd 408.2897.

Ethyl 1-benzyl-4-phenyl-1-azaspiro[5.6]dodeca-2,4-diene-3-carboxylate (2m).

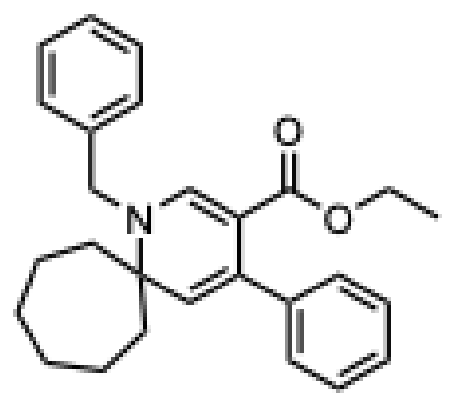

2m
Chemical Formula: $C_{27}H_{31}NO_2$
Molecular Weight: 401,55

Following **General Procedure C** starting from **1m** (53.8 mg, 0.13 mmol), the title compound **2m** was obtained (30.5 mg, 74.1 µmol, 57% yield) as a pale-yellow oil after purification by column chromatography of the crude residue (cyclohexane/EtOAc 95/5). **Rf** = 0.57 (SiO_2, cyclohexane/EtOAc 8/2). **ATR FTIR** *ν* = 2977, 2924, 2853, 1677, 1616, 1552, 1452, 1287, 1212, 1148, 1071, 757, 725, 697 cm^{-1}. **^{1}H NMR** (500 MHz, $CDCl_3$) δ 7.52 (s, 1H), 7.38-7.33 (m, 2H), 7.31-7.24 (m, 8H), 4.97 (s, 1H), 4.59 (s, 2H), 3.94 (q, *J* = 7.1 Hz, 2H), 2.04-1.96 (m, 2H), 1.92-1.85 (m, 2H), 1.62-1.50 (m, 8H), 0.94 (t, *J* = 7.1 Hz, 3H) ppm. **^{13}C NMR** (126 MHz, $CDCl_3$) δ 166.6, 149.5, 142.0, 139.1, 135.6, 129.0, 127.9, 127.7, 127.5, 126.9, 126.7, 120.0, 99.9, 63.8, 59.1, 53.7, 39.0, 29.6, 22.4, 14.2 ppm. **HRMS:** 402.2423 $[M+H]^+$, calcd 402.2428.

1-Benzyl-4-cyclohexyl-3-tosyl-1-azaspiro[5.5]undeca-2,4-diene (2n).

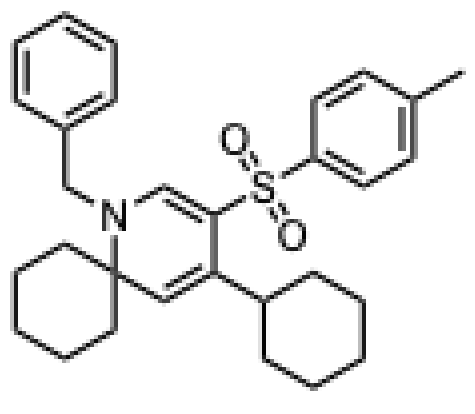

2n
Chemical Formula: $C_{30}H_{37}NO_2S$
Molecular Weight: 475,69

Following **General Procedure C** starting from **1n** (64 mg, 0.14 mmol), the title compound **2n** was obtained (26.6 mg, 55.9 µmol, 42% yield) as a pale-yellow oil after purification by column chromatography of the crude residue (cyclohexane/EtOAc 95/5). **Rf** = 0.38 (SiO_2, cyclohexane/EtOAc 8/2). **ATR FTIR** *ν* = 2922, 2850, 1624, 1554, 1449, 1281, 1129, 1089, 908, 729, 675, 659, 537 cm^{-1}. **^{1}H NMR** (500 MHz, $CDCl_3$) δ 7.72-7.68 (m, 2H), 7.49 (s, 1H), 7.38-7.32 (m, 2H), 7.30-7.20 (m, 5H), 5.02 (s, 1H), 4.52 (s, 2H), 2.48-2.42 (m, 1H), 2.40 (s, 3H), 1.86-1.82 (m, 2H), 1.64-1.53 (m, 6H), 1.52-1.39 (m, 5H), 1.12-0.93 (m, 6H) ppm. **^{13}C NMR** (126 MHz, $CDCl_3$) δ 148.7, 142.3, 142.0, 139.1, 137.5, 129.4, 128.9, 127.7, 126.8, 126.6, 112.4, 107.3, 60.4, 53.3, 37.9, 35.1, 34.4, 27.1, 26.4, 25.5, 21.6, 21.5 ppm. **HRMS:** 498.2454 $[M+Na]^+$, calcd 498.2437.

1-Benzyl-4-phenyl-3-tosyl-1-azaspiro[5.5]undeca-2,4-diene (2o).

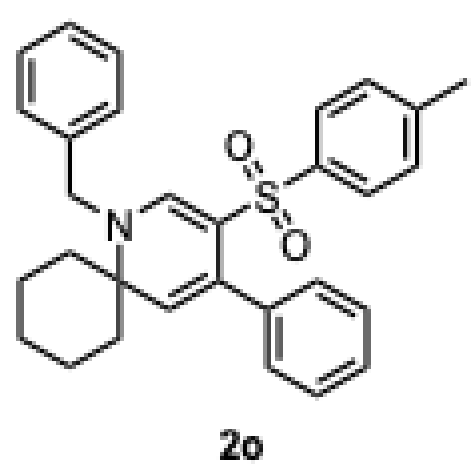

2o
Chemical Formula: $C_{30}H_{31}NO_2S$
Molecular Weight: 469,64

Following **General Procedure C** starting from **1o** (61.0 mg, 0.13 mmol), the title compound **2o** was obtained (38.6 mg, 82.2 µmol, 63% yield) as a white solid after purification by column chromatography of the crude residue (cyclohexane/EtOAc 95/5). **Rf** = 0.31 (SiO_2, cyclohexane/EtOAc 8/2). **ATR FTIR** *ν* = 3059, 3027, 2935, 2858, 1619, 1597, 1547, 1452, 1417, 1291, 1242, 1163, 1130, 1088, 907, 728, 711, 695, 656, 577, 529 cm^{-1}. **^{1}H NMR** (500 MHz, $CDCl_3$) δ 7.58 (s, 1H), 7.41-7.37 (m, 2H), 7.34-7.28 (m, 3H), 7.22-7.17 (m, 1H), 7.15-7.08 (m, 4H), 7.06-6.99 (m, 2H), 6.97-6.93 (m, 2H), 5.02 (s, 1H), 4.59 (s, 2H), 2.34 (s, 3H), 2.00-1.94 (m, 2H), 1.62-1.56 (m, 1H), 1.54-1.44 (m, 6H), 1.12-1.01 (m, 1H) ppm. **^{13}C NMR** (126 MHz, $CDCl_3$) δ 148.0, 141.9, 140.9, 138.9, 138.3, 133.5, 130.0, 129.0, 128.9, 127.8, 127.2, 127.2, 127.1, 126.7, 117.6, 108.5, 61.2, 53.4, 35.1, 27.1, 25.4, 21.6, 21.4 ppm. **HRMS:** 470.2138 $[M+H]^+$, calcd 470.2148.

Ethyl 1-(4-methoxybenzyl)-4-phenyl-1-azaspiro[5.5]undeca-2,4-diene-3-carboxylate (2p).

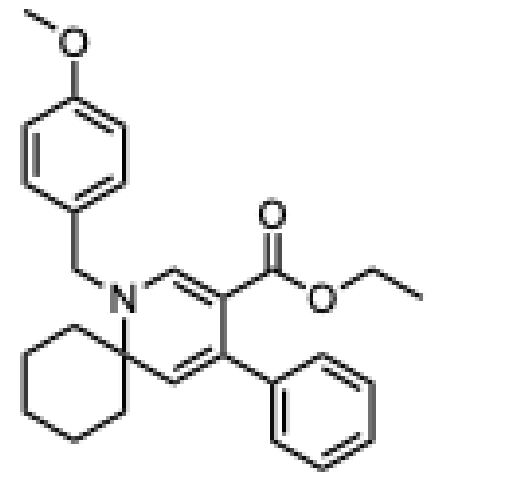

2p
Chemical Formula: $C_{27}H_{31}NO_3$
Molecular Weight: 417,55

Following **General Procedure C** starting from **1p** (62 mg, 0.15 mmol), the title compound **2p** was obtained (46.3 mg, 0.11 mmol, 75% yield) as a pale-yellow oil after purification by column chromatography of the crude residue (cyclohexane/EtOAc 95/5). **Rf** = 0.45 (SiO_2, cyclohexane/EtOAc 8/2). **ATR FTIR** ν = 2931, 2978, 2850, 1662, 1606, 1545, 1511, 1456, 1357, 1287, 1251, 1195, 1129, 1032, 954, 832, 753, 702 cm^{-1}. **^{1}H NMR** (500 MHz, $CDCl_3$) δ 7.63 (s, 1H), 7.30-7.25 (m, 5H), 7.20-7.16 (m, 2H), 6.89-6.85 (m, 2H), 5.08 (s, 1H), 4.50 (s, 2H), 3.95 (q, *J* = 7.1 Hz, 2H), 3.80 (s, 3H), 2.01-1.92 (m, 2H), 1.69-1.62 (m, 1H), 1.59-1.52 (m, 4H), 1.47 (td, *J* = 12.0, 5.2 Hz, 2H), 1.15-1.05 (m, 1H), 0.94 (t, *J* = 7.1 Hz, 3H) ppm. **^{13}C NMR** (126 MHz, $CDCl_3$) δ 166.8, 159.1, 150.2, 142.1, 136.1, 131.0, 128.1, 127.9, 127.5, 126.7, 116.3, 114.3, 99.9, 60.8, 59.0, 55.4, 53.1, 34.3, 25.6, 21.8, 14.2 ppm. **HRMS:** 440.2196 $[M+Na]^+$, calcd 440.2179.

Ethyl 4-cyclohexyl-1-(4-fluorobenzyl)-1-azaspiro[5.5]undeca-2,4-diene-3-carboxylate (2q).

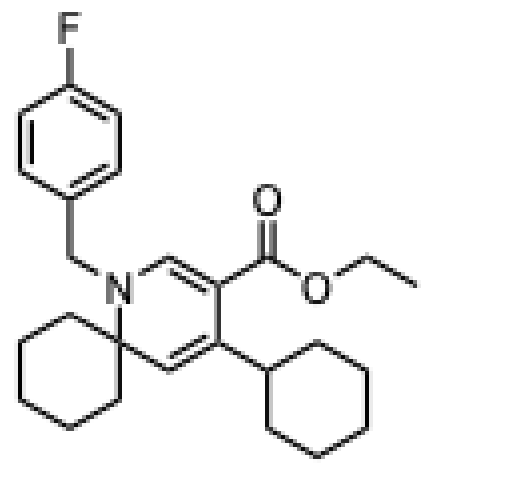

2q
Chemical Formula: $C_{26}H_{34}FNO_2$
Molecular Weight: 411,56

Following **General Procedure C** starting from **1q** (53.8 mg, 0.13 mmol), the title compound **2q** was obtained (38.4 mg, 93.3 μmol, 71% yield) as a pale-yellow oil after purification by column chromatography of the crude residue (cyclohexane/EtOAc 95/5). **Rf** = 0.54 (SiO_2, cyclohexane/EtOAc 8/2). **ATR FTIR** ν = 2925, 2851, 1677, 1554, 1509, 1219, 1134, 1070, 731 cm^{-1}. **^{1}H NMR** (500 MHz, $CDCl_3$) δ 7.47 (s, 1H), 7.16 (m, 2H), 7.05-6.96 (m, 2H), 5.00 (s, 1H), 4.44 (s, 2H), 4.14 (q, *J* = 7.1 Hz, 2H), 3.08-2.96 (m, 1H), 1.92-1.30 (m, 20H), 1.26 (t, *J* = 7.1 Hz, 3H) ppm. **^{13}C NMR** (126 MHz, $CDCl_3$) δ 166.2, 162.2 (d, *J* = 245.5 Hz), 150.2, 140.1, 135.4 (d, *J* = 3.2 Hz), 128.2 (d, *J* = 8.0 Hz), 115.7 (d, *J* = 21.5 Hz), 110.9, 99.9, 59.9, 59.1, 52.7, 38.9, 34.7, 33.8, 27.3, 27.1, 25.7, 21.7, 14.7 ppm. **^{19}F NMR** (282 MHz, $CDCl_3$) δ -115.5 ppm. **HRMS:** 412.2641 $[M+H]^+$**,** calcd 412.2646.

Ethyl 1-allyl-4-cyclohexyl-1-azaspiro[5.5]undeca-2,4-diene-3-carboxylate (2r).

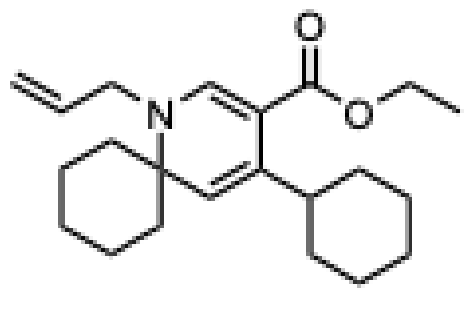

2r
Chemical Formula: $C_{22}H_{33}NO_2$
Molecular Weight: 343,51

Following **General Procedure C** starting from **1r** (44.6 mg, 0.13 mmol), the title compound **2r** was obtained (26.8 mg, 78.0 μmol, 60% yield) as a pale-yellow oil after purification by column chromatography of the crude residue (cyclohexane/EtOAc 95/5). **Rf** = 0.59 (SiO_2, cyclohexane/EtOAc 8/2). **ATR FTIR** ν = 2924, 2851, 1726, 1682, 1555, 1448, 1365, 1284, 1221, 988, 765 cm^{-1}. **^{1}H NMR** (500 MHz, $CDCl_3$) δ 7.41 (s, 1H), 5.84 (ddt, *J* = 17.1, 10.5, 5.4 Hz, 1H), 5.23-5.12 (m, 2H), 4.97 (s, 1H), 4.13 (q, J = 7.1 Hz, 2H), 3.84 (d, J = 5.4 Hz, 2H), 3.04-2.94 (m, 1H), 1.93-1.80 (m, 4H), 1.79-1.59 (m, 6H), 1.56-1.32 (m, 8H), 1.27 (t, J = 7.1 Hz, 3H), 1.14-1.03 (m, 2H) ppm. **^{13}C NMR** (126 MHz, $CDCl_3$) δ 167.0, 149.7, 139.8, 136.0, 117.2, 110.3, 98.8, 59.6, 58.9, 51.8, 38.8, 34.7, 33.8, 27.2, 26.9, 25.7, 21.7, 14.8 ppm. **HRMS:** 344.2576 $[M+H]^+$, calcd 344.2584.

Ethyl 1-butyl-4-phenyl-1-azaspiro[5.5]undeca-2,4-diene-3-carboxylate (2s).

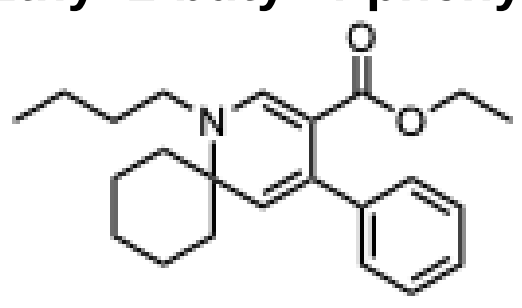

2s
Chemical Formula: $C_{23}H_{31}NO_2$
Molecular Weight: 353,51

Following **General Procedure C** starting from **1s** (45.4 mg, 0.13 mmol), the title compound **2s** was obtained (27.1 mg, 78 μmol, 60% yield) as a pale-yellow oil after purification by column chromatography of the crude residue (cyclohexane/EtOAc 95/5). **Rf** = 0.45 (SiO_2, cyclohexane/EtOAc 8/2). **ATR FTIR** ν = 2928, 2857, 1677, 1614, 1551, 1445, 1362, 1275, 1201, 1146, 1069, 908, 756, 731, 698 cm^{-1}. **^{1}H NMR** (500 MHz, $CDCl_3$) δ 7.55 (s, 1H), 7.29-7.21 (m, 5H), 5.02 (s, 1H), 3.95 (q, *J* = 7.1 Hz, 2H), 3.29-3.25 (m, 2H), 2.06-2.03 (m, 2H), 1.73-1.66 (m, 1H), 1.65-1.56 (m, 6H), 1.53-1.45 (m, 2H), 1.39-1.34 (m, 2H), 1.19-1.12 (m, 1H), 0.96 (t, *J* = 7.1 Hz, 3H), 0.93 (t, *J* = 7.4 Hz, 3H) ppm. **^{13}C NMR** (126 MHz, $CDCl_3$) δ 166.9, 149.8, 142.2, 136.0, 127.9, 127.4, 126.6, 115.7, 98.8, 60.4, 58.9, 49.8, 34.6, 34.4, 25.7, 21.8, 20.2, 14.2, 14.0 ppm. **HRMS:** 354.2431 $[M+H]^+$, calcd 354.2428.

2. ¹H and ¹³C NMR spectra

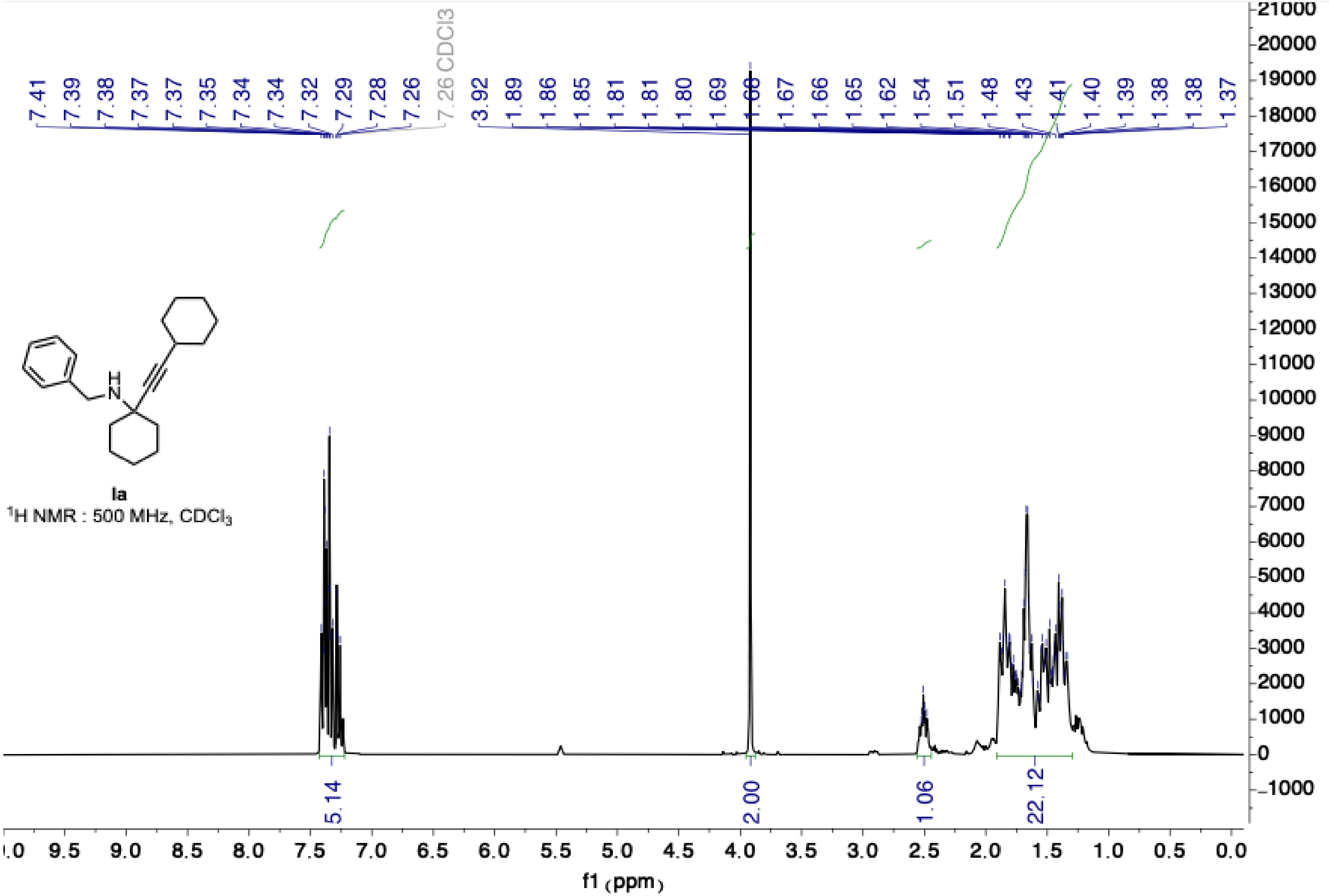

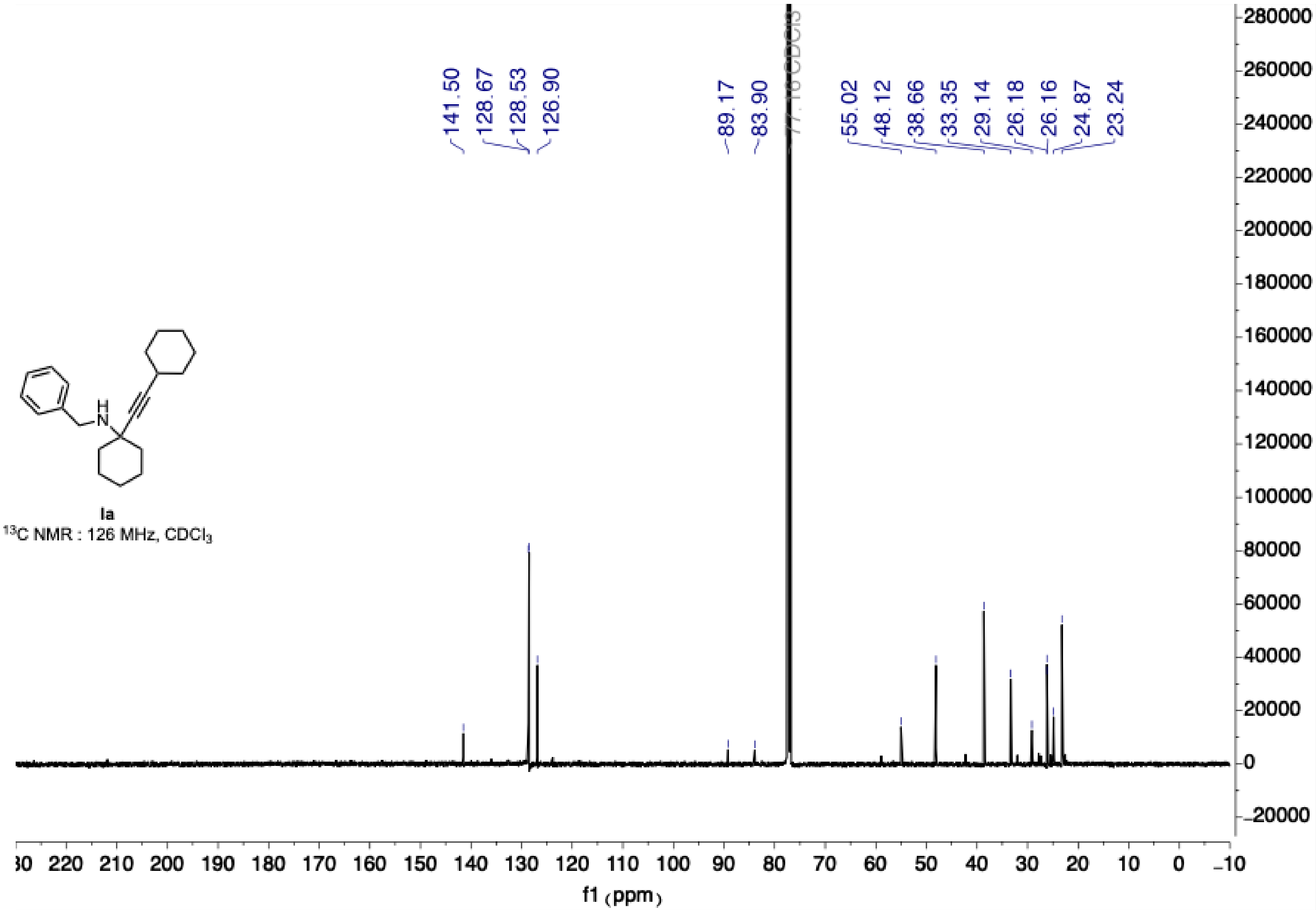
141.50
128.67
128.53
126.90
89.17
83.90
77.16 CDCl3
55.02
48.12
38.66
33.35
29.14
26.18
26.16
24.87
23.24
Ia
^{13}C NMR : 126 MHz, $CDCl_3$
280000
260000
240000
220000
200000
180000
160000
140000
120000
100000
80000
60000
40000
20000
0
-20000
230 220 210 200 190 180 170 160 150 140 130 120 110 100 90 80 70 60 50 40 30 20 10 0 -10
f1 (ppm)

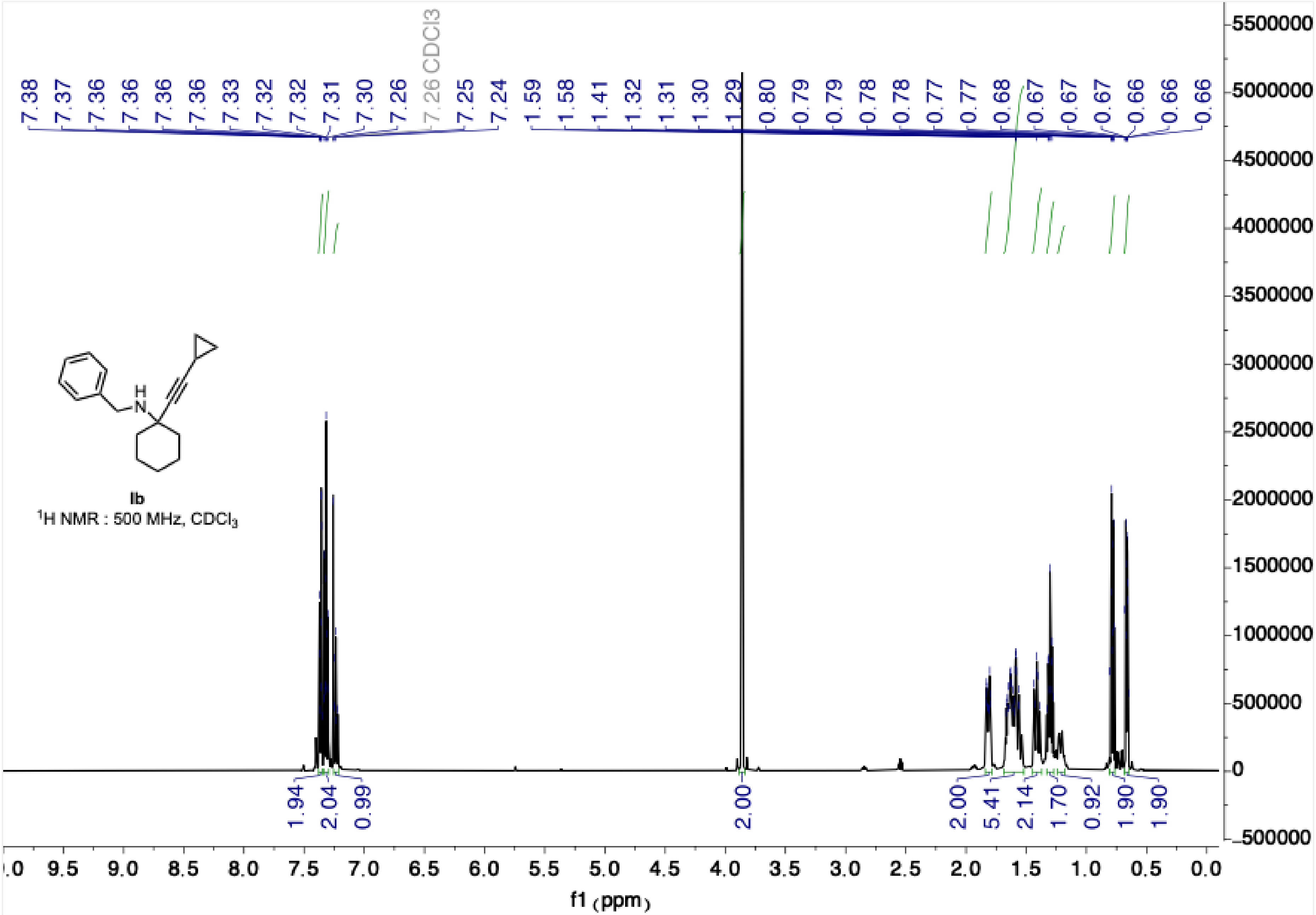

Ib
^{1}H NMR : 500 MHz, CDCl$_3$
7.38 7.37 7.36 7.36 7.36 7.36 7.33 7.32 7.32 7.31 7.30 7.26 7.26 CDCl3 7.25 7.24 1.59 1.58 1.41 1.32 1.31 1.30 1.29 0.80 0.79 0.79 0.78 0.78 0.77 0.77 0.68 0.67 0.67 0.67 0.66 0.66 0.66
1.94 2.04 0.99 2.00 2.00 5.41 2.14 1.70 0.92 1.90 1.90
f1 (ppm)

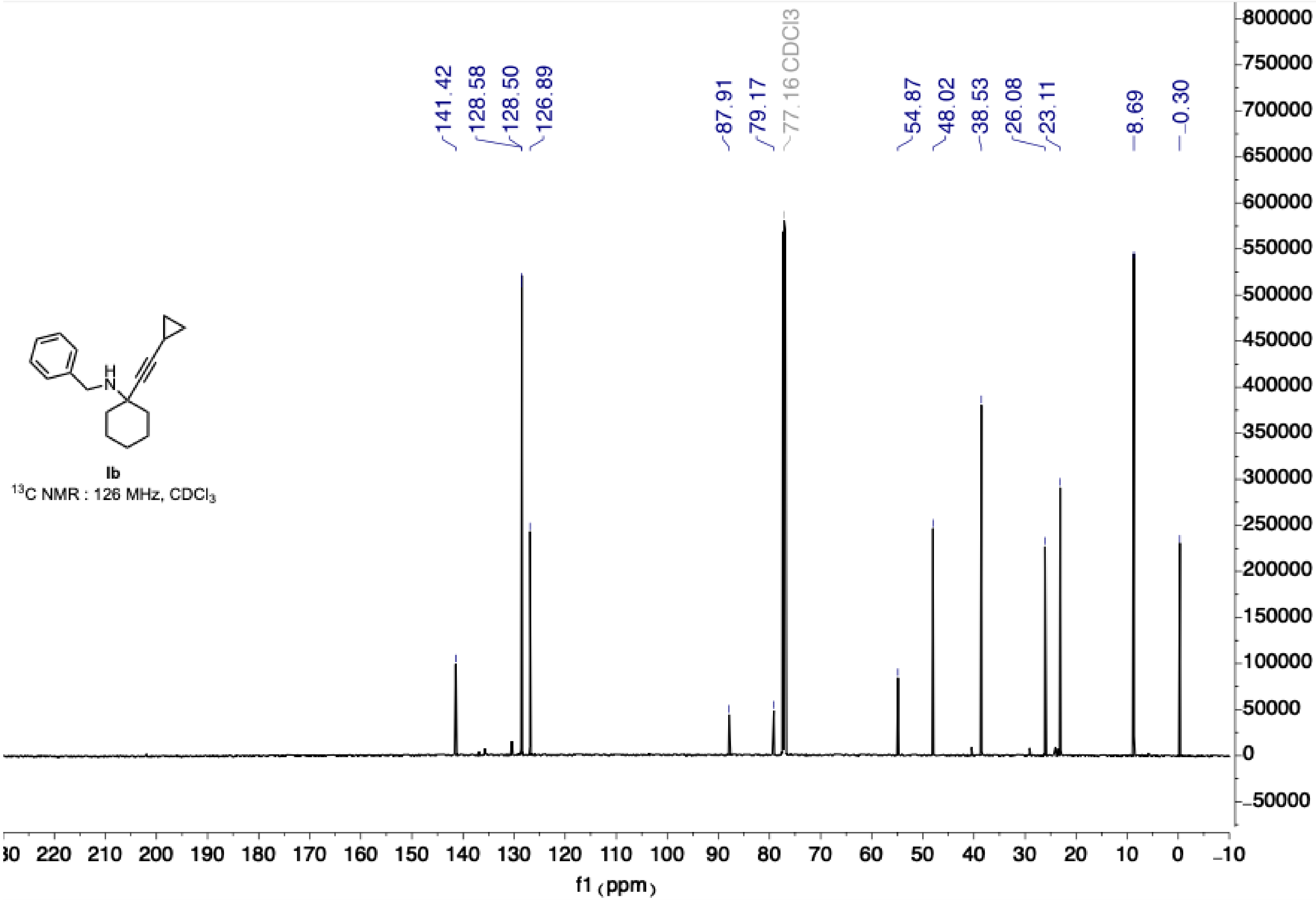

141.42
128.58
128.50
126.89
87.91
79.17
77.16 CDCl3
54.87
48.02
38.53
26.08
23.11
8.69
-0.30
Ib
^{13}C NMR : 126 MHz, $CDCl_3$
f1 (ppm)

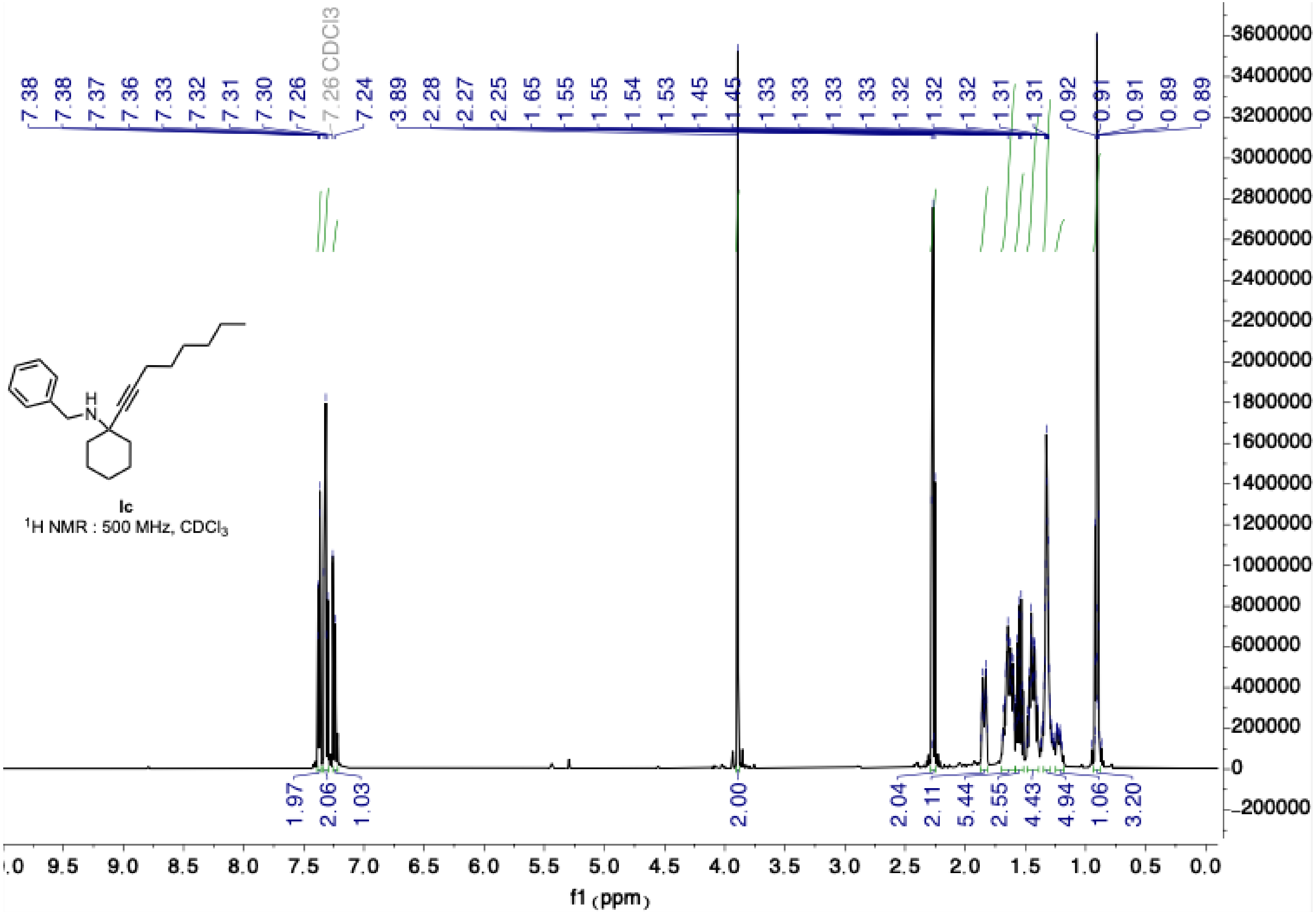

Ic
¹H NMR : 500 MHz, CDCl3
7.26 CDCl3
f1 (ppm)

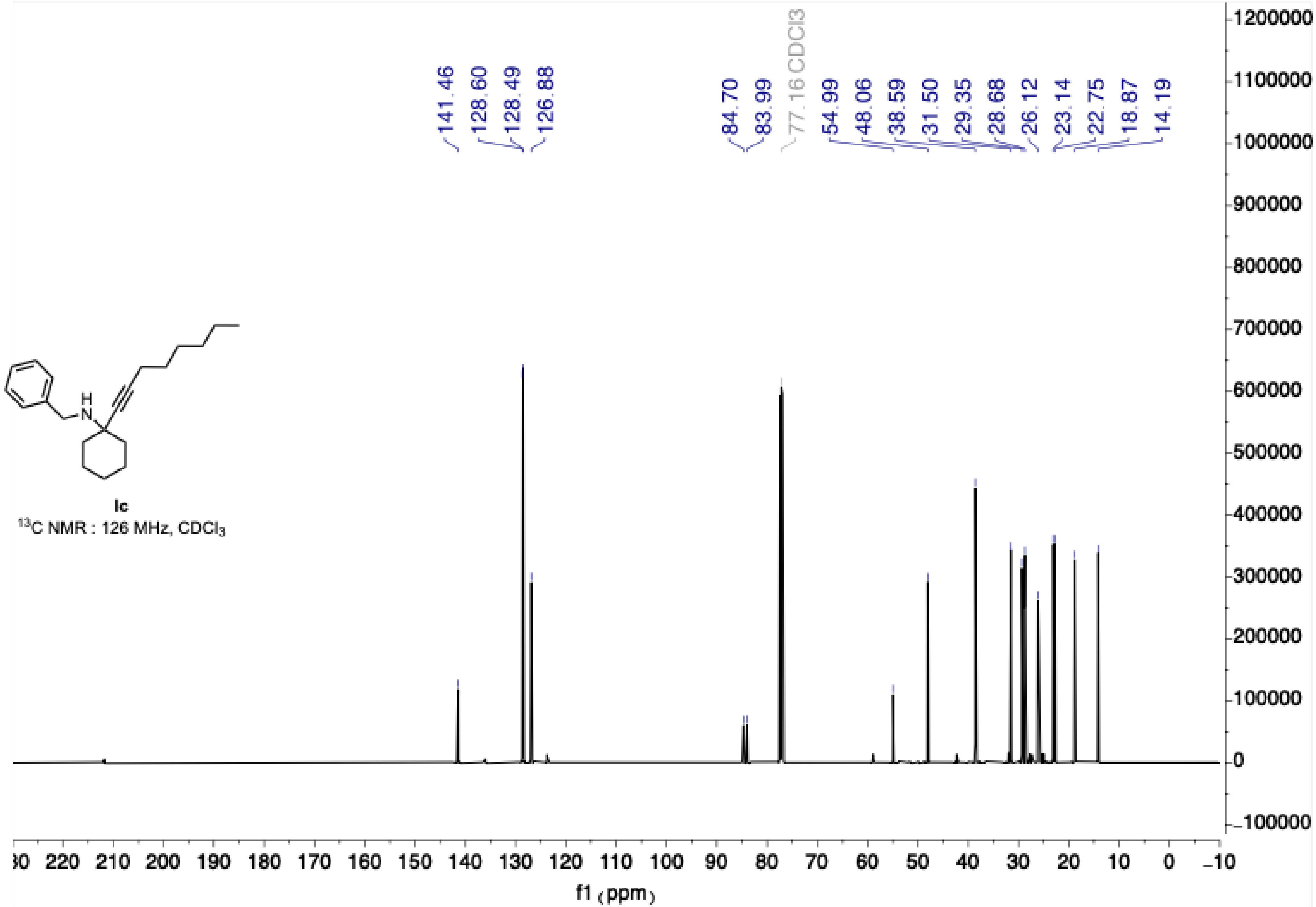
141.46
128.60
128.49
126.88
84.70
83.99
77.16 CDCl3
54.99
48.06
38.59
31.50
29.35
28.68
26.12
23.14
22.75
18.87
14.19
Ic
13C NMR : 126 MHz, CDCl3
f1 (ppm)

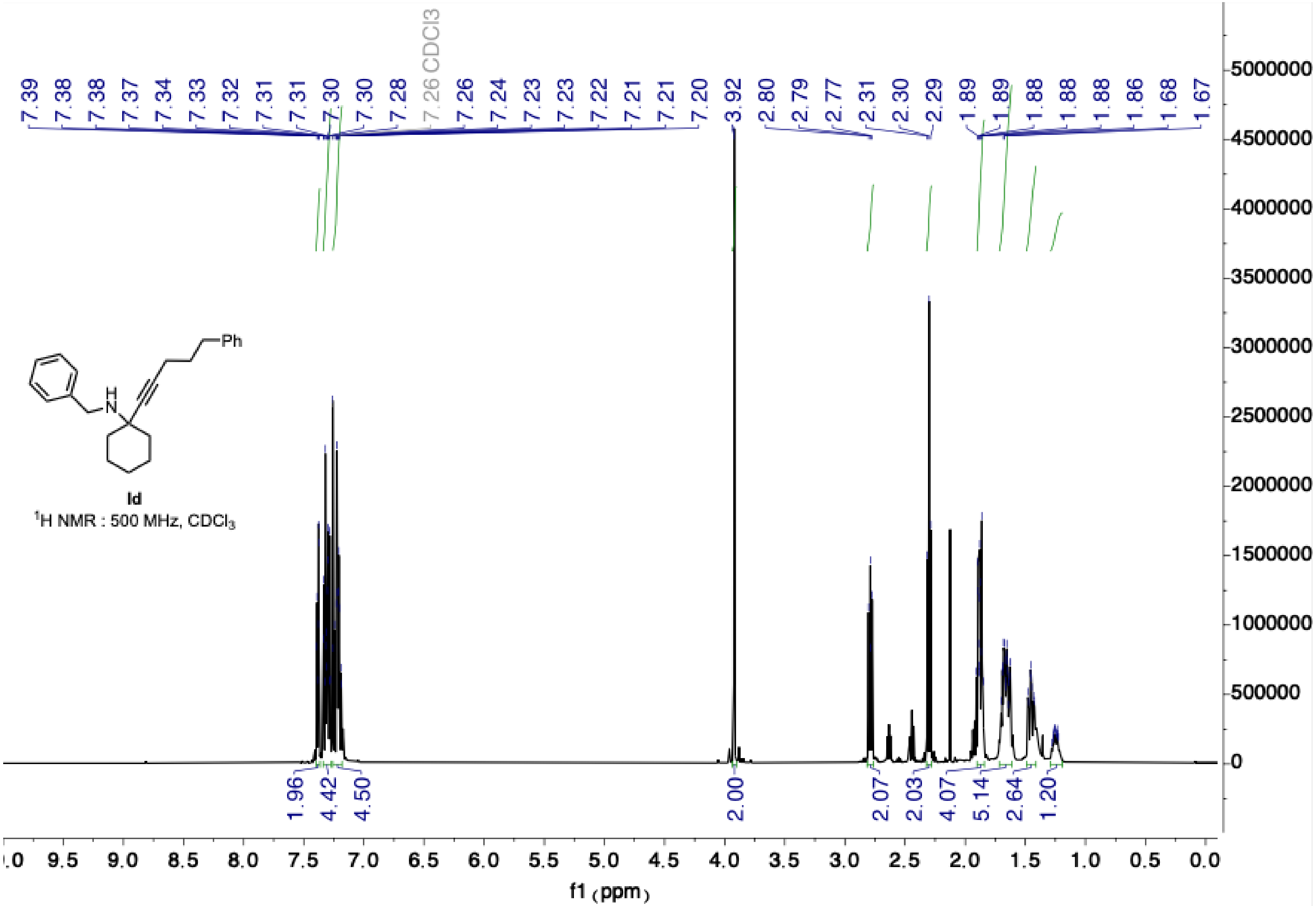

Ph
H
N
Id
1H NMR : 500 MHz, CDCl3
7.39 7.38 7.38 7.37 7.34 7.33 7.32 7.31 7.31 7.30 7.30 7.28 7.26 CDCl3 7.26 7.24 7.23 7.23 7.22 7.21 7.21 7.20 3.92 2.80 2.79 2.77 2.31 2.30 2.29 1.89 1.89 1.88 1.88 1.88 1.86 1.68 1.67
1.96 4.42 4.50 2.00 2.07 2.03 4.07 5.14 2.64 1.20
5000000 4500000 4000000 3500000 3000000 2500000 2000000 1500000 1000000 500000 0
.0 9.5 9.0 8.5 8.0 7.5 7.0 6.5 6.0 5.5 5.0 4.5 4.0 3.5 3.0 2.5 2.0 1.5 1.0 0.5 0.0
f1 (ppm)

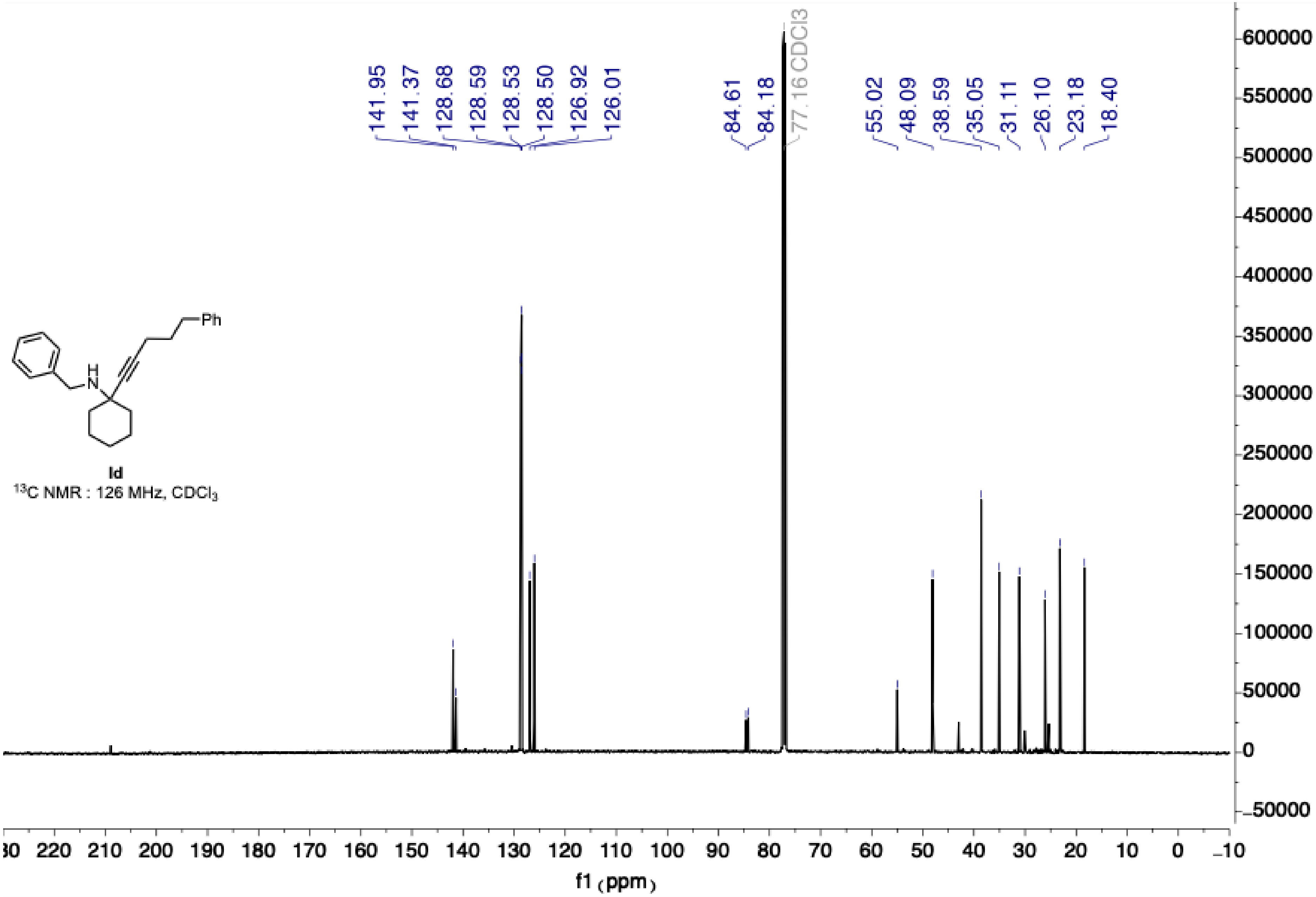
141.95
141.37
128.68
128.59
128.53
128.50
126.92
126.01
84.61
84.18
77.16 CDCl3
55.02
48.09
38.59
35.05
31.11
26.10
23.18
18.40
Ph
H
N
Id
13C NMR : 126 MHz, CDCl3
f1 (ppm)

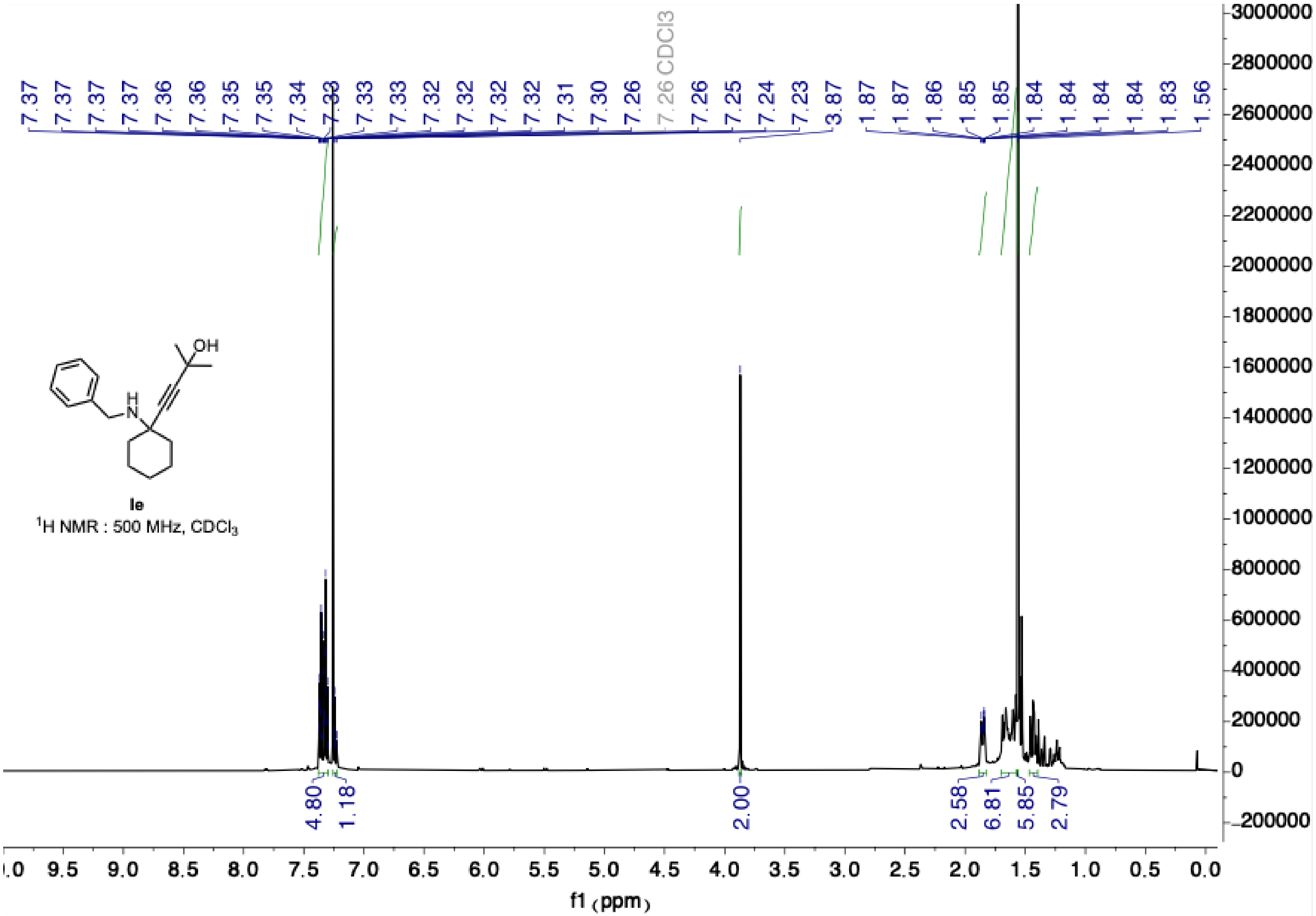
7.37 7.37 7.37 7.37 7.36 7.36 7.35 7.35 7.34 7.33 7.33 7.33 7.32 7.32 7.32 7.32 7.31 7.30 7.26 7.26 CDCl3 7.26 7.25 7.24 7.23 3.87 1.87 1.87 1.86 1.85 1.85 1.84 1.84 1.84 1.84 1.83 1.56
OH
H
N
Ie
1H NMR : 500 MHz, CDCl3
4.80 1.18 2.00 2.58 6.81 5.85 2.79
f1 (ppm)

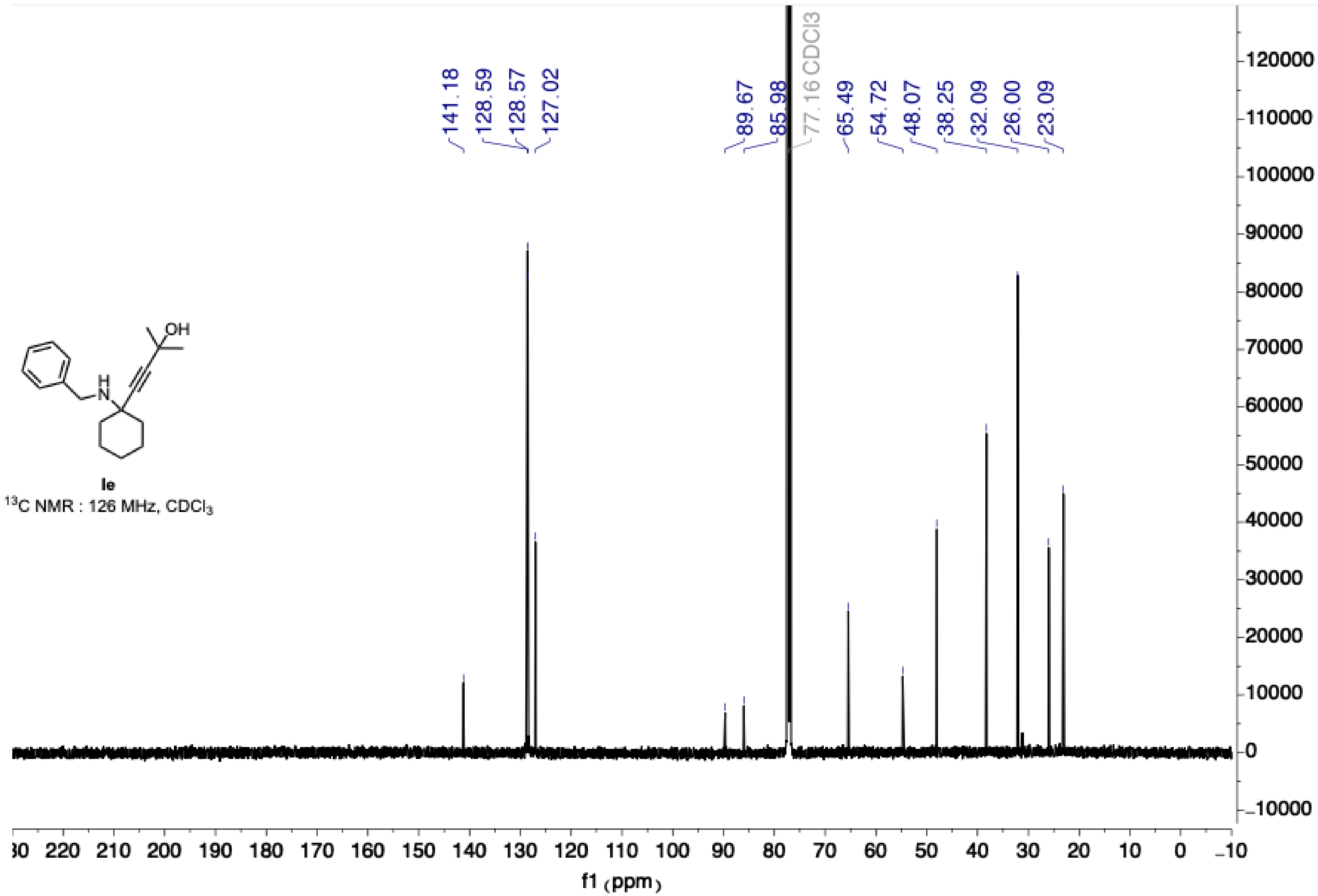
141.18
128.59
128.57
127.02
89.67
85.98
77.16 CDCl3
65.49
54.72
48.07
38.25
32.09
26.00
23.09
OH
H
N
Ie
^{13}C NMR : 126 MHz, CDCl$_3$
f1 (ppm)

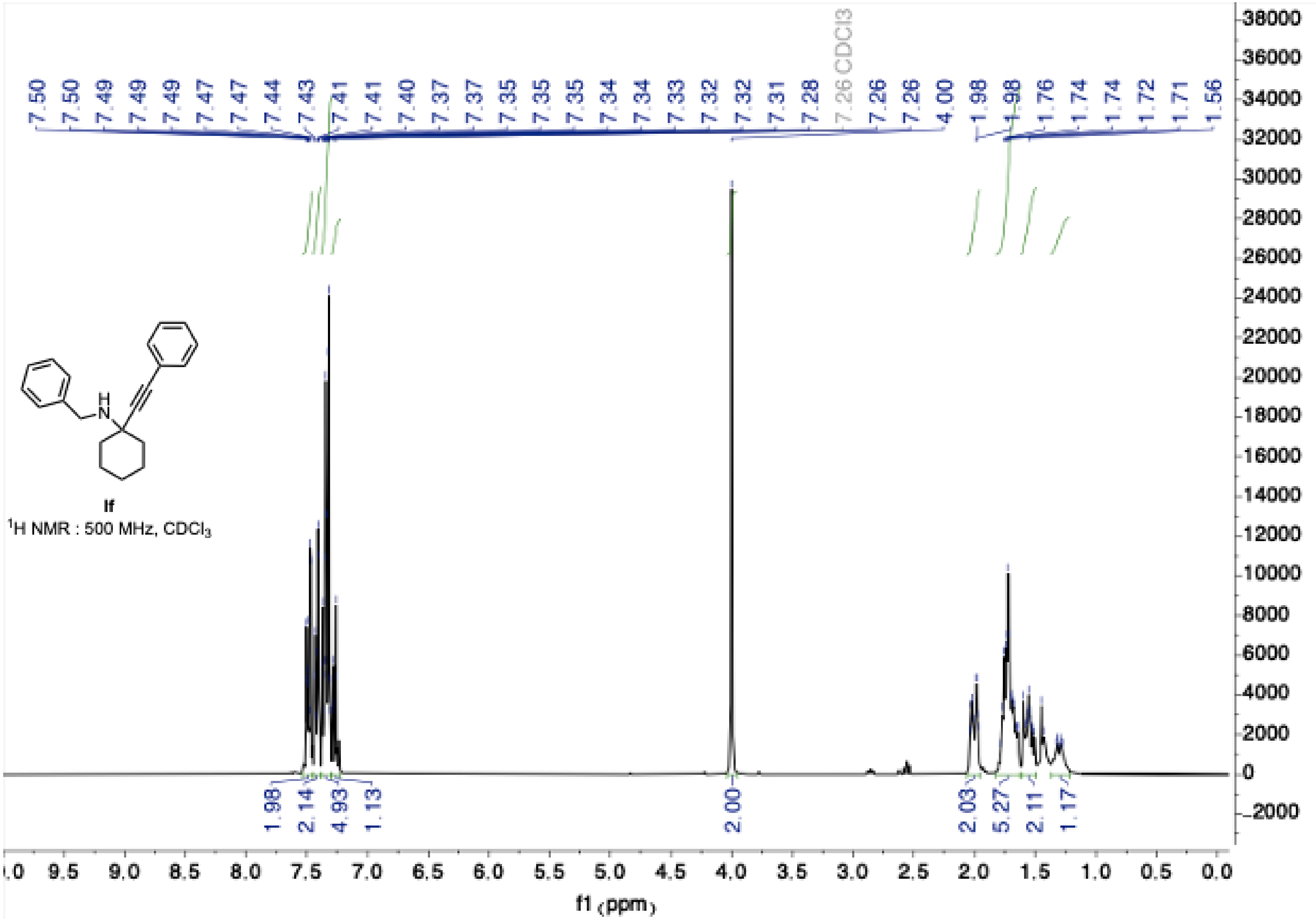

If
^{1}H NMR : 500 MHz, CDCl$_3$
7.50 7.50 7.49 7.49 7.49 7.47 7.47 7.44 7.43 7.41 7.41 7.40 7.37 7.37 7.35 7.35 7.35 7.34 7.34 7.33 7.32 7.32 7.31 7.28 7.26 CDCl3 7.26 7.26 4.00 1.98 1.98 1.76 1.74 1.74 1.72 1.71 1.56
1.98 2.14 4.93 1.13 2.00 2.03 5.27 2.11 1.17
f1 (ppm)

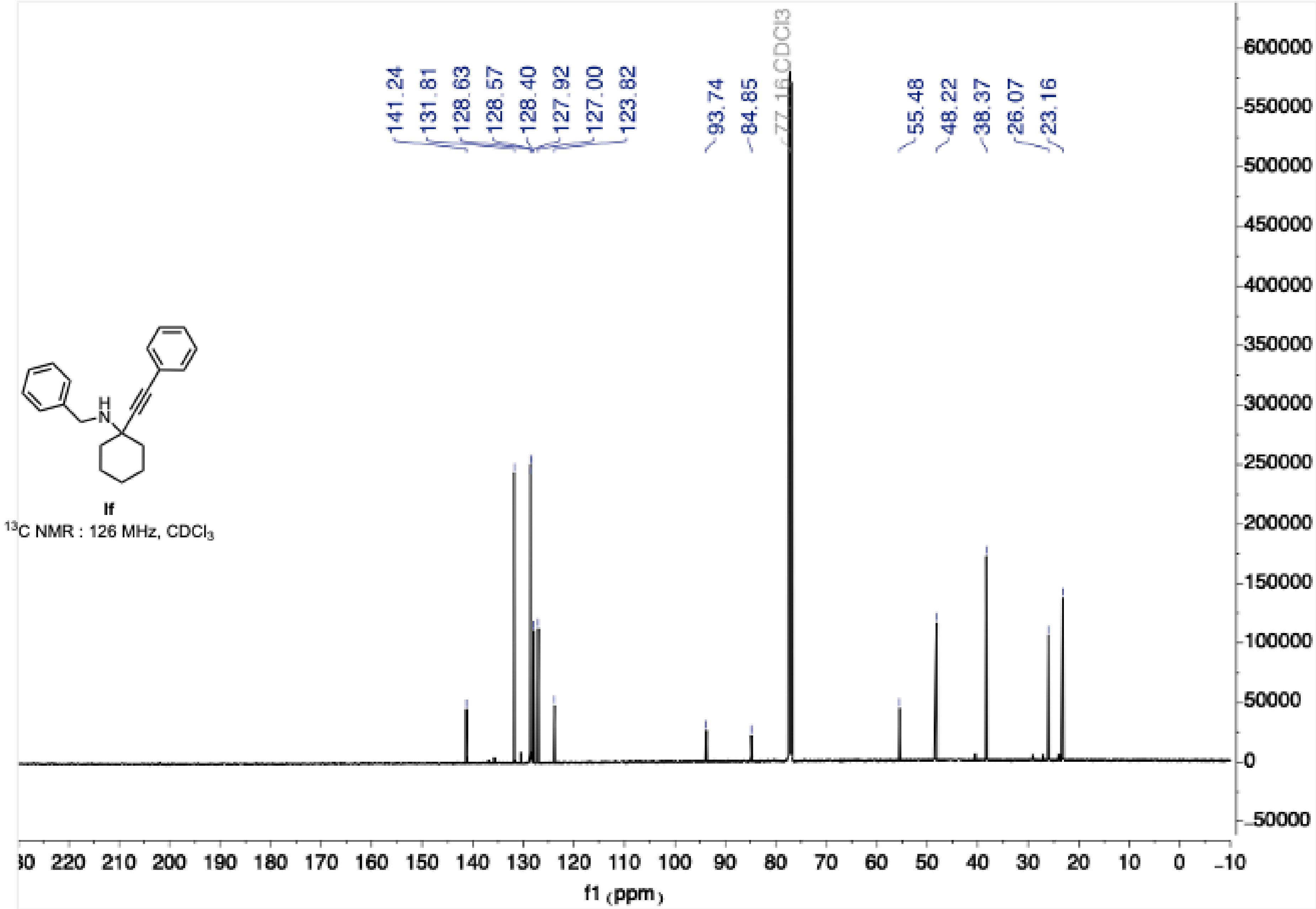
141.24
131.81
128.63
128.57
128.40
127.92
127.00
123.82
93.74
84.85
77.16 CDCl3
55.48
48.22
38.37
26.07
23.16
If
13C NMR : 126 MHz, CDCl3
f1 (ppm)

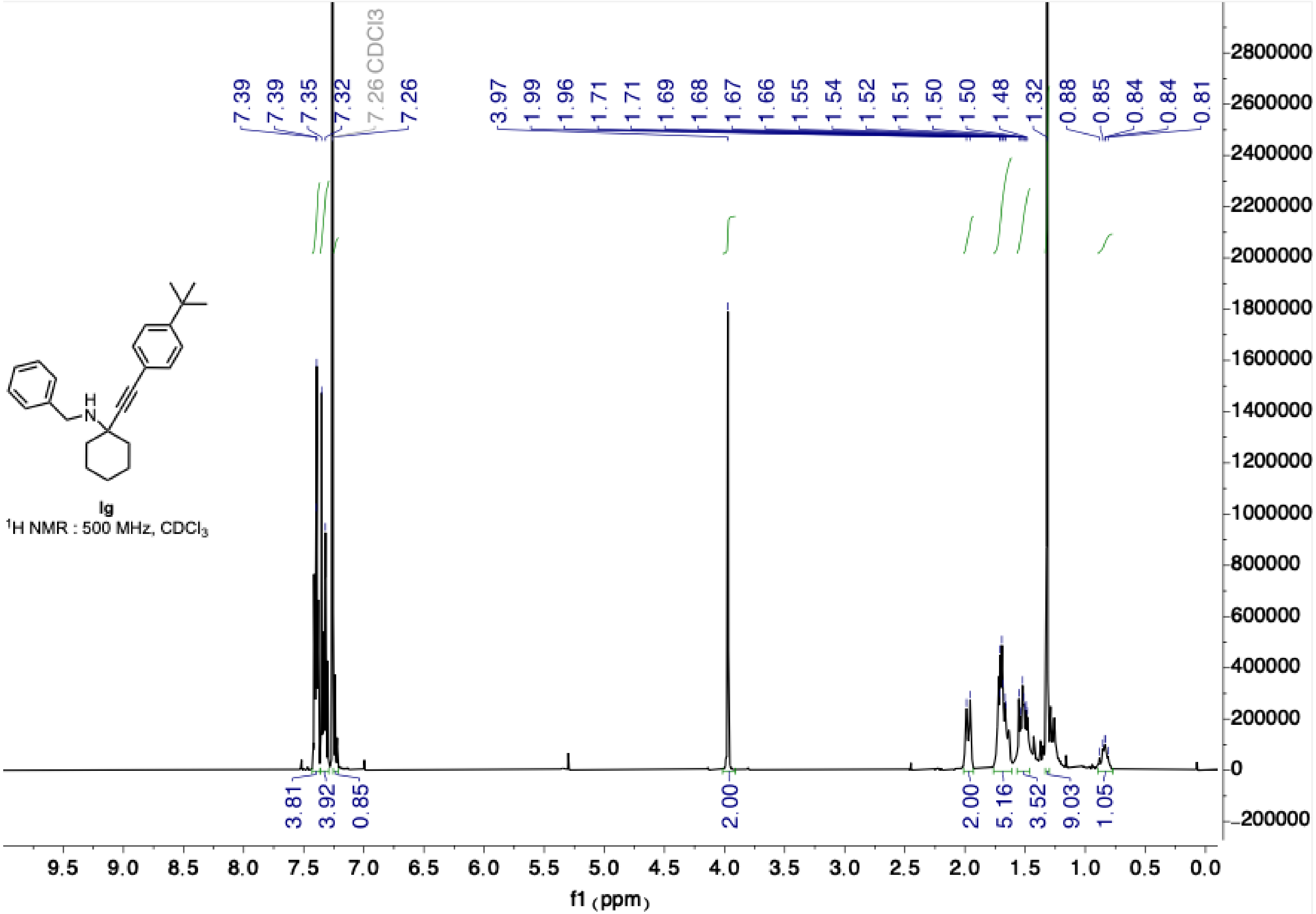

Ig

^{1}H NMR : 500 MHz, $CDCl_3$

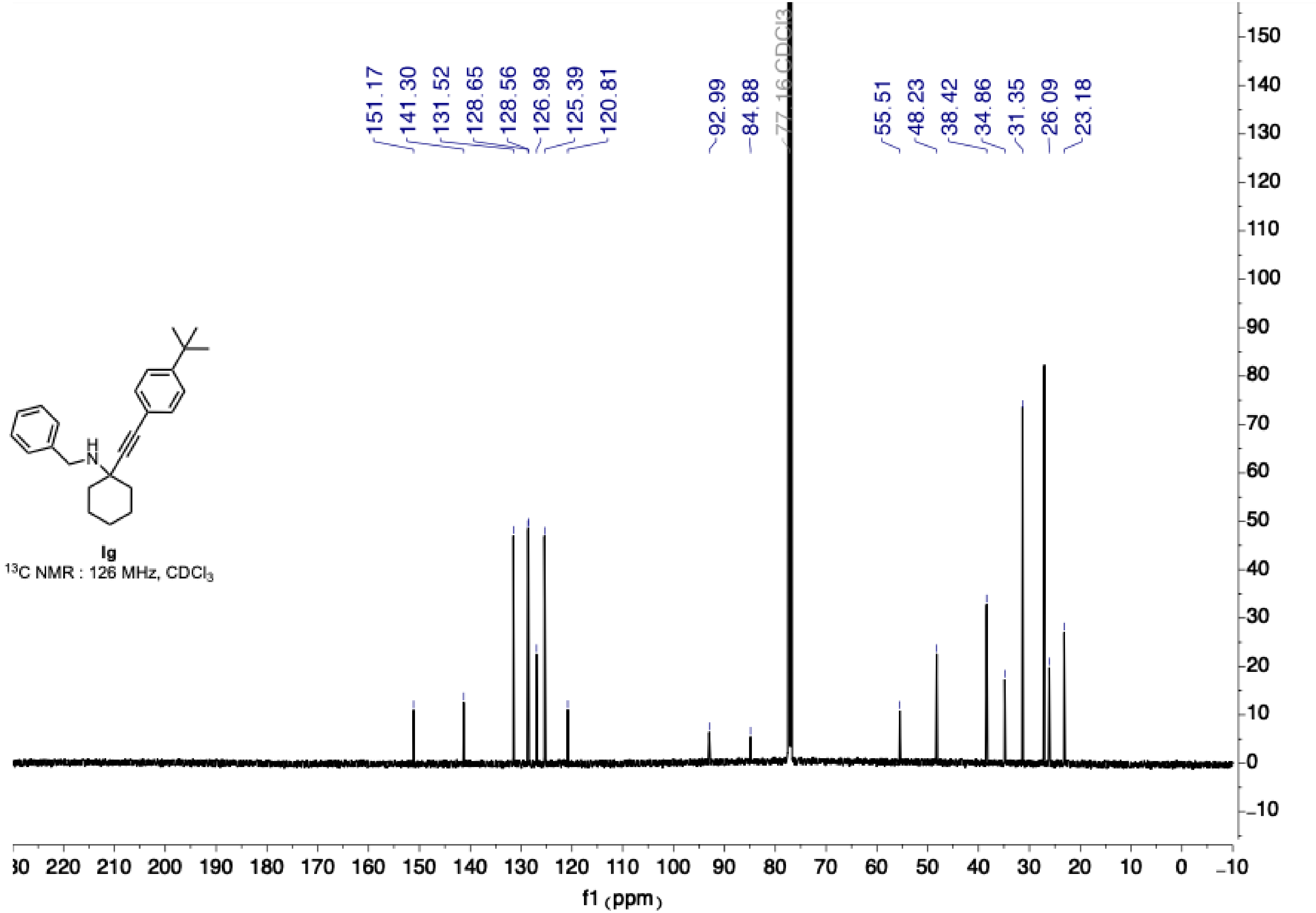
151.17
141.30
131.52
128.65
128.56
126.98
125.39
120.81
92.99
84.88
77.16 CDCl3
55.51
48.23
38.42
34.86
31.35
26.09
23.18
Ig
^{13}C NMR : 126 MHz, $CDCl_3$
f1 (ppm)

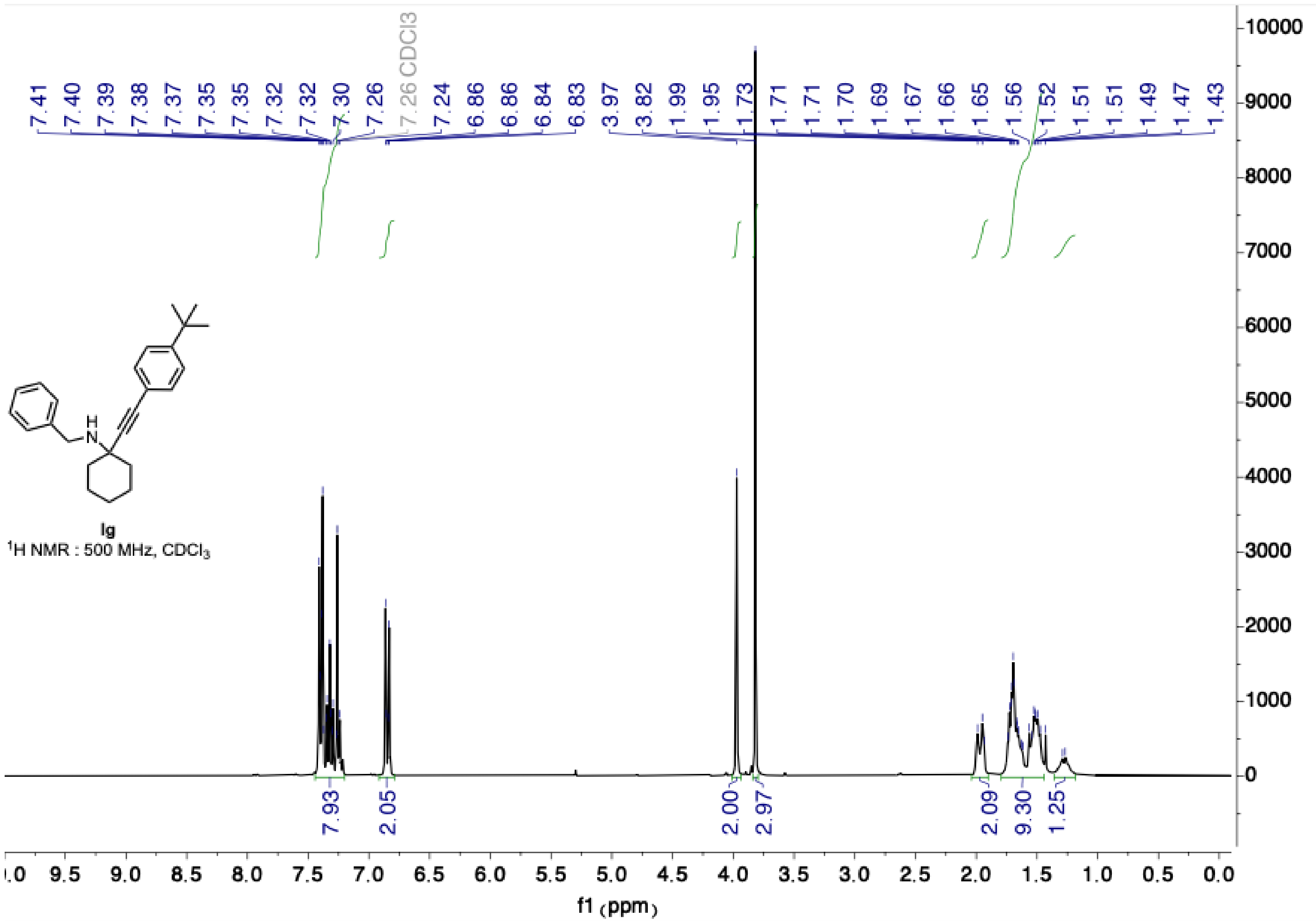

7.41 7.40 7.39 7.38 7.37 7.35 7.35 7.32 7.32 7.30 7.26 7.26 CDCl3 7.24 6.86 6.86 6.84 6.83 3.97 3.82 1.99 1.95 1.73 1.71 1.71 1.70 1.69 1.67 1.66 1.65 1.56 1.52 1.51 1.51 1.49 1.47 1.43
Ig
^{1}H NMR : 500 MHz, CDCl$_3$
7.93 2.05 2.00 2.97 2.09 9.30 1.25
f1 (ppm)

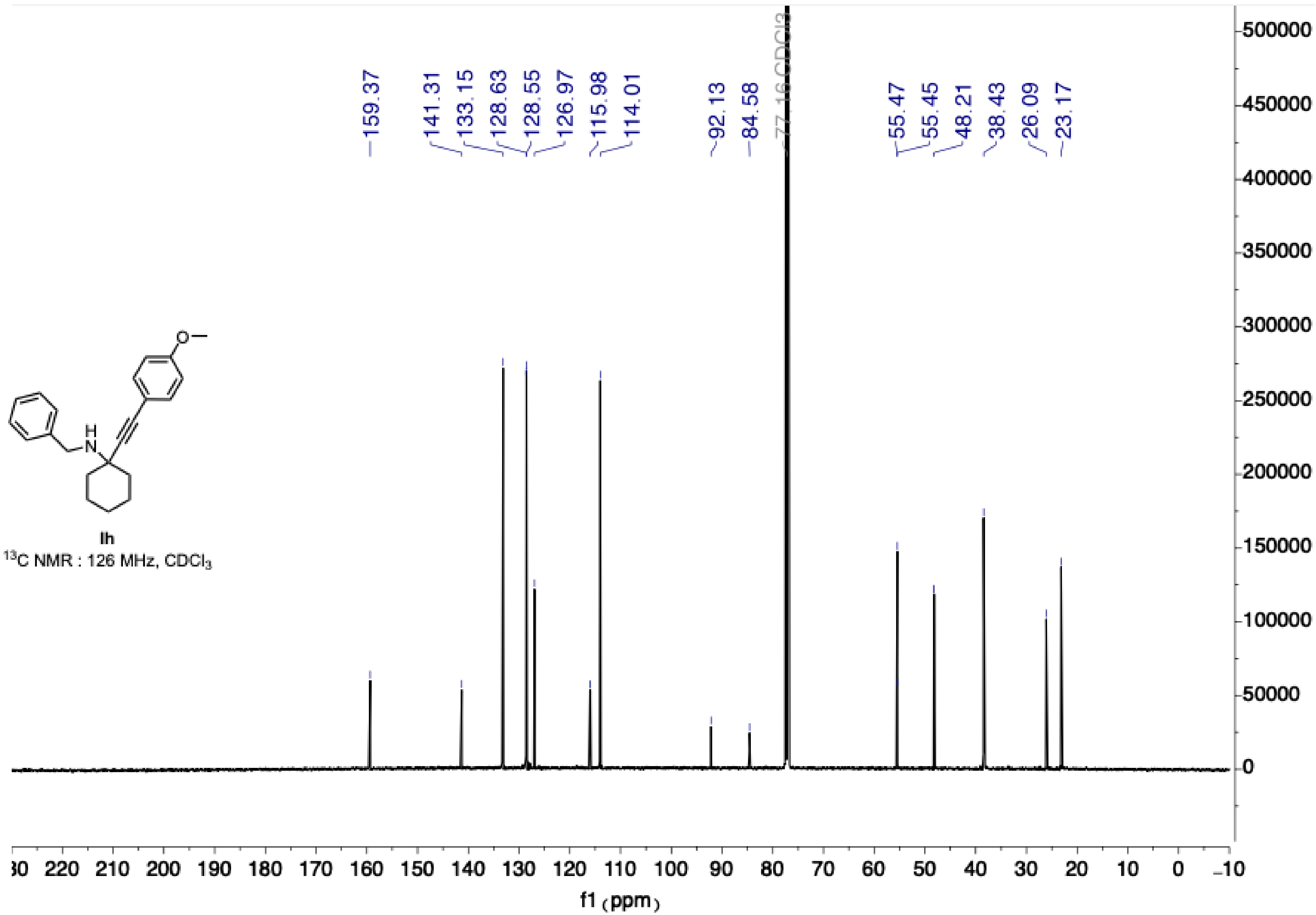
159.37
141.31
133.15
128.63
128.55
126.97
115.98
114.01
92.13
84.58
77.16 CDCl3
55.47
55.45
48.21
38.43
26.09
23.17
Ih
^{13}C NMR : 126 MHz, $CDCl_3$
f1 (ppm)
500000
450000
400000
350000
300000
250000
200000
150000
100000
50000
0

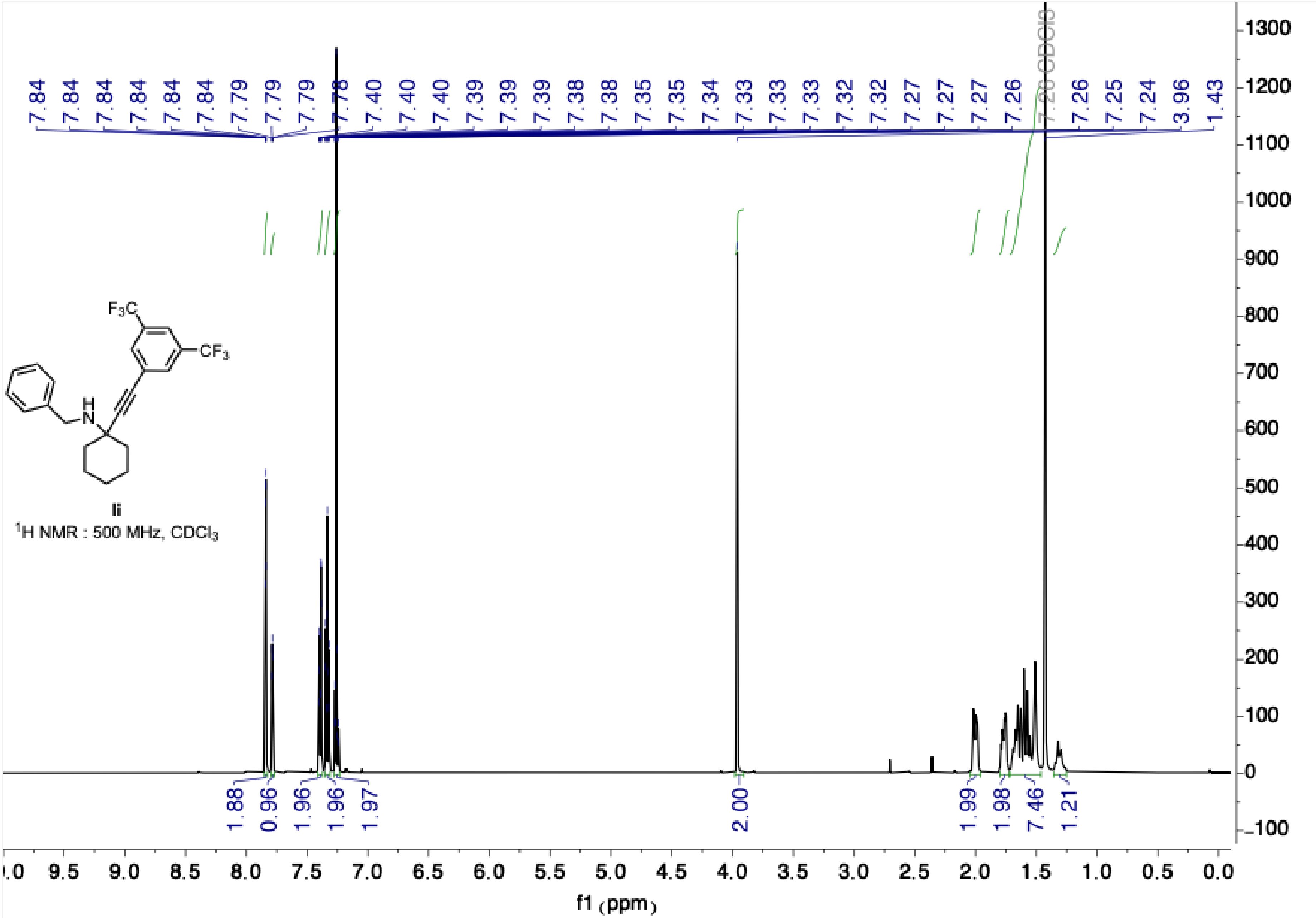

7.84 7.84 7.84 7.84 7.84 7.84 7.79 7.79 7.79 7.78 7.40 7.40 7.40 7.39 7.39 7.39 7.38 7.38 7.35 7.35 7.34 7.33 7.33 7.33 7.32 7.32 7.27 7.27 7.27 7.26 7.26 CDCl3 7.26 7.25 7.24 3.96 1.43
F3C
CF3
H
N
Ii
1H NMR : 500 MHz, CDCl3
1.88 0.96 1.96 1.96 1.97 2.00 1.99 1.98 7.46 1.21
f1 (ppm)

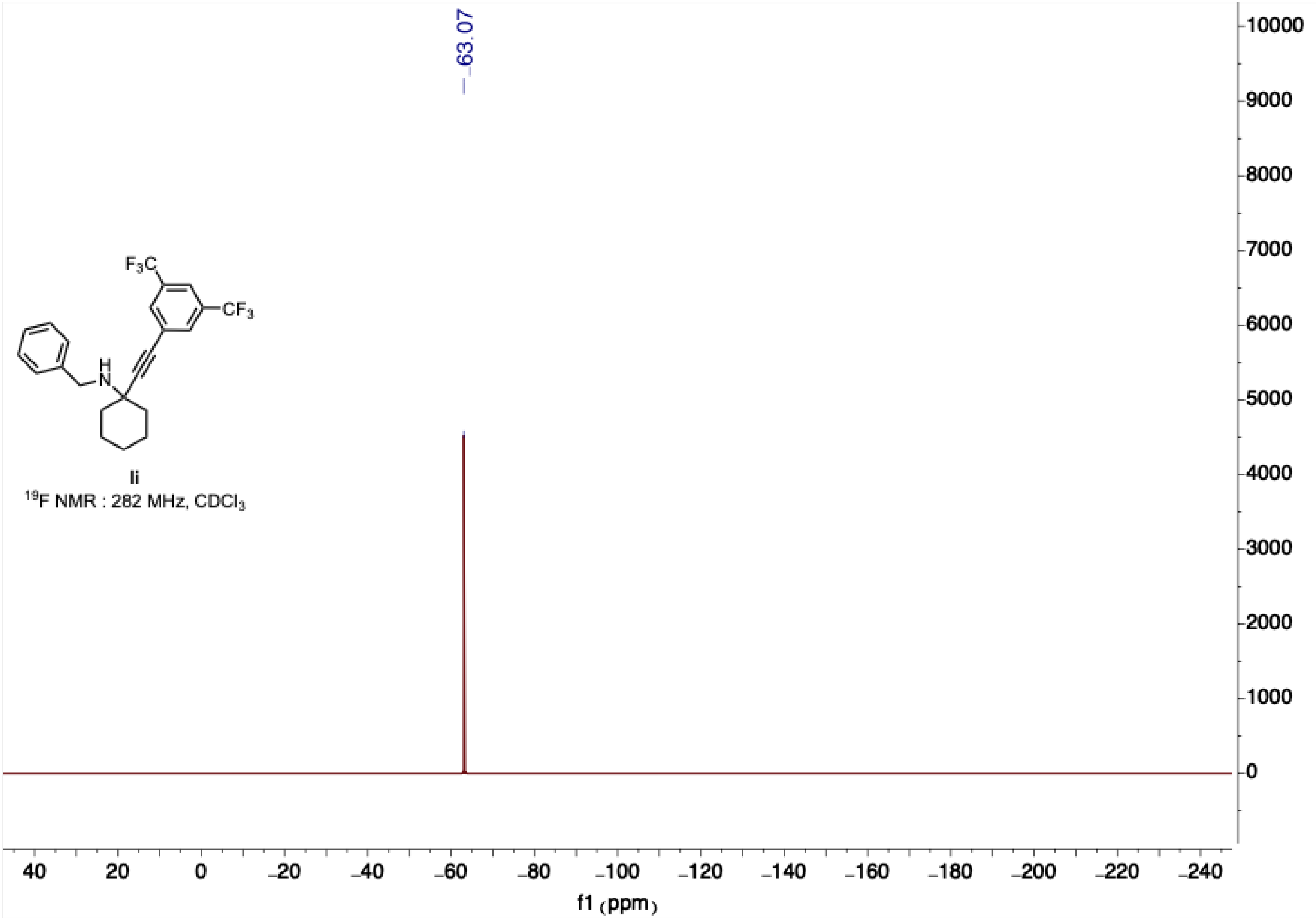

-63.07
F3C
CF3
H
N
1i
19F NMR : 282 MHz, CDCl3
10000
9000
8000
7000
6000
5000
4000
3000
2000
1000
0
40
20
0
-20
-40
-60
-80
-100
-120
-140
-160
-180
-200
-220
-240
f1 (ppm)

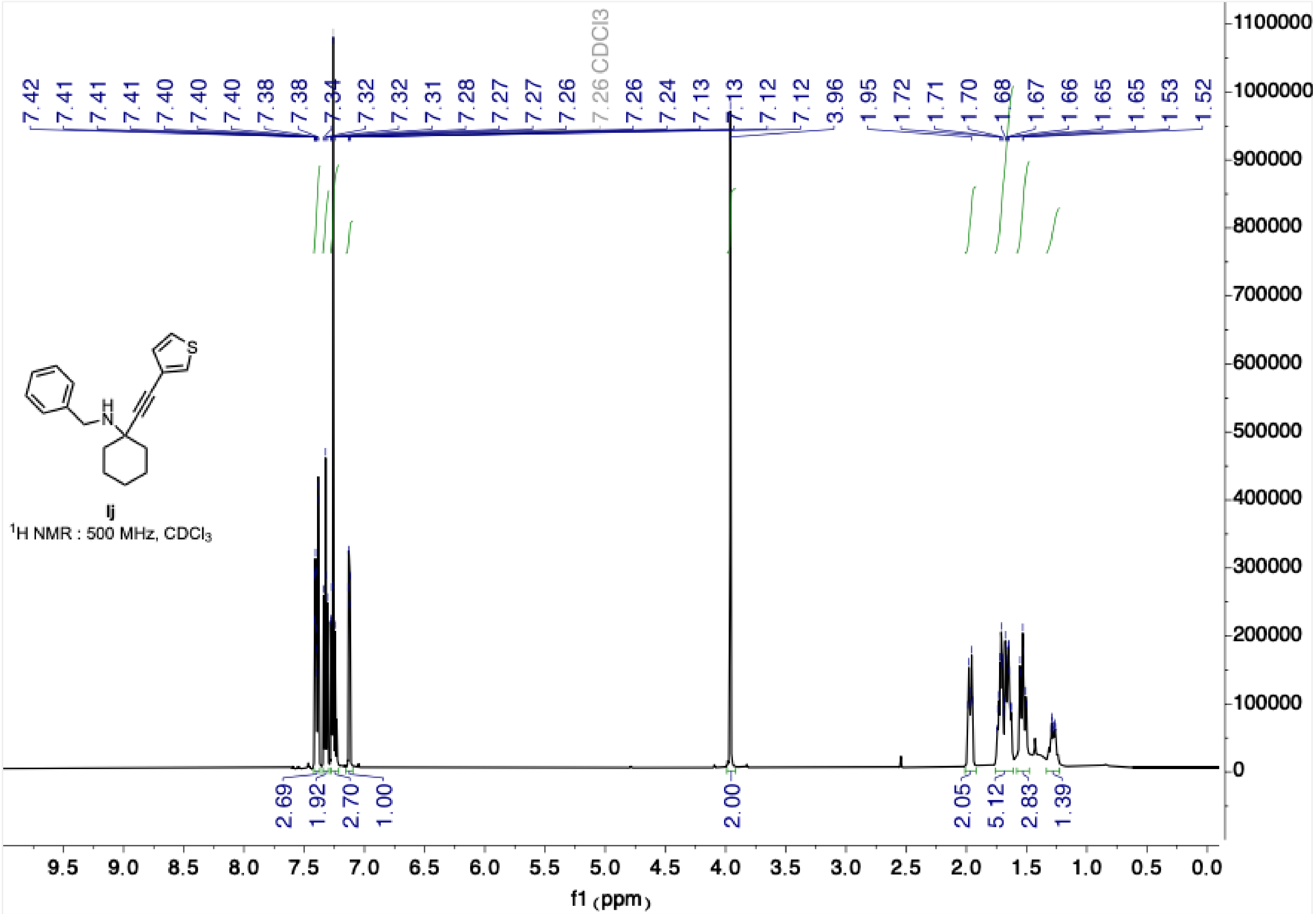
7.42 7.41 7.41 7.41 7.40 7.40 7.40 7.38 7.38 7.34 7.32 7.32 7.31 7.28 7.27 7.27 7.26 7.26 CDCl3 7.26 7.24 7.13 7.13 7.12 7.12 3.96 1.95 1.72 1.71 1.70 1.68 1.67 1.66 1.65 1.65 1.53 1.52
Ij
1H NMR : 500 MHz, CDCl3
2.69 1.92 2.70 1.00 2.00 2.05 5.12 2.83 1.39
f1 (ppm)

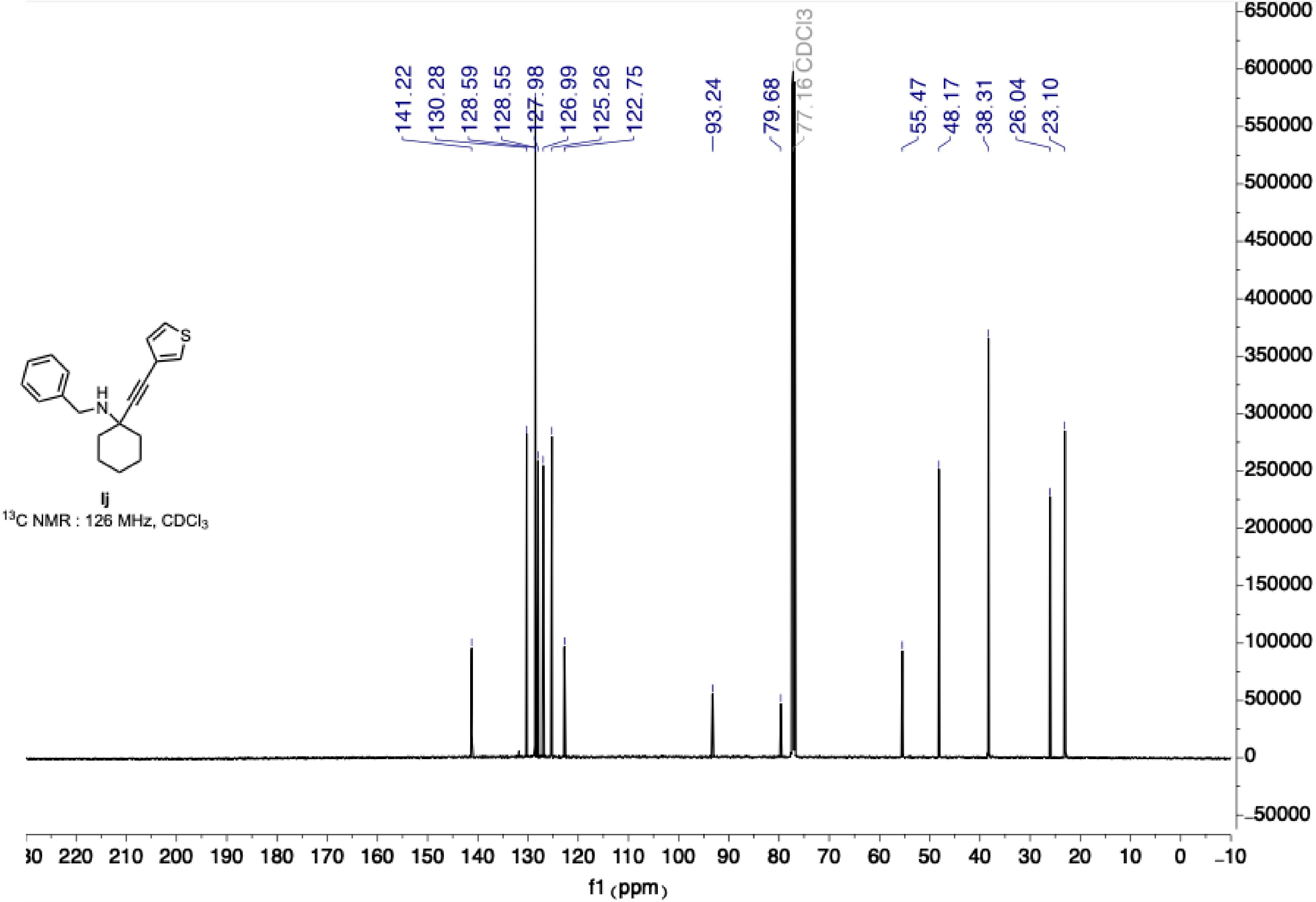
141.22
130.28
128.59
128.55
127.98
126.99
125.26
122.75
93.24
79.68
77.16 CDCl3
55.47
48.17
38.31
26.04
23.10
H
N
S
Ij
^{13}C NMR : 126 MHz, $CDCl_3$
f1 (ppm)

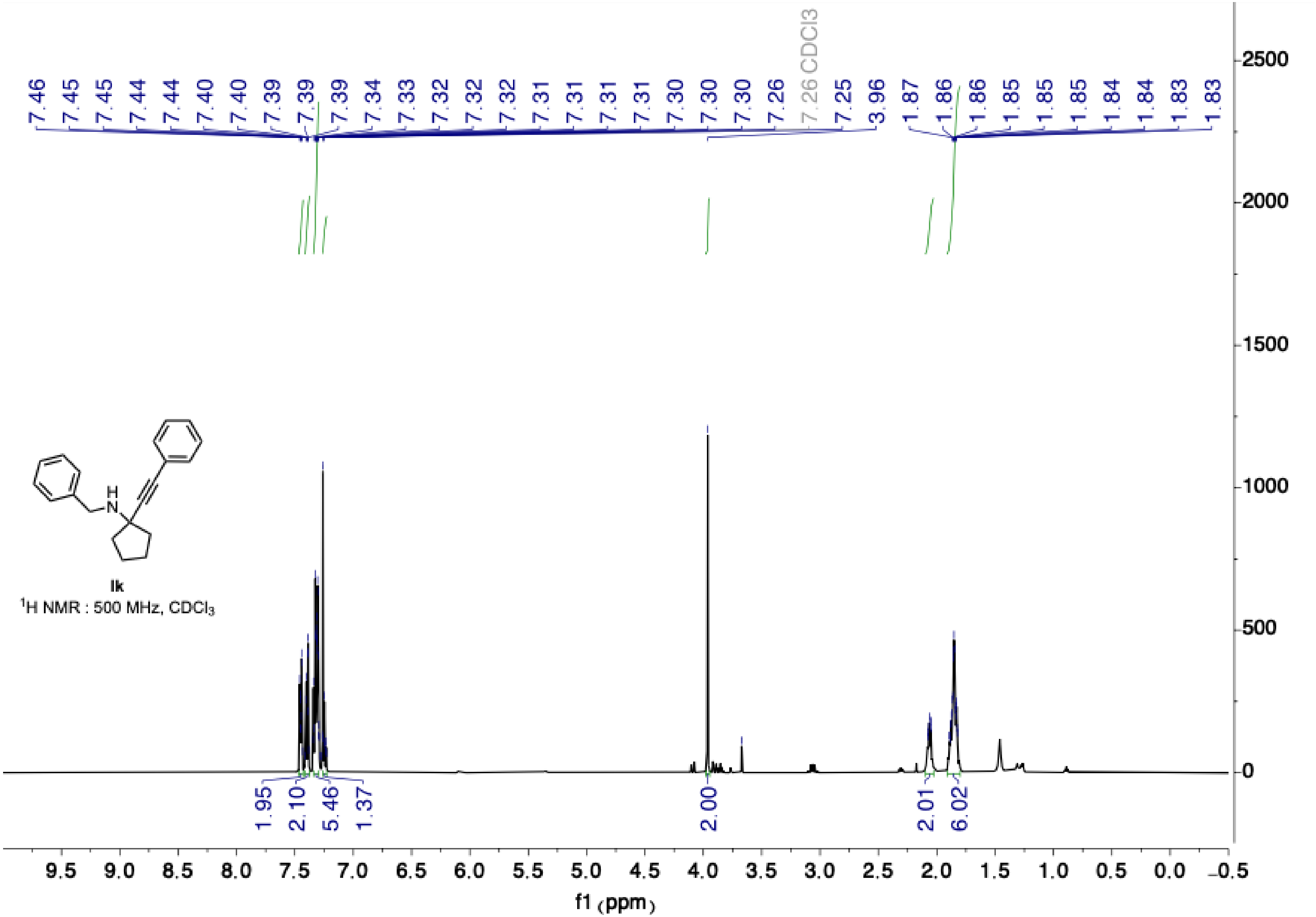

Ik
^{1}H NMR : 500 MHz, CDCl$_3$
f1 (ppm)

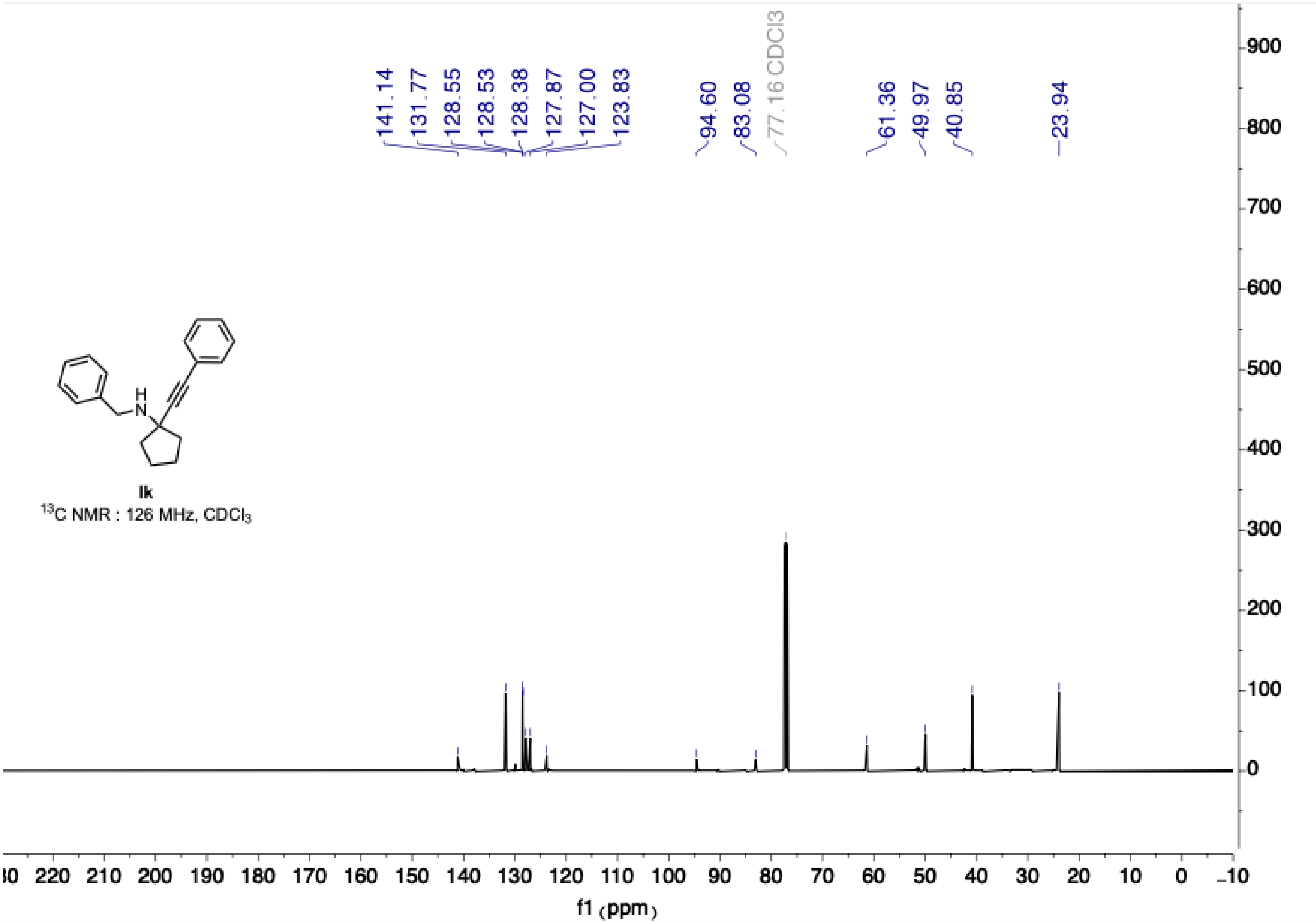
141.14
131.77
128.55
128.53
128.38
127.87
127.00
123.83
94.60
83.08
77.16 CDCl3
61.36
49.97
40.85
23.94
Ik
13C NMR : 126 MHz, CDCl3
f1 (ppm)

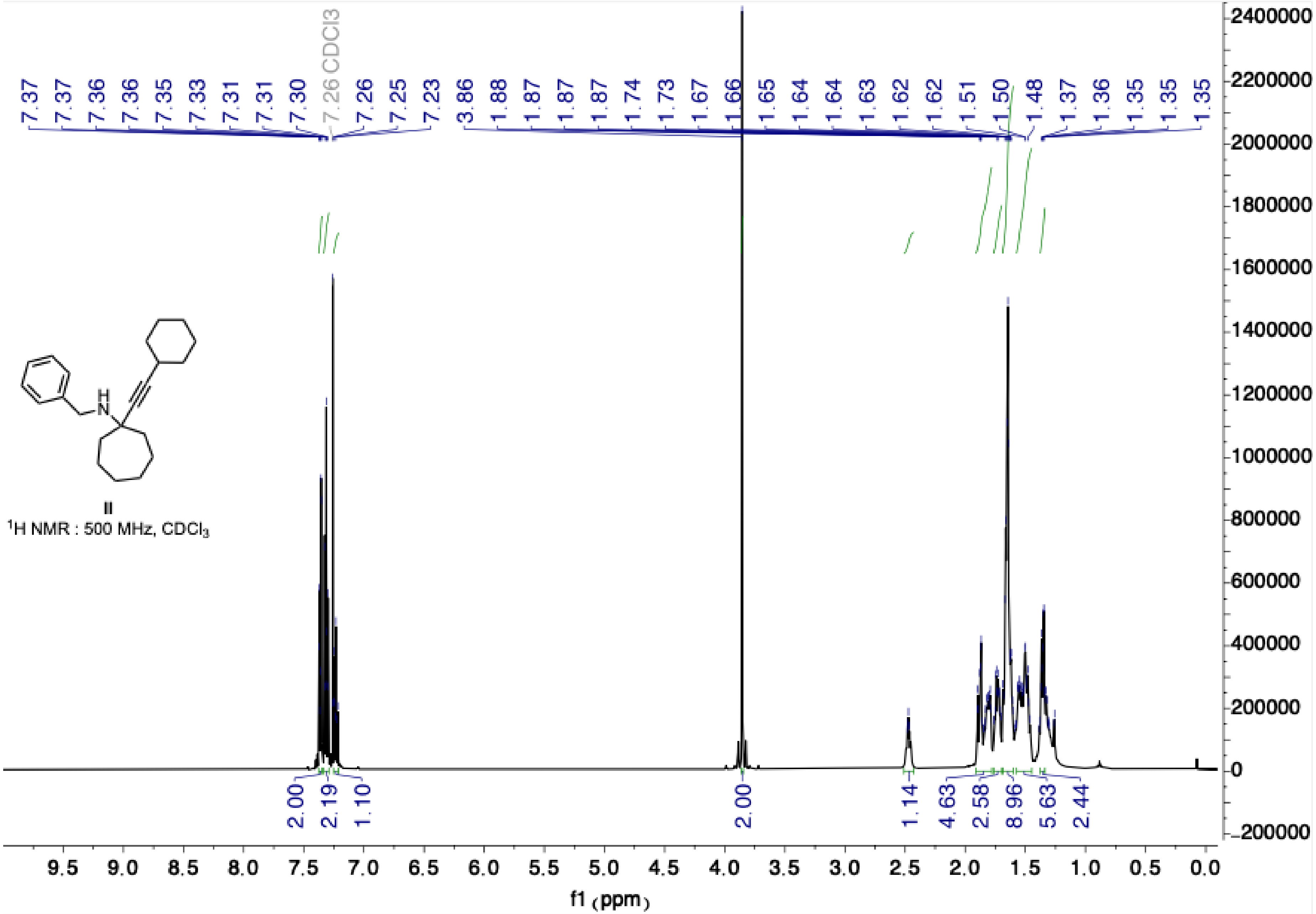

7.37 7.37 7.36 7.36 7.35 7.33 7.31 7.31 7.30 7.26 CDCl3 7.26 7.25 7.23 3.86 1.88 1.87 1.87 1.87 1.74 1.73 1.67 1.66 1.65 1.64 1.64 1.63 1.62 1.62 1.51 1.50 1.48 1.37 1.36 1.35 1.35 1.35
II
^{1}H NMR : 500 MHz, CDCl$_3$
2.00 2.19 1.10 2.00 1.14 4.63 2.58 8.96 5.63 2.44
f1 (ppm)

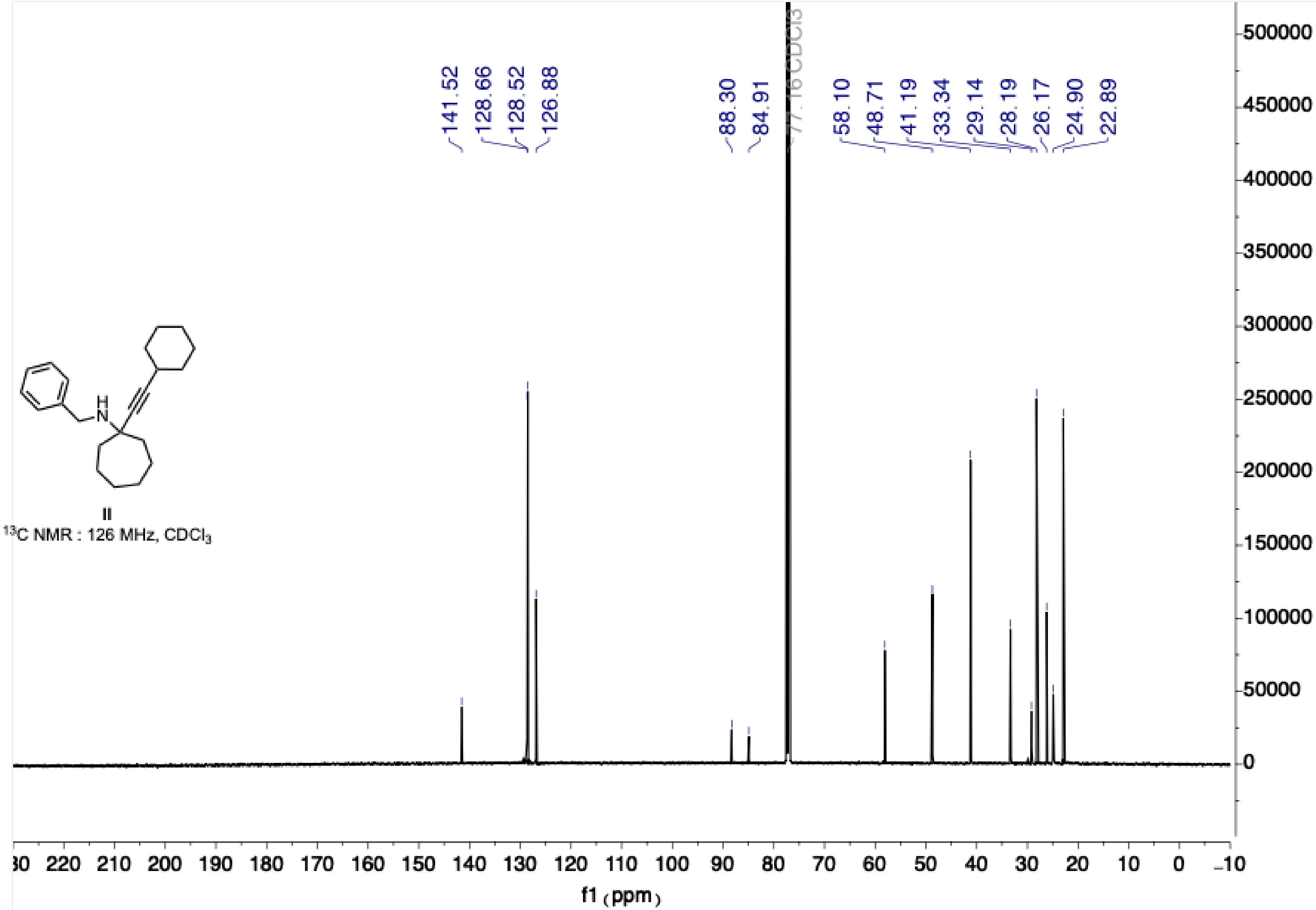

141.52
128.66
128.52
126.88
88.30
84.91
77.16 CDCl3
58.10
48.71
41.19
33.34
29.14
28.19
26.17
24.90
22.89
H
N
II
13C NMR : 126 MHz, CDCl3
500000
450000
400000
350000
300000
250000
200000
150000
100000
50000
0
30 220 210 200 190 180 170 160 150 140 130 120 110 100 90 80 70 60 50 40 30 20 10 0 -10
f1 (ppm)

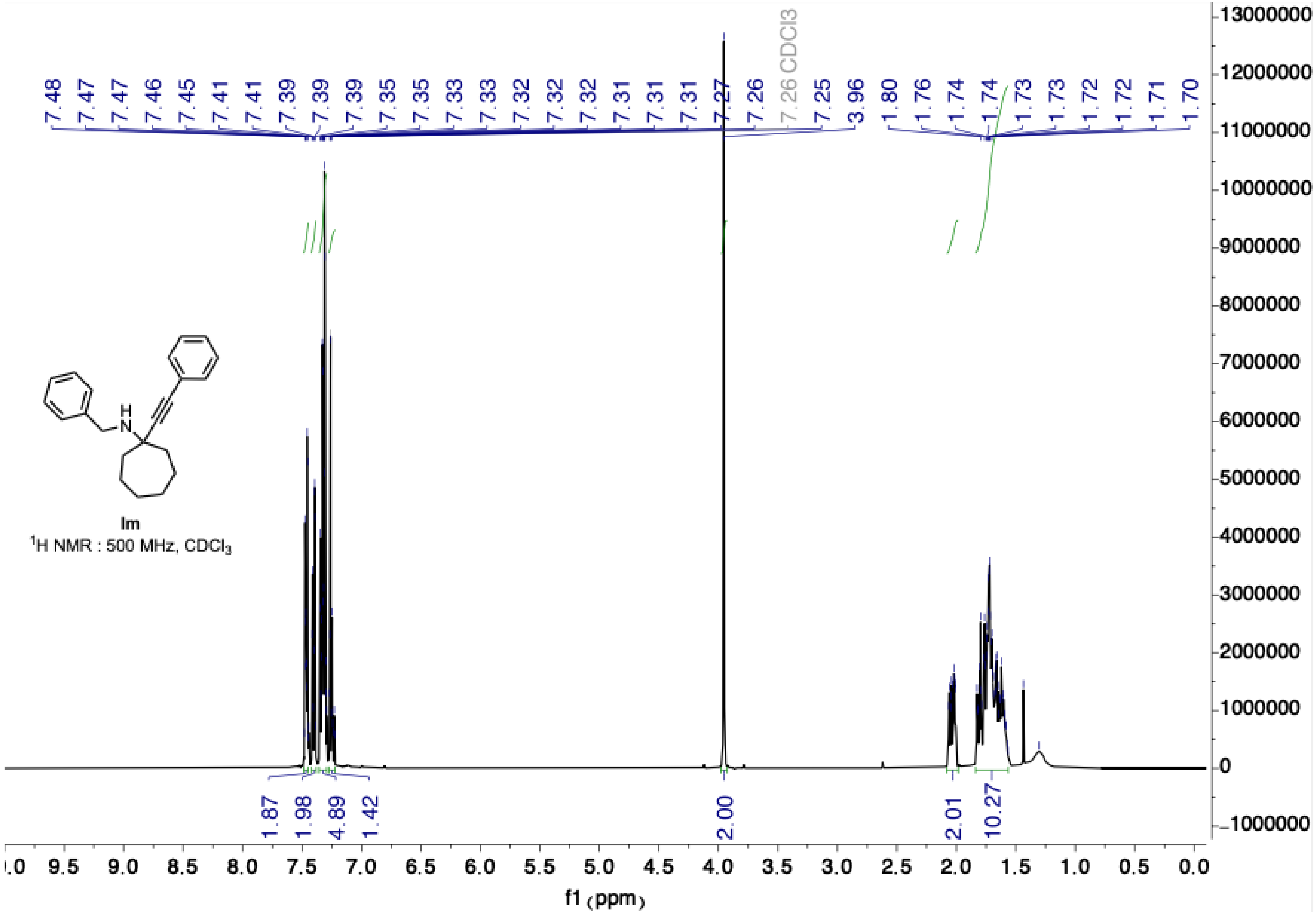

7.48 7.47 7.47 7.46 7.45 7.41 7.41 7.39 7.39 7.39 7.35 7.35 7.33 7.33 7.32 7.32 7.32 7.31 7.31 7.31 7.27 7.26 7.26 CDCl3 7.25 3.96 1.80 1.76 1.74 1.74 1.73 1.73 1.72 1.72 1.71 1.70
Im
^{1}H NMR : 500 MHz, $CDCl_3$
1.87 1.98 4.89 1.42 2.00 2.01 10.27
f1 (ppm)

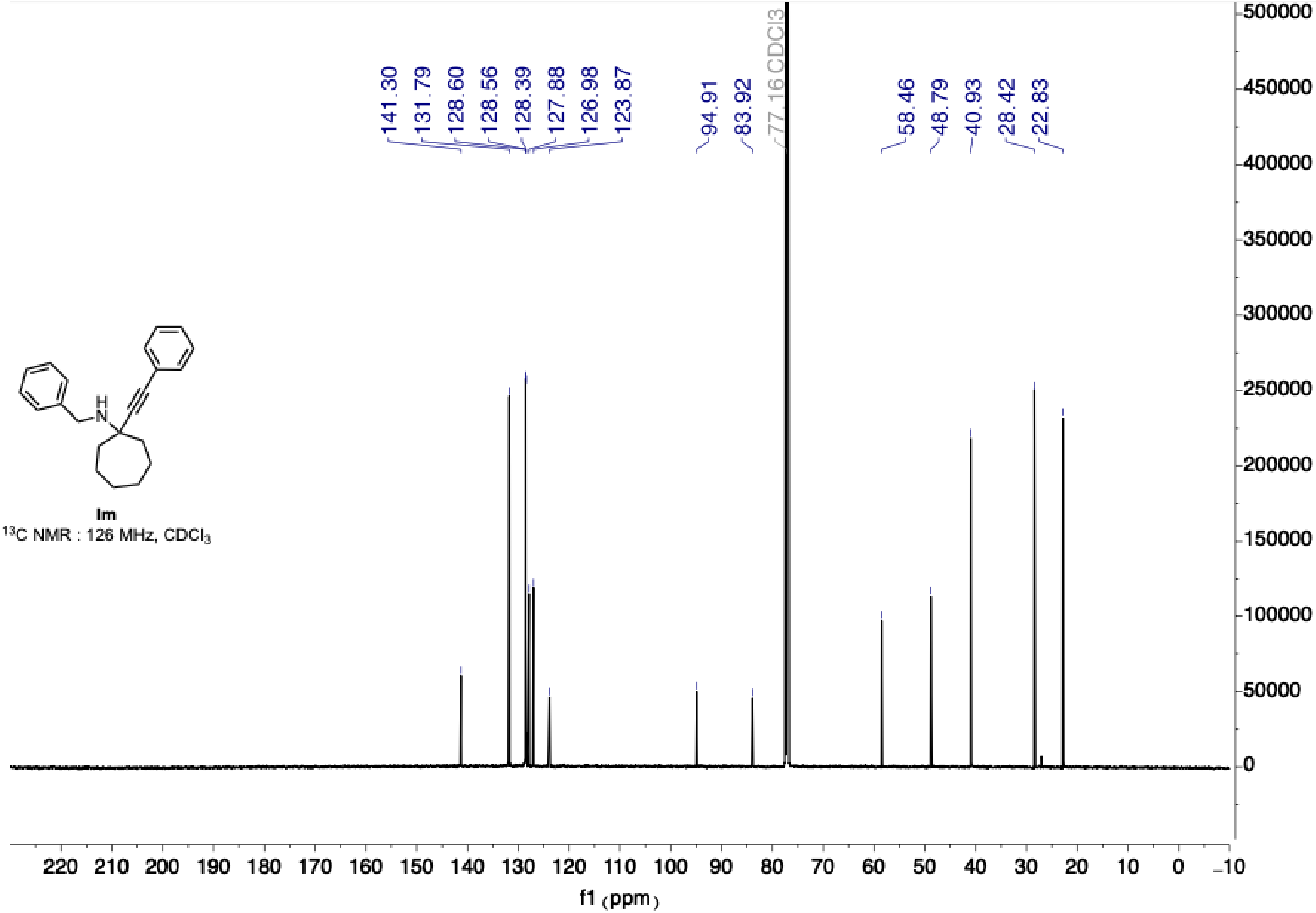

Im

^{13}C NMR : 126 MHz, $CDCl_3$

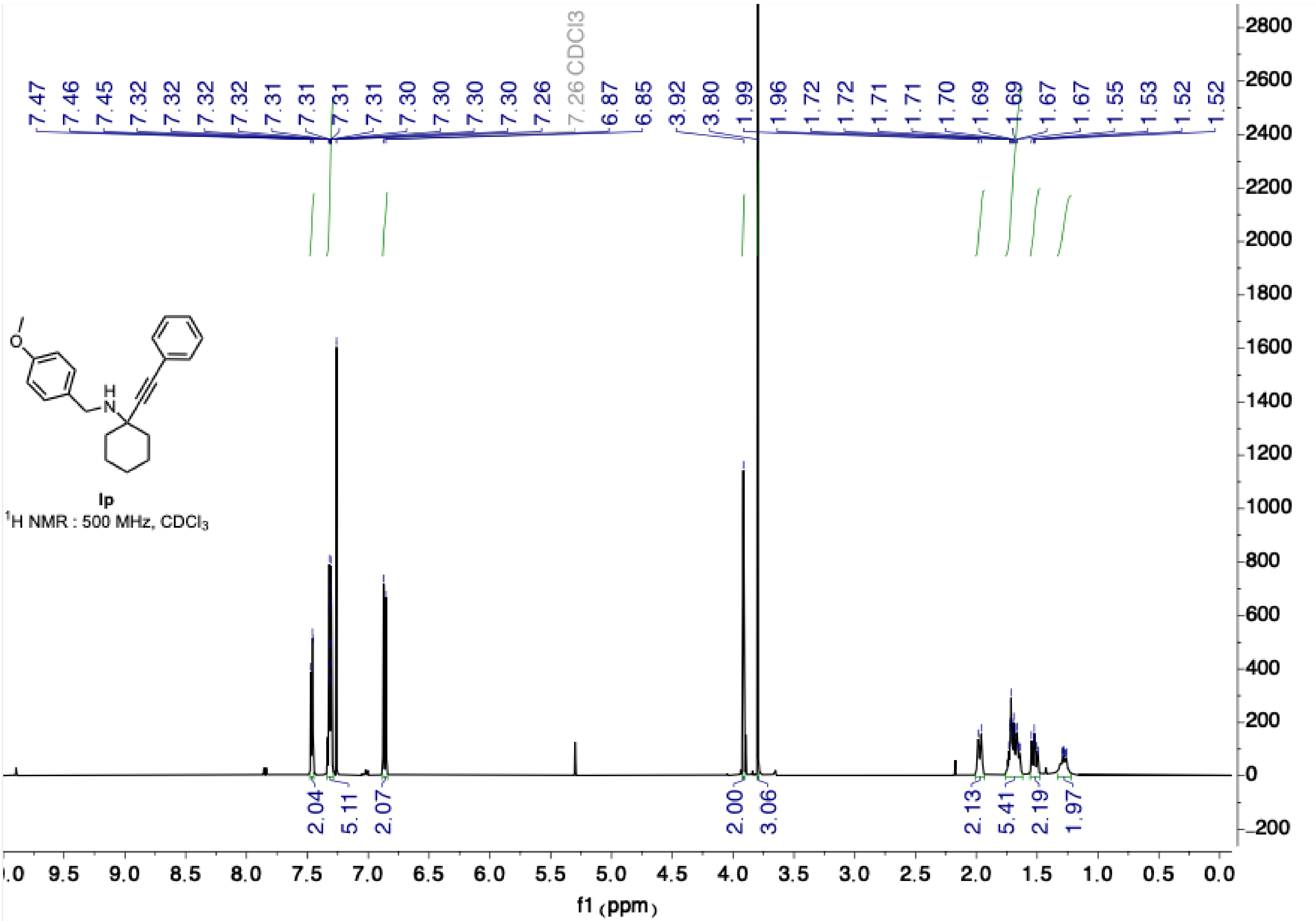

7.47 7.46 7.45 7.32 7.32 7.32 7.32 7.31 7.31 7.31 7.31 7.30 7.30 7.30 7.30 7.26 7.26 CDCl3 6.87 6.85 3.92 3.80 1.99 1.96 1.72 1.72 1.71 1.71 1.70 1.69 1.69 1.67 1.67 1.55 1.53 1.52 1.52
Ip
^{1}H NMR : 500 MHz, CDCl$_3$
2.04 5.11 2.07 2.00 3.06 2.13 5.41 2.19 1.97
f1 (ppm)

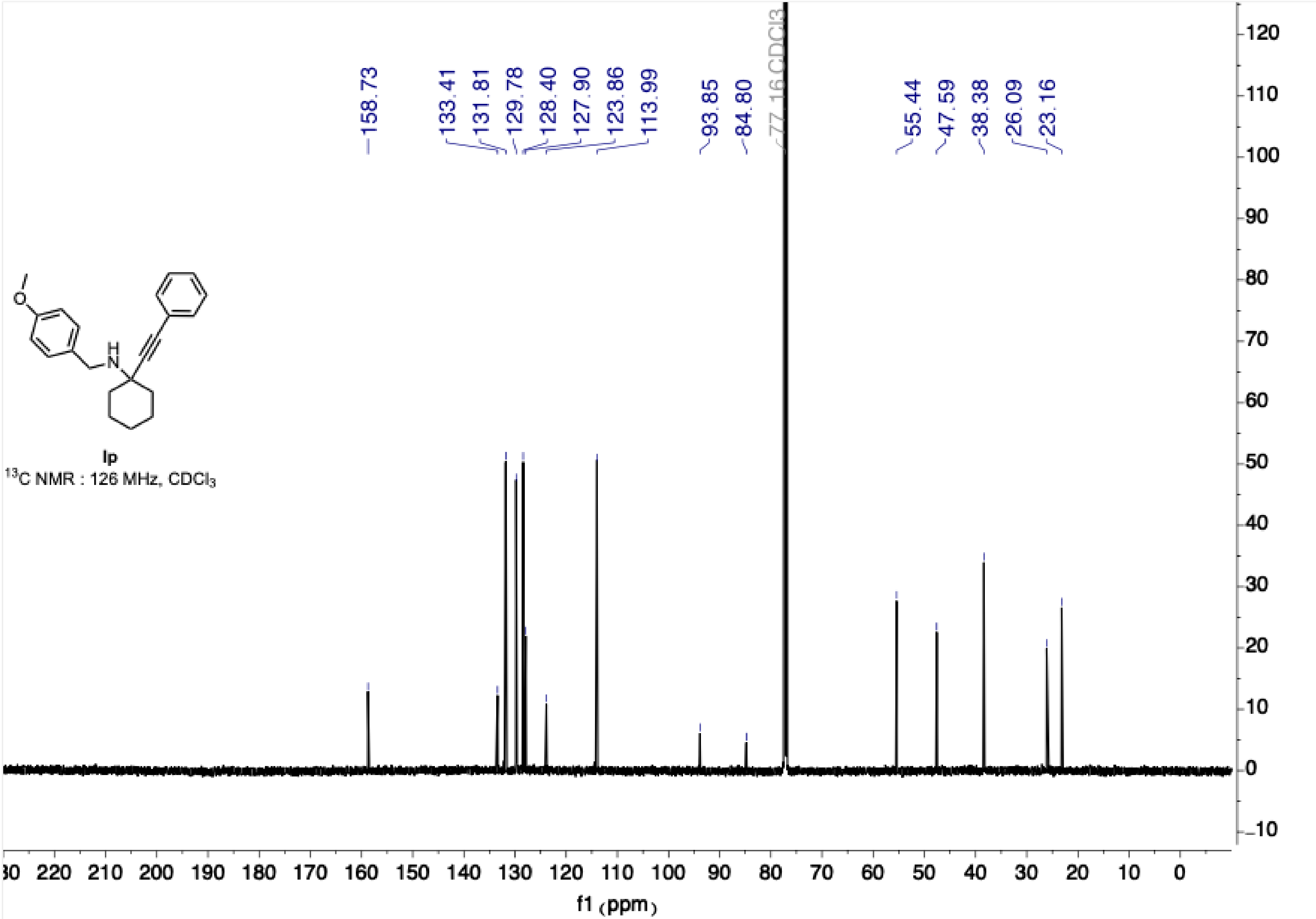

Ip

^{13}C NMR : 126 MHz, $CDCl_3$

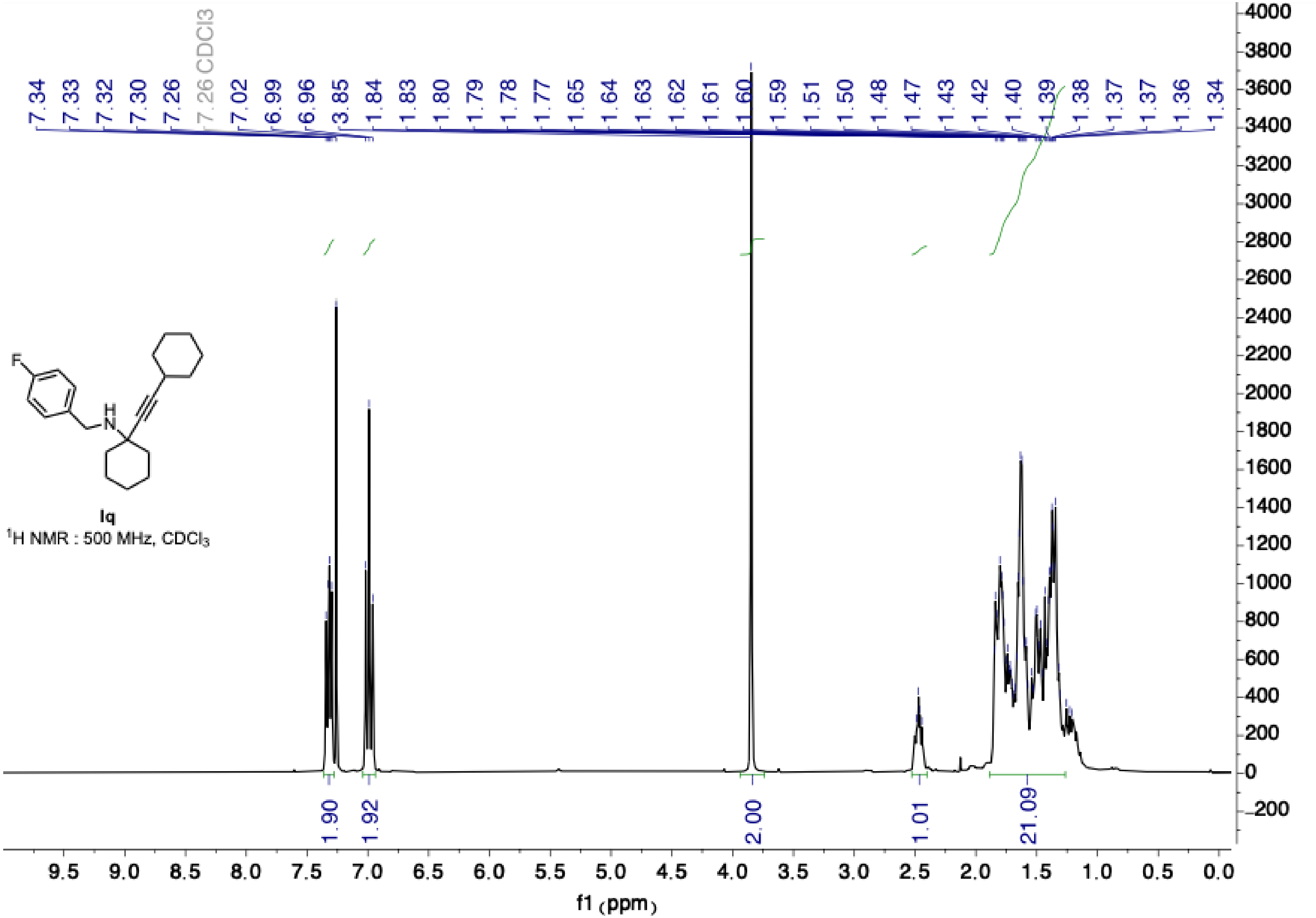

Iq

^{1}H NMR : 500 MHz, $CDCl_3$

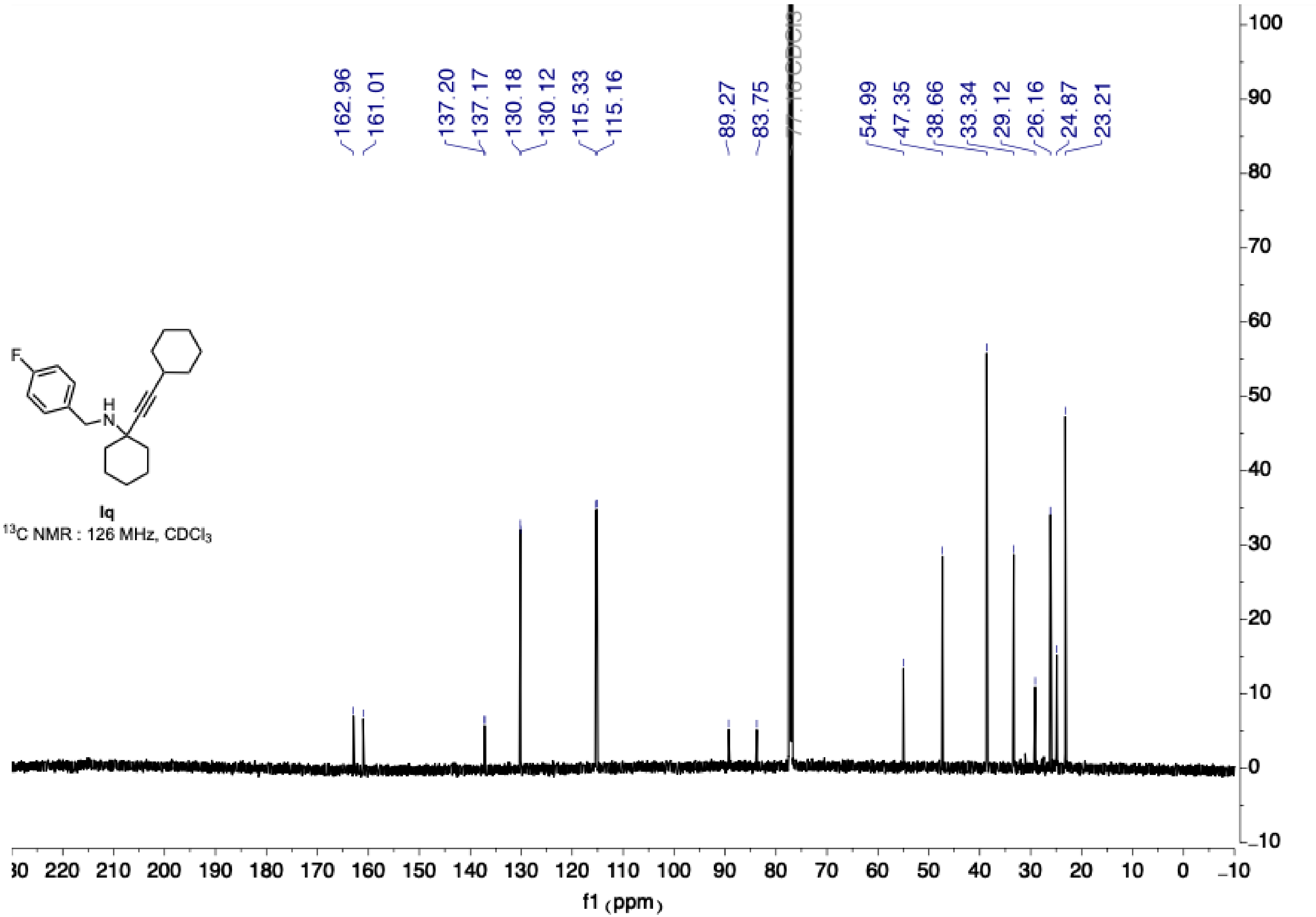
162.96
161.01
137.20
137.17
130.18
130.12
115.33
115.16
89.27
83.75
77.16 CDCl3
54.99
47.35
38.66
33.34
29.12
26.16
24.87
23.21
F
H
N
Iq
^{13}C NMR : 126 MHz, CDCl$_3$
f1 (ppm)

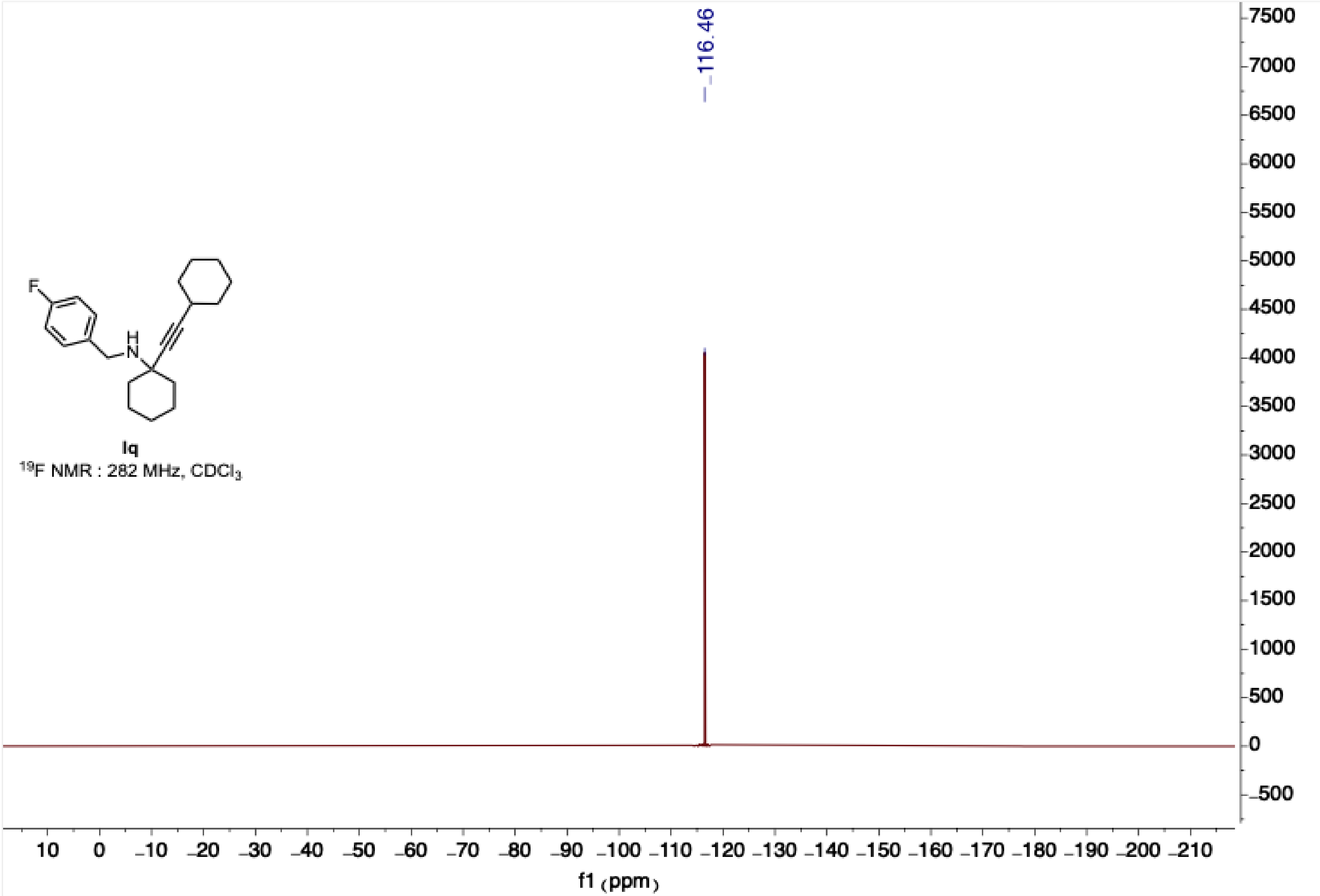

–116.46
F
H
N
Iq
^{19}F NMR : 282 MHz, CDCl$_3$
7500
7000
6500
6000
5500
5000
4500
4000
3500
3000
2500
2000
1500
1000
500
0
–500
10 0 –10 –20 –30 –40 –50 –60 –70 –80 –90 –100 –110 –120 –130 –140 –150 –160 –170 –180 –190 –200 –210
f1 (ppm)

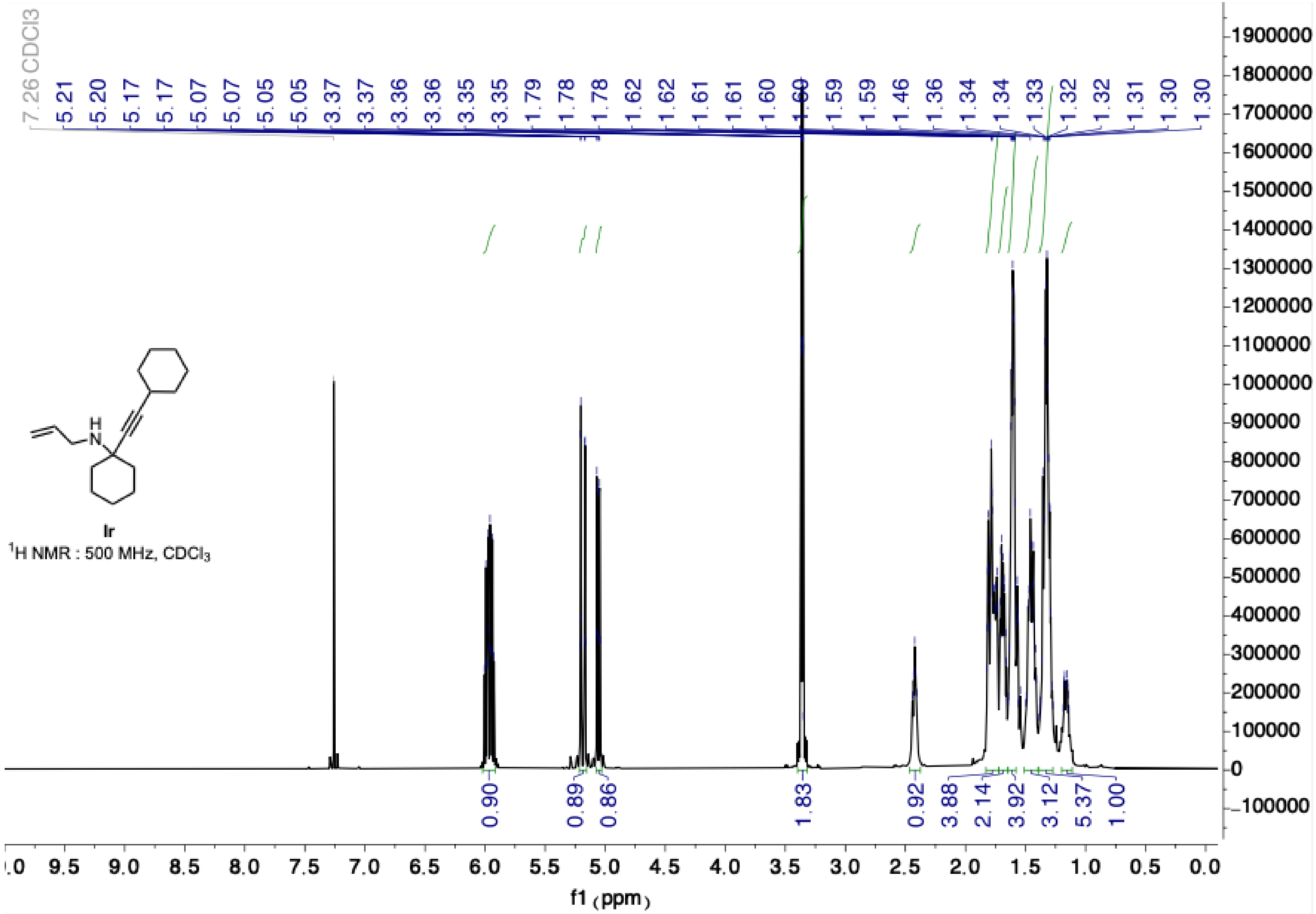
7.26 CDCl3
5.21 5.20 5.17 5.17 5.07 5.07 5.05 5.05 3.37 3.37 3.36 3.36 3.35 3.35 1.79 1.78 1.78 1.62 1.62 1.61 1.61 1.60 1.60 1.59 1.59 1.46 1.36 1.34 1.34 1.33 1.32 1.32 1.31 1.30 1.30
0.90 0.89 0.86 1.83 0.92 3.88 2.14 3.92 3.12 5.37 1.00
Ir
1H NMR : 500 MHz, CDCl3
f1 (ppm)

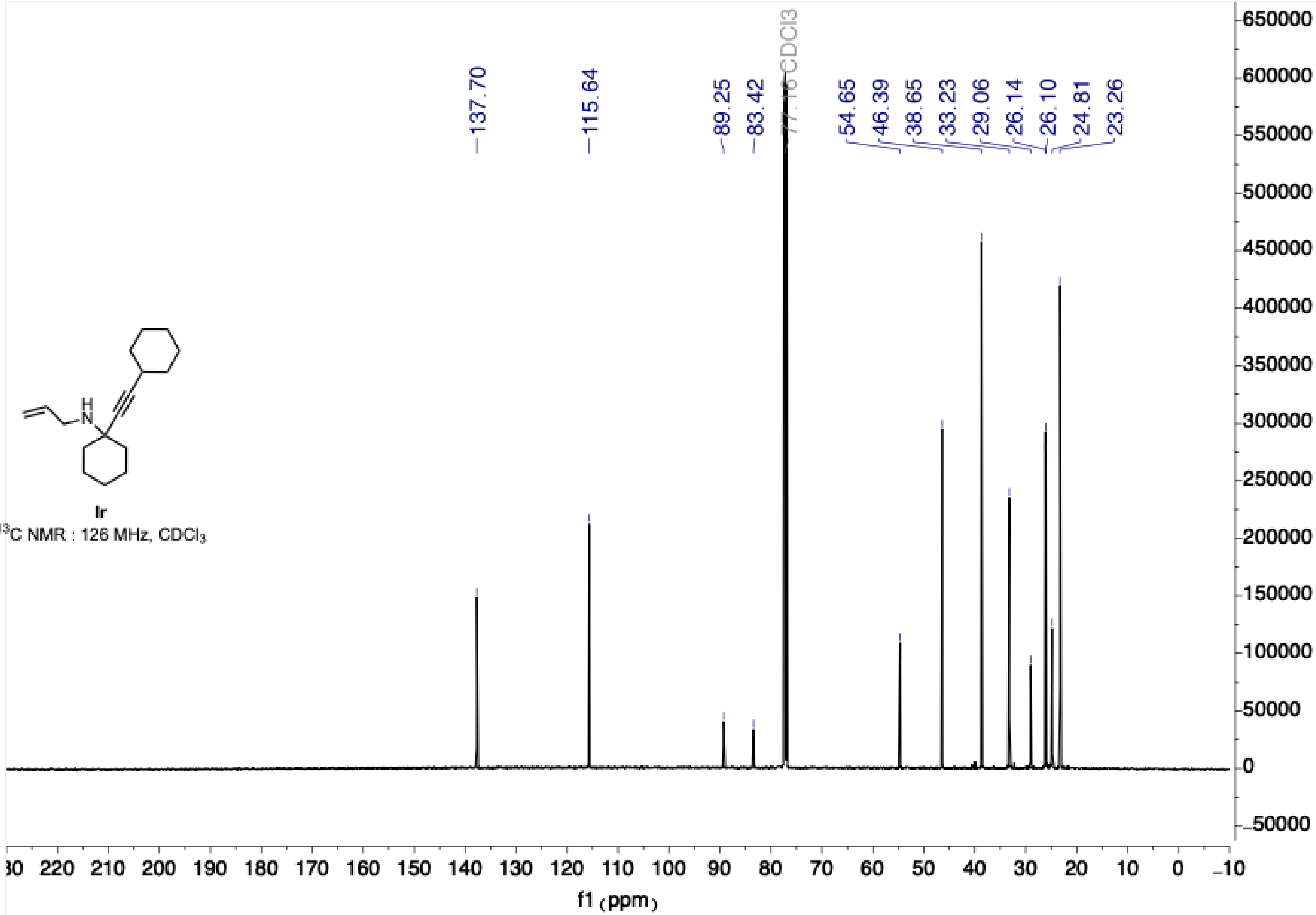

Ir

^{13}C NMR : 126 MHz, $CDCl_3$

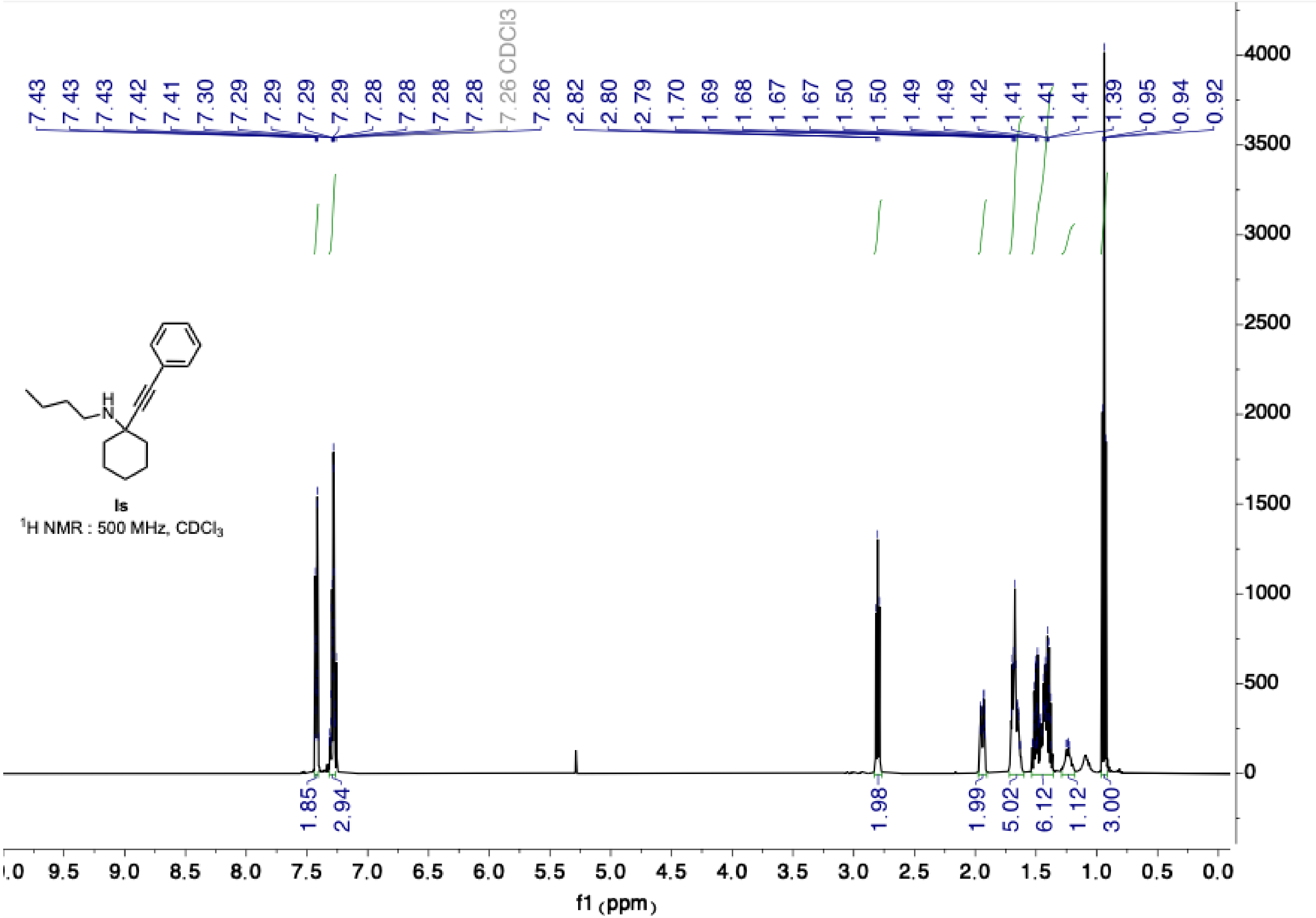

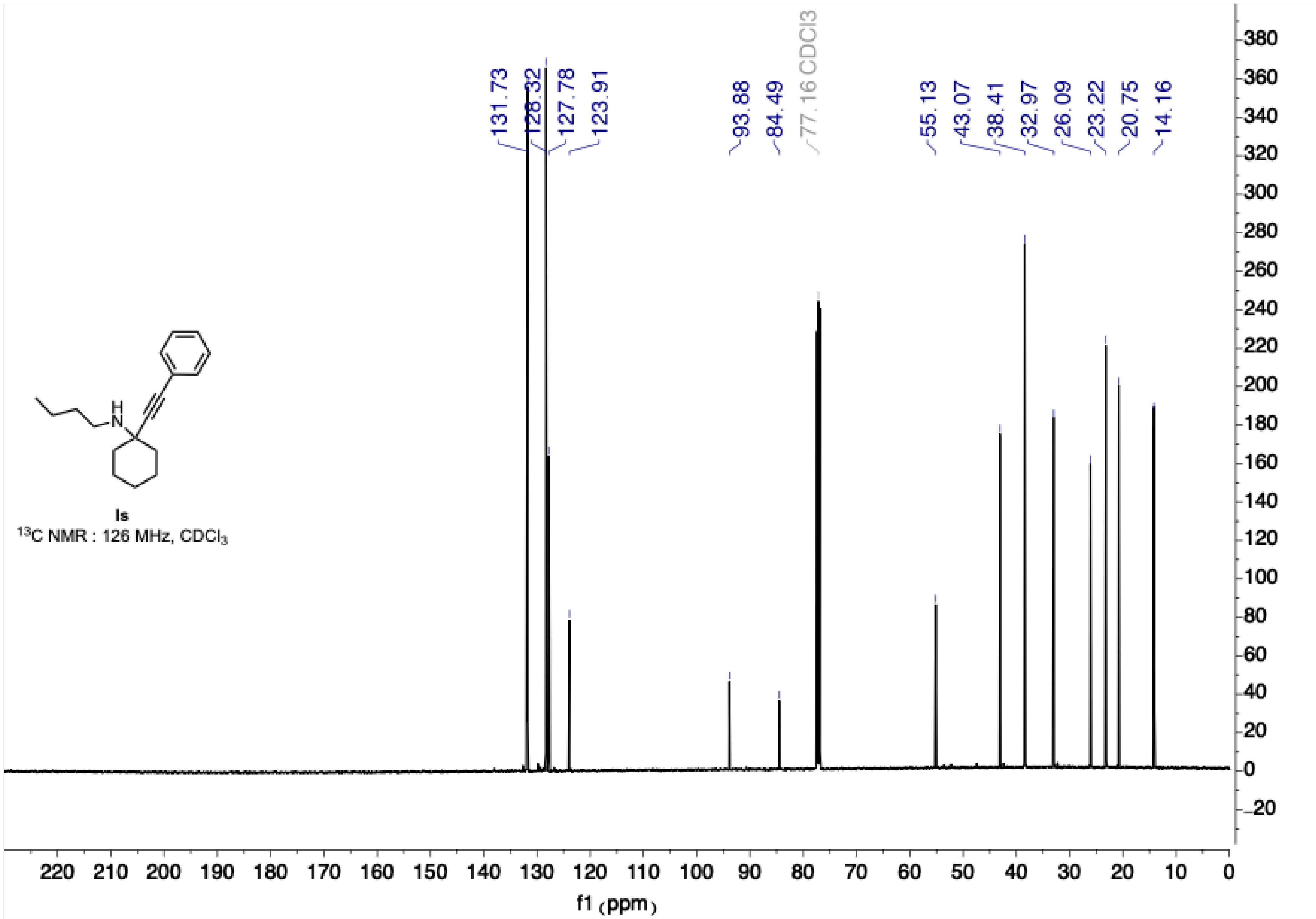
131.73
128.32
127.78
123.91
93.88
84.49
77.16 CDCl3
55.13
43.07
38.41
32.97
26.09
23.22
20.75
14.16
Is
13C NMR : 126 MHz, CDCl3
f1 (ppm)

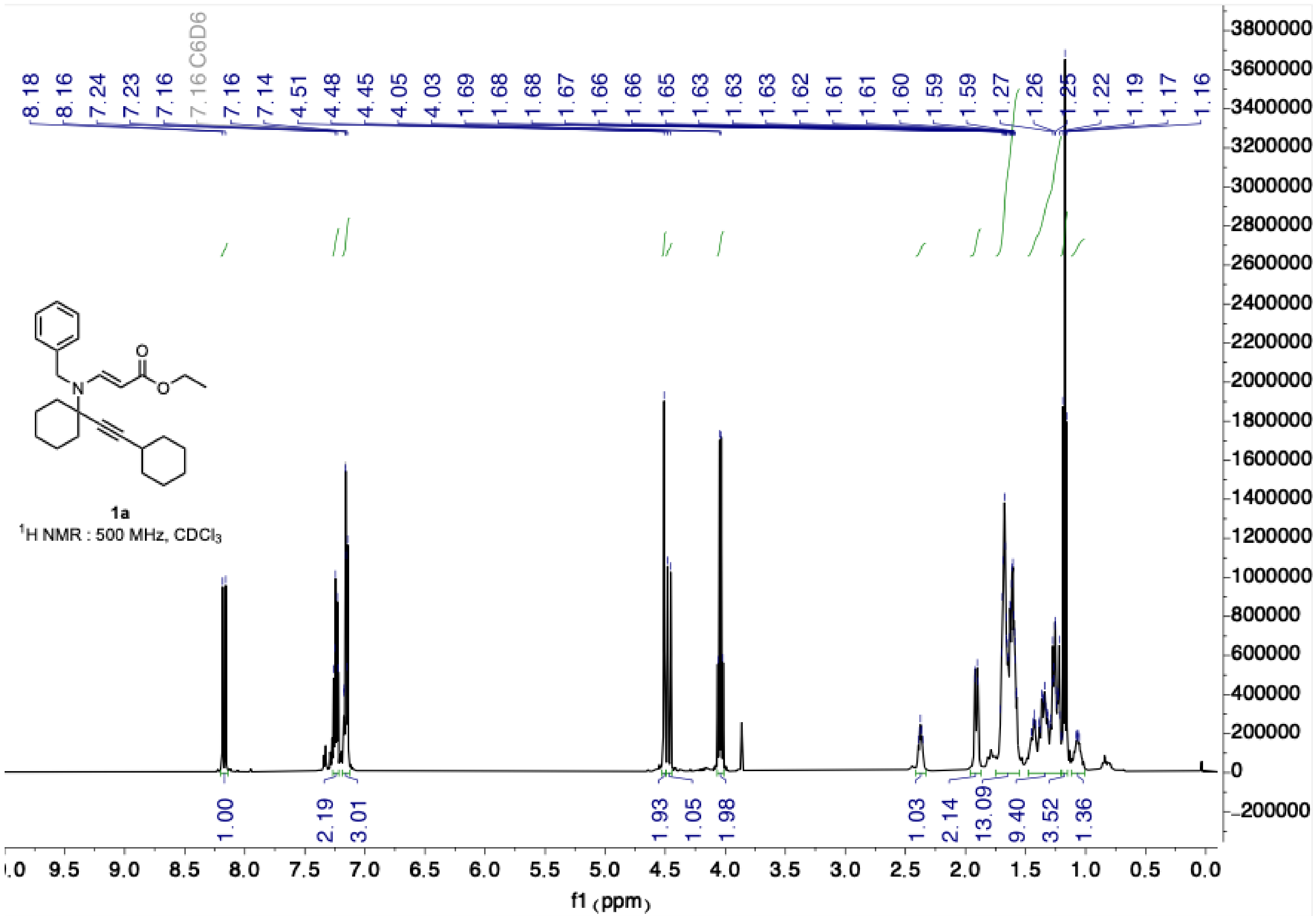
1a
¹H NMR : 500 MHz, CDCl3
f1 (ppm)

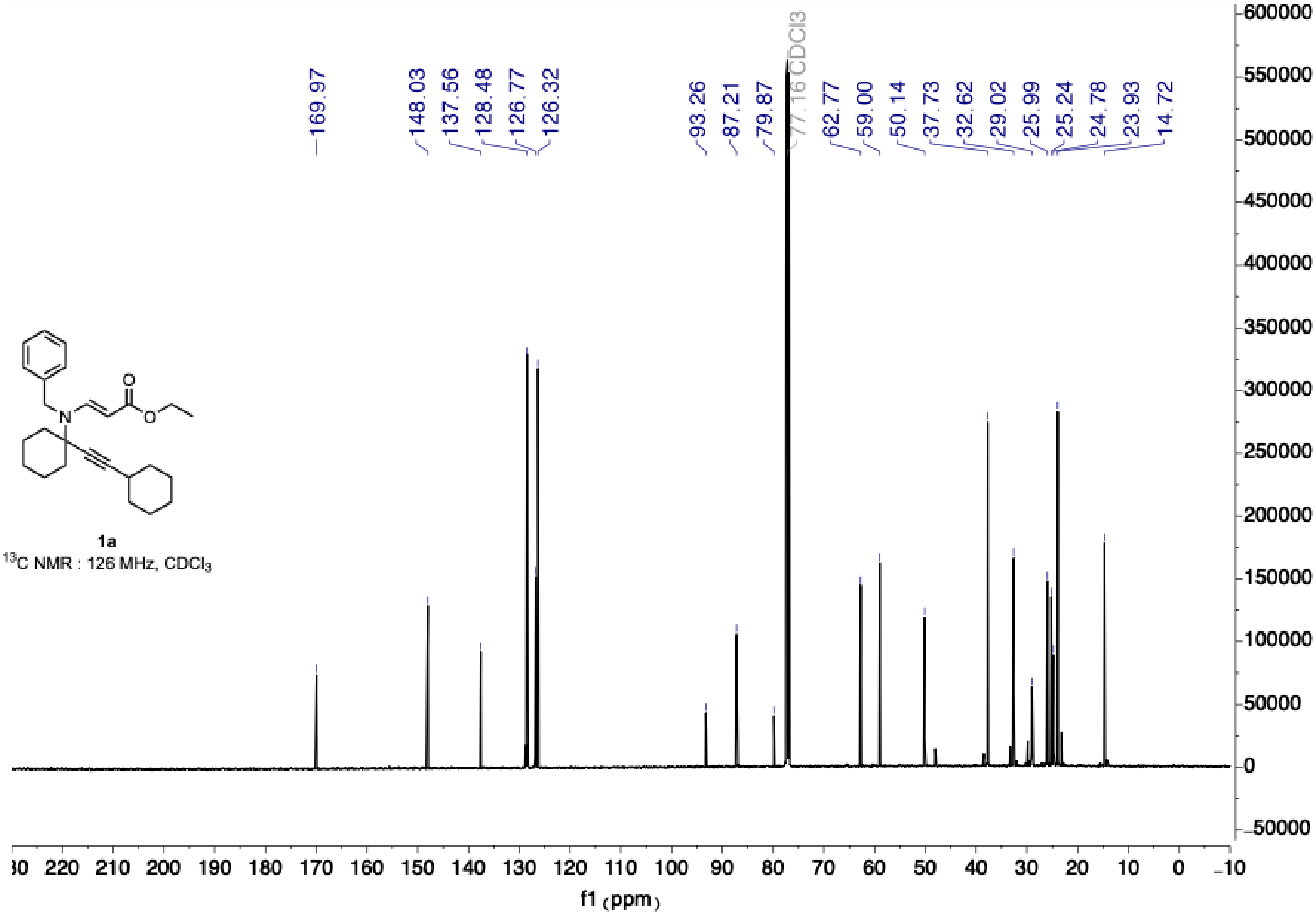

1a

^{13}C NMR : 126 MHz, $CDCl_3$

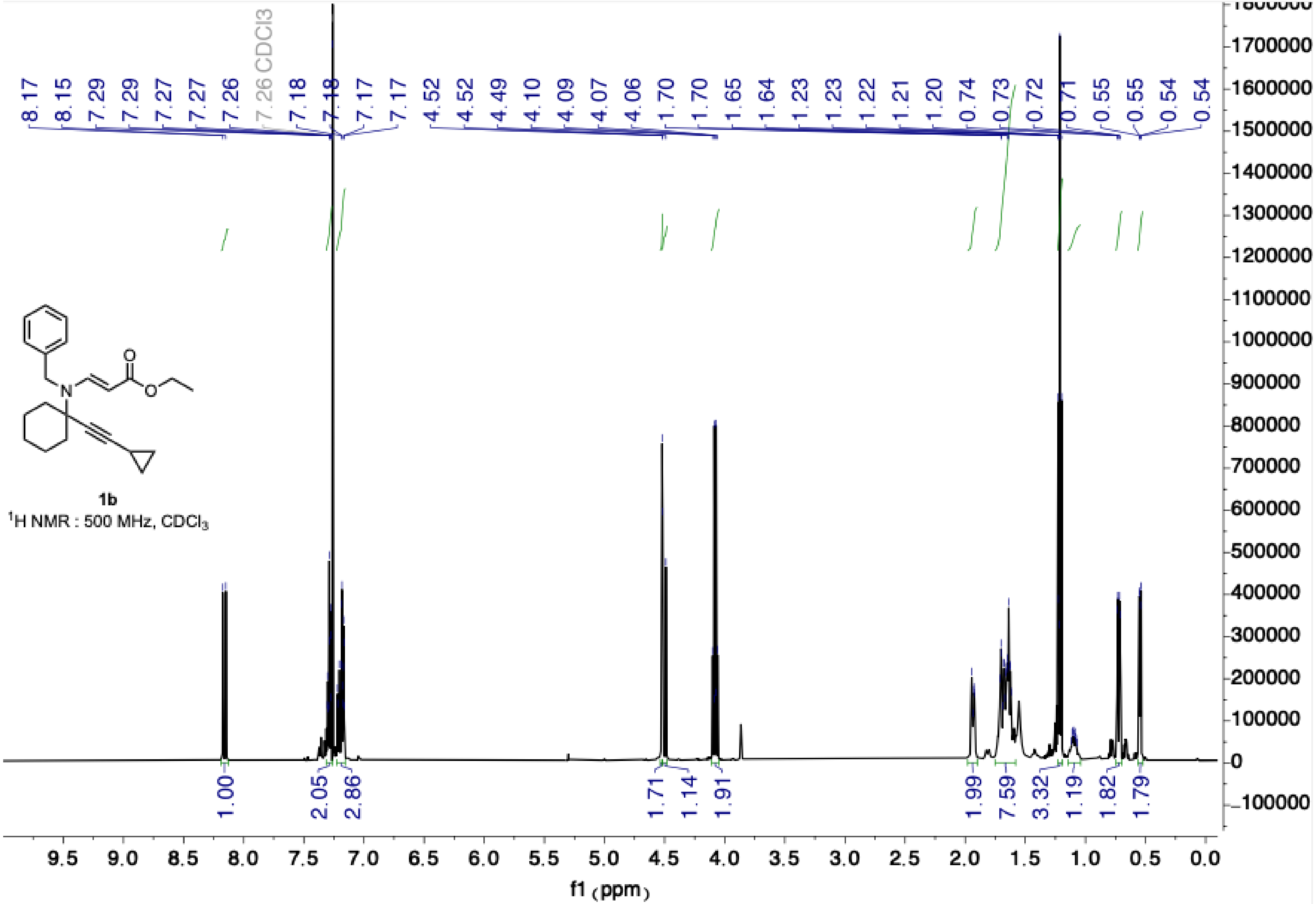

1b

^1H NMR : 500 MHz, $CDCl_3$

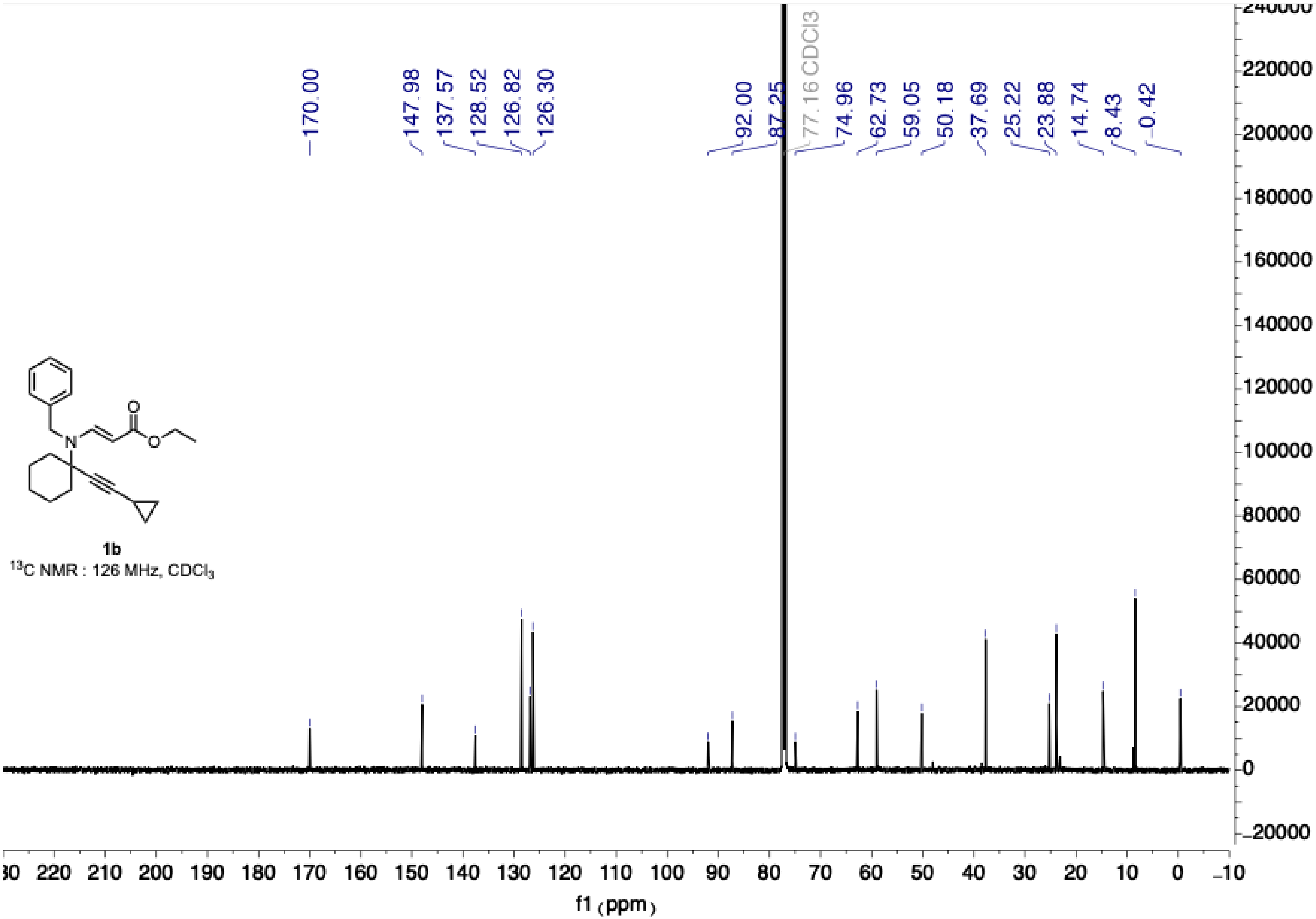

1b
^{13}C NMR : 126 MHz, $CDCl_3$

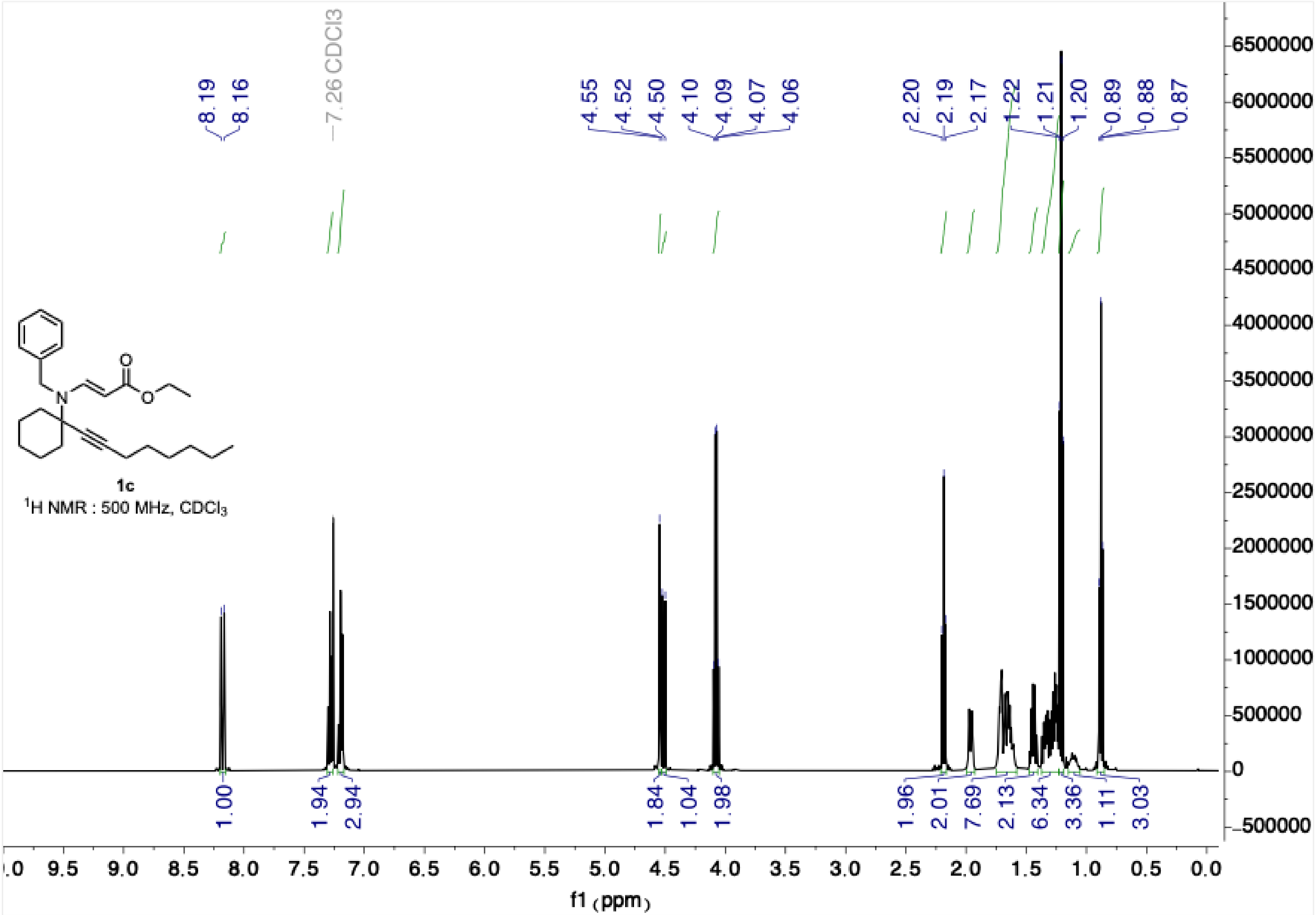
8.19
8.16
7.26 CDCl3
4.55
4.52
4.50
4.10
4.09
4.07
4.06
2.20
2.19
2.17
1.22
1.21
1.20
0.89
0.88
0.87
1.00
1.94
2.94
1.84
1.04
1.98
1.96
2.01
7.69
2.13
6.34
3.36
1.11
3.03
N
O
O
1c
1H NMR : 500 MHz, CDCl3
6500000
6000000
5500000
5000000
4500000
4000000
3500000
3000000
2500000
2000000
1500000
1000000
500000
0
-500000
9.5
9.0
8.5
8.0
7.5
7.0
6.5
6.0
5.5
5.0
4.5
4.0
3.5
3.0
2.5
2.0
1.5
1.0
0.5
0.0
f1 (ppm)

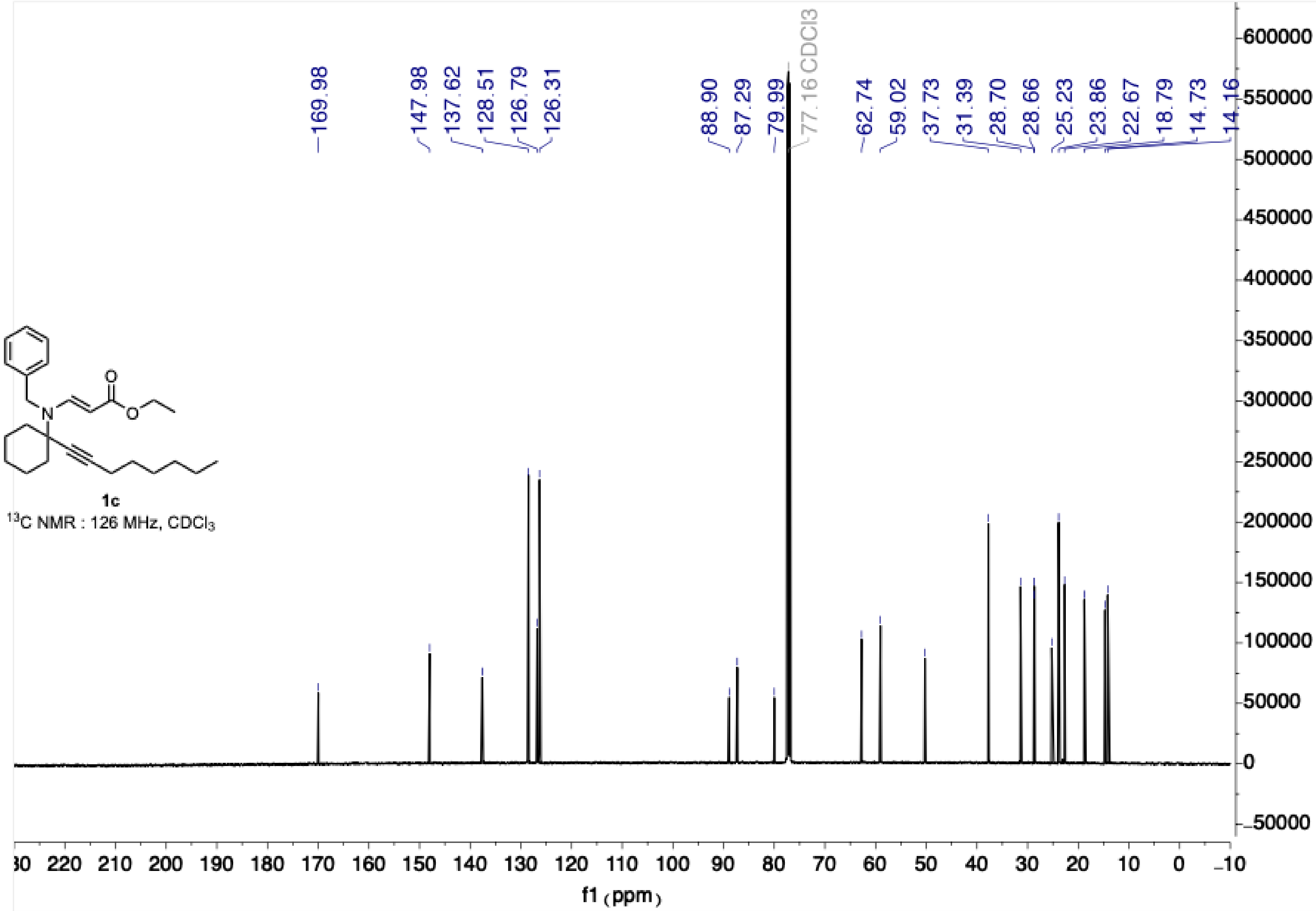
169.98
147.98
137.62
128.51
126.79
126.31
88.90
87.29
79.99
77.16 CDCl3
62.74
59.02
37.73
31.39
28.70
28.66
25.23
23.86
22.67
18.79
14.73
14.16
1c
13C NMR : 126 MHz, CDCl3
f1 (ppm)

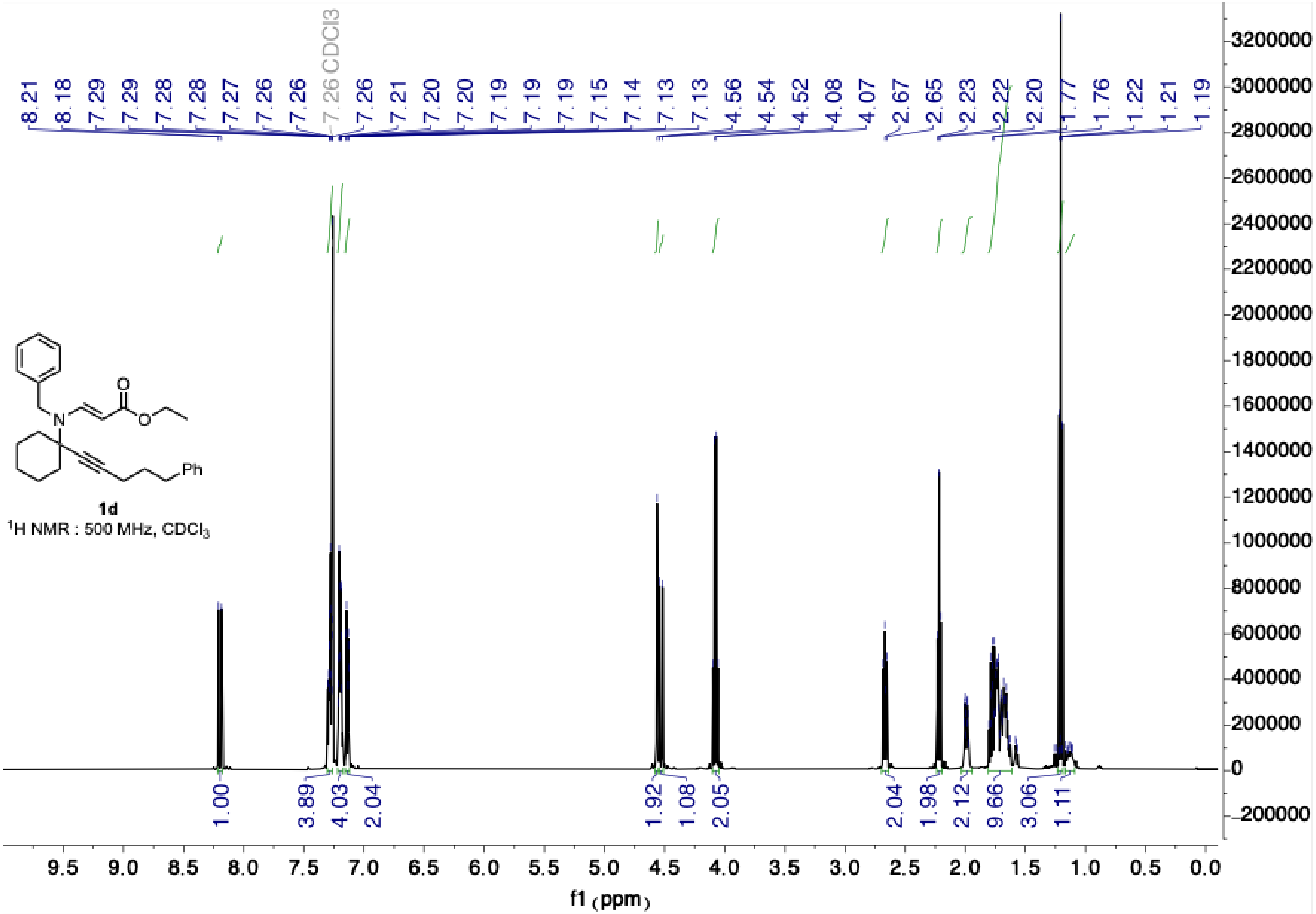
1d
1H NMR : 500 MHz, CDCl3
f1 (ppm)

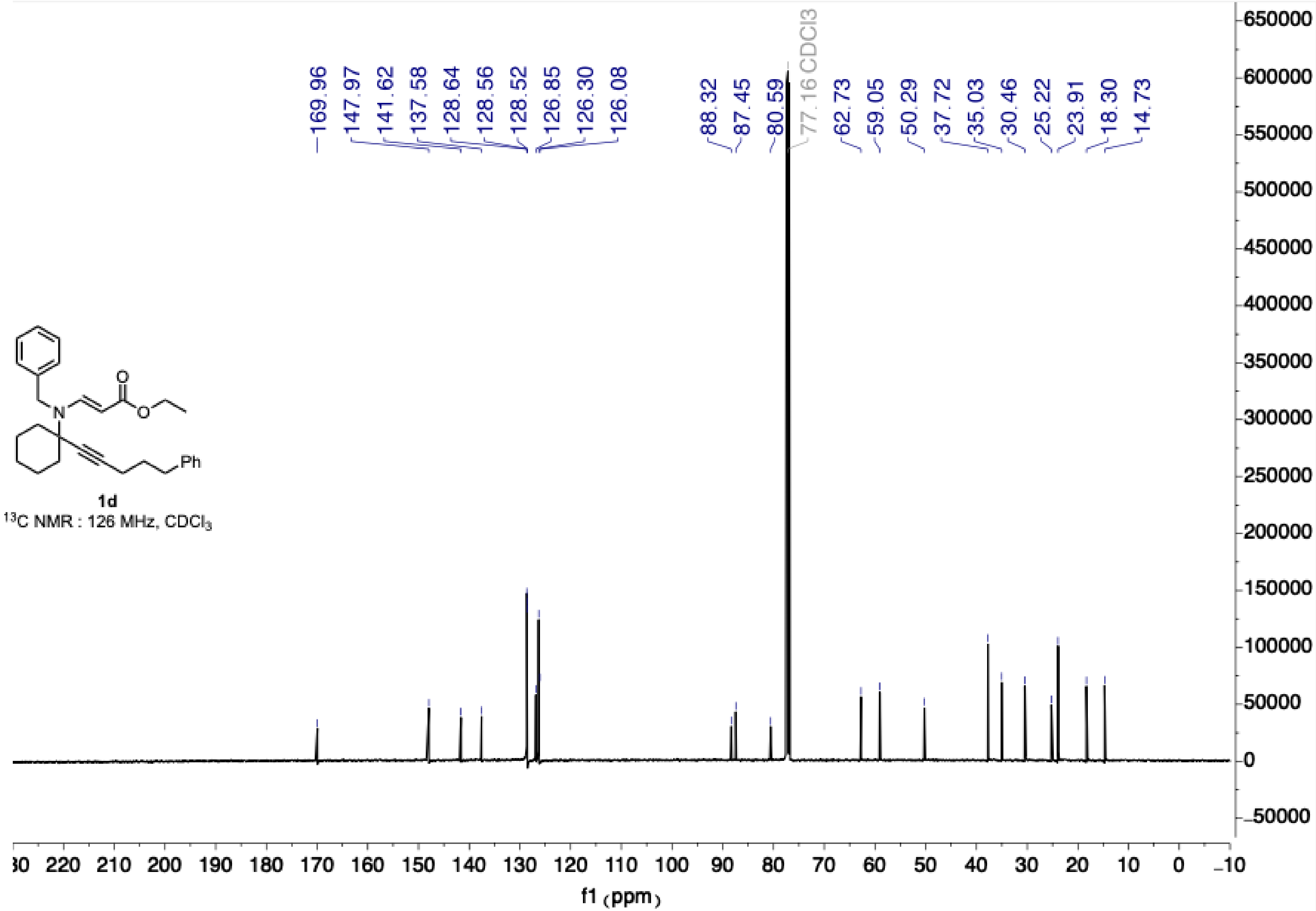
169.96
147.97
141.62
137.58
128.64
128.56
128.52
126.85
126.30
126.08
88.32
87.45
80.59
77.16 CDCl3
62.73
59.05
50.29
37.72
35.03
30.46
25.22
23.91
18.30
14.73
O
N
O
Ph
1d
^{13}C NMR : 126 MHz, $CDCl_3$
f1 (ppm)

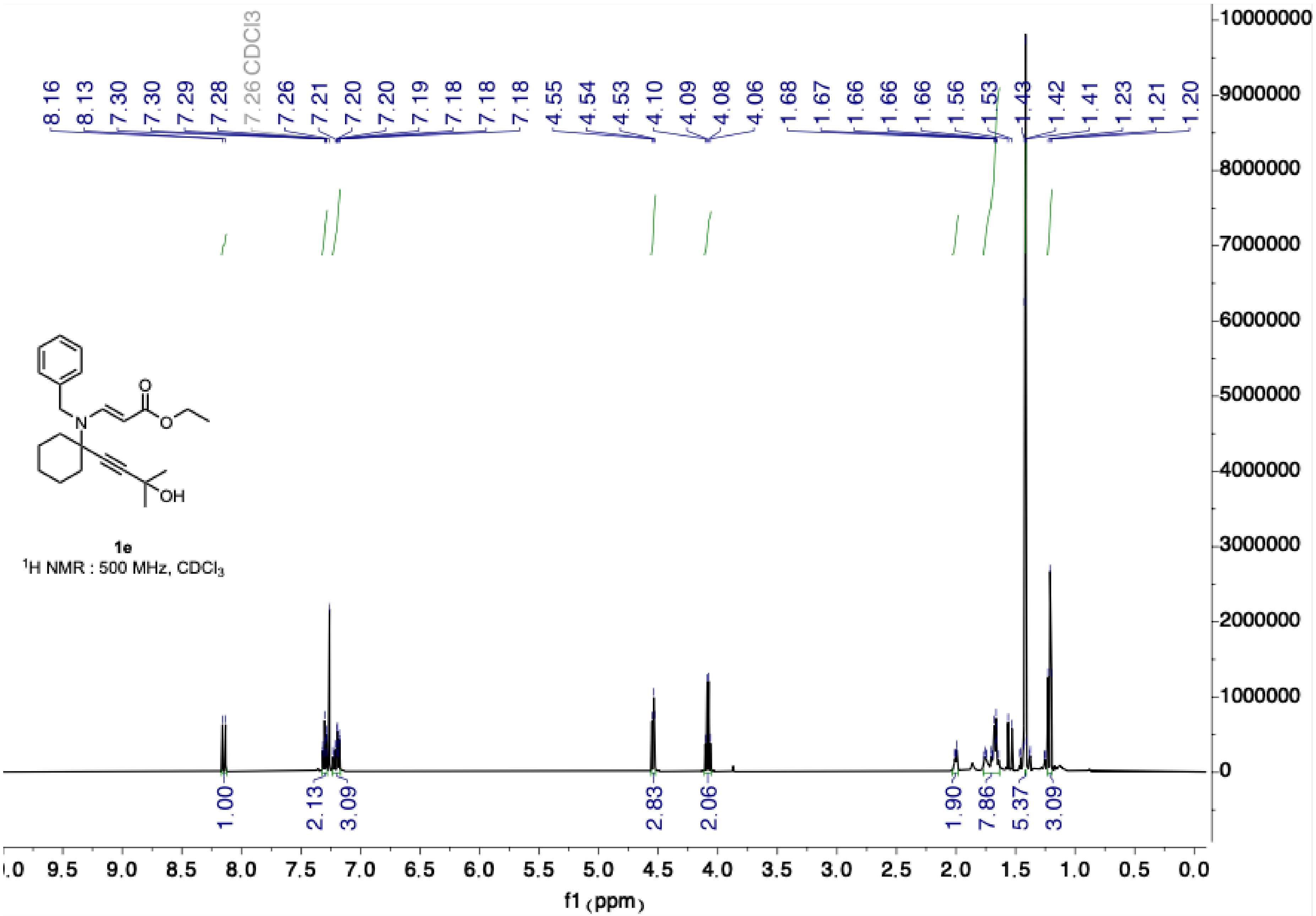
8.16 8.13 7.30 7.30 7.29 7.28 7.26 CDCl3 7.26 7.21 7.20 7.20 7.19 7.18 7.18 7.18 4.55 4.54 4.53 4.10 4.09 4.08 4.06 1.68 1.67 1.66 1.66 1.66 1.56 1.53 1.43 1.42 1.41 1.23 1.21 1.20
1.00 2.13 3.09 2.83 2.06 1.90 7.86 5.37 3.09
N
O
O
OH
1e
1H NMR : 500 MHz, CDCl3
10000000 9000000 8000000 7000000 6000000 5000000 4000000 3000000 2000000 1000000 0
9.5 9.0 8.5 8.0 7.5 7.0 6.5 6.0 5.5 5.0 4.5 4.0 3.5 3.0 2.5 2.0 1.5 1.0 0.5 0.0
f1 (ppm)

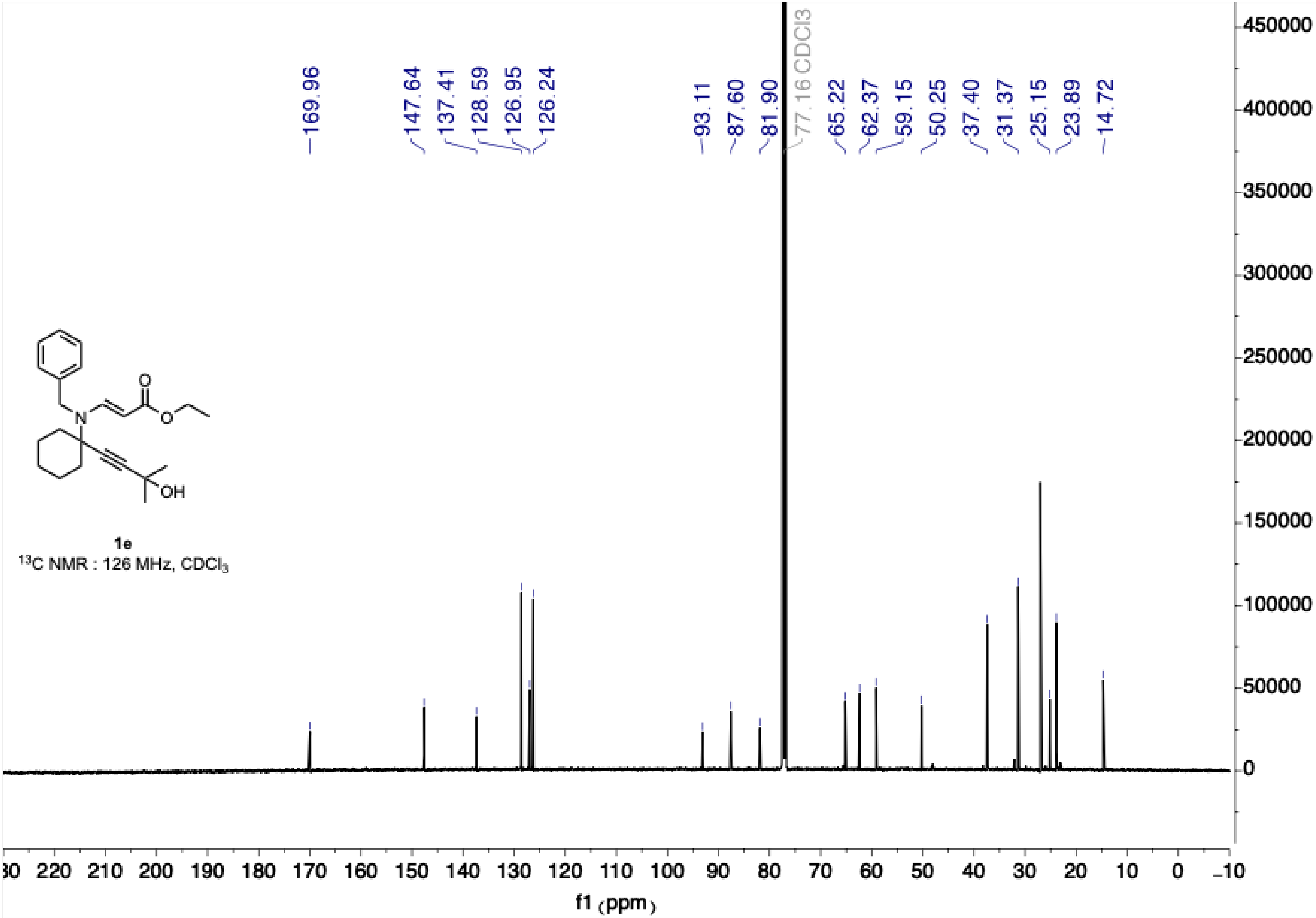
169.96
147.64
137.41
128.59
126.95
126.24
93.11
87.60
81.90
77.16 CDCl3
65.22
62.37
59.15
50.25
37.40
31.37
25.15
23.89
14.72
O
N
O
OH
1e
13C NMR : 126 MHz, CDCl3
450000
400000
350000
300000
250000
200000
150000
100000
50000
0
30 220 210 200 190 180 170 160 150 140 130 120 110 100 90 80 70 60 50 40 30 20 10 0 -10
f1 (ppm)

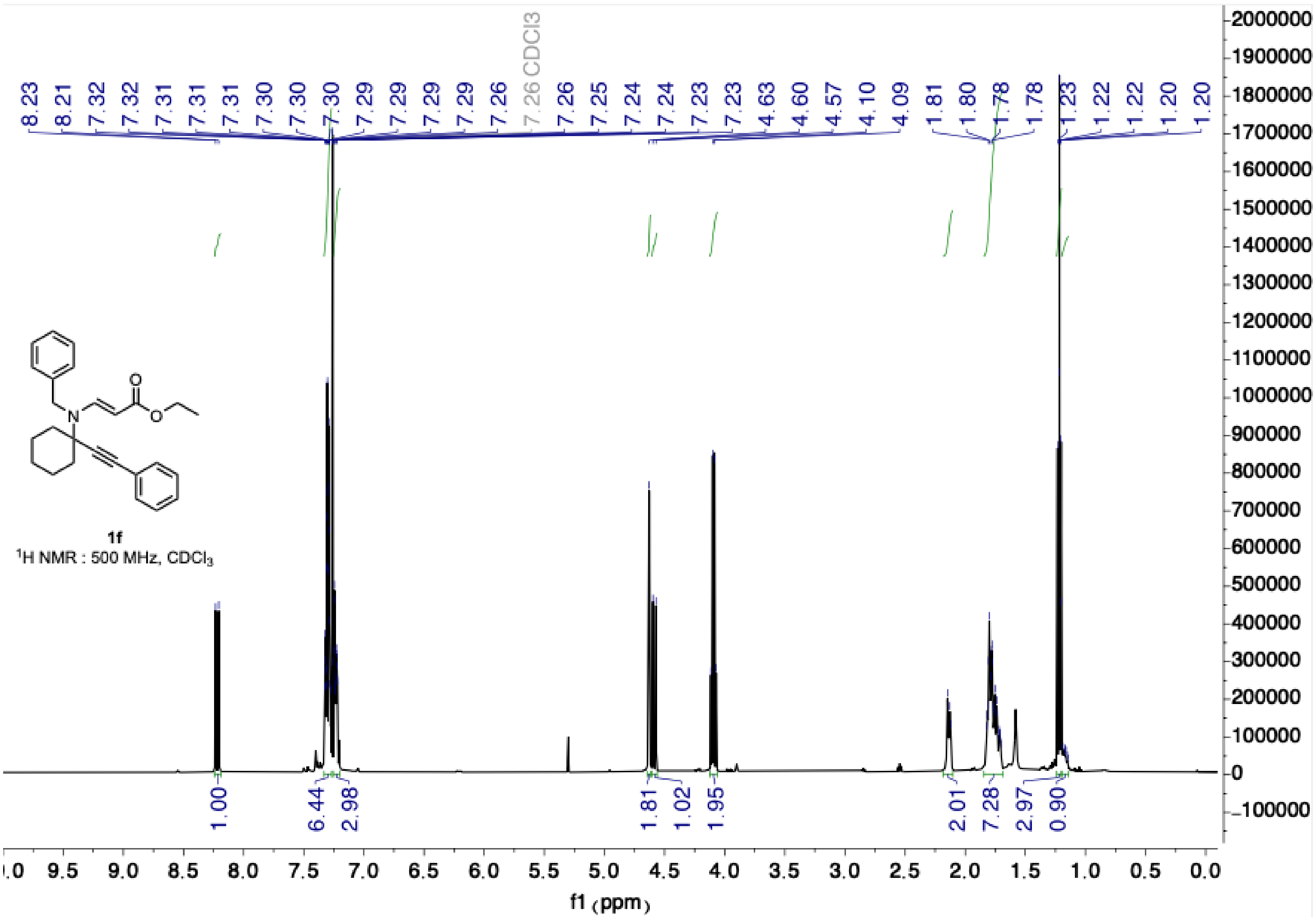

1f

^{1}H NMR : 500 MHz, $CDCl_3$

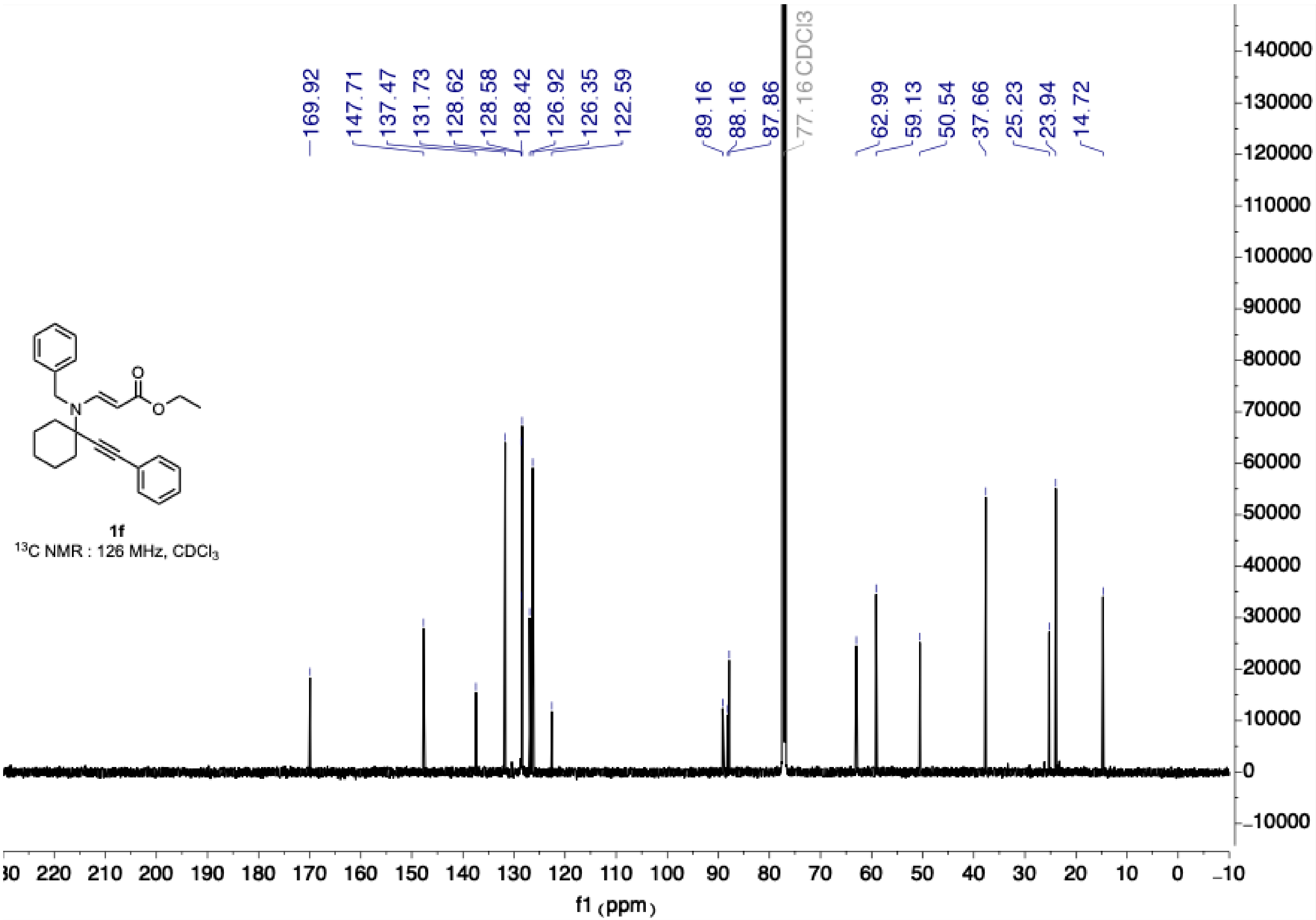
169.92
147.71
137.47
131.73
128.62
128.58
128.42
126.92
126.35
122.59
89.16
88.16
87.86
77.16 CDCl3
62.99
59.13
50.54
37.66
25.23
23.94
14.72
1f
^{13}C NMR : 126 MHz, $CDCl_3$
f1 (ppm)

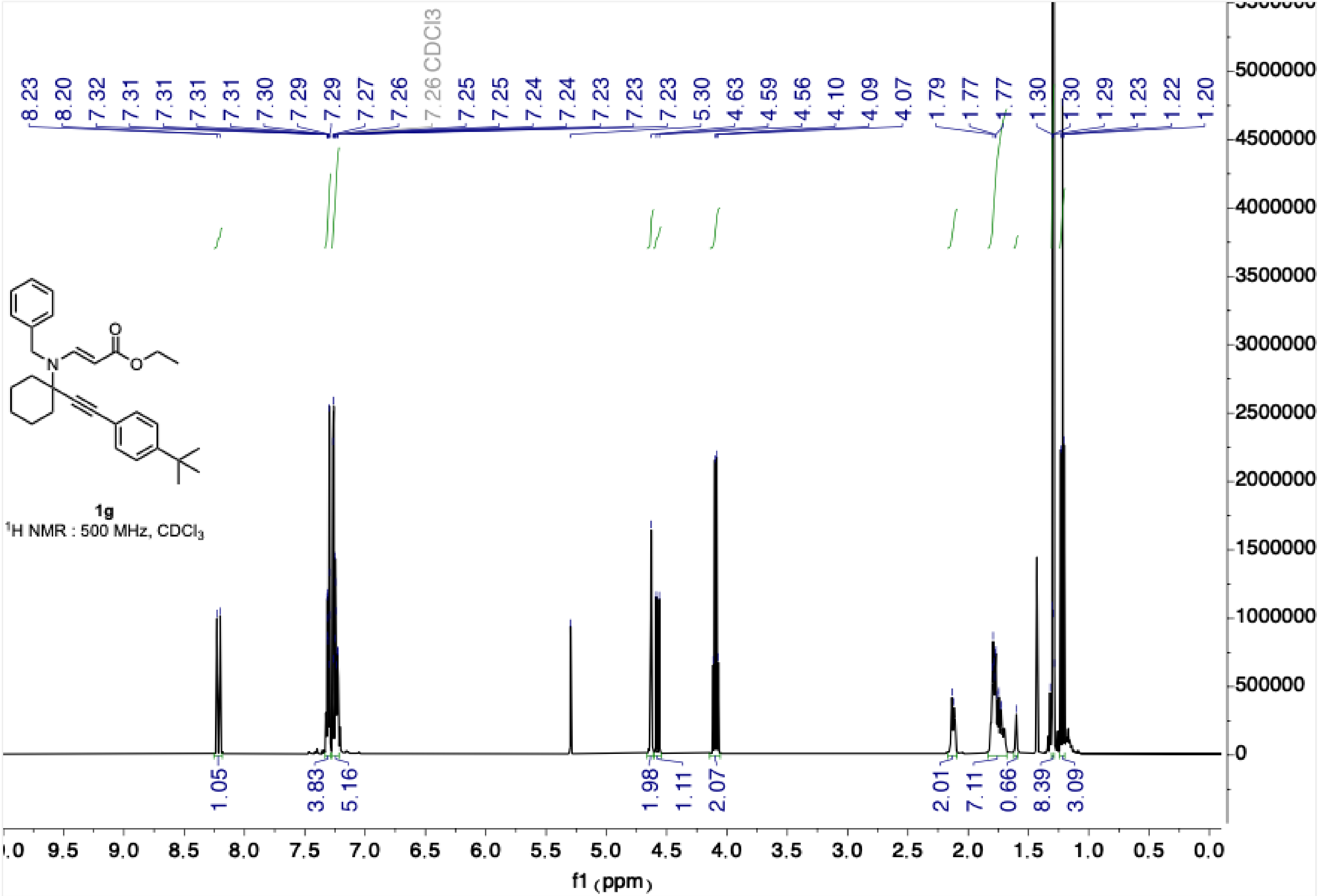

1g

^{1}H NMR : 500 MHz, $CDCl_3$

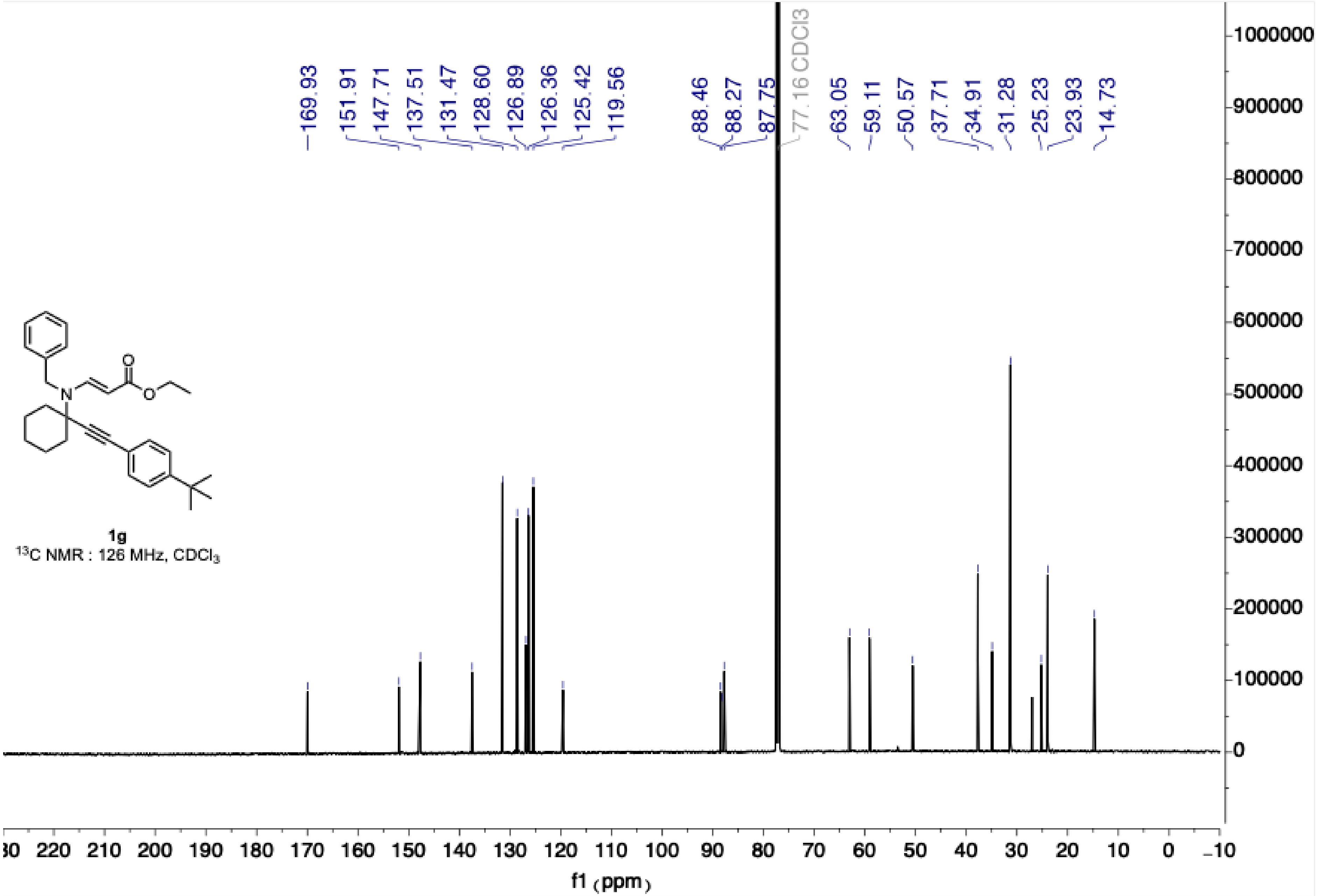
169.93
151.91
147.71
137.51
131.47
128.60
126.89
126.36
125.42
119.56
88.46
88.27
87.75
77.16 CDCl3
63.05
59.11
50.57
37.71
34.91
31.28
25.23
23.93
14.73
1g
13C NMR : 126 MHz, CDCl3
f1 (ppm)

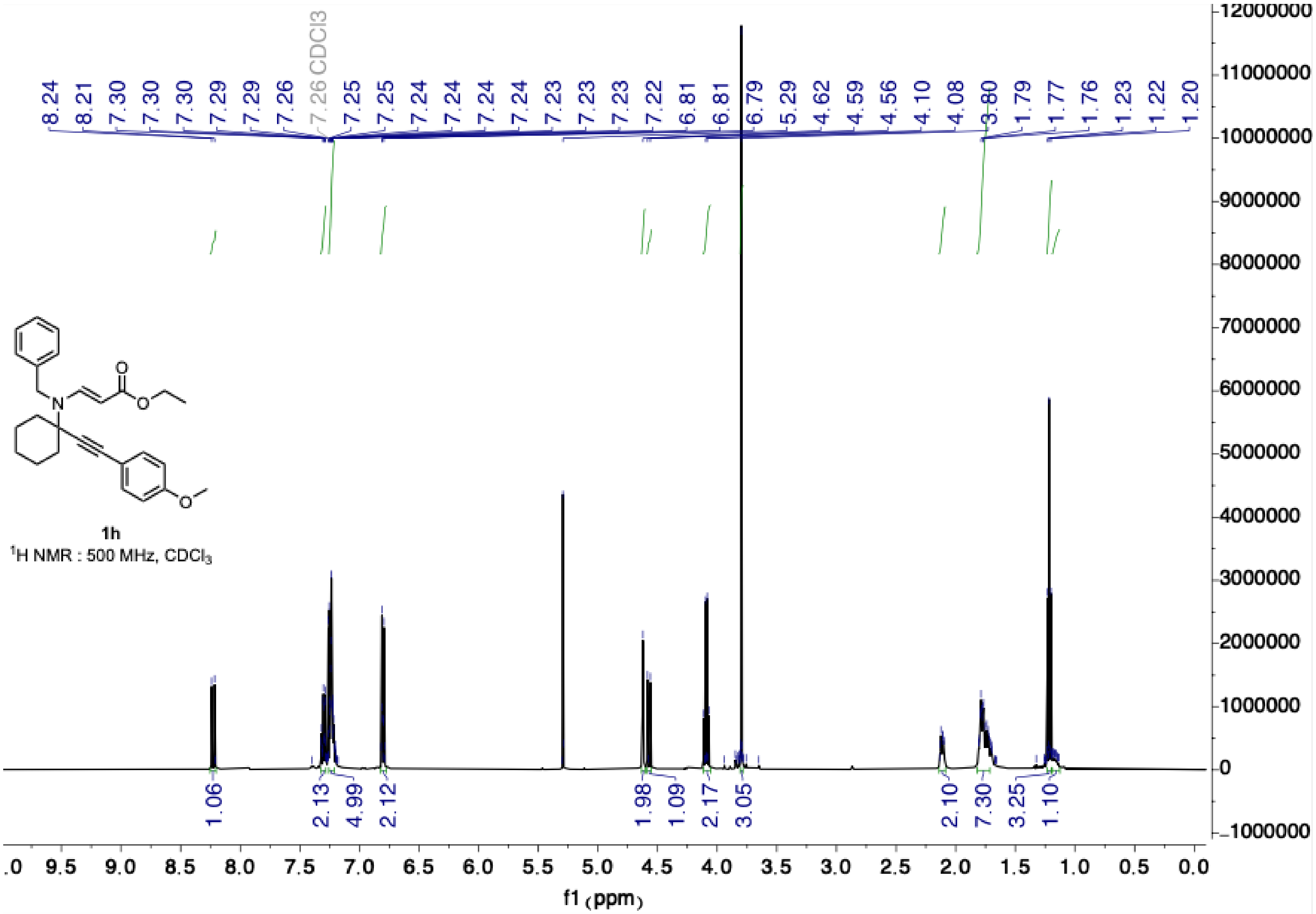

1h

^{1}H NMR : 500 MHz, $CDCl_3$

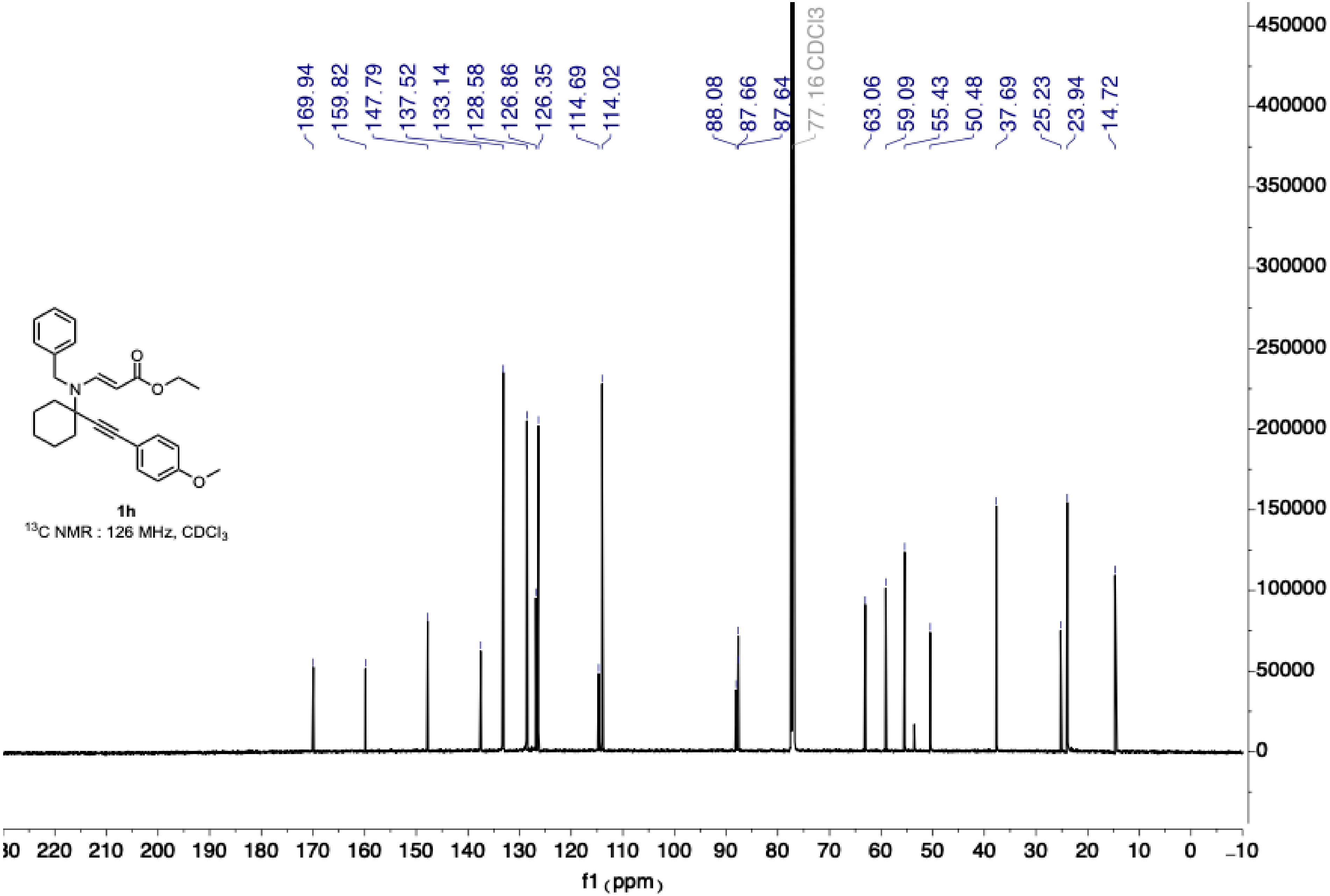

1h

^{13}C NMR : 126 MHz, $CDCl_3$

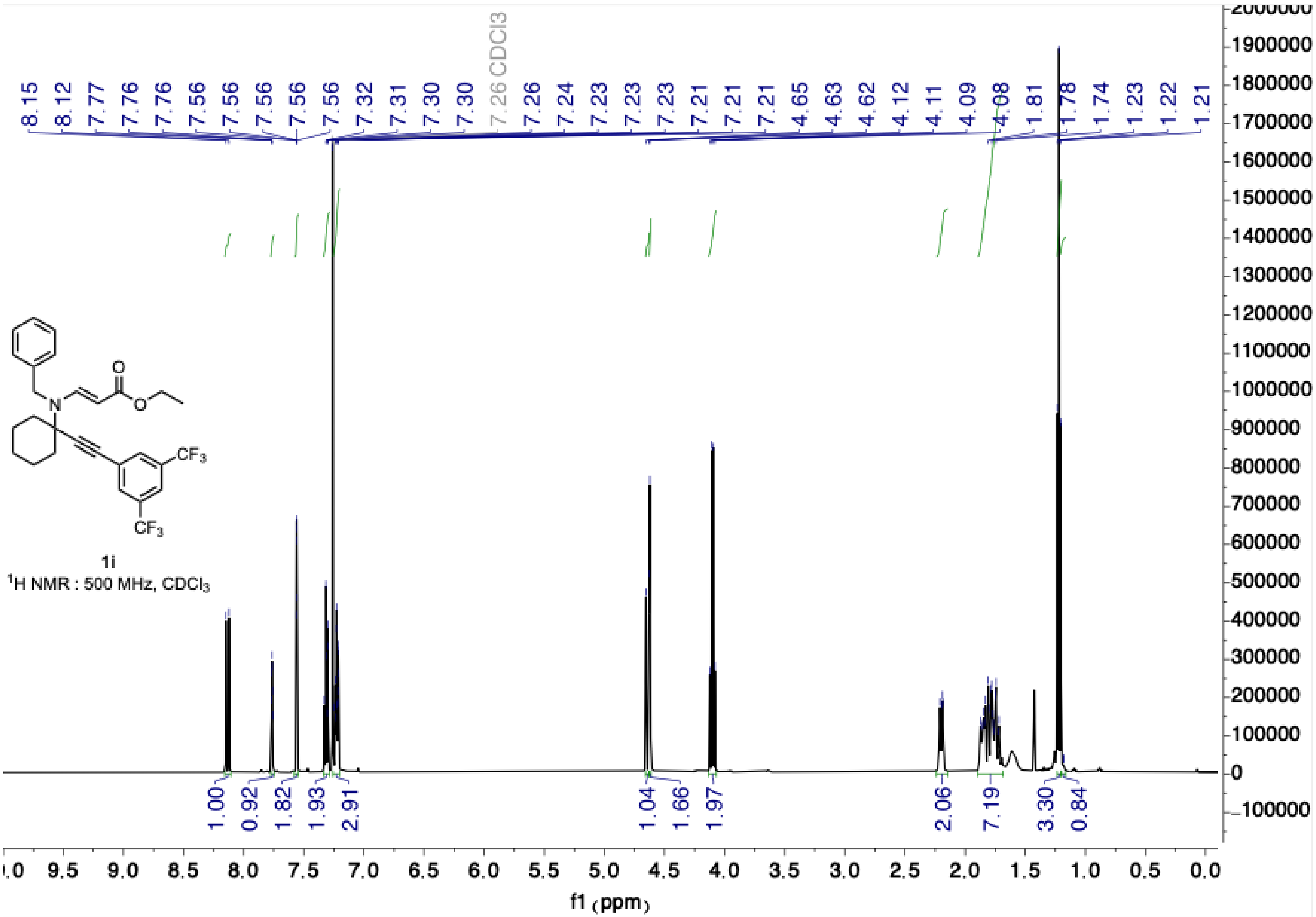

1i
1H NMR : 500 MHz, CDCl3
7.26 CDCl3
f1 (ppm)

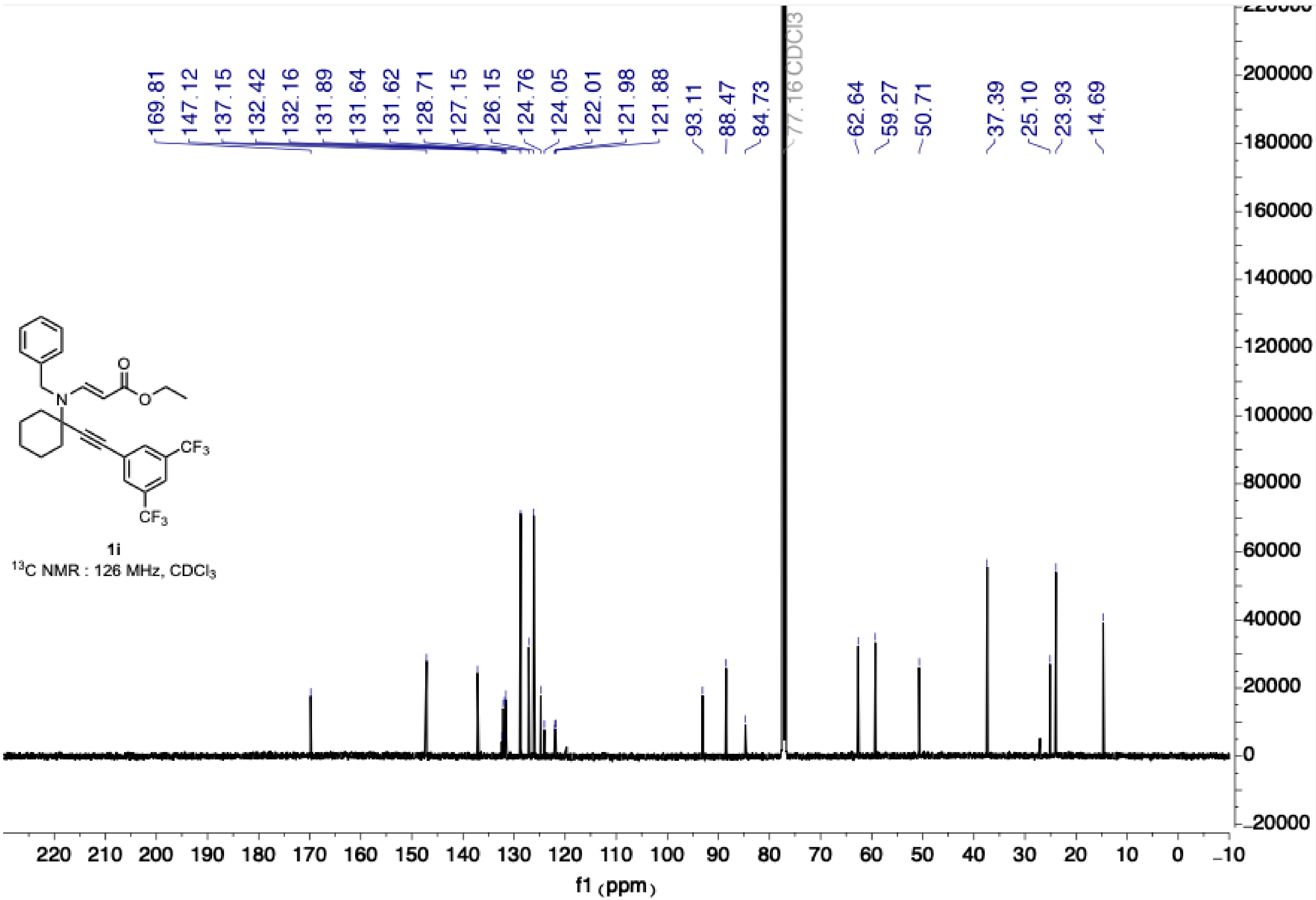

1i

^{13}C NMR : 126 MHz, $CDCl_3$

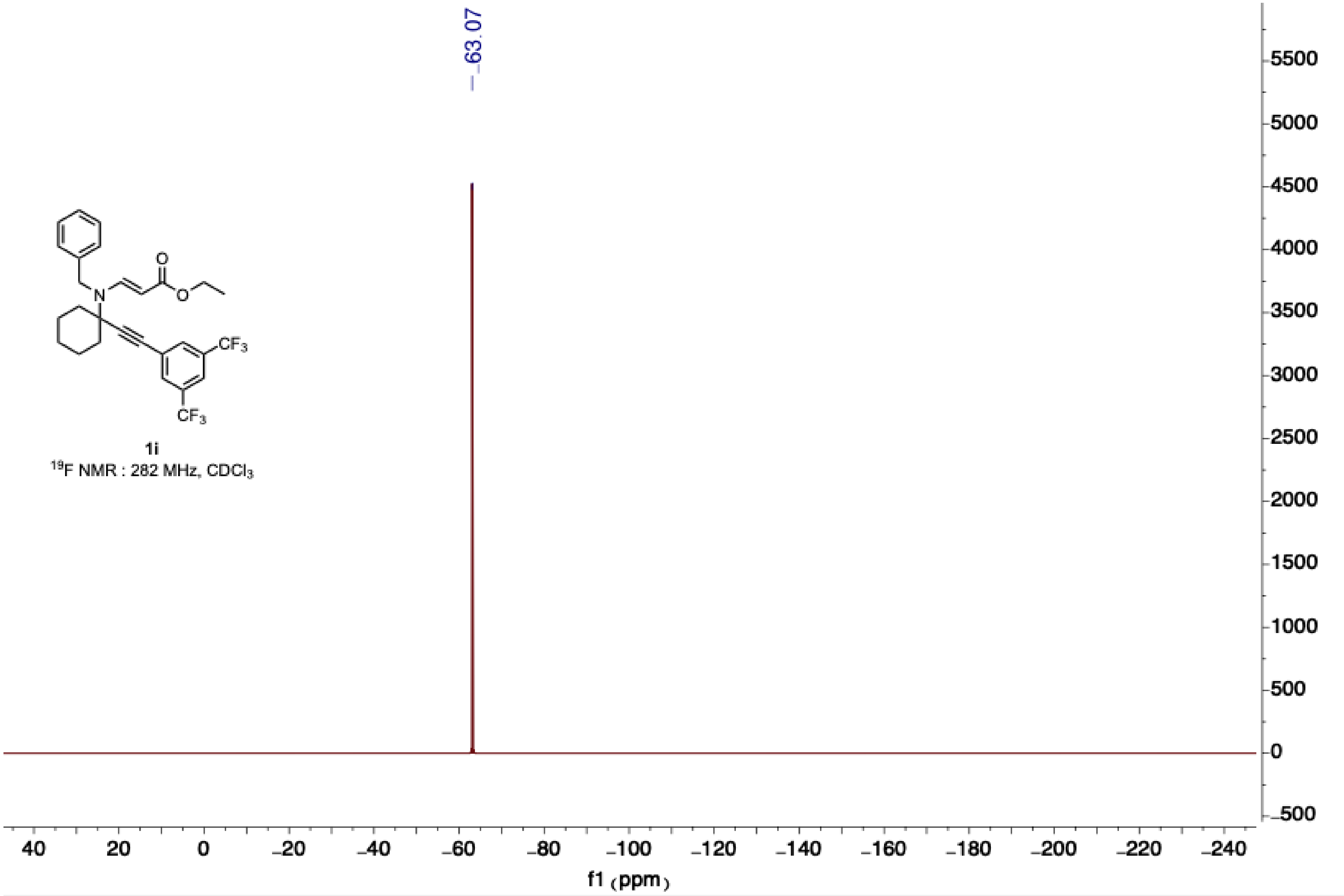

-63.07
CF3
CF3
N
O
O
1i
19F NMR : 282 MHz, CDCl3
5500
5000
4500
4000
3500
3000
2500
2000
1500
1000
500
0
-500
40
20
0
-20
-40
-60
-80
-100
-120
-140
-160
-180
-200
-220
-240
f1 (ppm)

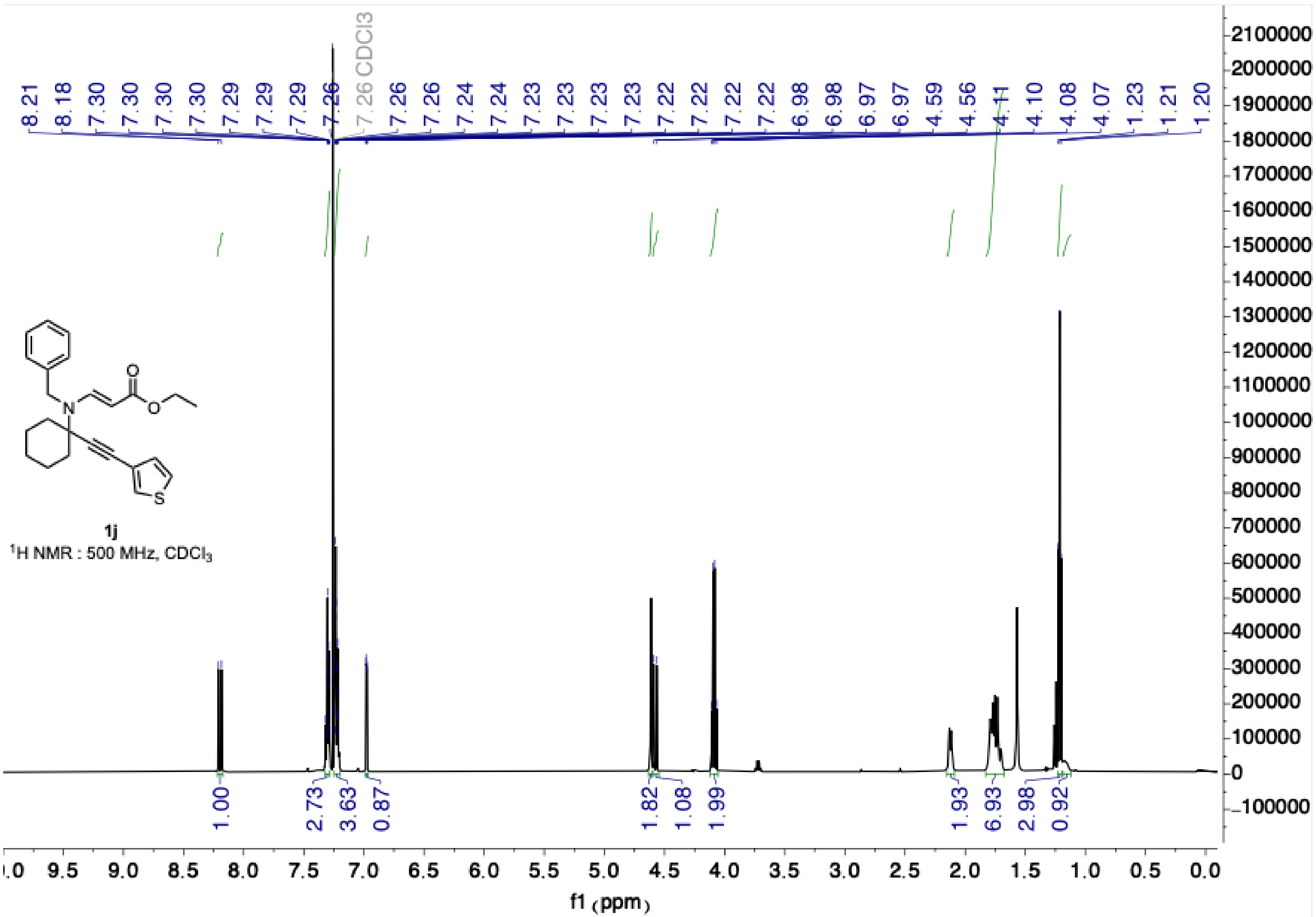
8.21 8.18 7.30 7.30 7.30 7.30 7.29 7.29 7.29 7.26 7.26 CDCl3 7.26 7.26 7.24 7.24 7.23 7.23 7.23 7.23 7.22 7.22 7.22 7.22 6.98 6.98 6.97 6.97 4.59 4.56 4.11 4.10 4.08 4.07 1.23 1.21 1.20
1.00 2.73 3.63 0.87 1.82 1.08 1.99 1.93 6.93 2.98 0.92
O
N
O
S
1j
1H NMR : 500 MHz, CDCl3
f1 (ppm)

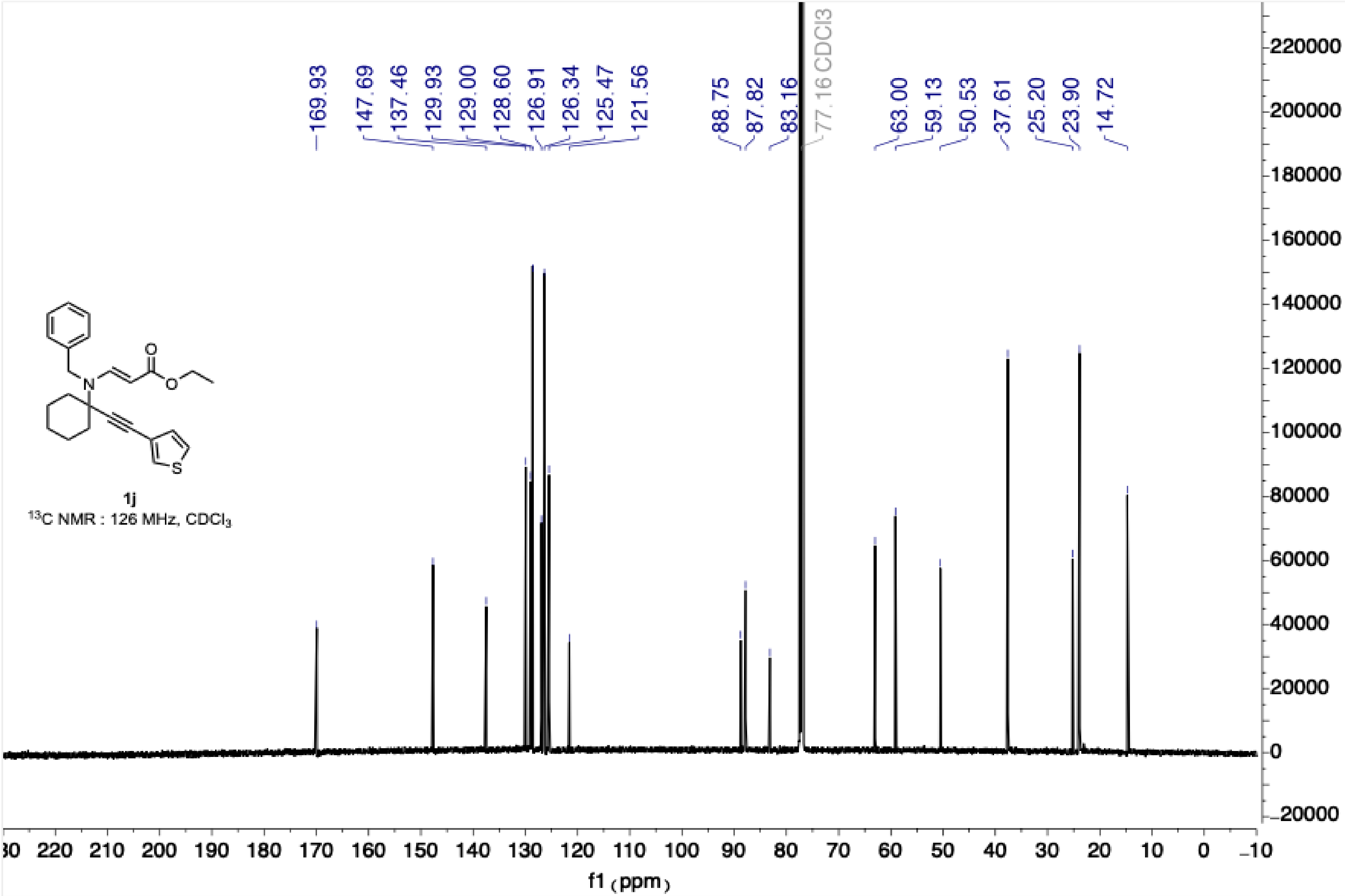

1j

^{13}C NMR : 126 MHz, $CDCl_3$

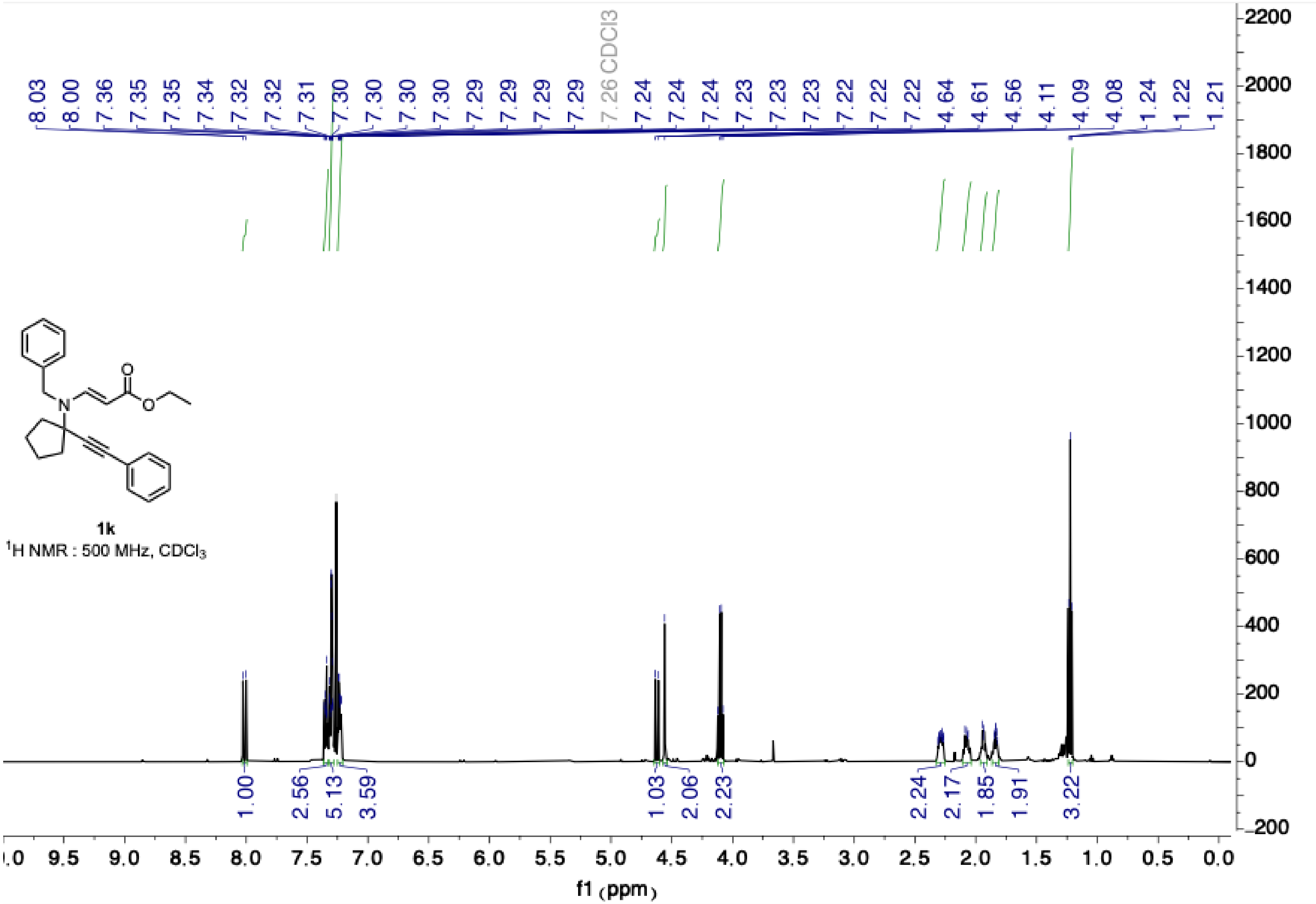

1k

^{1}H NMR : 500 MHz, $CDCl_3$

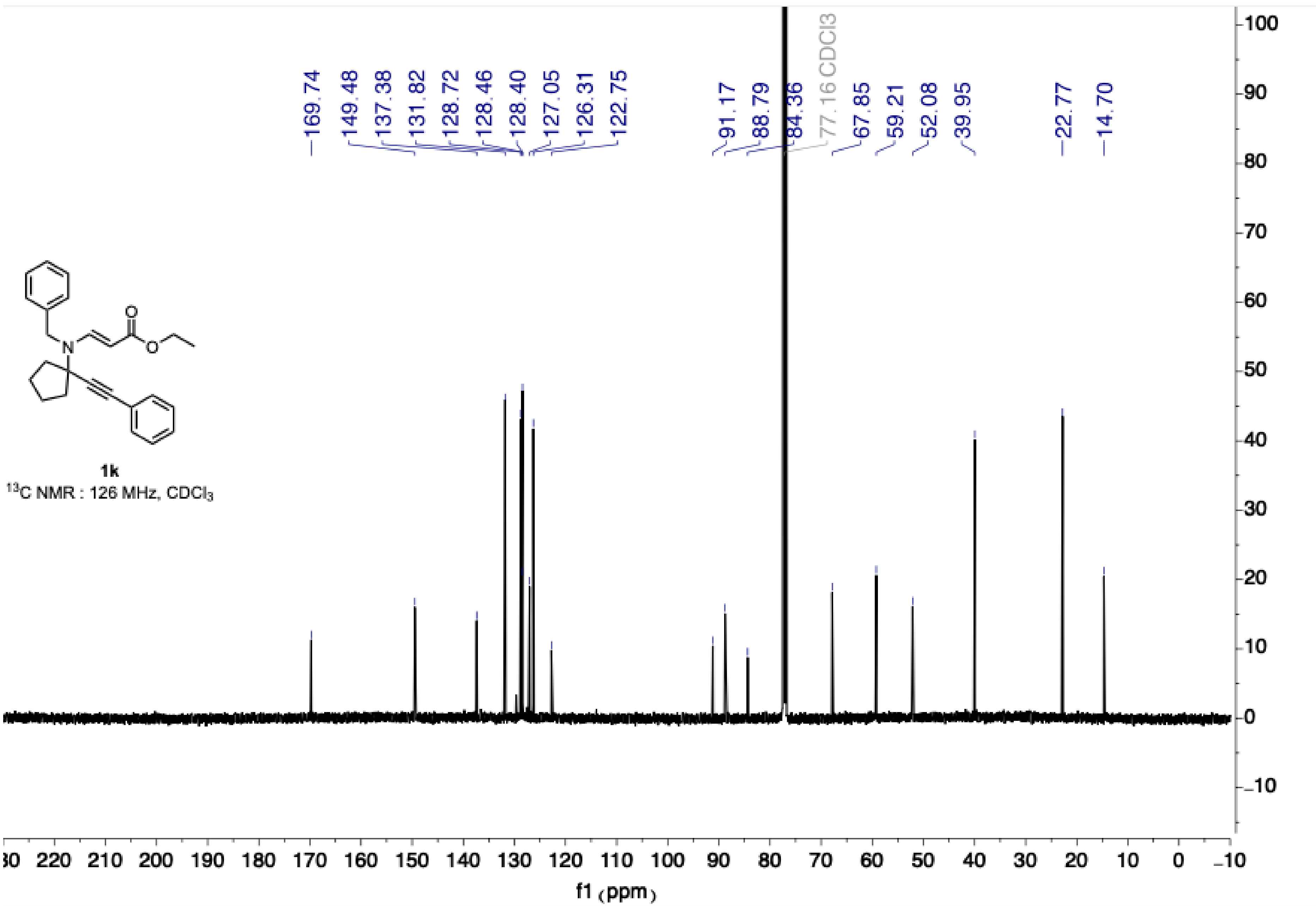
169.74
149.48
137.38
131.82
128.72
128.46
128.40
127.05
126.31
122.75
91.17
88.79
84.36
77.16 CDCl3
67.85
59.21
52.08
39.95
22.77
14.70
1k
^{13}C NMR : 126 MHz, CDCl$_3$
f1 (ppm)

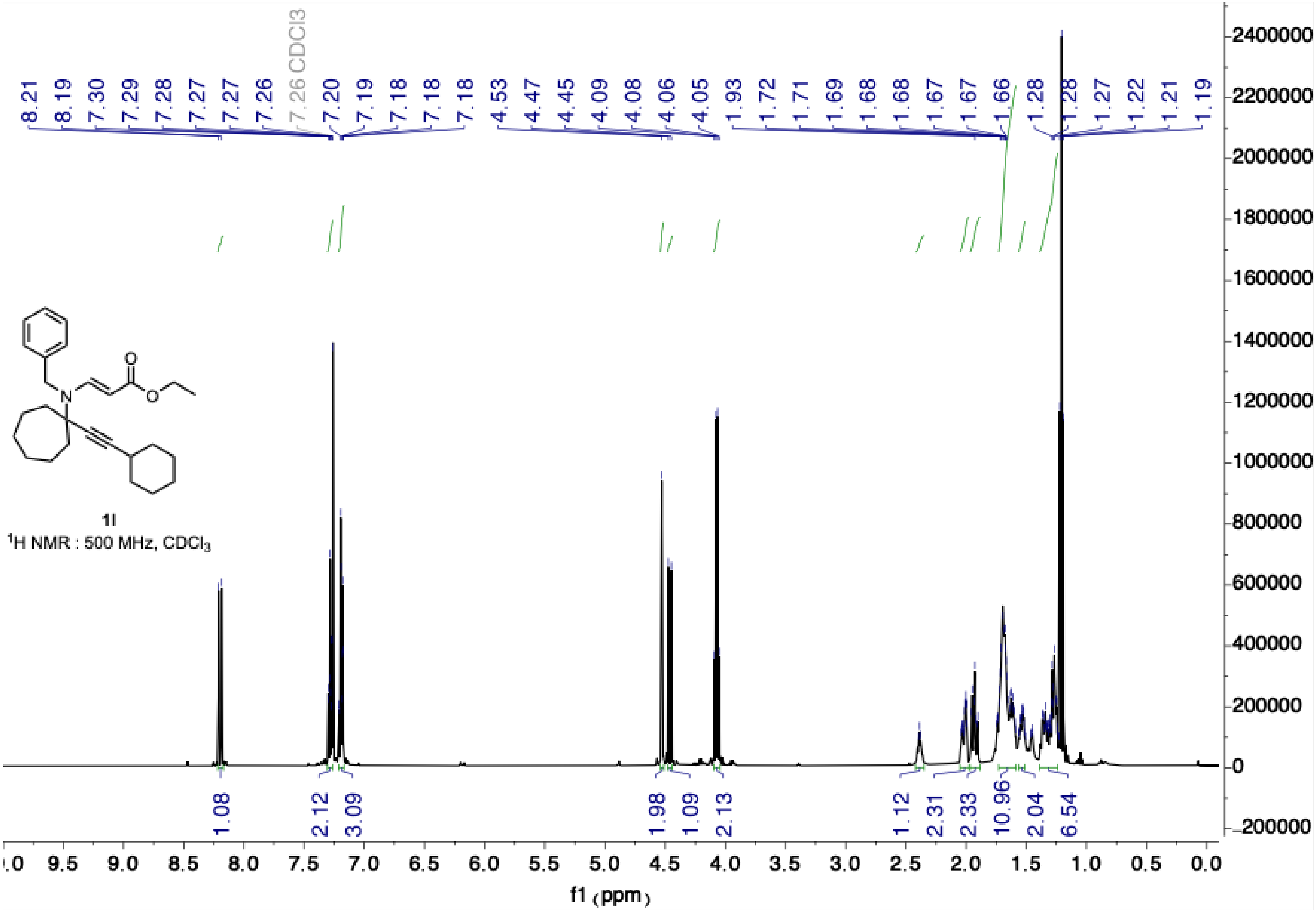

1l
^{1}H NMR : 500 MHz, CDCl$_3$
f1 (ppm)

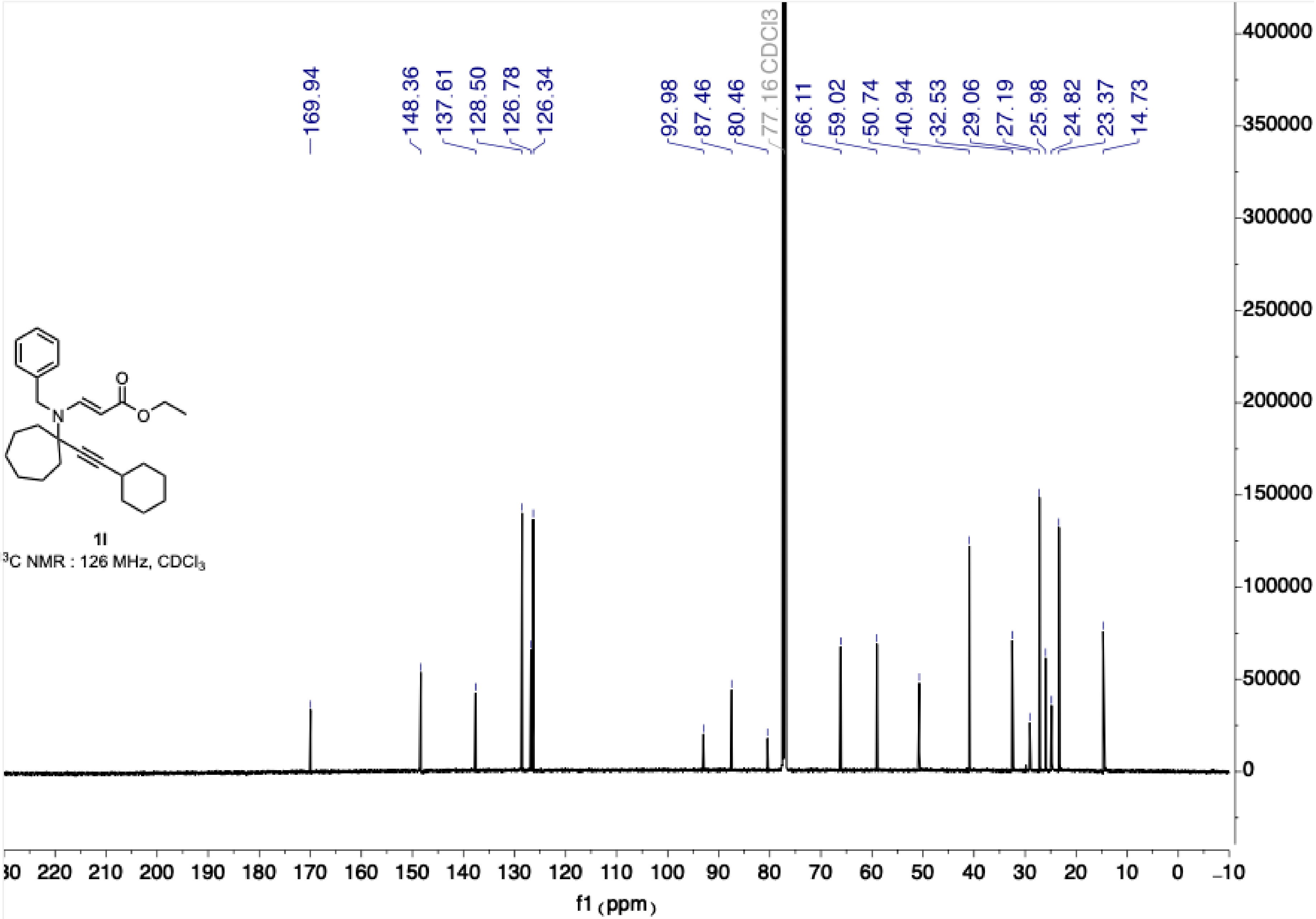

1l

^{13}C NMR : 126 MHz, $CDCl_3$

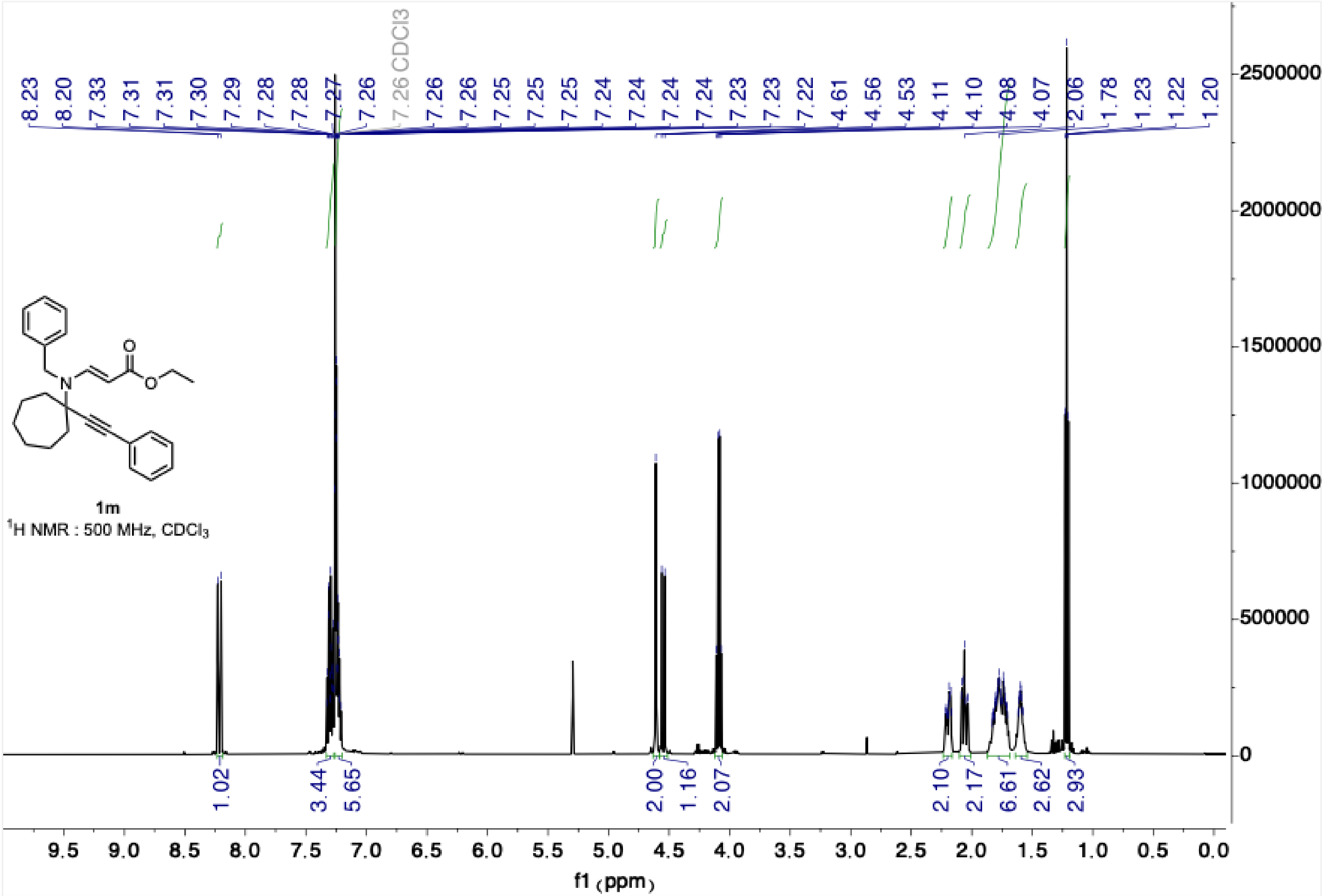
1m
1H NMR : 500 MHz, CDCl3
CDCl3
f1 (ppm)

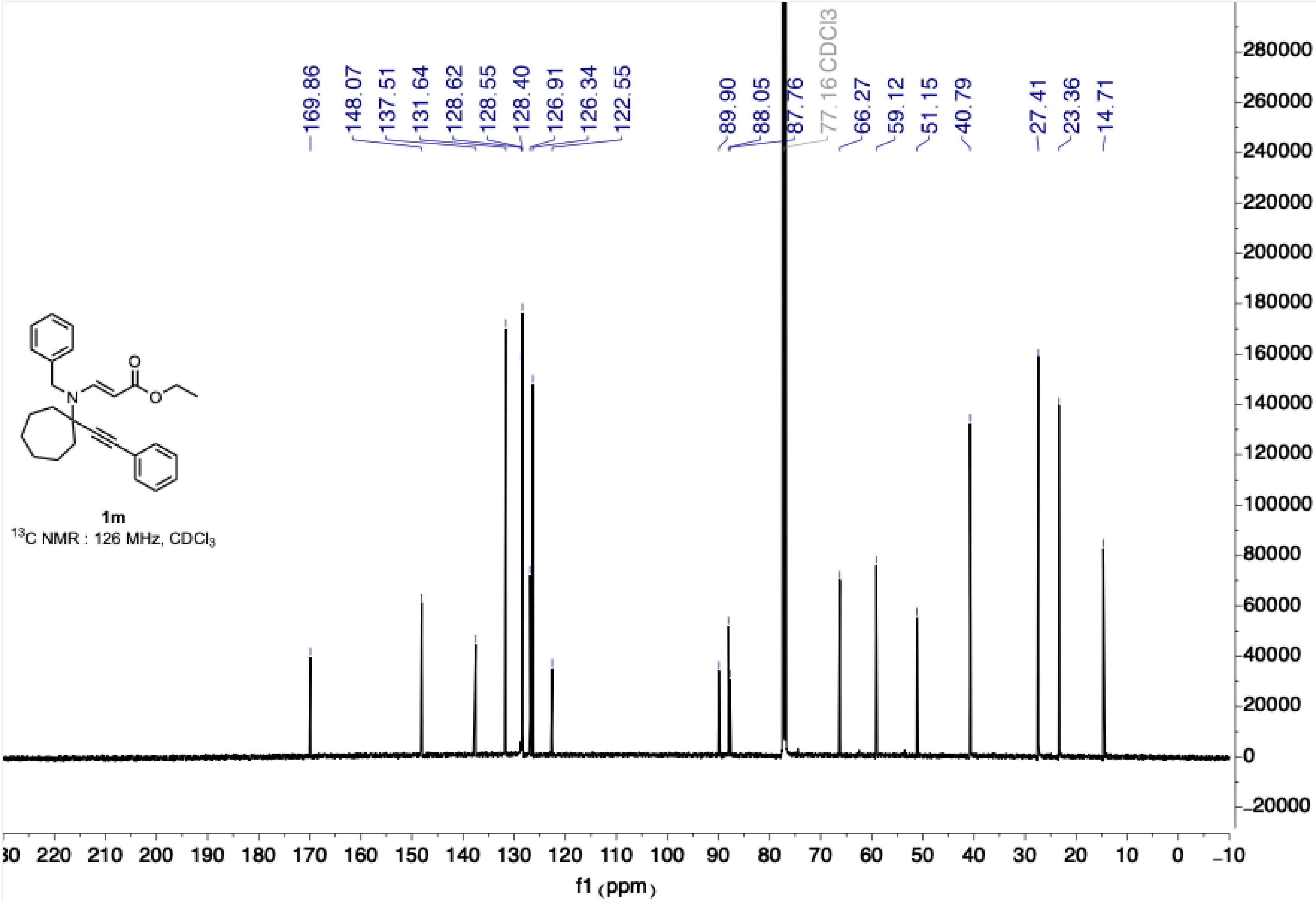

1m

^{13}C NMR : 126 MHz, $CDCl_3$

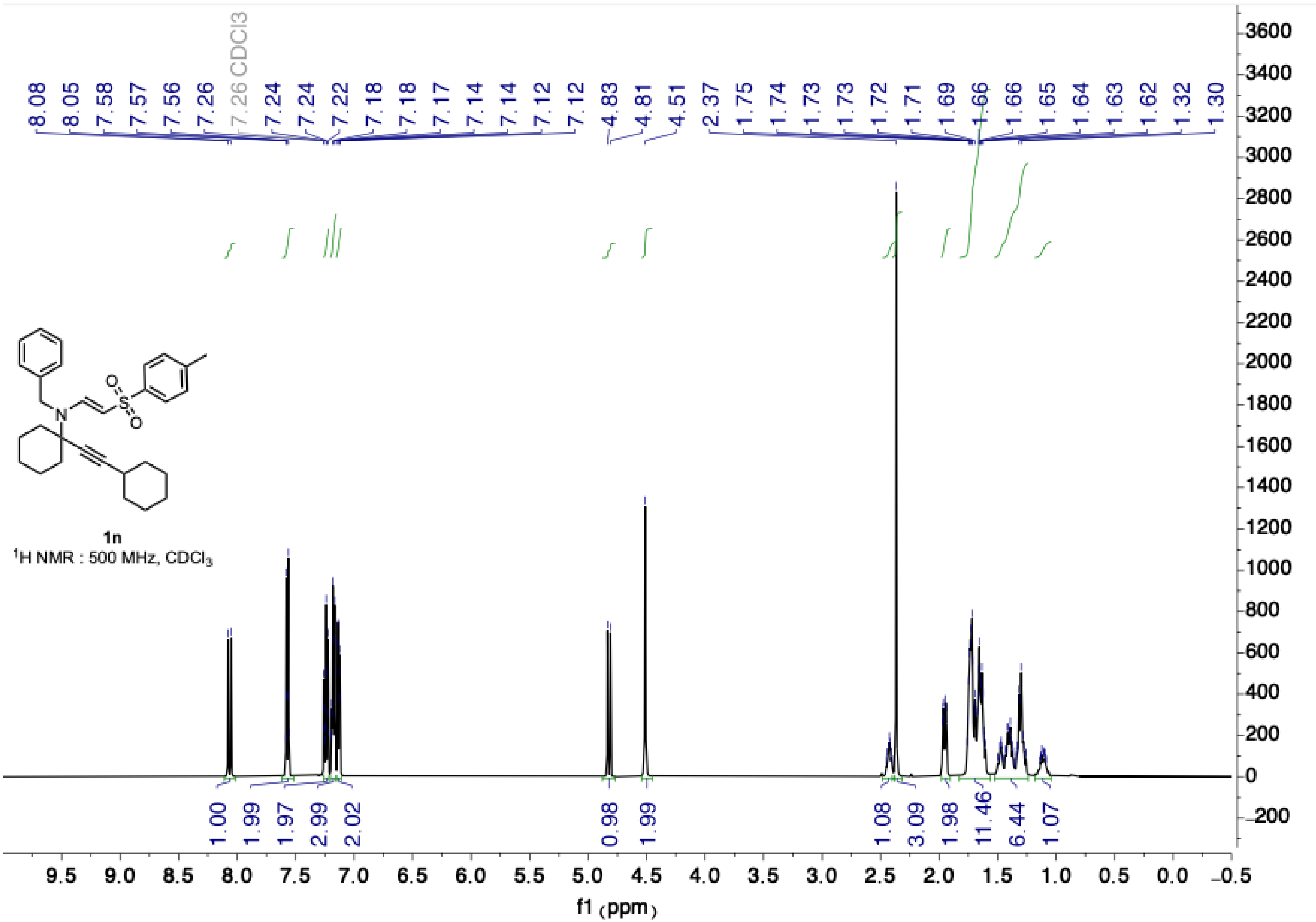
8.08 8.05 7.58 7.57 7.56 7.26 7.26 CDCl3 7.24 7.24 7.22 7.18 7.18 7.17 7.14 7.14 7.12 7.12 4.83 4.81 4.51 2.37 1.75 1.74 1.73 1.73 1.72 1.71 1.69 1.66 1.66 1.65 1.64 1.63 1.62 1.32 1.30
1.00 1.99 1.97 2.99 2.02 0.98 1.99 1.08 3.09 1.98 11.46 6.44 1.07
1n
1H NMR : 500 MHz, CDCl3
f1 (ppm)

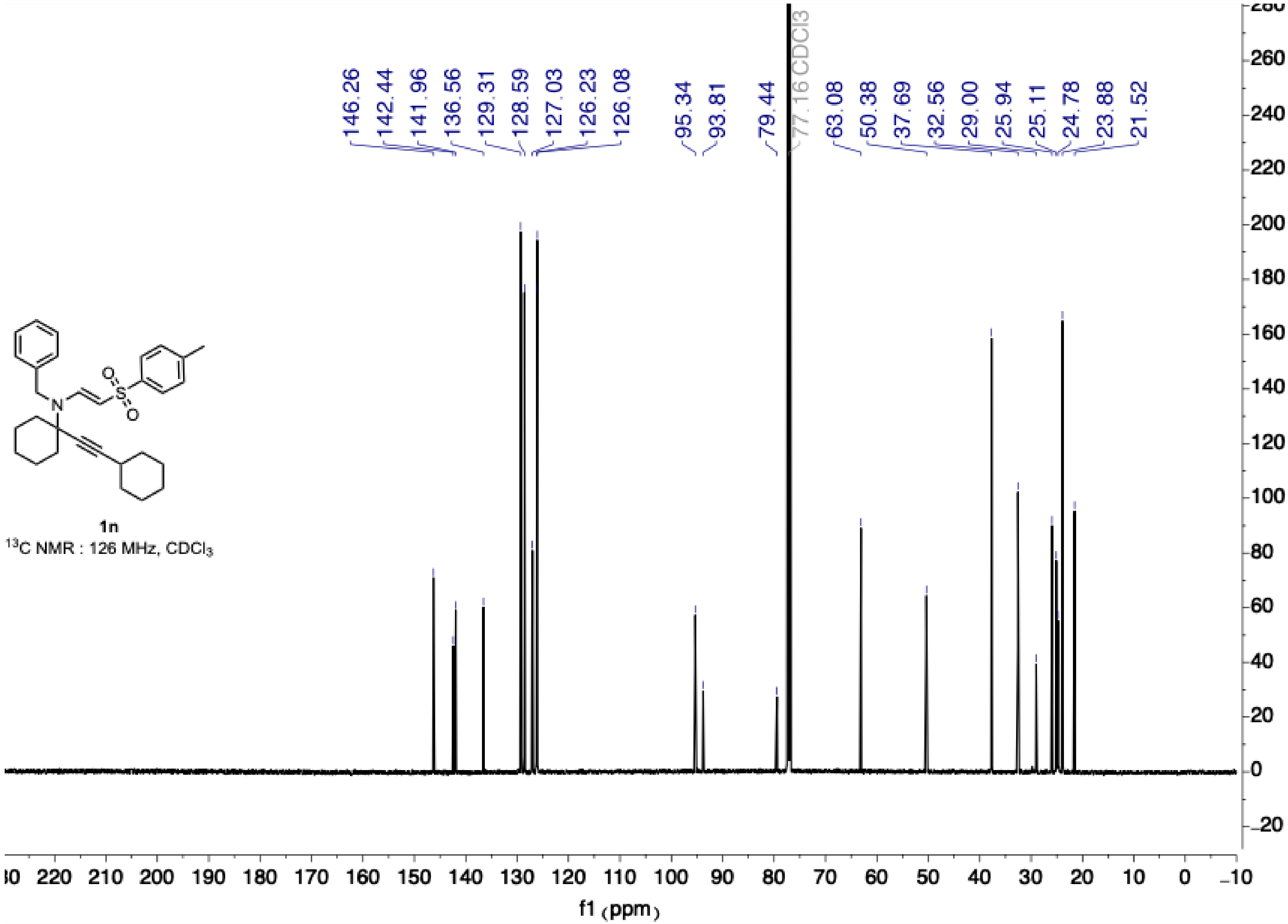

146.26
142.44
141.96
136.56
129.31
128.59
127.03
126.23
126.08
95.34
93.81
79.44
77.16 CDCl3
63.08
50.38
37.69
32.56
29.00
25.94
25.11
24.78
23.88
21.52
1n
13C NMR : 126 MHz, CDCl3
f1 (ppm)

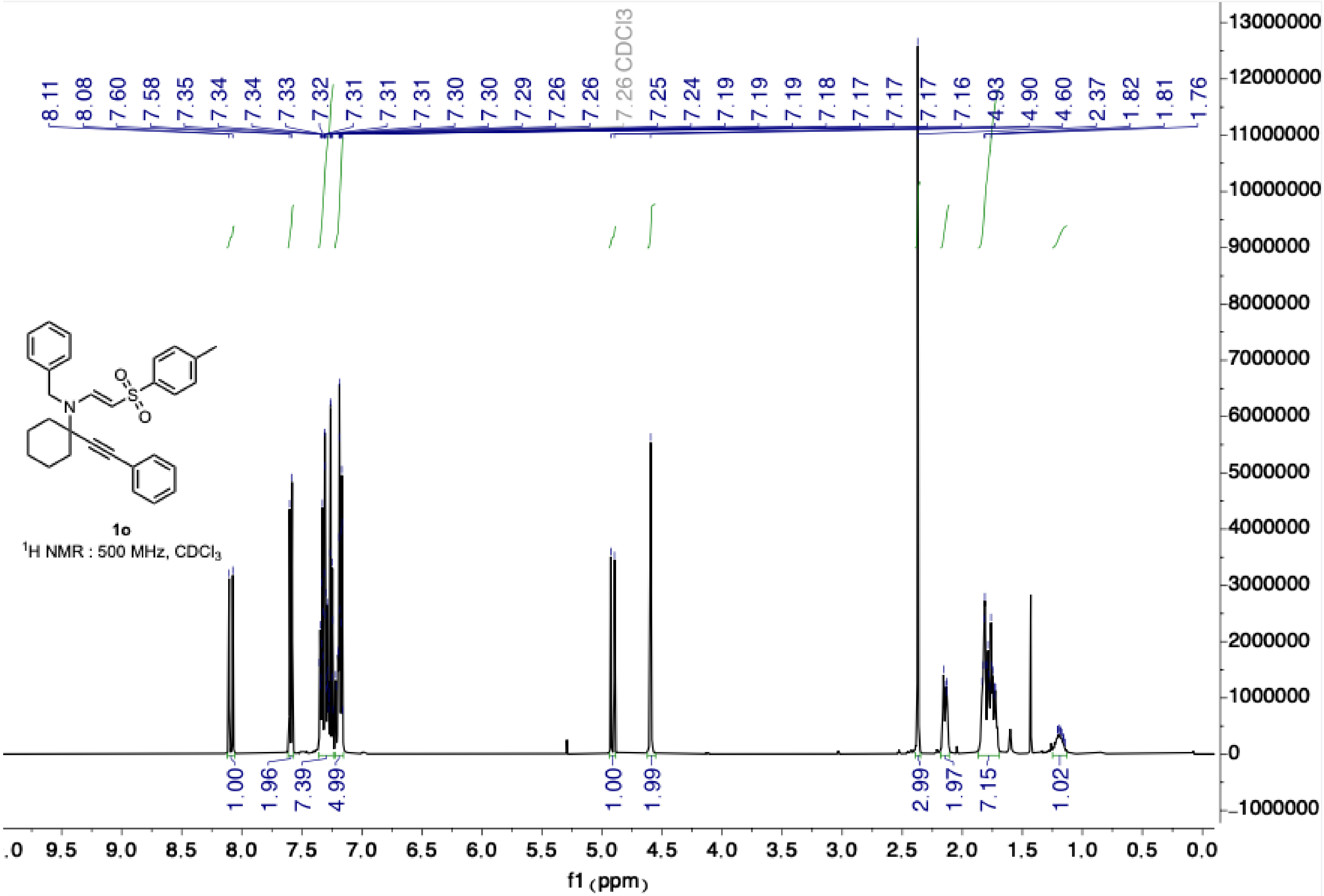

1o
^{1}H NMR : 500 MHz, CDCl$_3$
f1 (ppm)

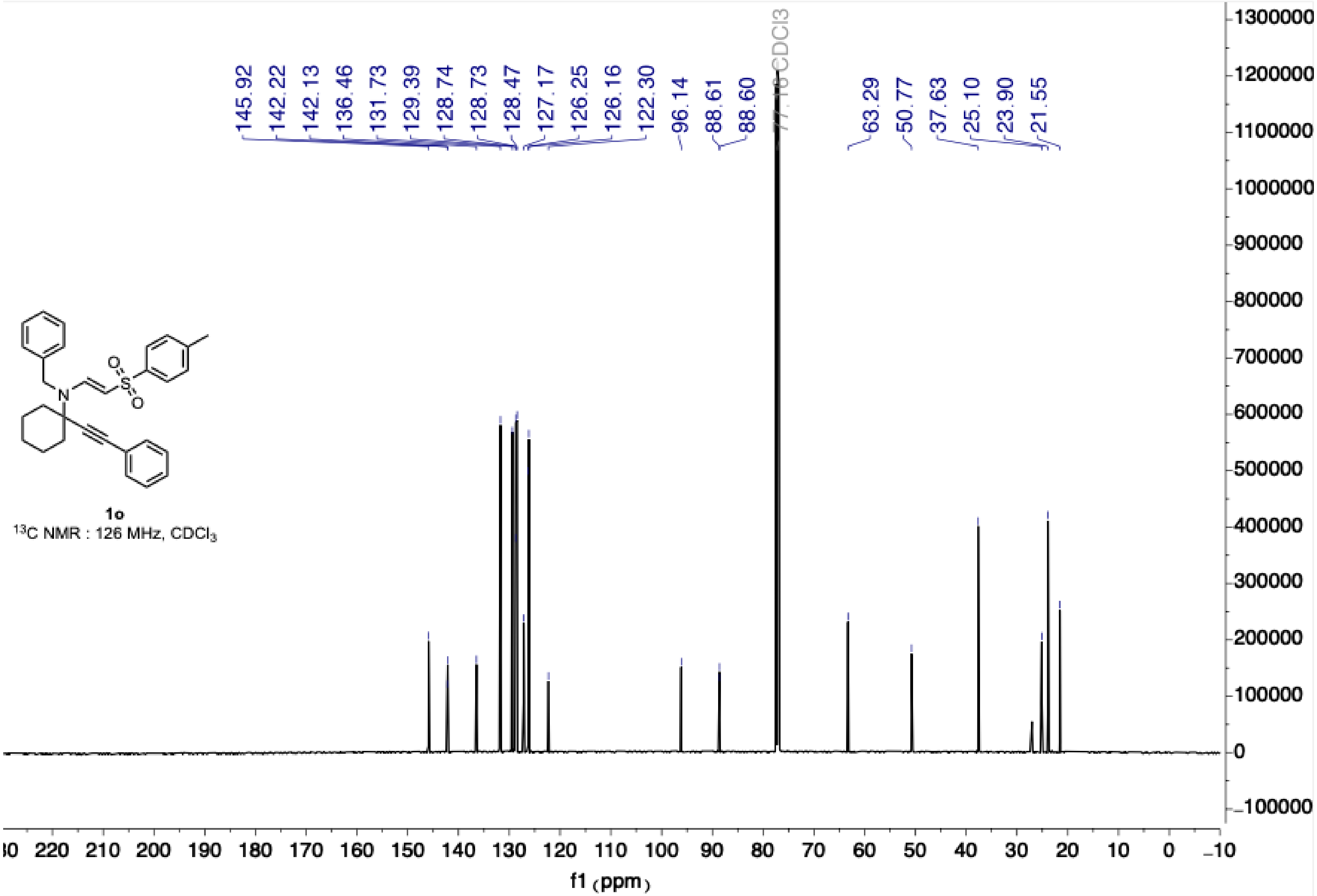
145.92
142.22
142.13
136.46
131.73
129.39
128.74
128.73
128.47
127.17
126.25
126.16
122.30
96.14
88.61
88.60
77.16 CDCl3
63.29
50.77
37.63
25.10
23.90
21.55
1o
^{13}C NMR : 126 MHz, $CDCl_3$
f1 (ppm)

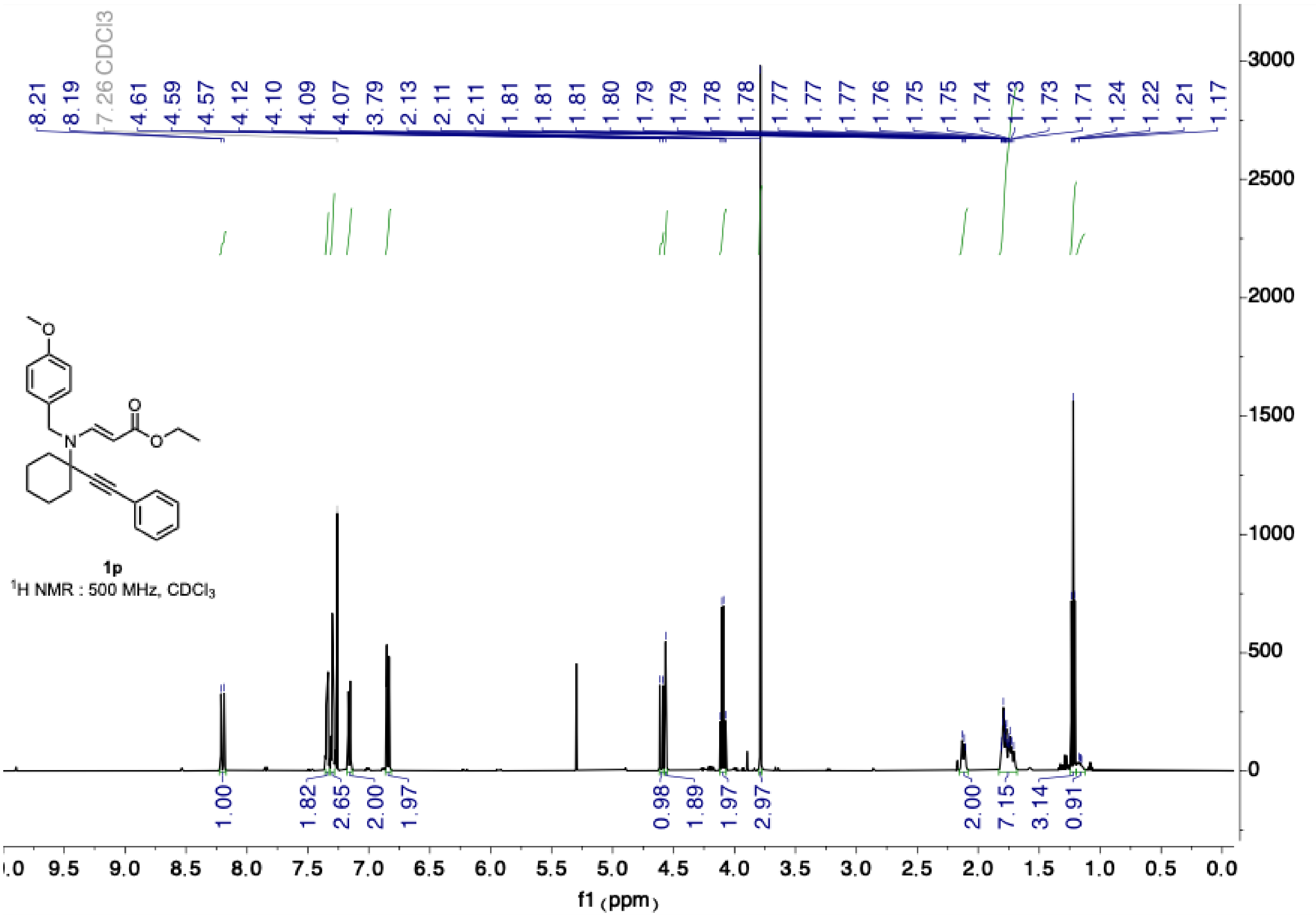

1p

^{1}H NMR : 500 MHz, $CDCl_3$

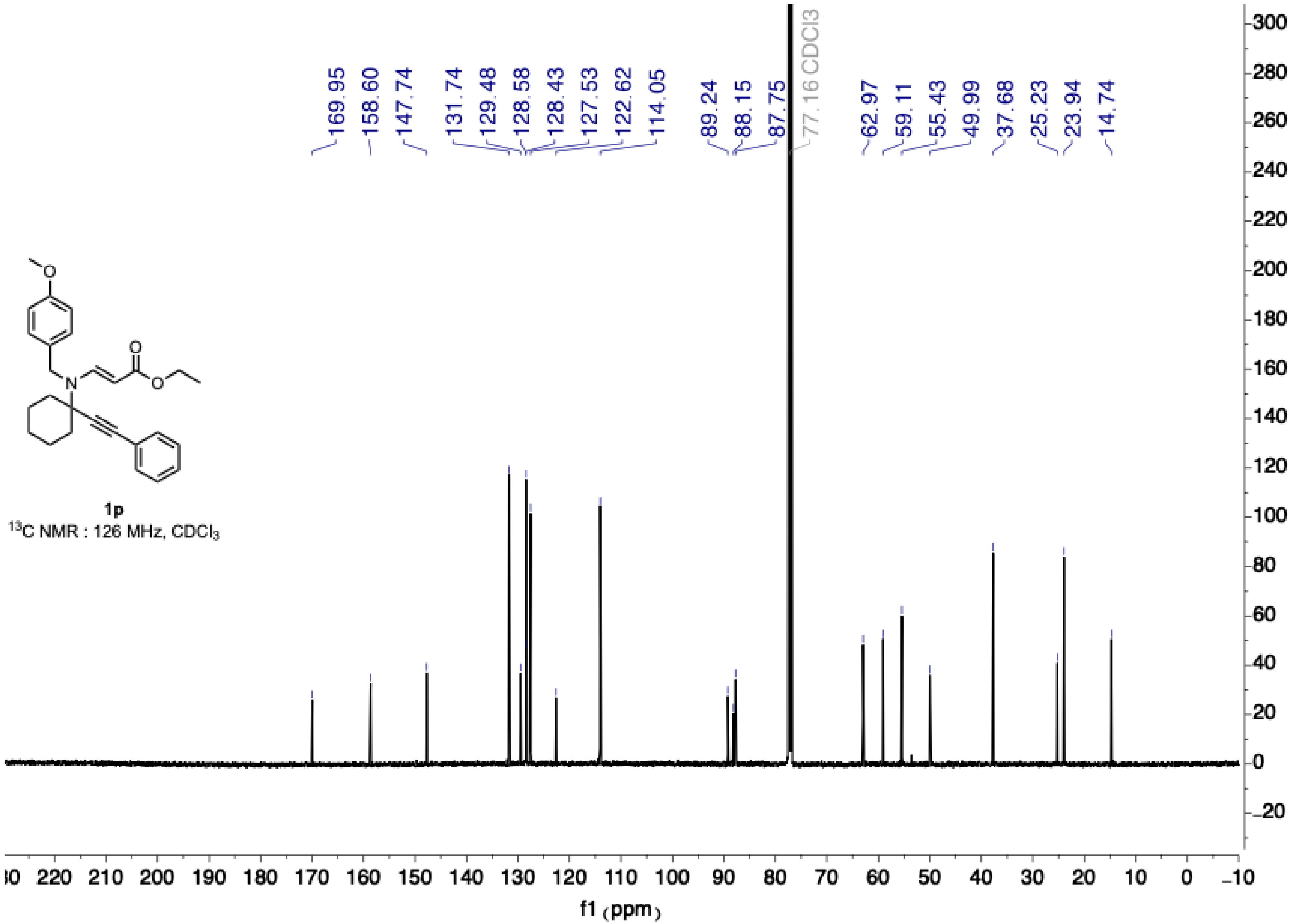
169.95
158.60
147.74
131.74
129.48
128.58
128.43
127.53
122.62
114.05
89.24
88.15
87.75
77.16 CDCl3
62.97
59.11
55.43
49.99
37.68
25.23
23.94
14.74
1p
^{13}C NMR : 126 MHz, $CDCl_3$
f1 (ppm)

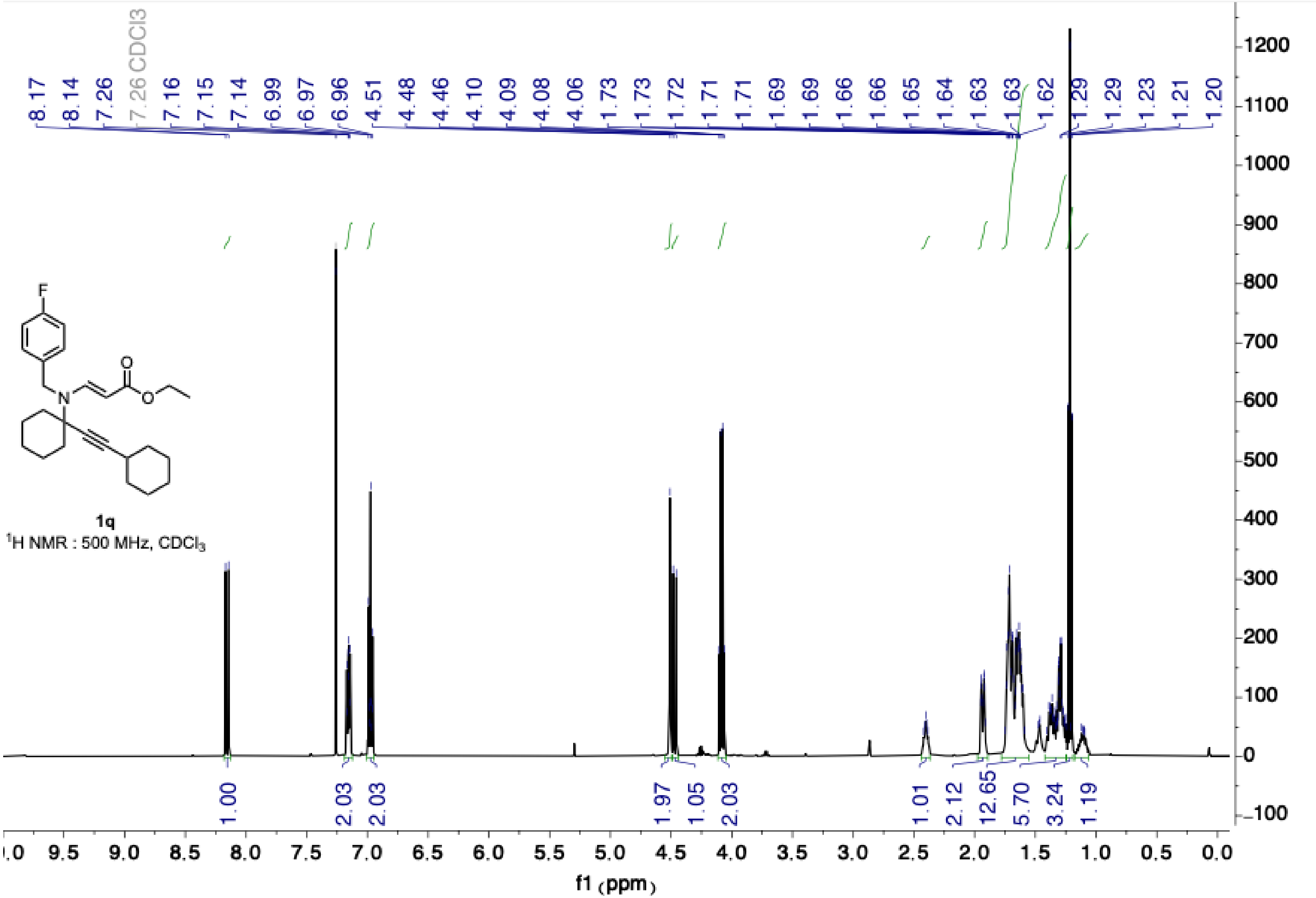

1q
1H NMR : 500 MHz, CDCl3
8.17 8.14 7.26 7.26 CDCl3 7.16 7.15 7.14 6.99 6.97 6.96 4.51 4.48 4.46 4.10 4.09 4.08 4.06 1.73 1.73 1.72 1.71 1.71 1.69 1.69 1.66 1.66 1.65 1.64 1.63 1.63 1.62 1.29 1.29 1.23 1.21 1.20
1.00 2.03 2.03 1.97 1.05 2.03 1.01 2.12 12.65 5.70 3.24 1.19
f1 (ppm)

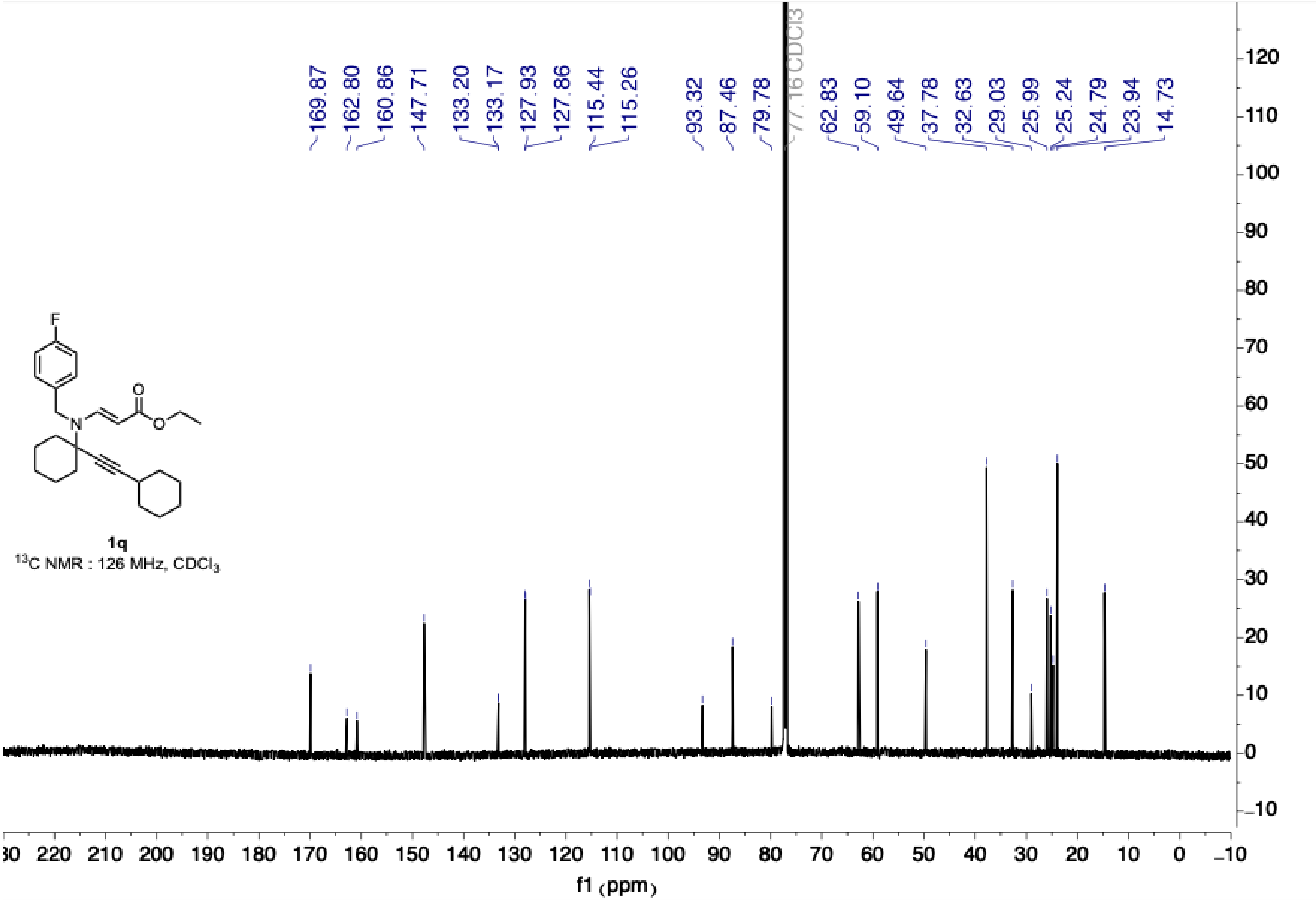
169.87
162.80
160.86
147.71
133.20
133.17
127.93
127.86
115.44
115.26
93.32
87.46
79.78
77.16 CDCl3
62.83
59.10
49.64
37.78
32.63
29.03
25.99
25.24
24.79
23.94
14.73
F
O
N
O
1q
13C NMR : 126 MHz, CDCl3
f1 (ppm)

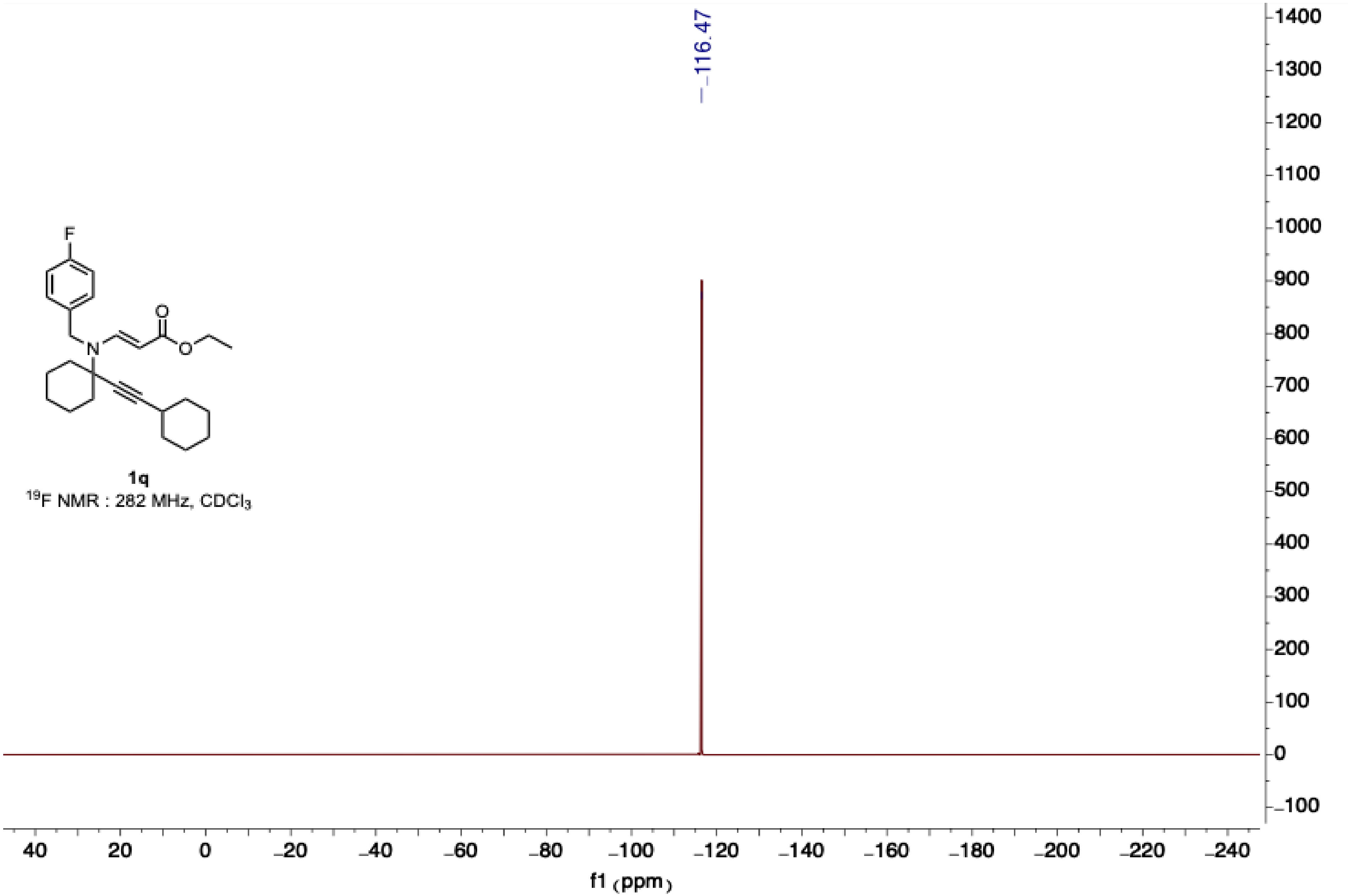

-116.47
F
O
N
O
1q
^{19}F NMR : 282 MHz, CDCl$_3$
1400
1300
1200
1100
1000
900
800
700
600
500
400
300
200
100
0
-100
40 20 0 -20 -40 -60 -80 -100 -120 -140 -160 -180 -200 -220 -240
f1 (ppm)

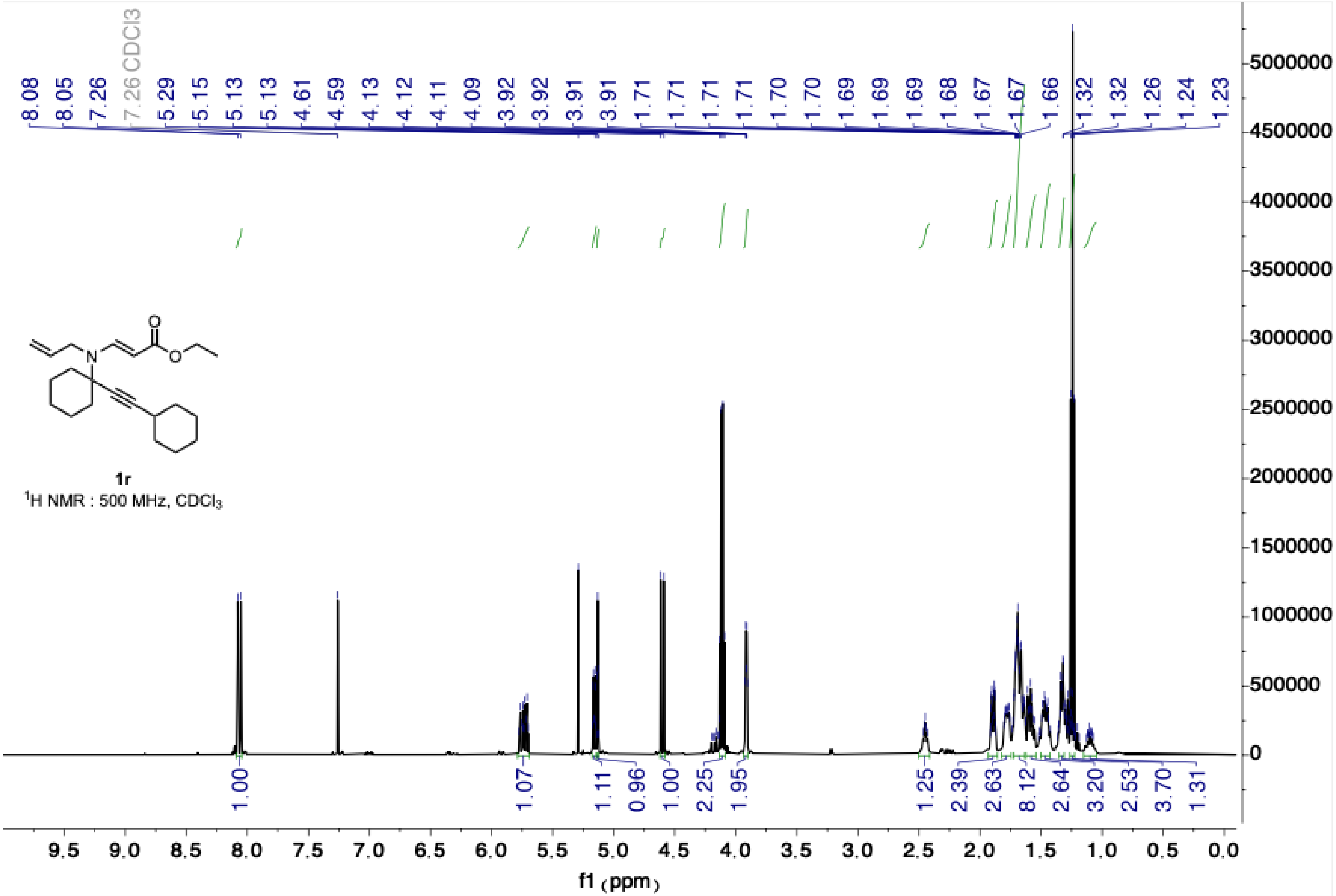
1r
^{1}H NMR : 500 MHz, CDCl$_3$
f1 (ppm)

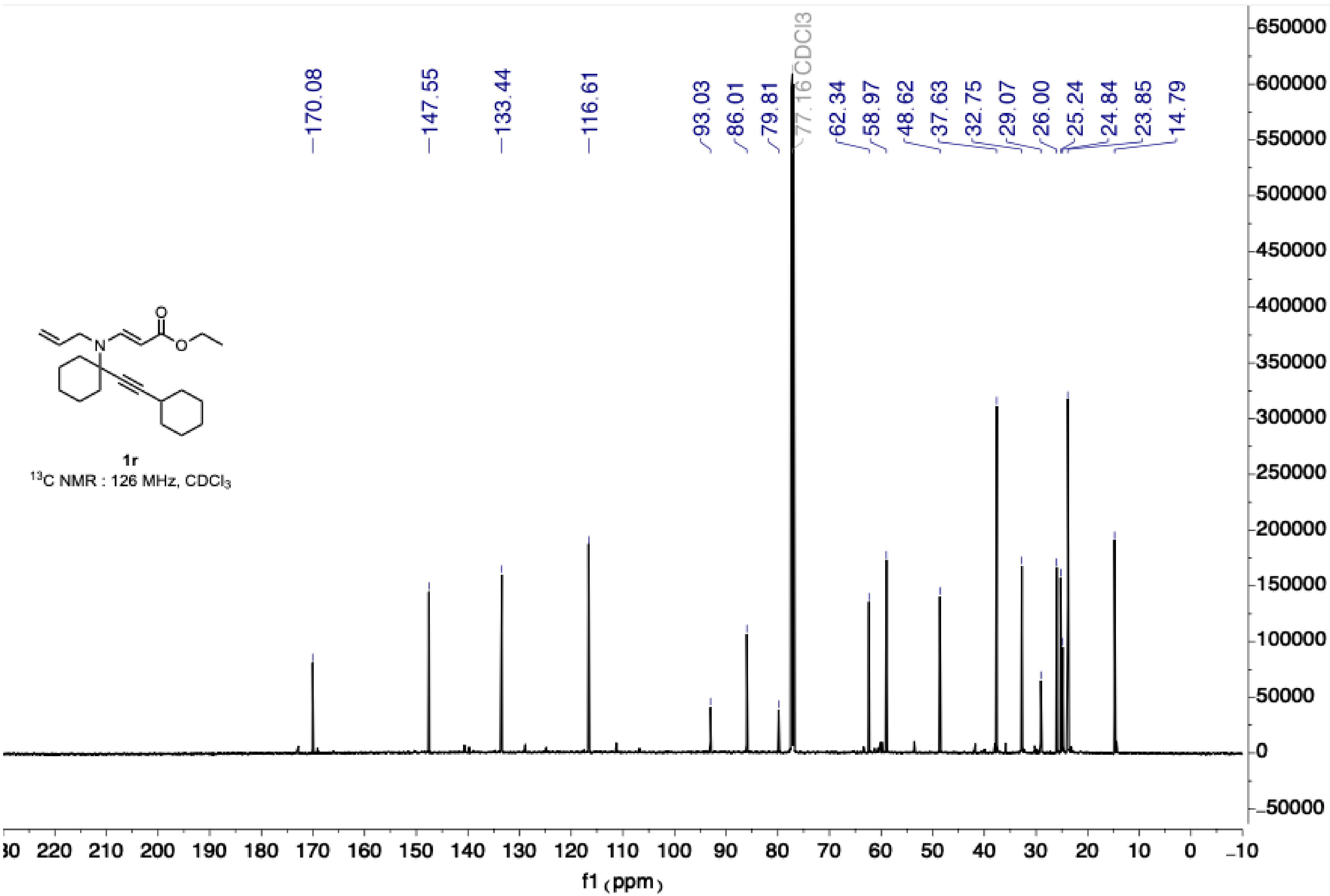

1r
^{13}C NMR : 126 MHz, CDCl$_3$
170.08
147.55
133.44
116.61
93.03
86.01
79.81
77.16 CDCl3
62.34
58.97
48.62
37.63
32.75
29.07
26.00
25.24
24.84
23.85
14.79
f1 (ppm)

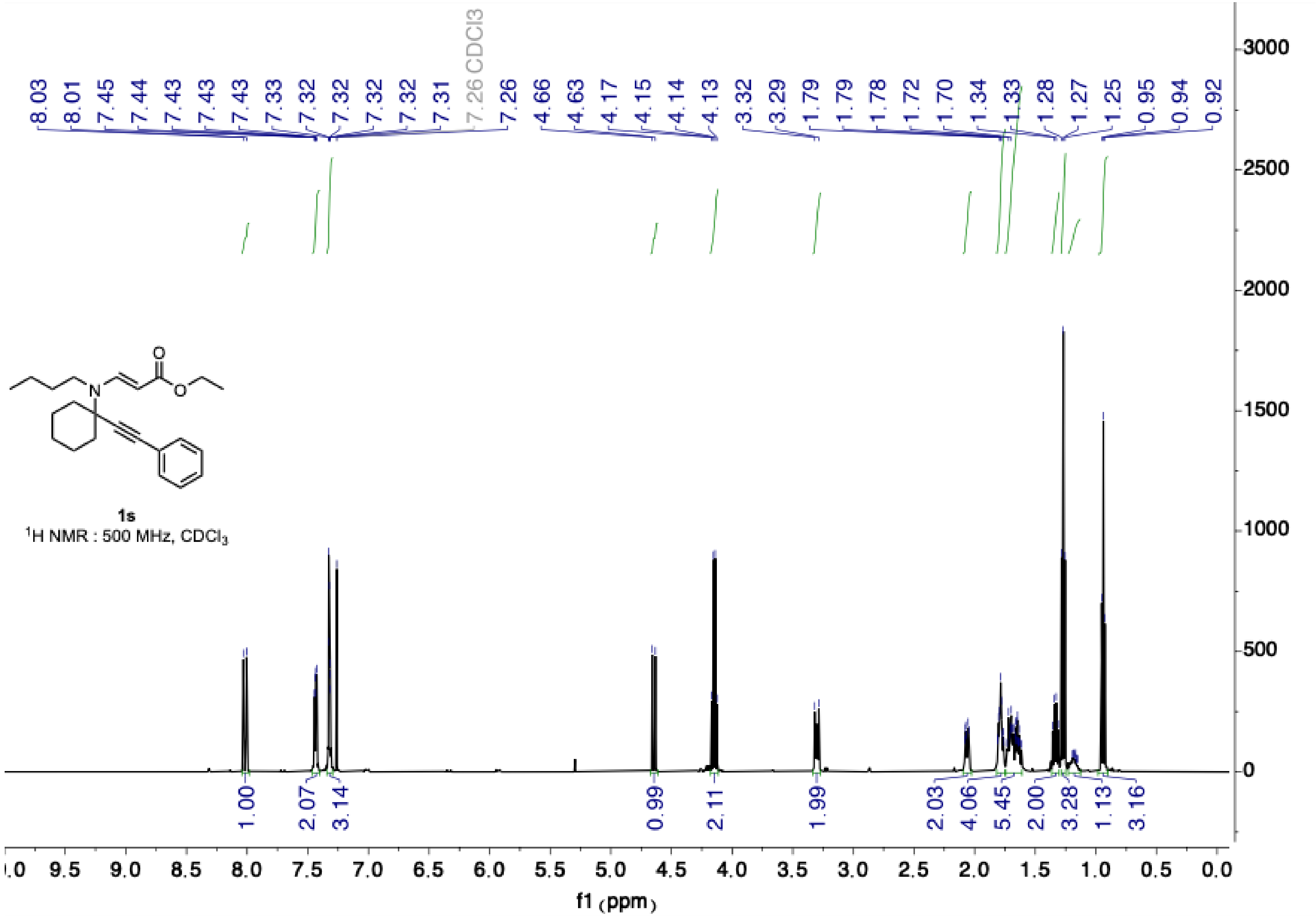

1s

^1H NMR : 500 MHz, $CDCl_3$

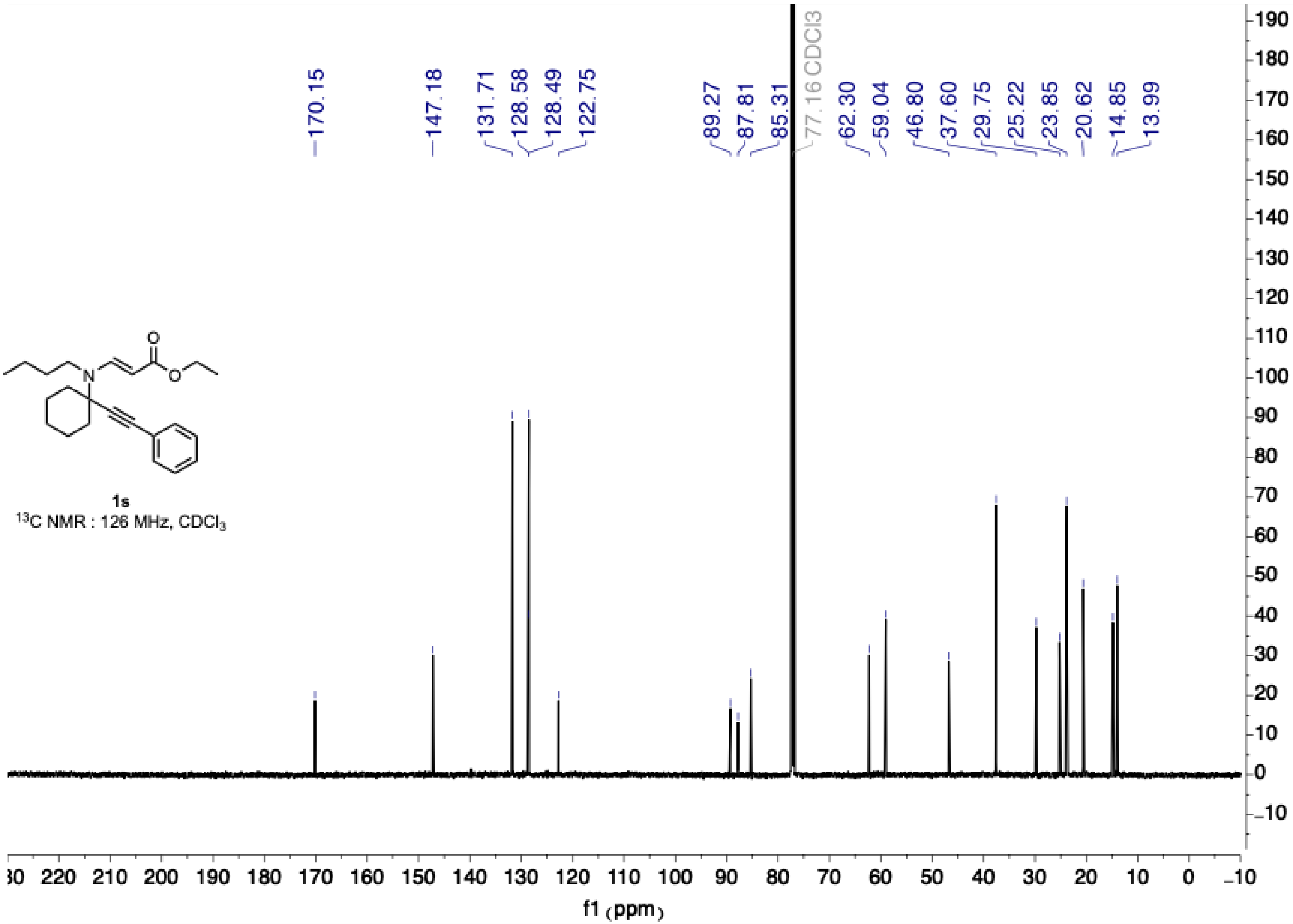

1s

^{13}C NMR : 126 MHz, $CDCl_3$

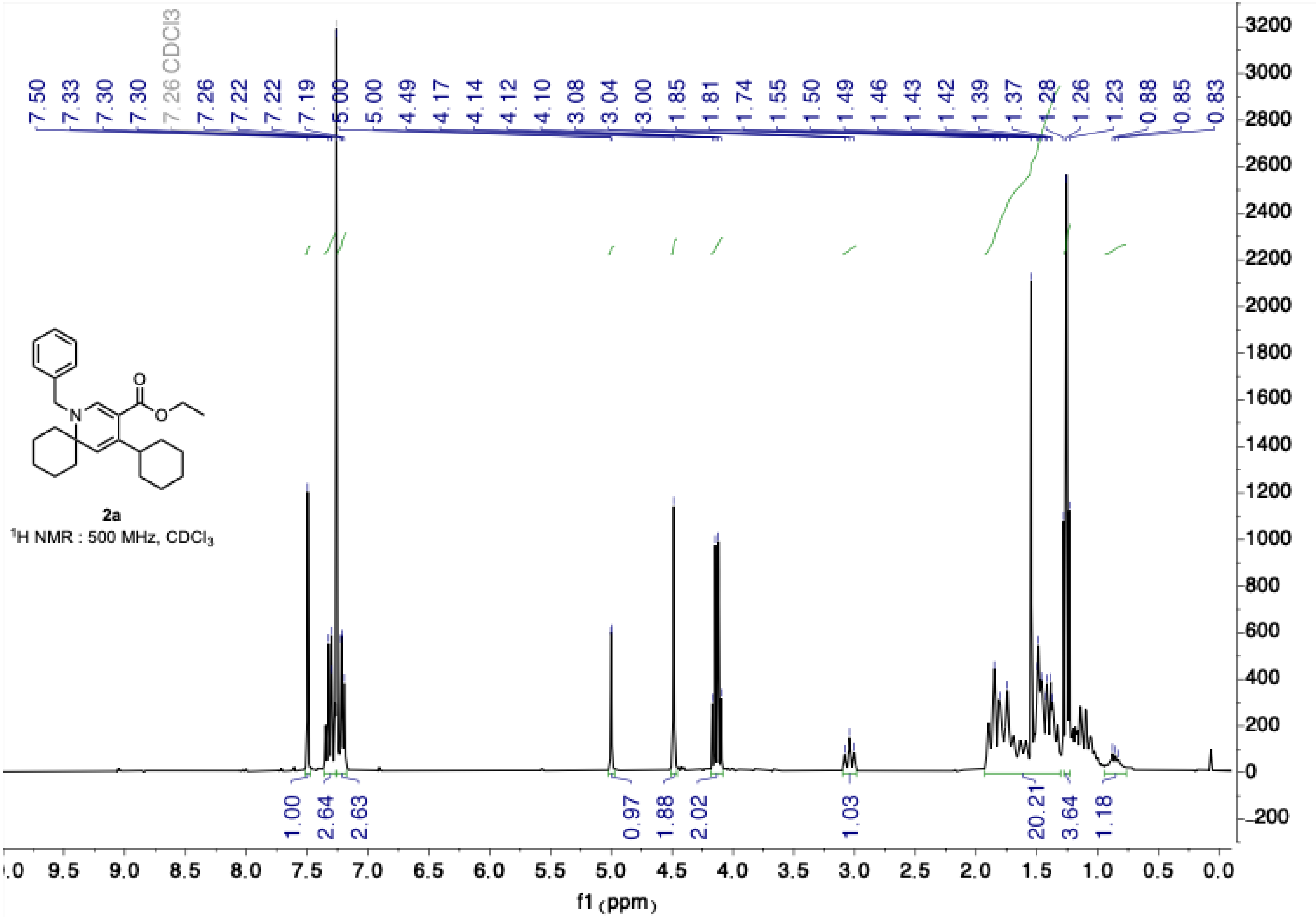
2a
¹H NMR : 500 MHz, CDCl₃
7.26 CDCl3
f1 (ppm)

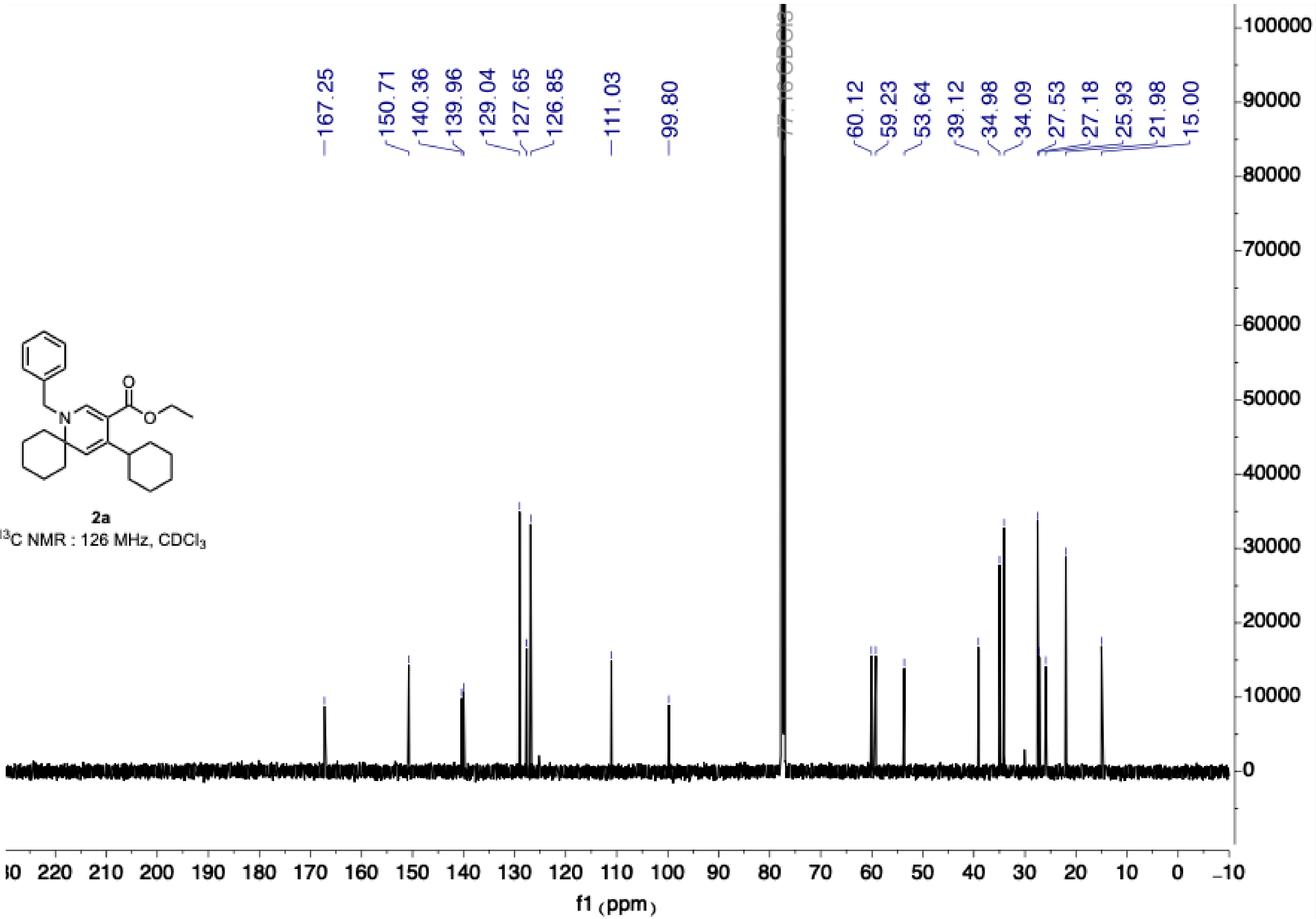

2a

^{13}C NMR : 126 MHz, $CDCl_3$

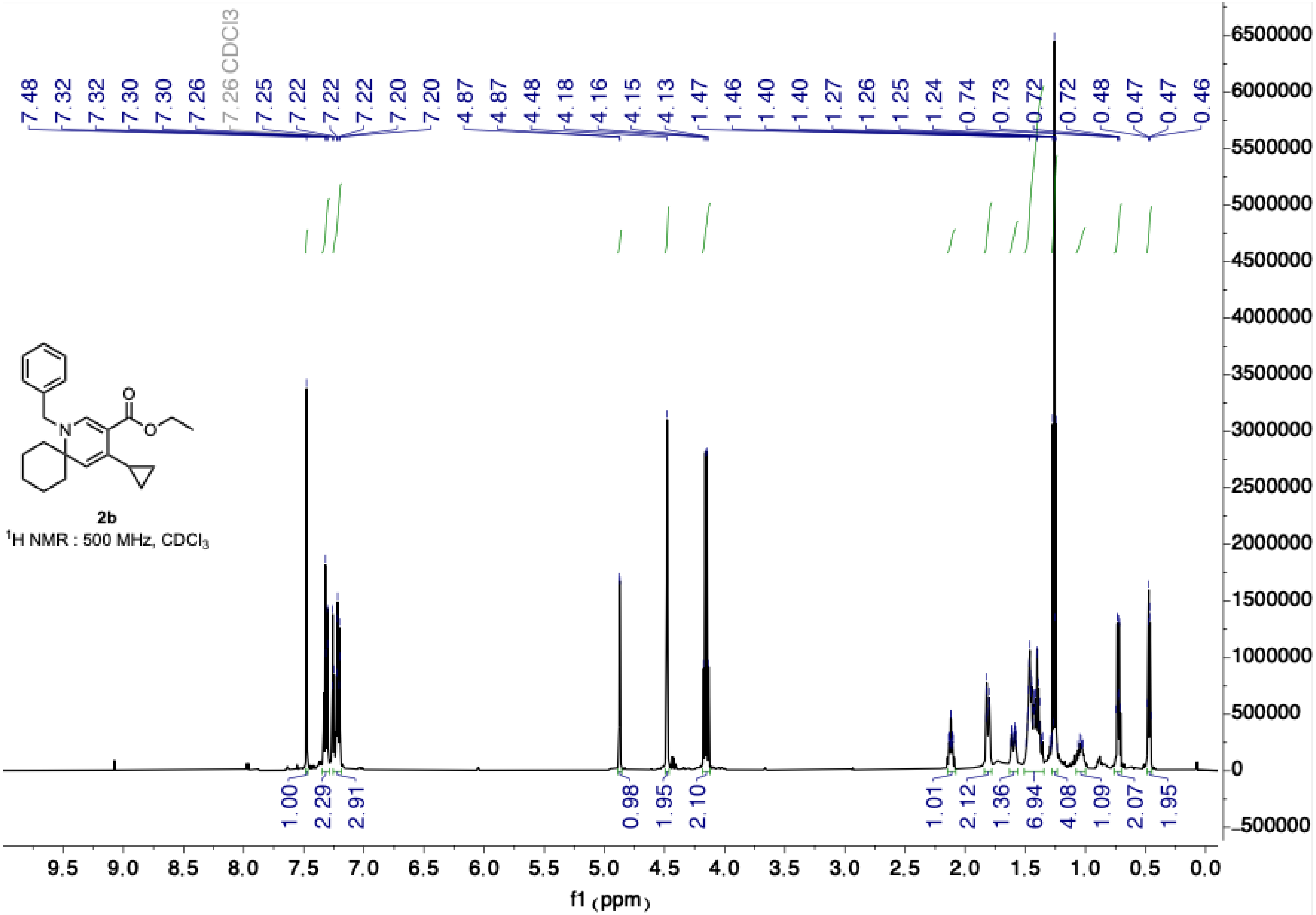

2b
1H NMR : 500 MHz, CDCl3
f1 (ppm)

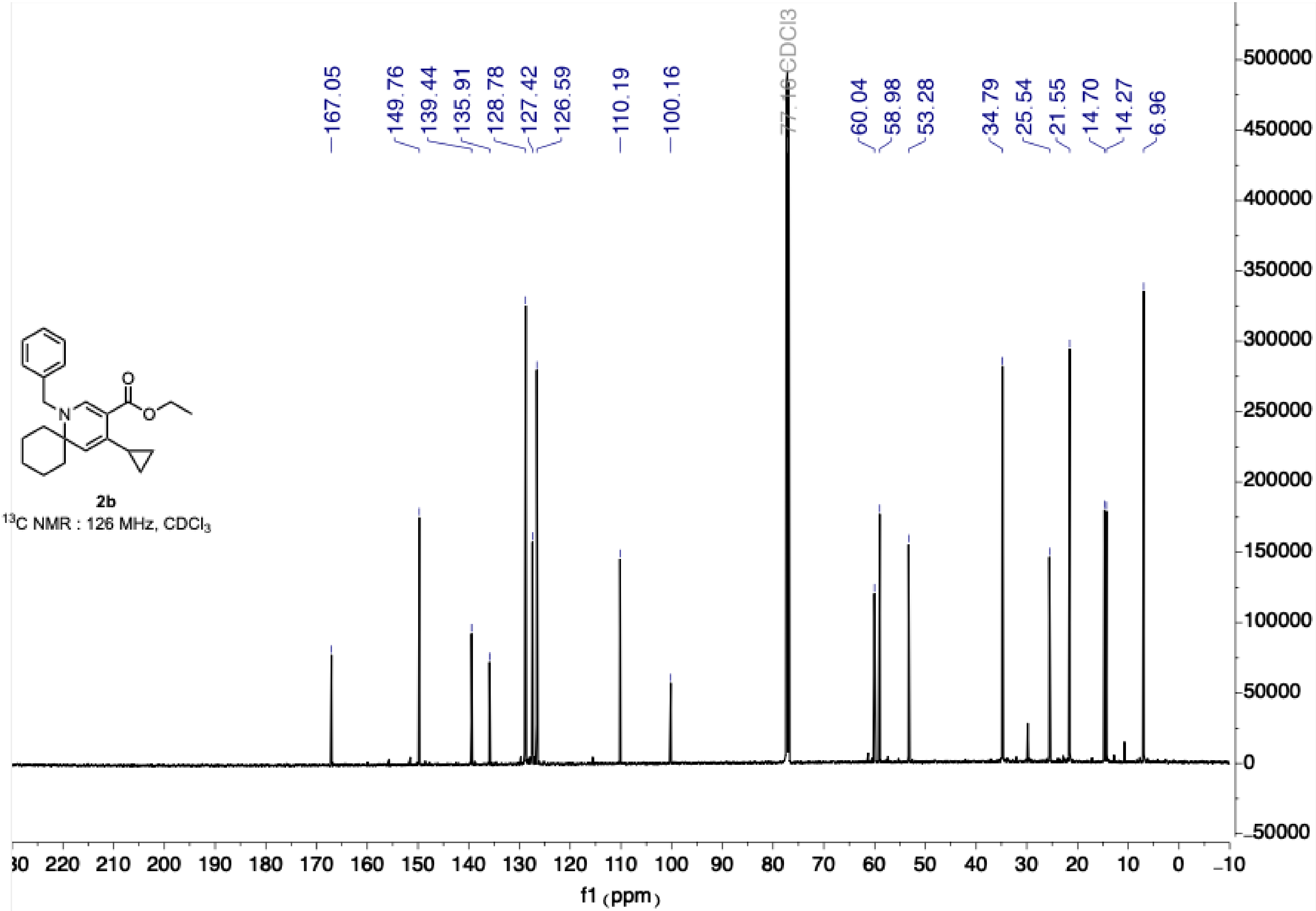
167.05
149.76
139.44
135.91
128.78
127.42
126.59
110.19
100.16
77.16 CDCl3
60.04
58.98
53.28
34.79
25.54
21.55
14.70
14.27
6.96
2b
^{13}C NMR : 126 MHz, $CDCl_3$
f1 (ppm)

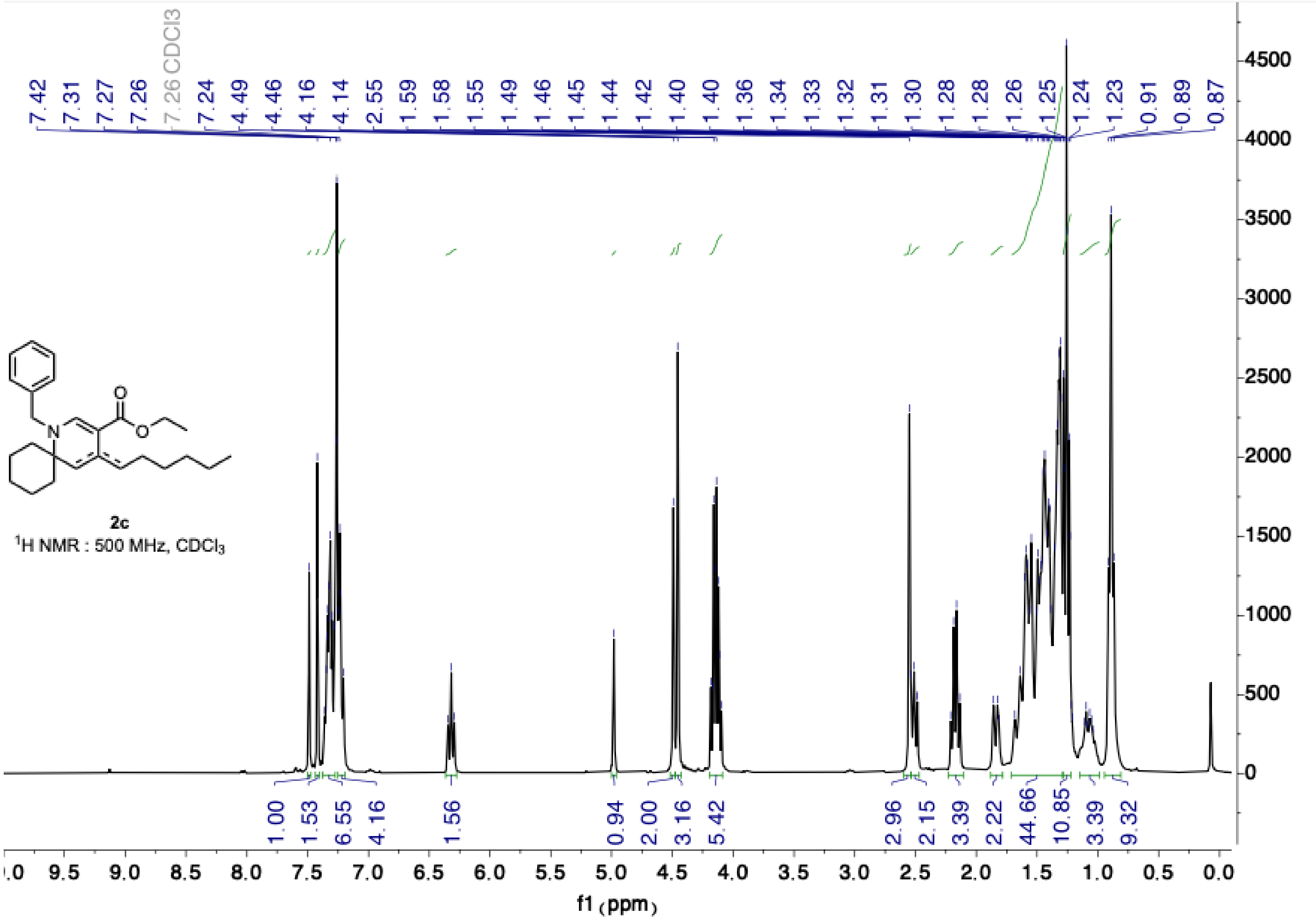
2c
1H NMR : 500 MHz, CDCl3
f1 (ppm)

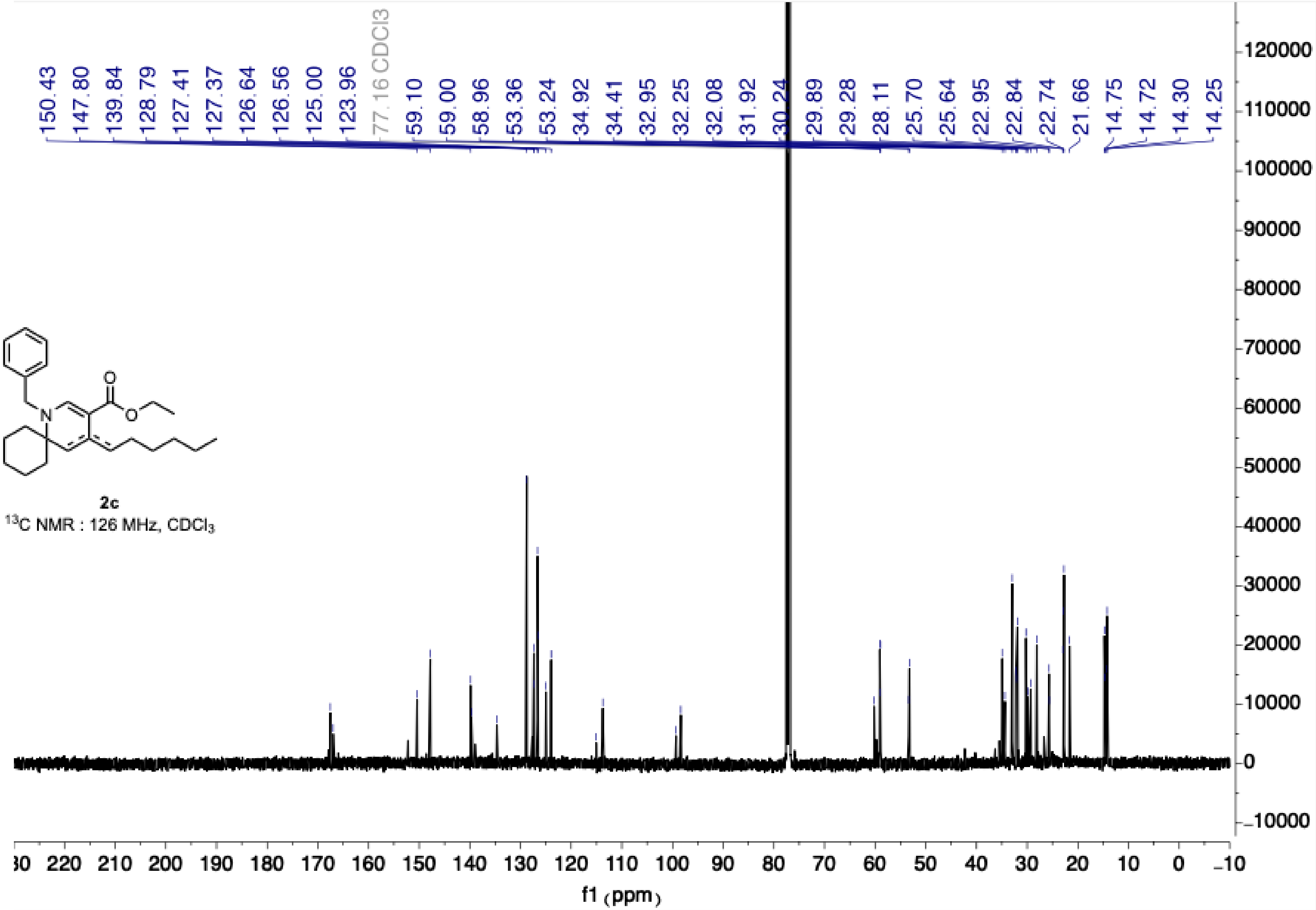
150.43
147.80
139.84
128.79
127.41
127.37
126.64
126.56
125.00
123.96
77.16 CDCl3
59.10
59.00
58.96
53.36
53.24
34.92
34.41
32.95
32.25
32.08
31.92
30.24
29.89
29.28
28.11
25.70
25.64
22.95
22.84
22.74
21.66
14.75
14.72
14.30
14.25
2c
^{13}C NMR : 126 MHz, CDCl$_3$
f1 (ppm)

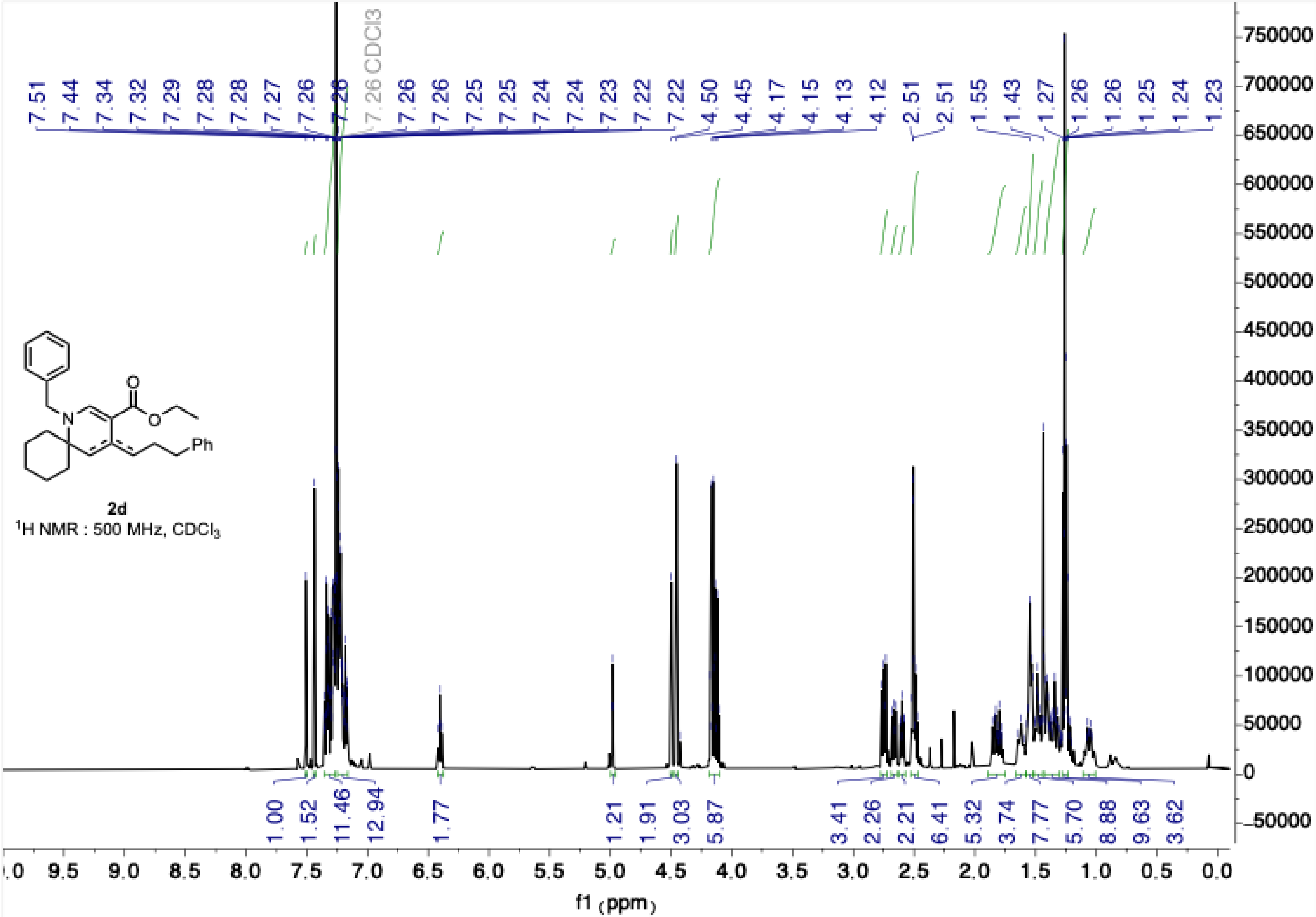
2d
^{1}H NMR : 500 MHz, CDCl$_3$
f1 (ppm)

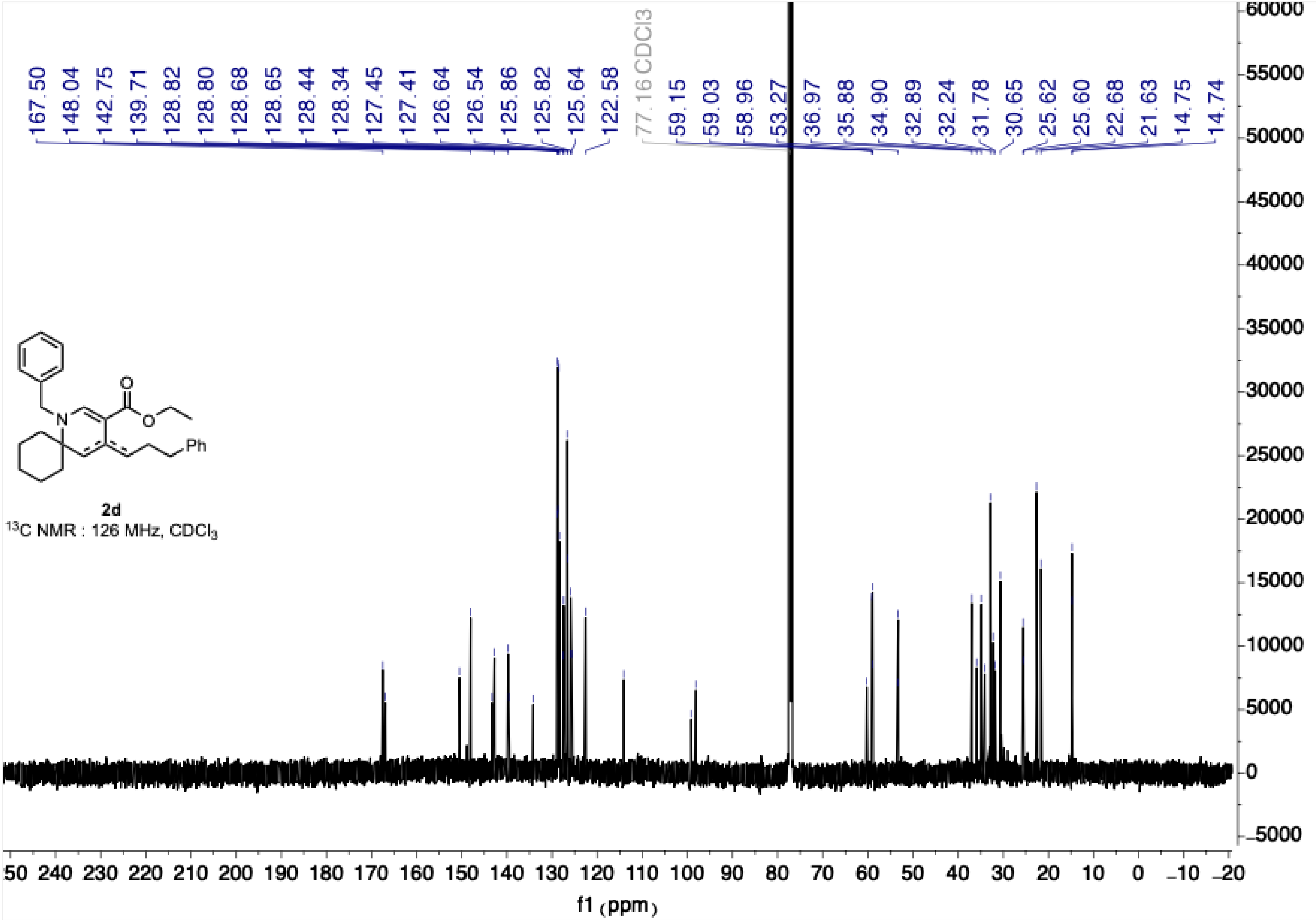
167.50
148.04
142.75
139.71
128.82
128.80
128.68
128.65
128.44
128.34
127.45
127.41
126.64
126.54
125.86
125.82
125.64
122.58
77.16 CDCl3
59.15
59.03
58.96
53.27
36.97
35.88
34.90
32.89
32.24
31.78
30.65
25.62
25.60
22.68
21.63
14.75
14.74
N
O
O
Ph
2d
^{13}C NMR : 126 MHz, $CDCl_3$
f1 (ppm)

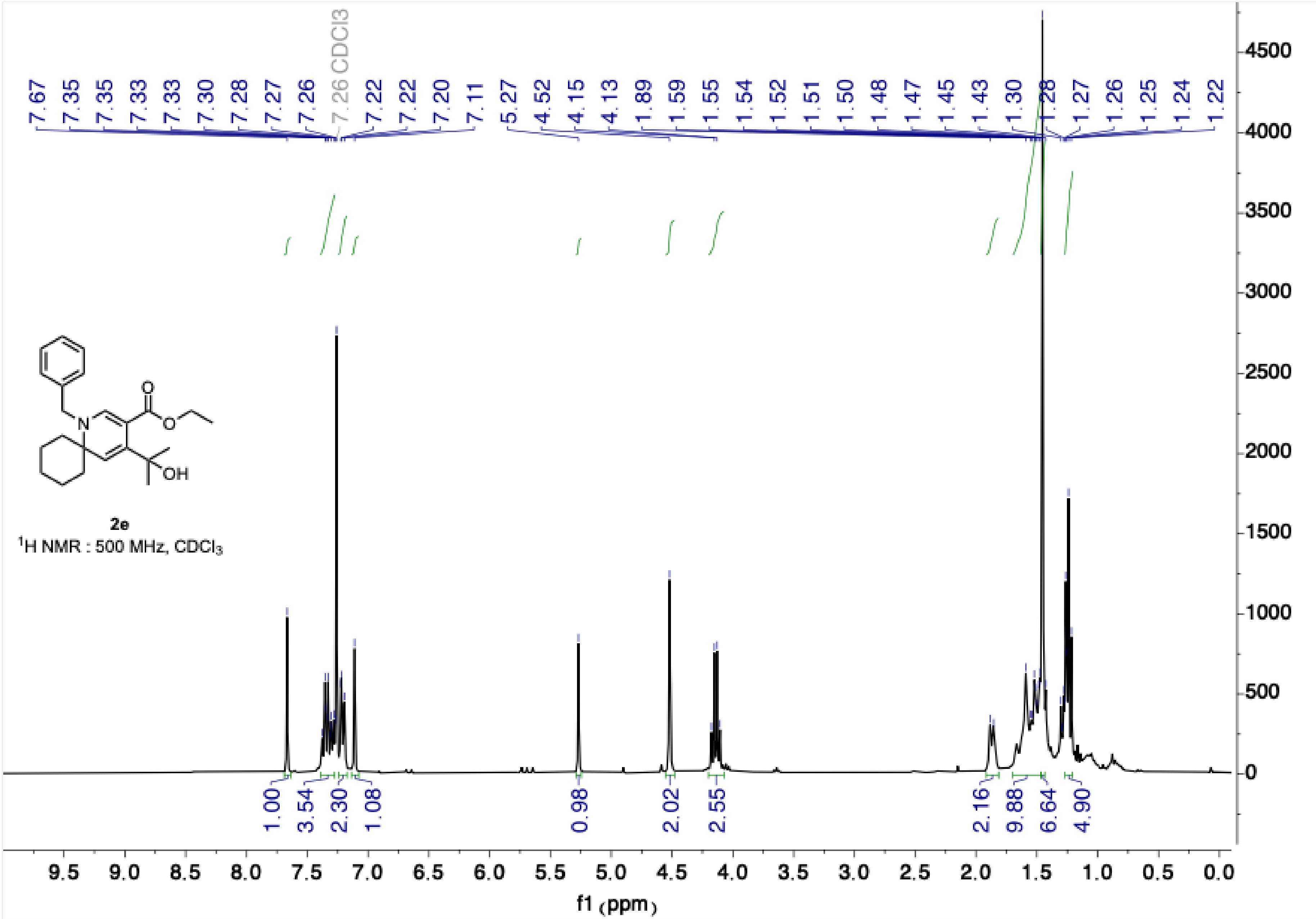
7.67
7.35
7.35
7.33
7.33
7.30
7.28
7.27
7.26
7.26 CDCl3
7.22
7.22
7.20
7.11
5.27
4.52
4.15
4.13
1.89
1.59
1.55
1.54
1.52
1.51
1.50
1.48
1.47
1.45
1.43
1.30
1.28
1.27
1.26
1.25
1.24
1.22
2e
1H NMR : 500 MHz, CDCl3
1.00
3.54
2.30
1.08
0.98
2.02
2.55
2.16
9.88
6.64
4.90
f1 (ppm)

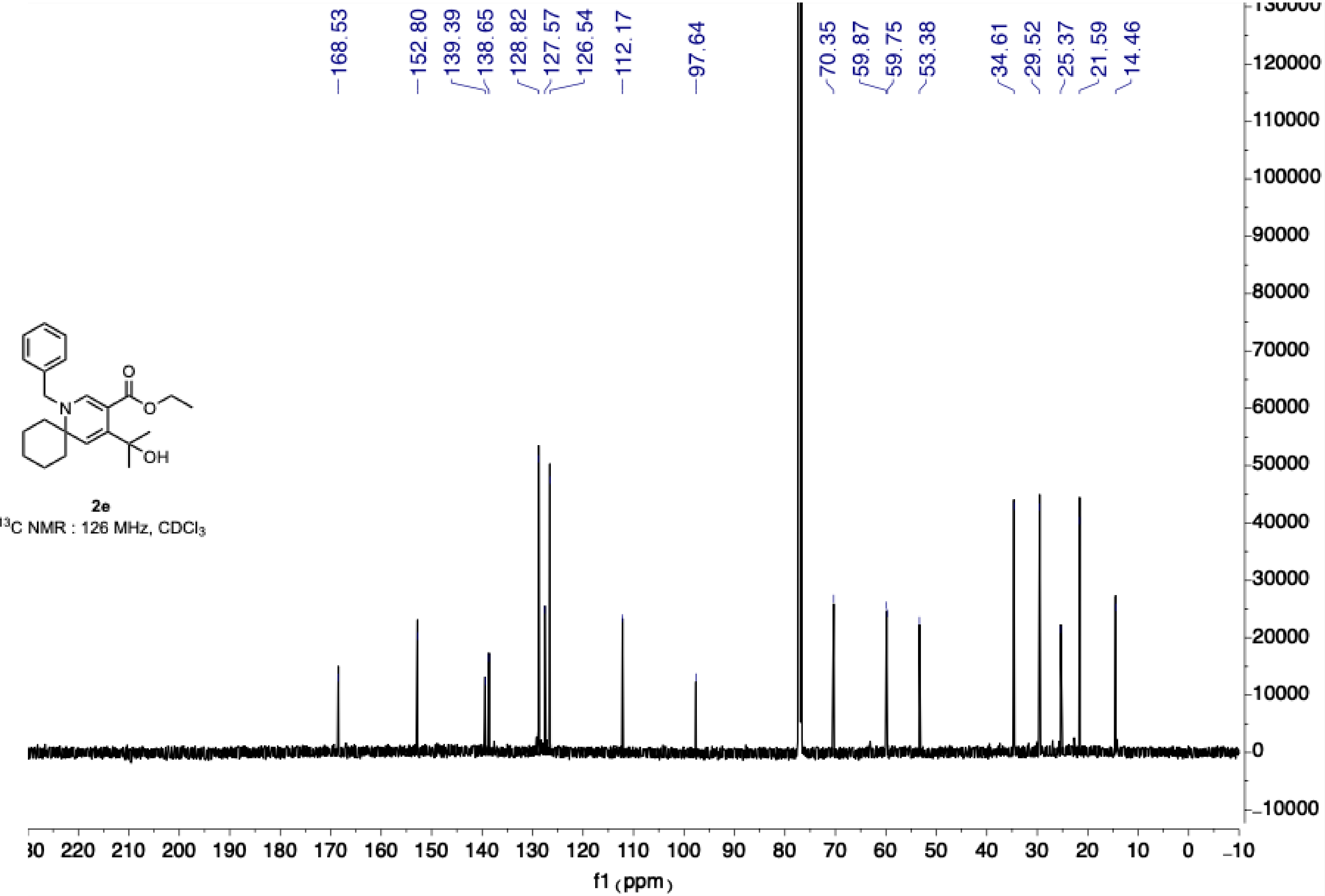

2e

^{13}C NMR : 126 MHz, $CDCl_3$

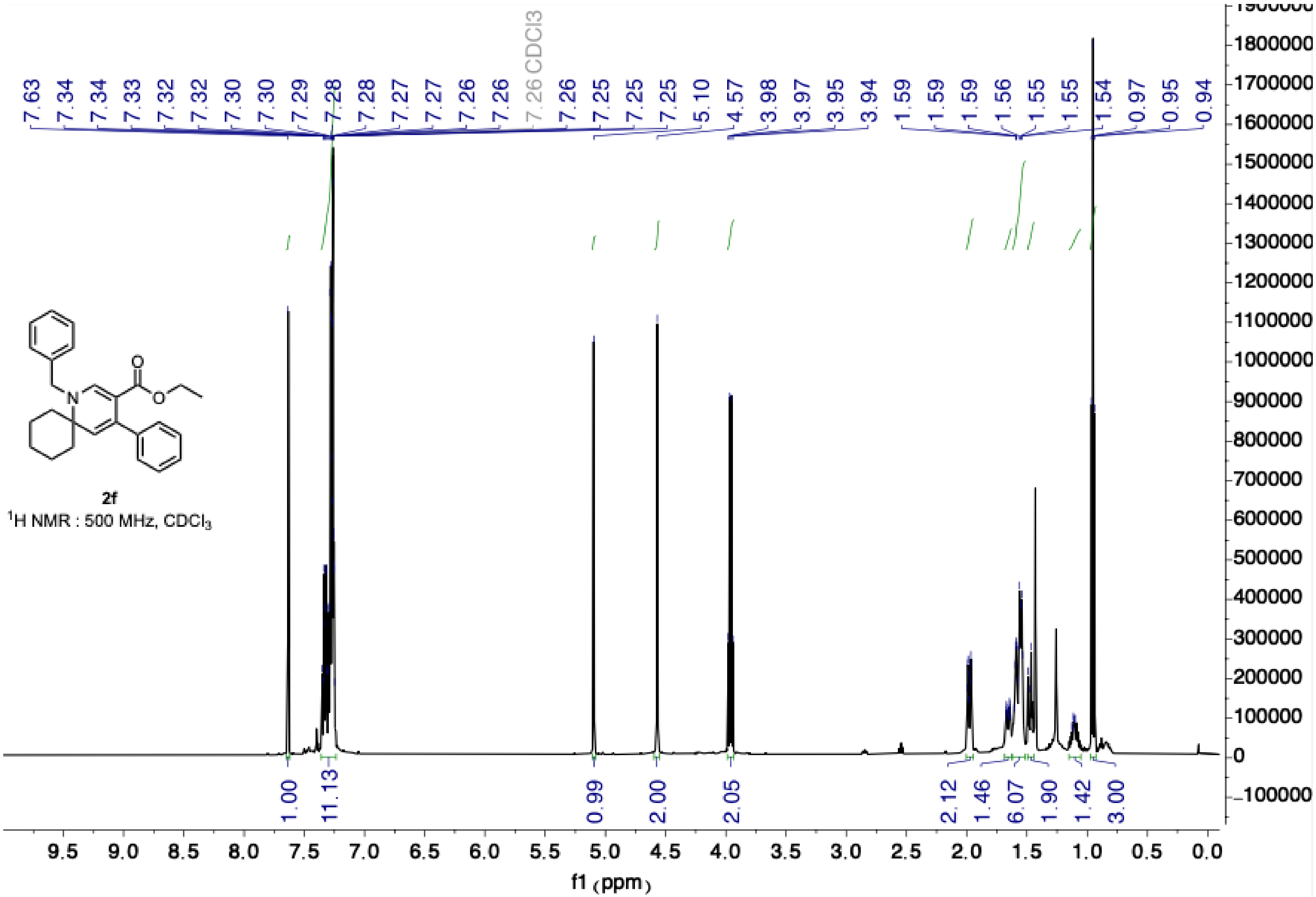
7.63 7.34 7.34 7.33 7.32 7.32 7.30 7.30 7.29 7.28 7.28 7.27 7.27 7.26 7.26 7.26 CDCl3 7.26 7.25 7.25 7.25 5.10 4.57 3.98 3.97 3.95 3.94 1.59 1.59 1.59 1.56 1.55 1.55 1.54 0.97 0.95 0.94
2f
^{1}H NMR : 500 MHz, CDCl$_3$
1.00 11.13 0.99 2.00 2.05 2.12 1.46 6.07 1.90 1.42 3.00
f1 (ppm)

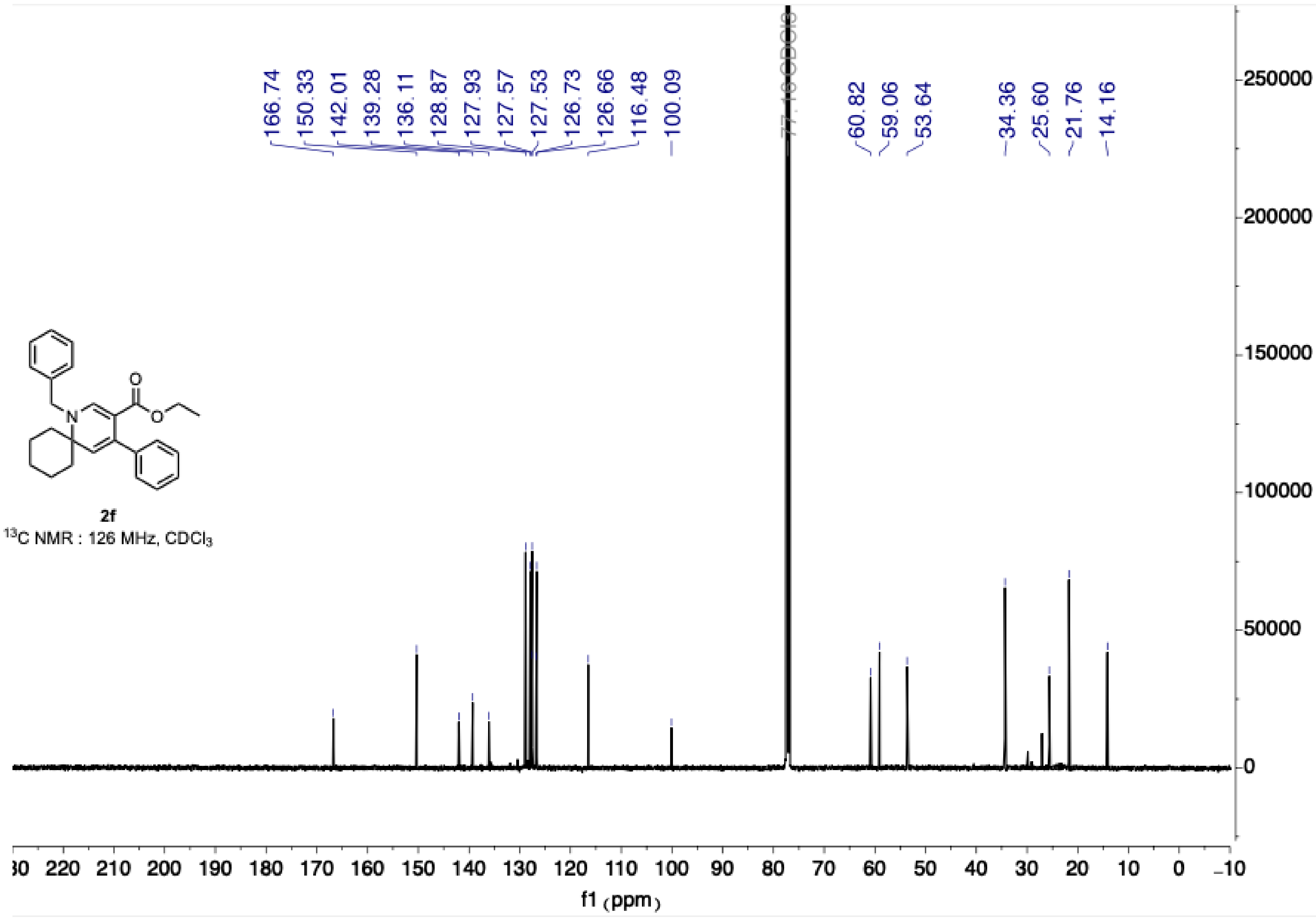
166.74
150.33
142.01
139.28
136.11
128.87
127.93
127.57
127.53
126.73
126.66
116.48
100.09
77.16 CDCl3
60.82
59.06
53.64
34.36
25.60
21.76
14.16
2f
^{13}C NMR : 126 MHz, $CDCl_3$
f1 (ppm)

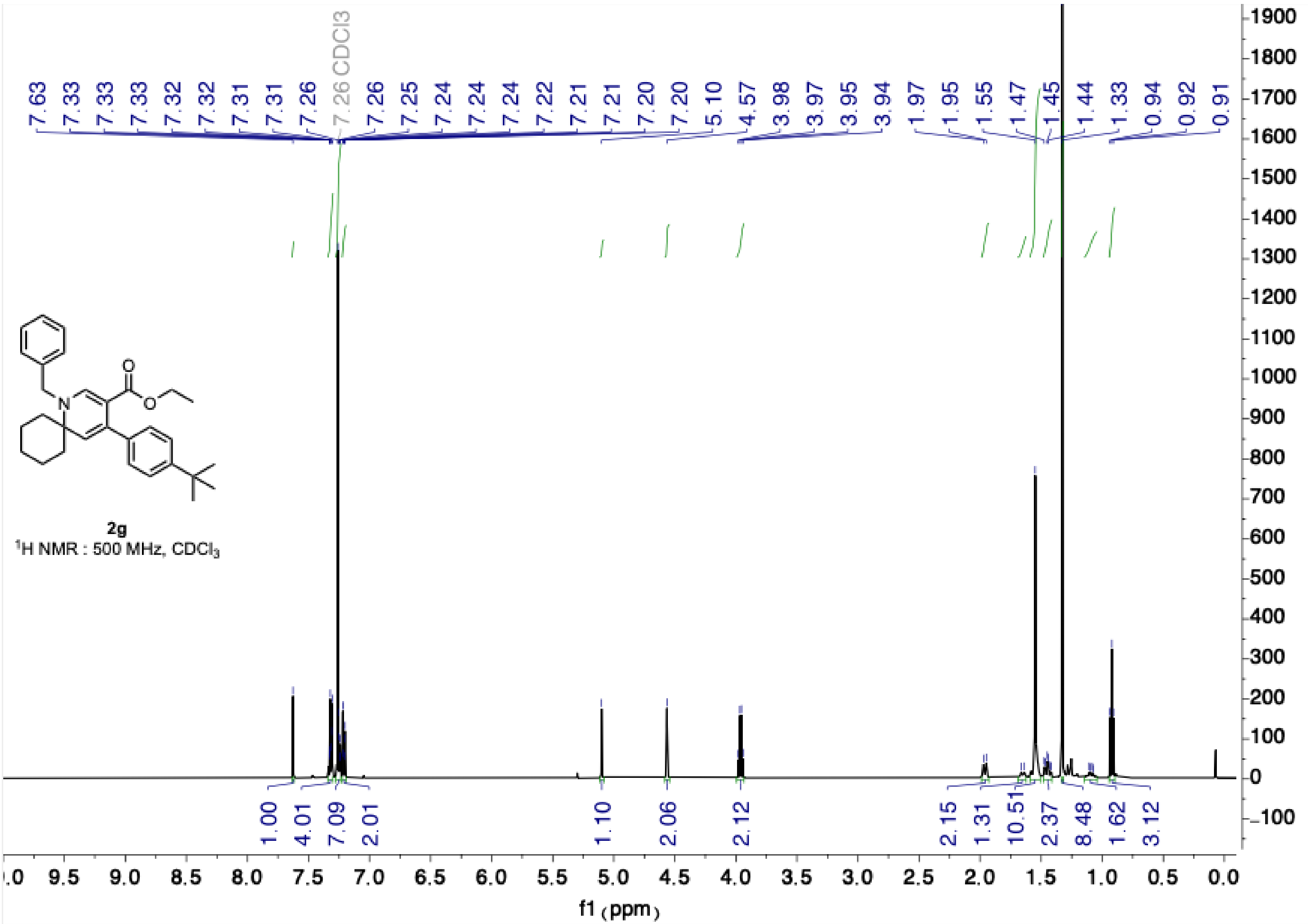
7.63 7.33 7.33 7.33 7.32 7.32 7.31 7.31 7.26 7.26 CDCl3 7.26 7.25 7.24 7.24 7.24 7.22 7.21 7.21 7.20 7.20 5.10 4.57 3.98 3.97 3.95 3.94 1.97 1.95 1.55 1.47 1.45 1.44 1.33 0.94 0.92 0.91
1.00 4.01 7.09 2.01 1.10 2.06 2.12 2.15 1.31 10.51 2.37 8.48 1.62 3.12
2g
^{1}H NMR : 500 MHz, CDCl$_3$
f1 (ppm)

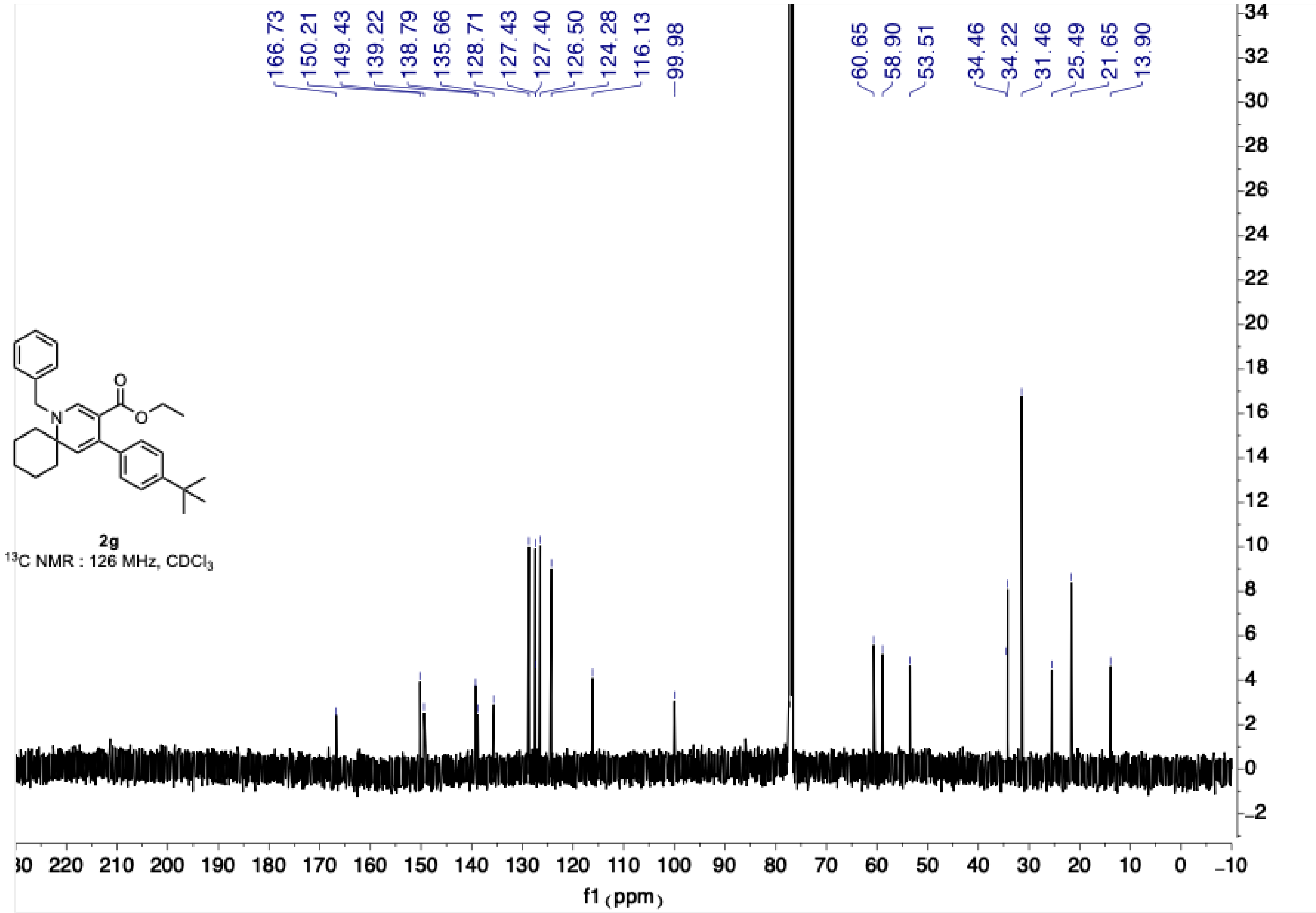
166.73
150.21
149.43
139.22
138.79
135.66
128.71
127.43
127.40
126.50
124.28
116.13
99.98
60.65
58.90
53.51
34.46
34.22
31.46
25.49
21.65
13.90
2g
^{13}C NMR : 126 MHz, CDCl$_3$
f1 (ppm)

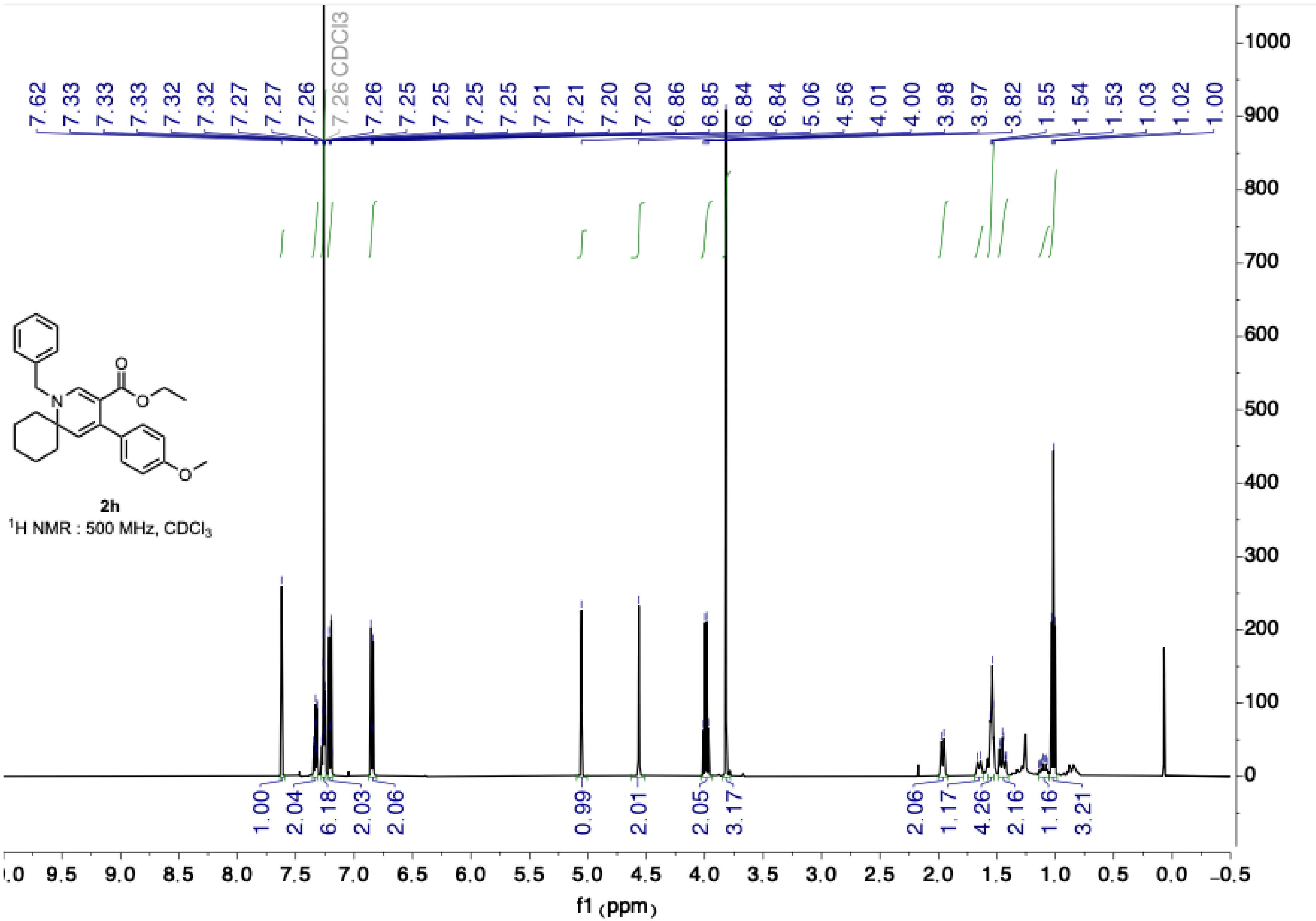

2h

^{1}H NMR : 500 MHz, $CDCl_3$

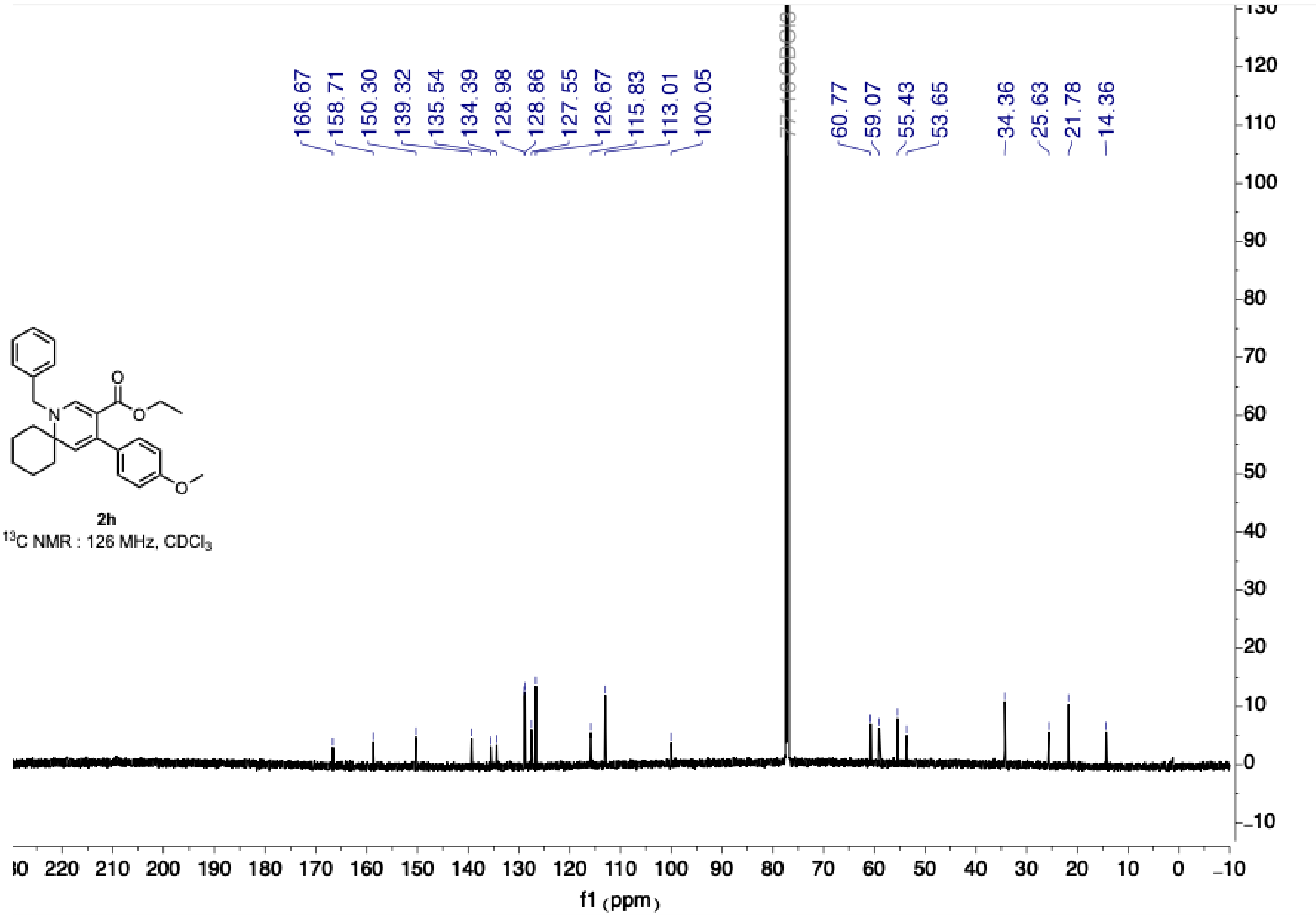

2h

^{13}C NMR : 126 MHz, $CDCl_3$

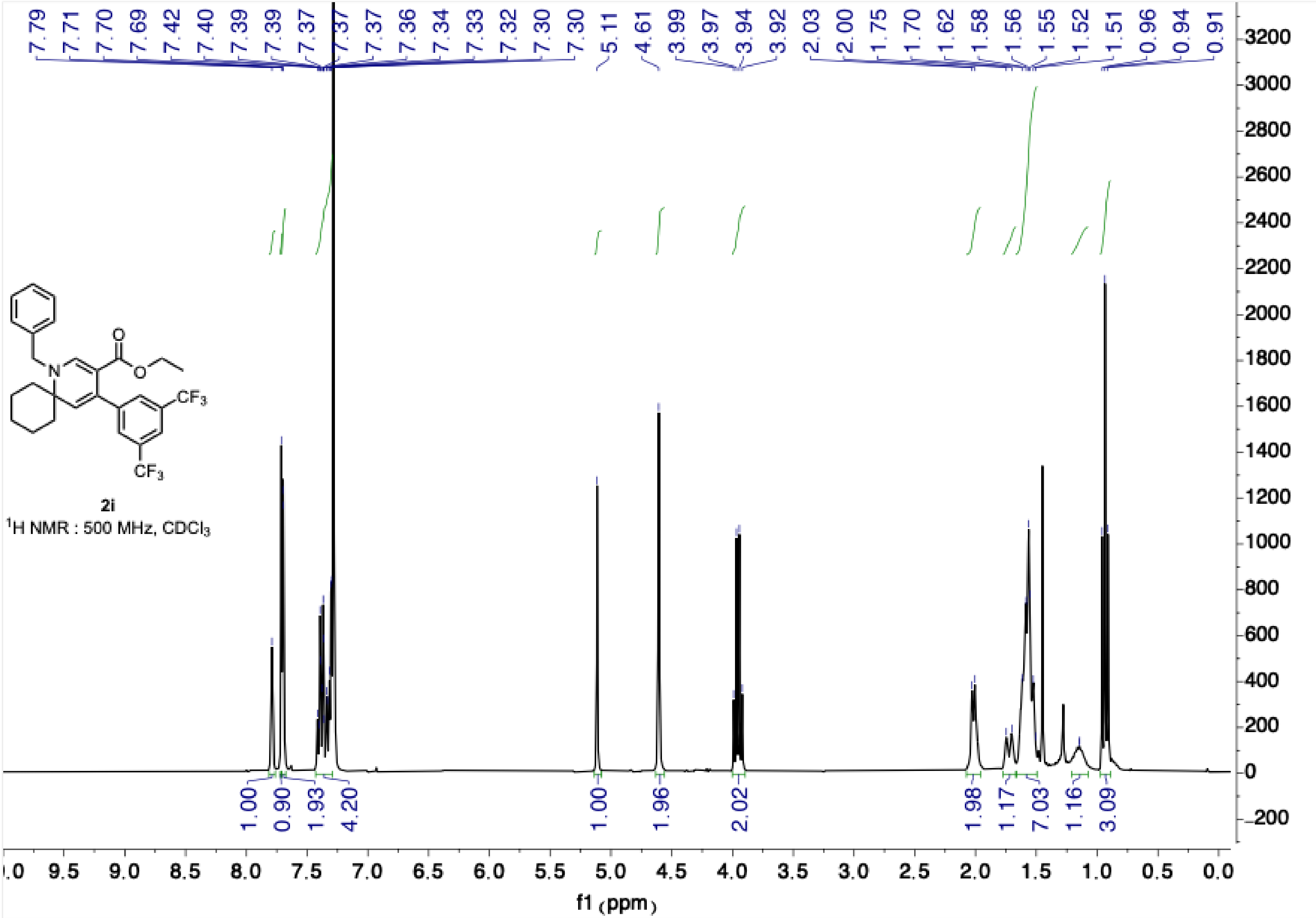

2i
^{1}H NMR : 500 MHz, CDCl3
7.79 7.71 7.70 7.69 7.42 7.40 7.39 7.39 7.37 7.37 7.37 7.36 7.34 7.33 7.32 7.30 7.30 5.11 4.61 3.99 3.97 3.94 3.92 2.03 2.00 1.75 1.70 1.62 1.58 1.56 1.55 1.52 1.51 0.96 0.94 0.91
1.00 0.90 1.93 4.20 1.00 1.96 2.02 1.98 1.17 7.03 1.16 3.09
f1 (ppm)

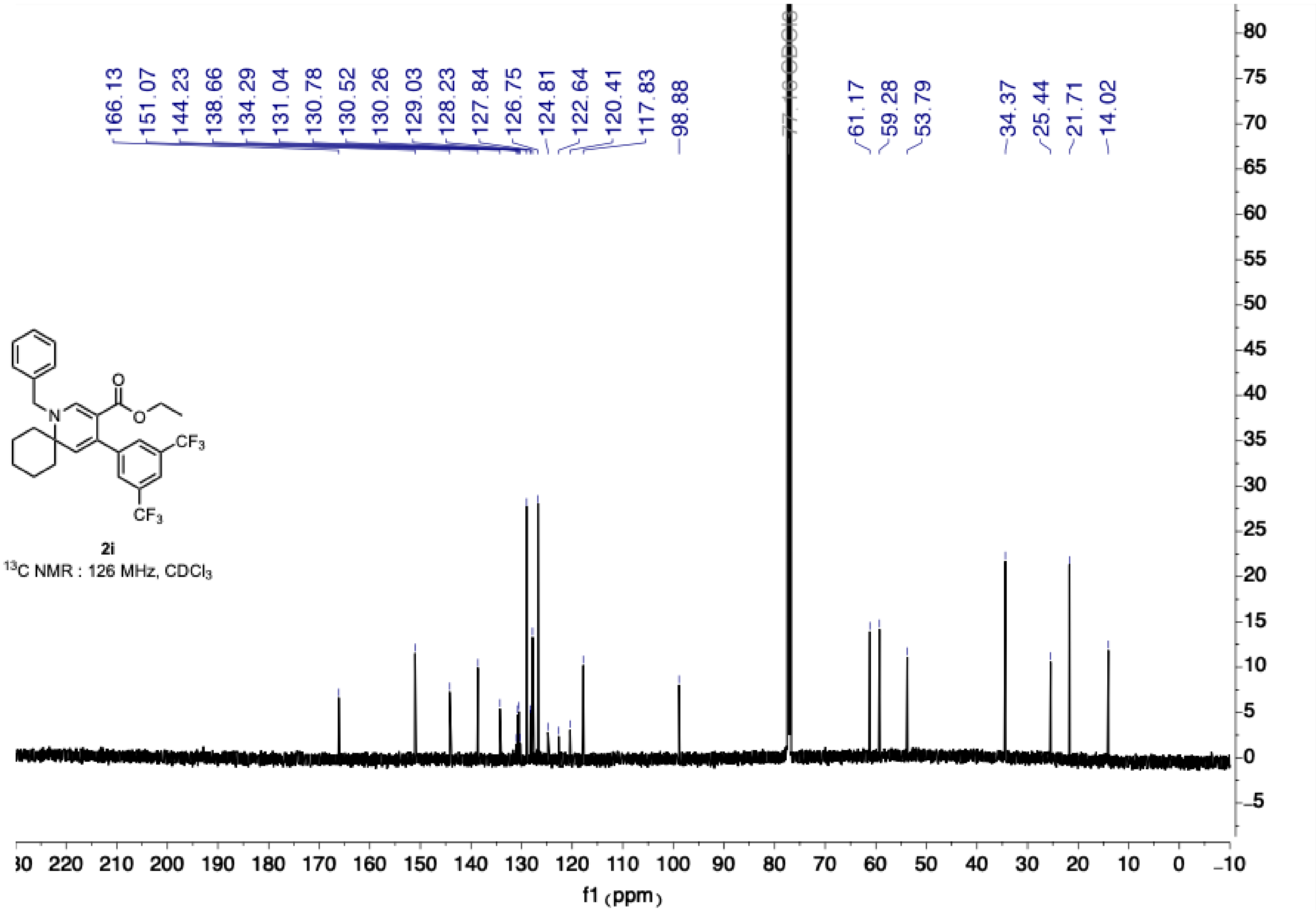
166.13
151.07
144.23
138.66
134.29
131.04
130.78
130.52
130.26
129.03
128.23
127.84
126.75
124.81
122.64
120.41
117.83
98.88
77.16 CDCl3
61.17
59.28
53.79
34.37
25.44
21.71
14.02
2i
13C NMR : 126 MHz, CDCl3
f1 (ppm)

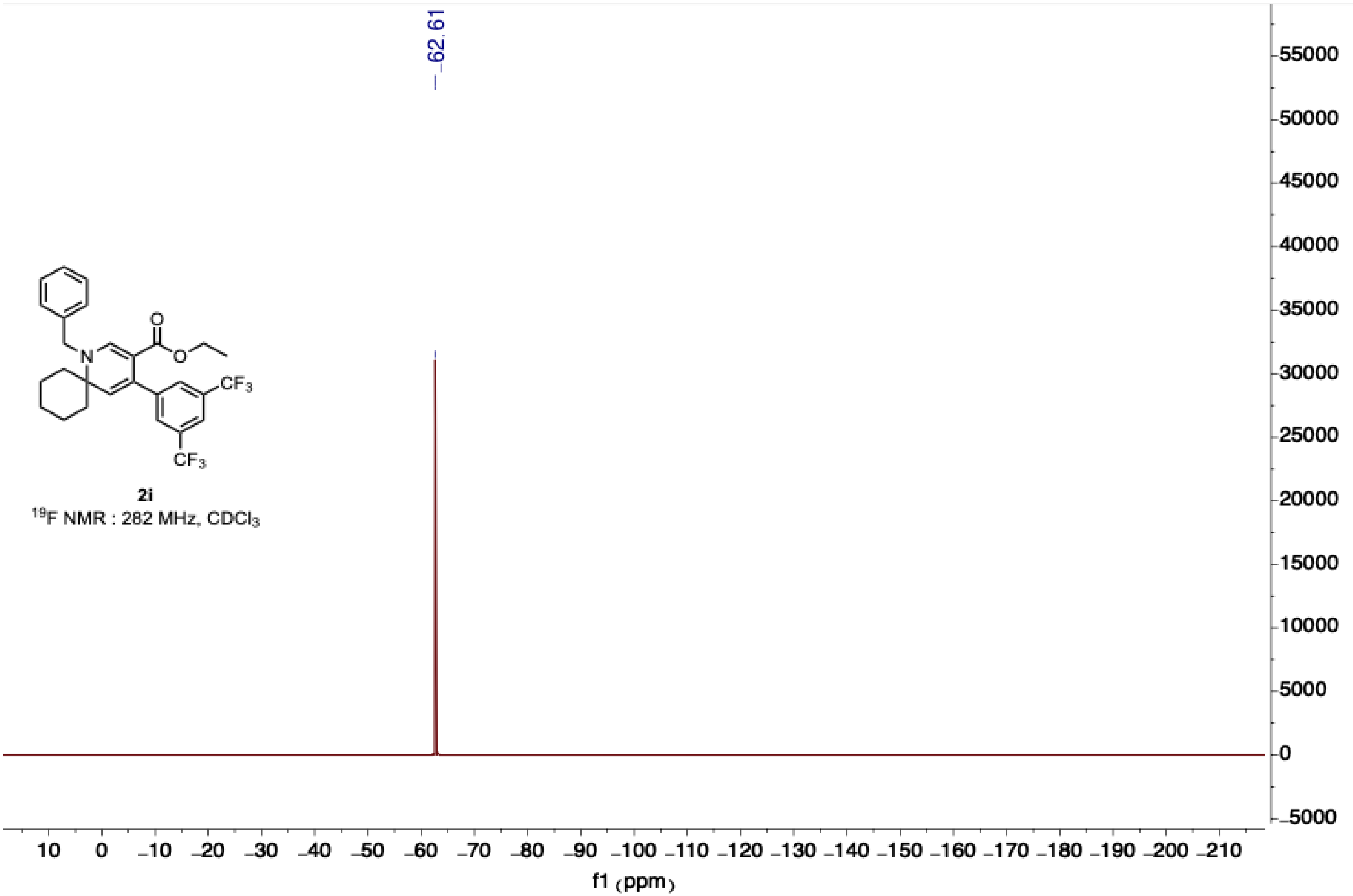

-62.61
2i
19F NMR : 282 MHz, CDCl3
CF3
CF3
N
O
O
55000
50000
45000
40000
35000
30000
25000
20000
15000
10000
5000
0
-5000
10 0 -10 -20 -30 -40 -50 -60 -70 -80 -90 -100 -110 -120 -130 -140 -150 -160 -170 -180 -190 -200 -210
f1 (ppm)

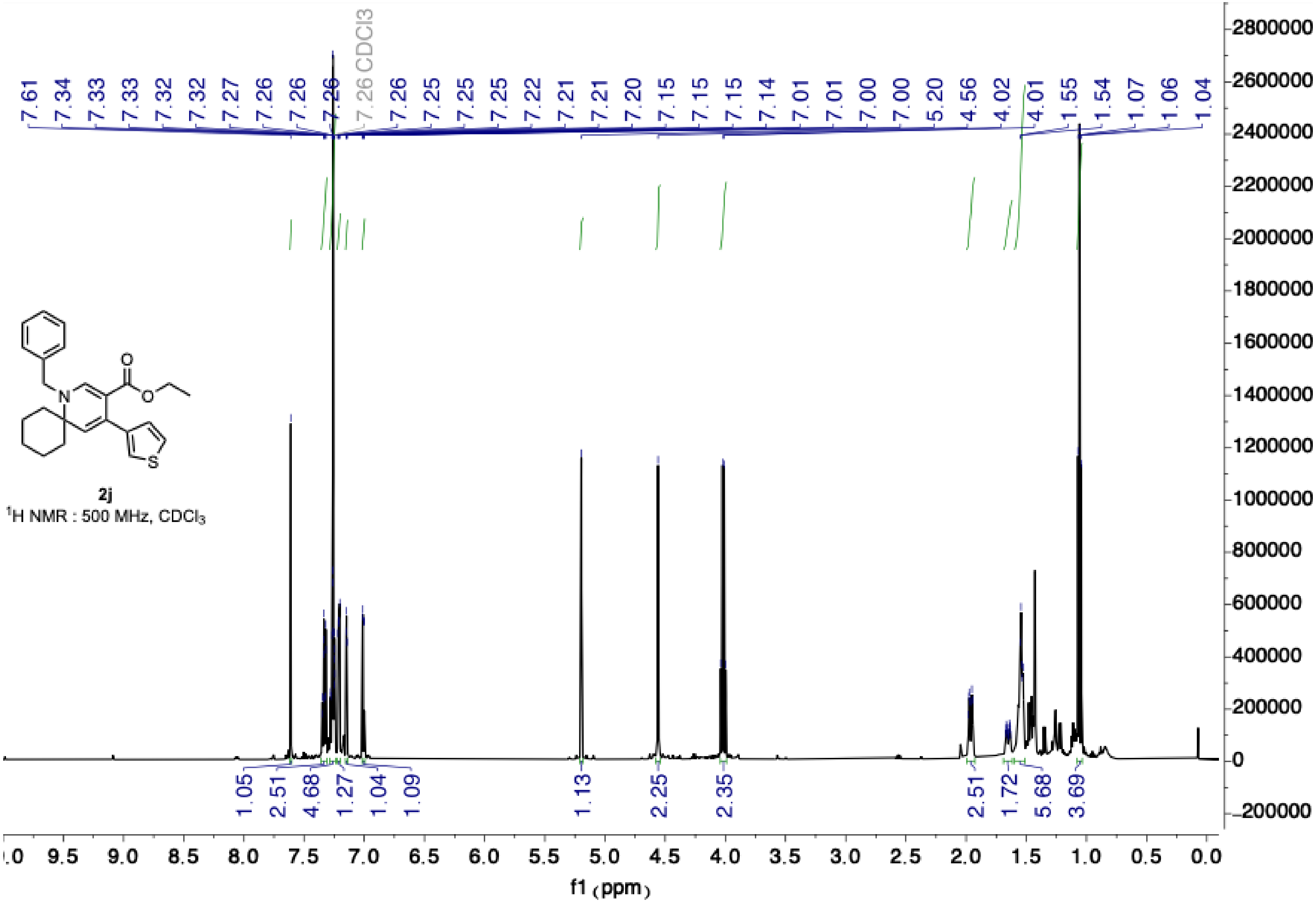
2j
1H NMR : 500 MHz, CDCl3
f1 (ppm)

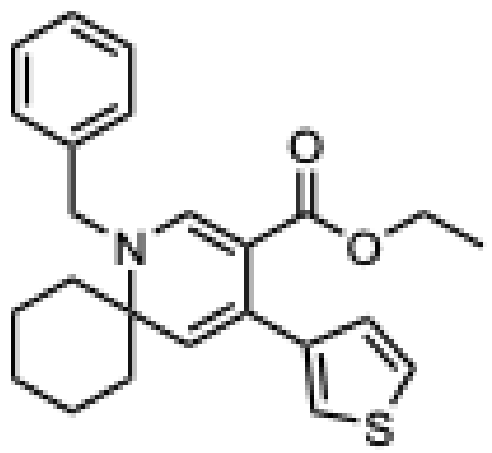

2j

^{13}C NMR : 126 MHz, $CDCl_3$

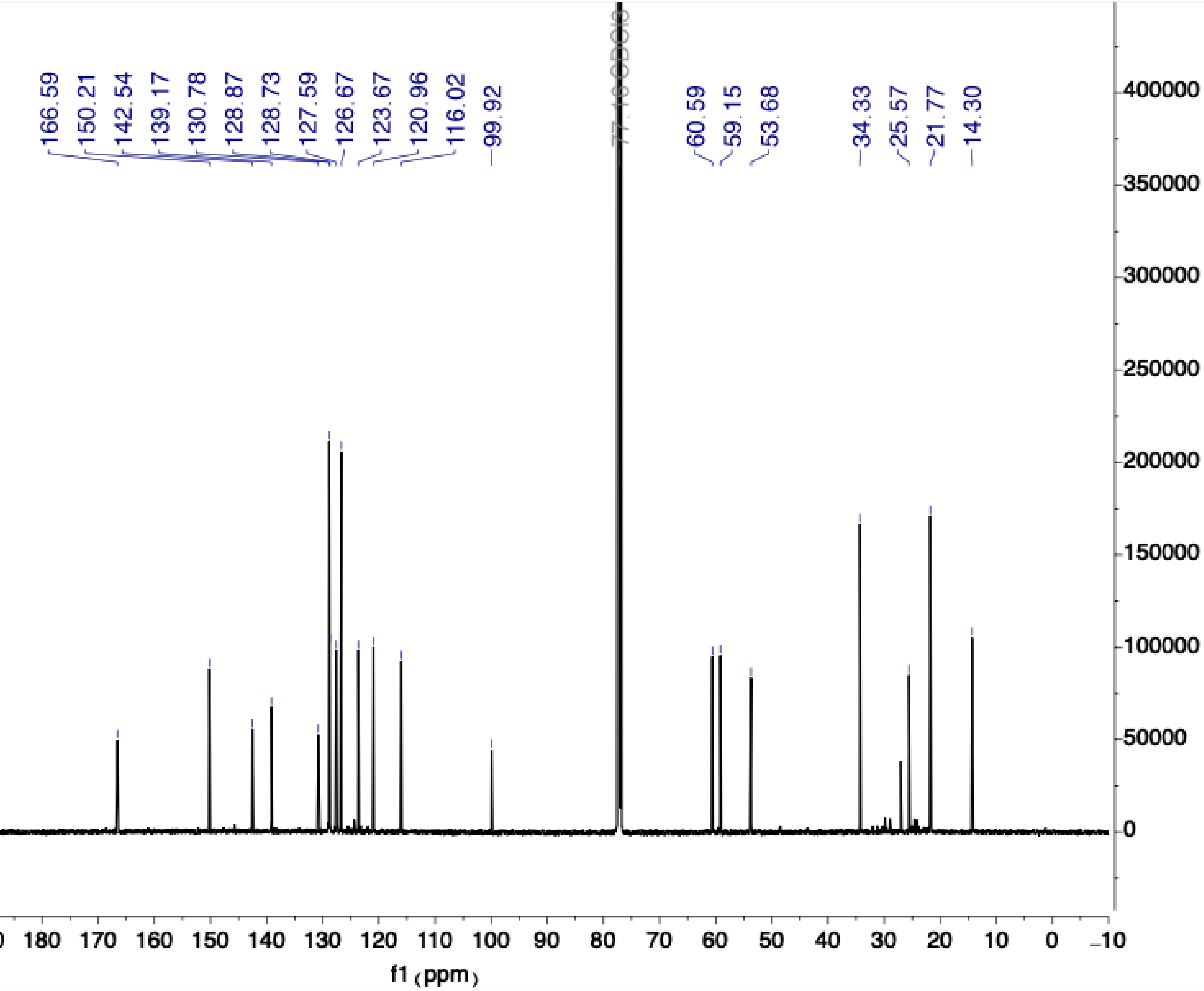

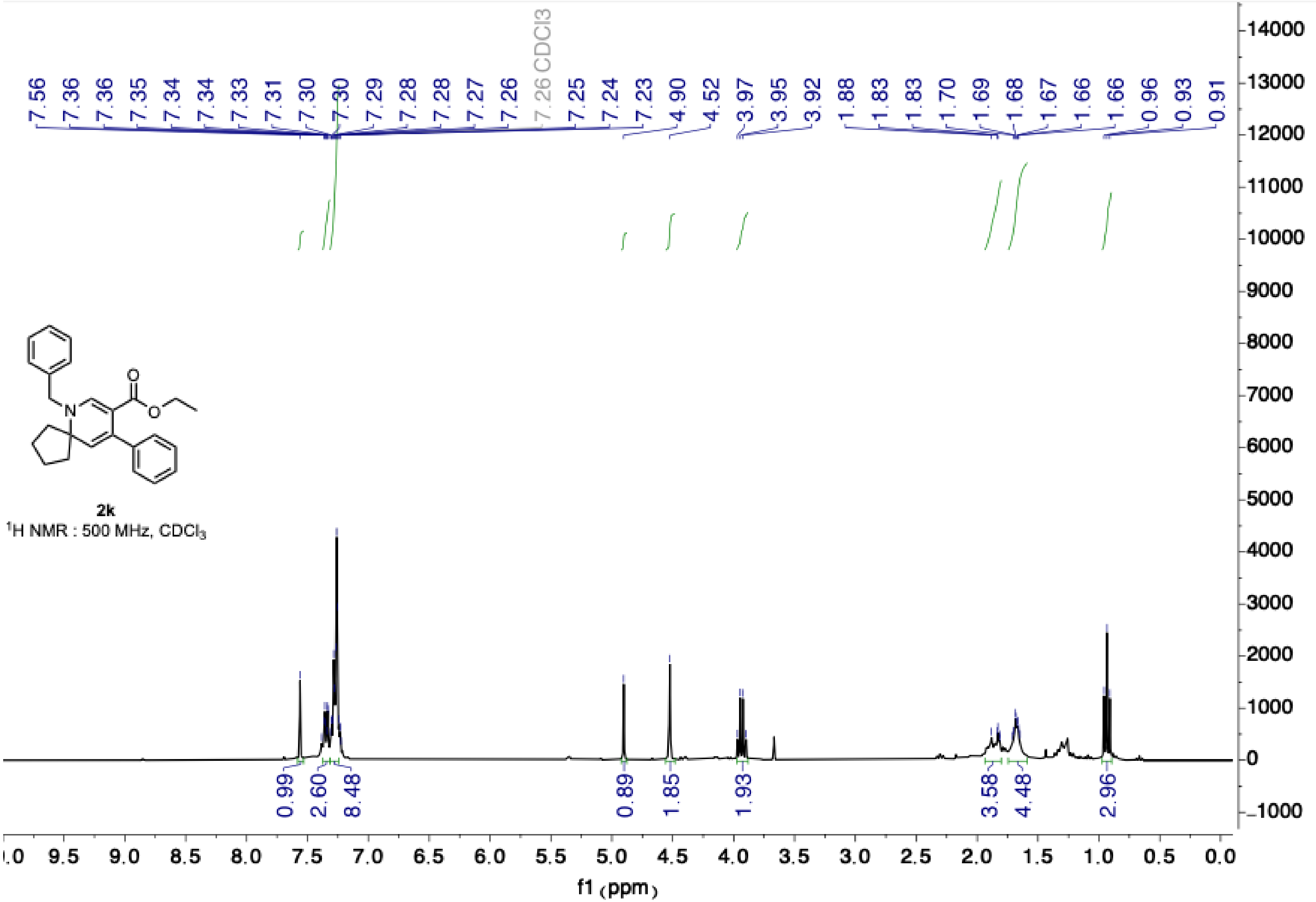

2k

^{1}H NMR : 500 MHz, $CDCl_3$

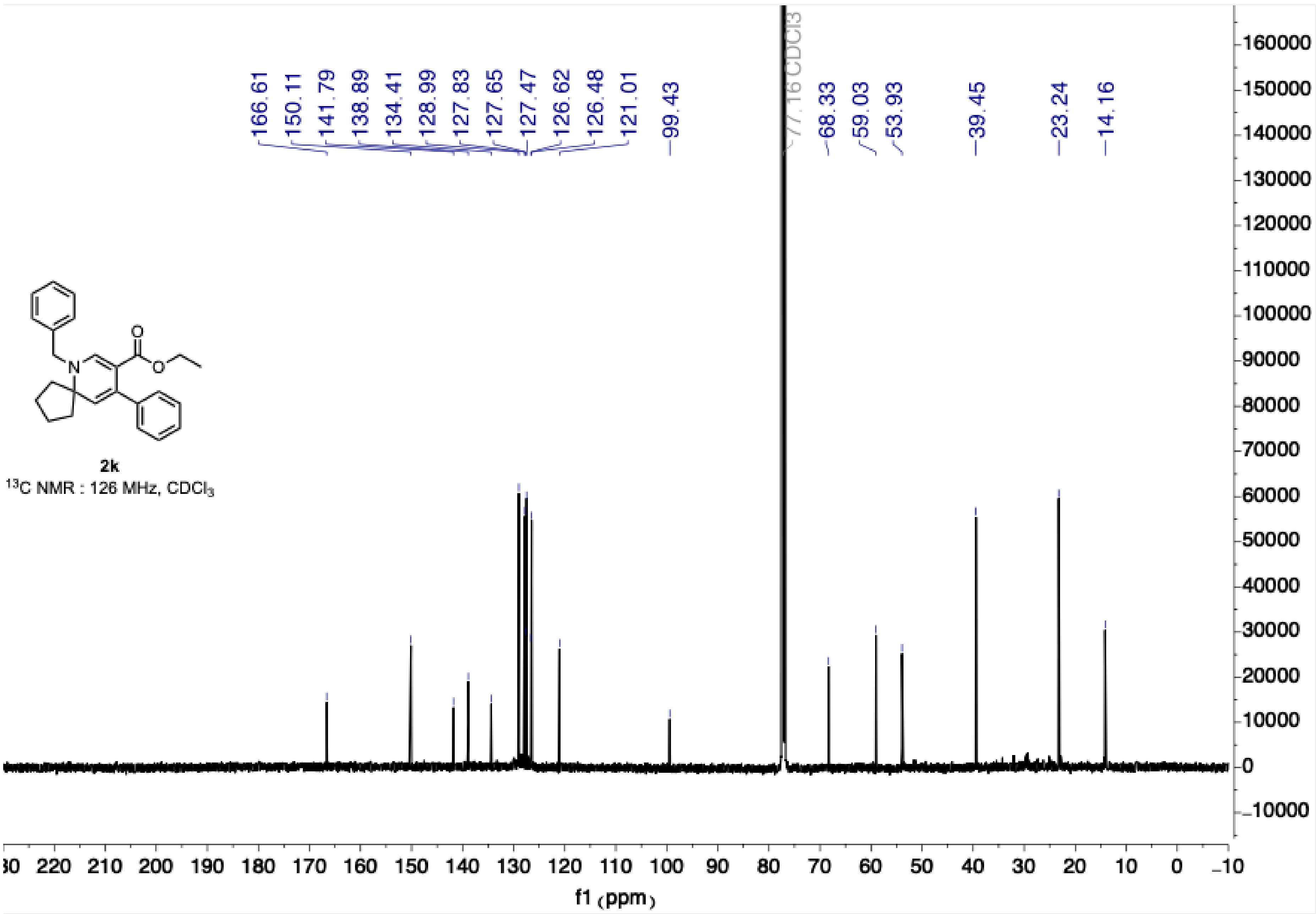

166.61
150.11
141.79
138.89
134.41
128.99
127.83
127.65
127.47
126.62
126.48
121.01
99.43
77.16 CDCl3
68.33
59.03
53.93
39.45
23.24
14.16
2k
13C NMR : 126 MHz, CDCl3
f1 (ppm)

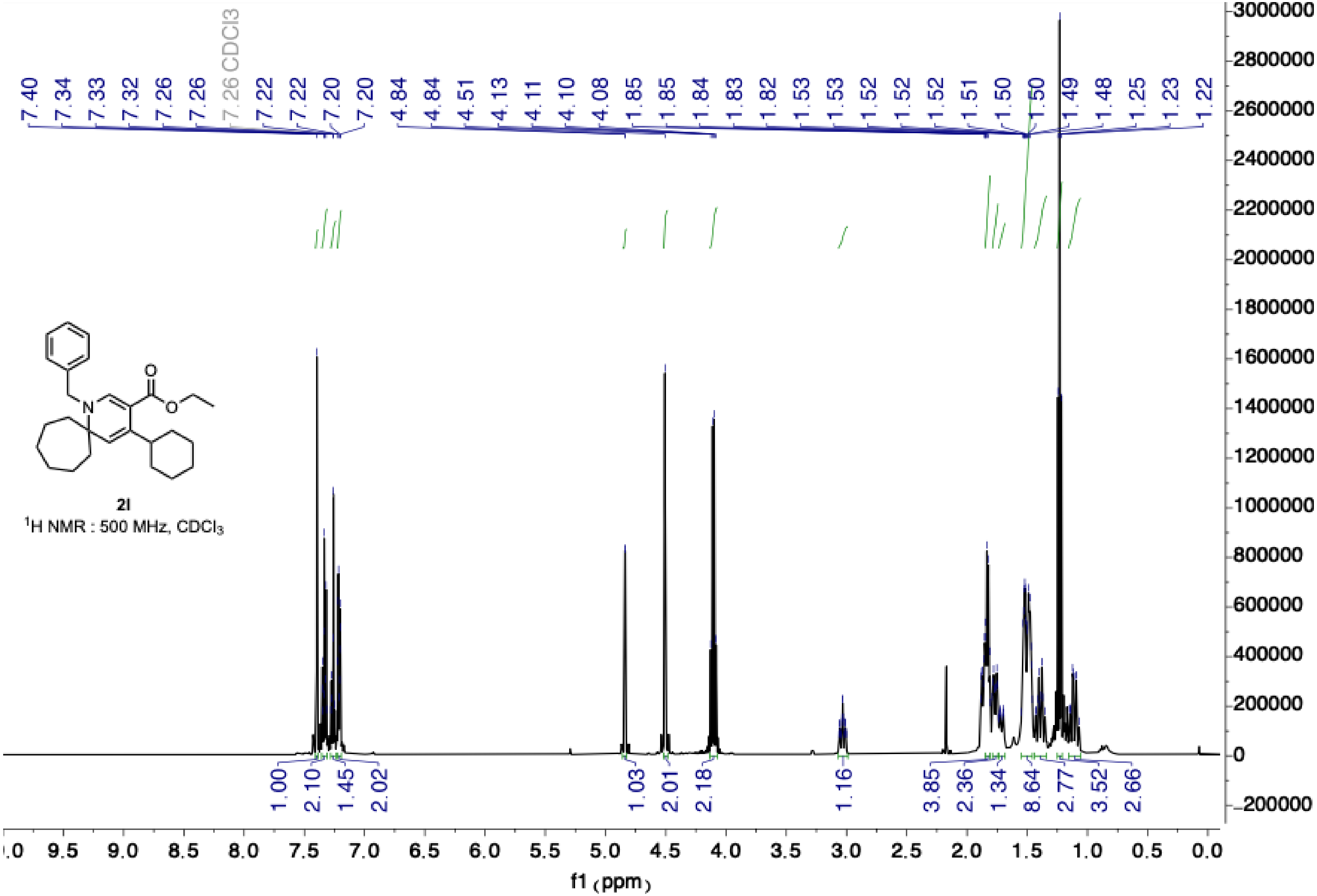

2l
^{1}H NMR : 500 MHz, CDCl$_3$
f1 (ppm)

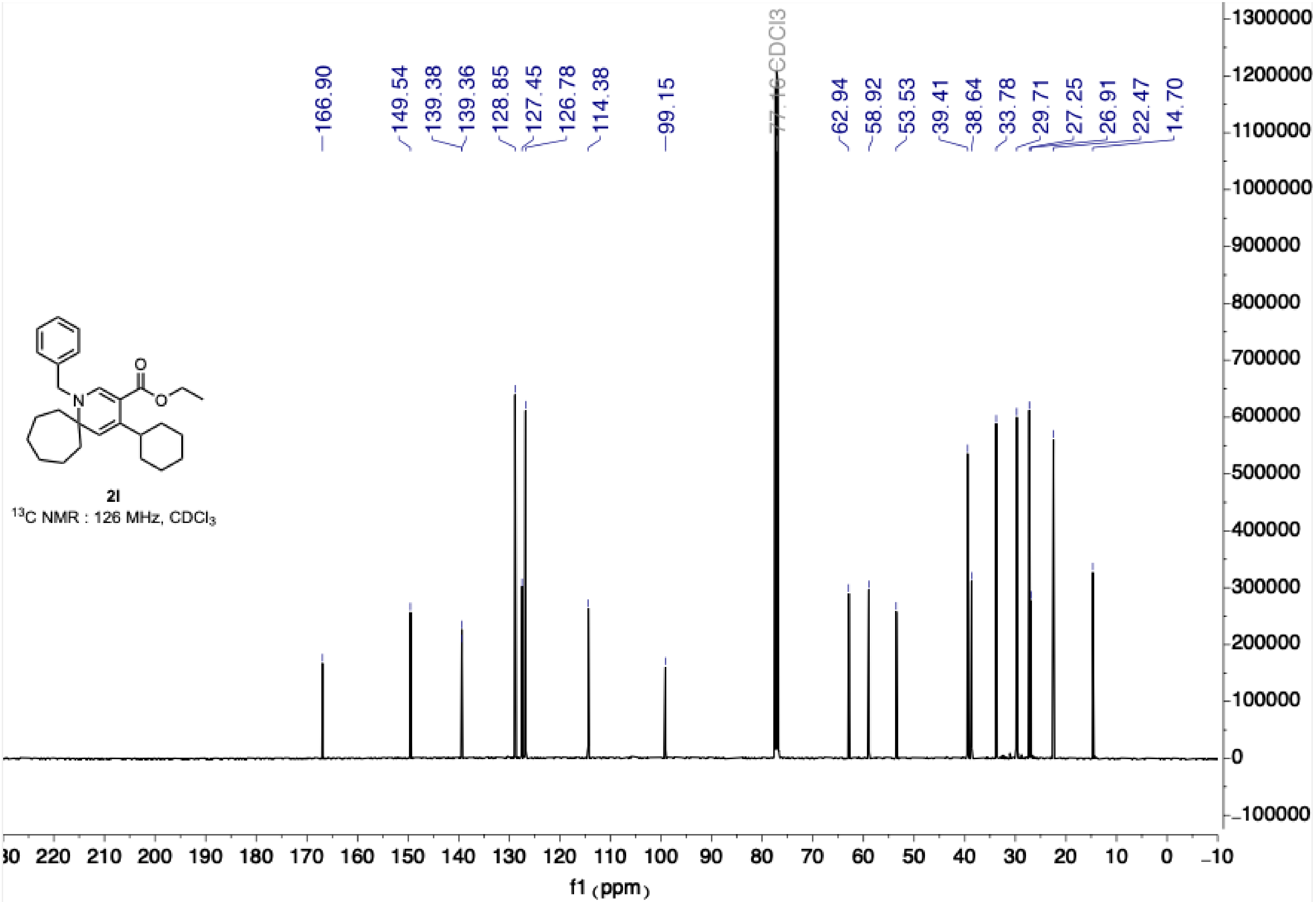
166.90
149.54
139.38
139.36
128.85
127.45
126.78
114.38
99.15
77.16 CDCl3
62.94
58.92
53.53
39.41
38.64
33.78
29.71
27.25
26.91
22.47
14.70
2l
^{13}C NMR : 126 MHz, CDCl$_3$
f1 (ppm)

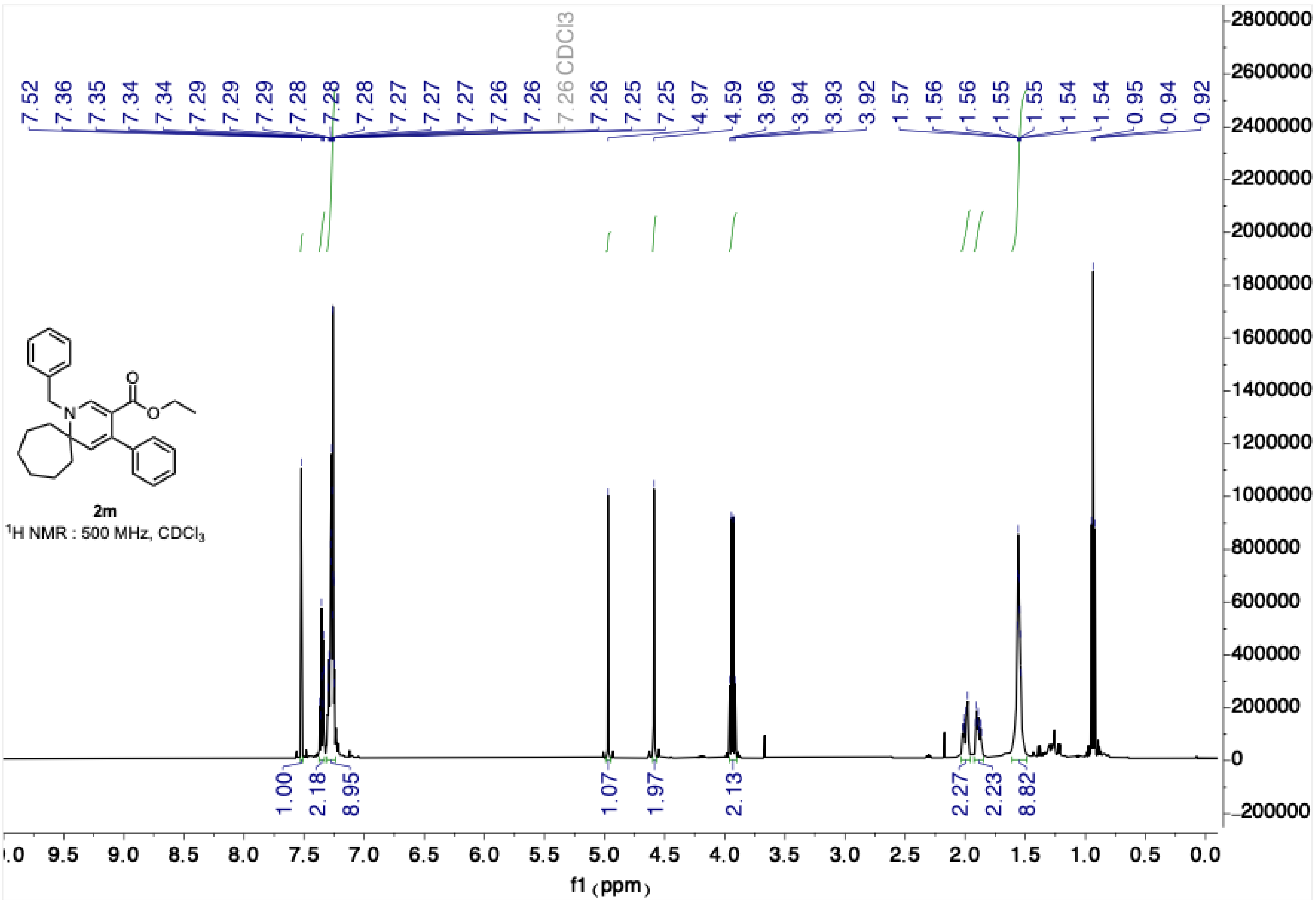

2m
¹H NMR : 500 MHz, CDCl3
7.52 7.36 7.35 7.34 7.34 7.29 7.29 7.29 7.28 7.28 7.28 7.27 7.27 7.27 7.26 7.26 7.26 CDCl3 7.26 7.25 7.25 4.97 4.59 3.96 3.94 3.93 3.92 1.57 1.56 1.56 1.55 1.55 1.54 1.54 0.95 0.94 0.92
1.00 2.18 8.95 1.07 1.97 2.13 2.27 2.23 8.82
f1 (ppm)

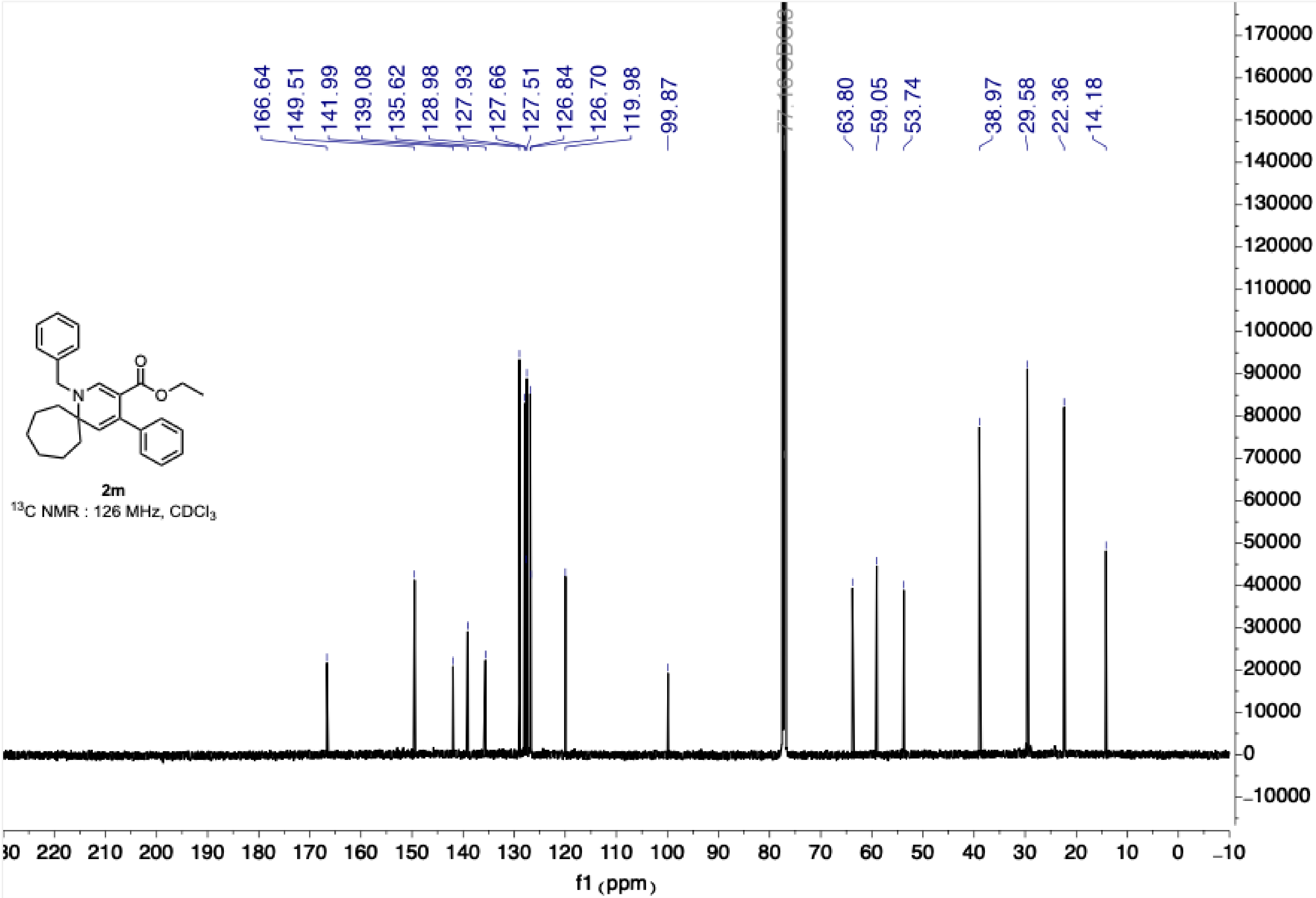
166.64
149.51
141.99
139.08
135.62
128.98
127.93
127.66
127.51
126.84
126.70
119.98
99.87
77.16 CDCl3
63.80
59.05
53.74
38.97
29.58
22.36
14.18
N
O
O
2m
13C NMR : 126 MHz, CDCl3
f1 (ppm)

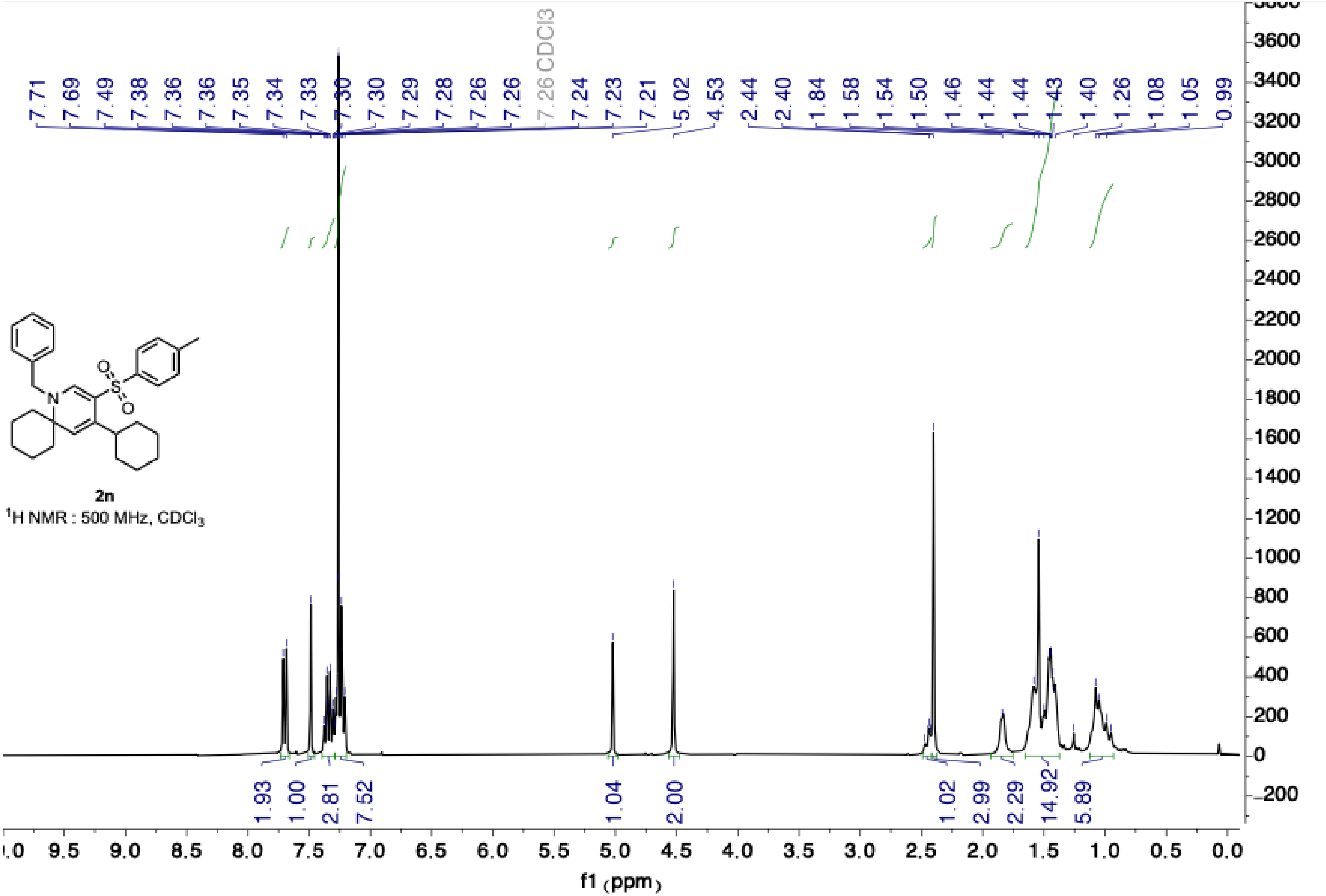

2n
^{1}H NMR : 500 MHz, $CDCl_3$

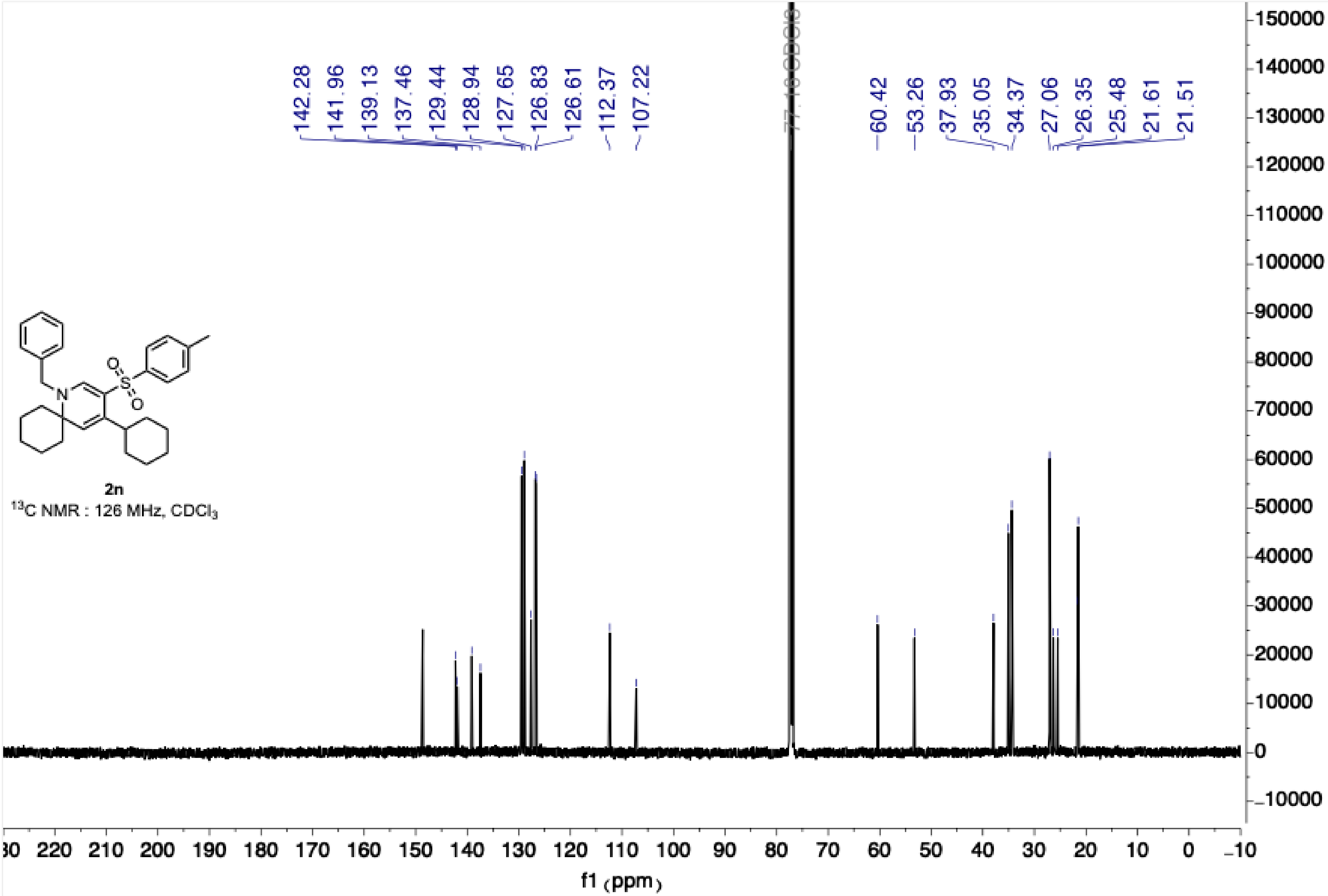
142.28
141.96
139.13
137.46
129.44
128.94
127.65
126.83
126.61
112.37
107.22
60.42
53.26
37.93
35.05
34.37
27.06
26.35
25.48
21.61
21.51
2n
^{13}C NMR : 126 MHz, CDCl$_3$
f1 (ppm)

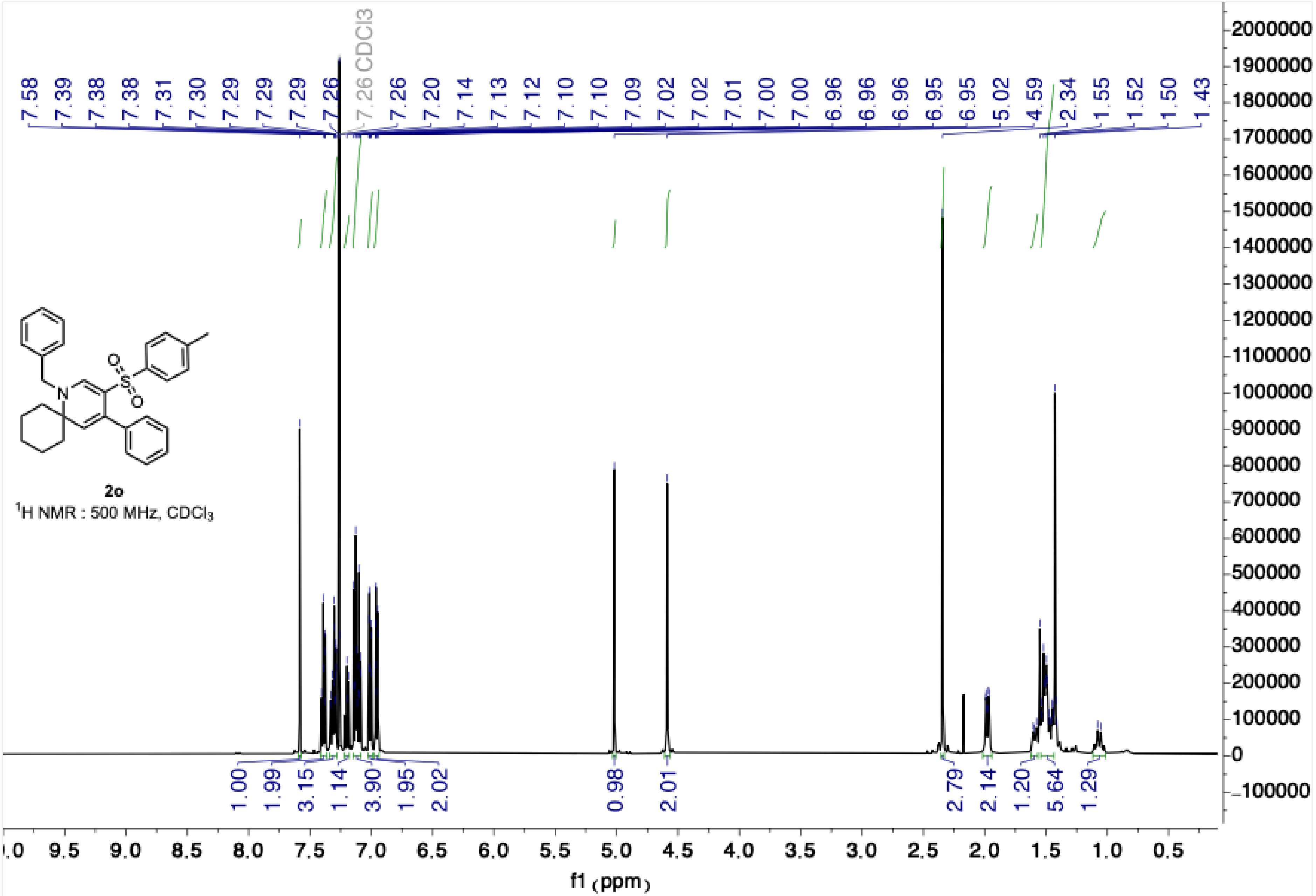
7.58
7.39
7.38
7.38
7.31
7.30
7.29
7.29
7.29
7.26
7.26 CDCl3
7.26
7.20
7.14
7.13
7.12
7.10
7.10
7.09
7.02
7.02
7.01
7.00
7.00
6.96
6.96
6.96
6.95
6.95
5.02
4.59
2.34
1.55
1.52
1.50
1.43
N
O
S
O
2o
1H NMR : 500 MHz, CDCl3
1.00
1.99
3.15
1.14
3.90
1.95
2.02
0.98
2.01
2.79
2.14
1.20
5.64
1.29
f1 (ppm)

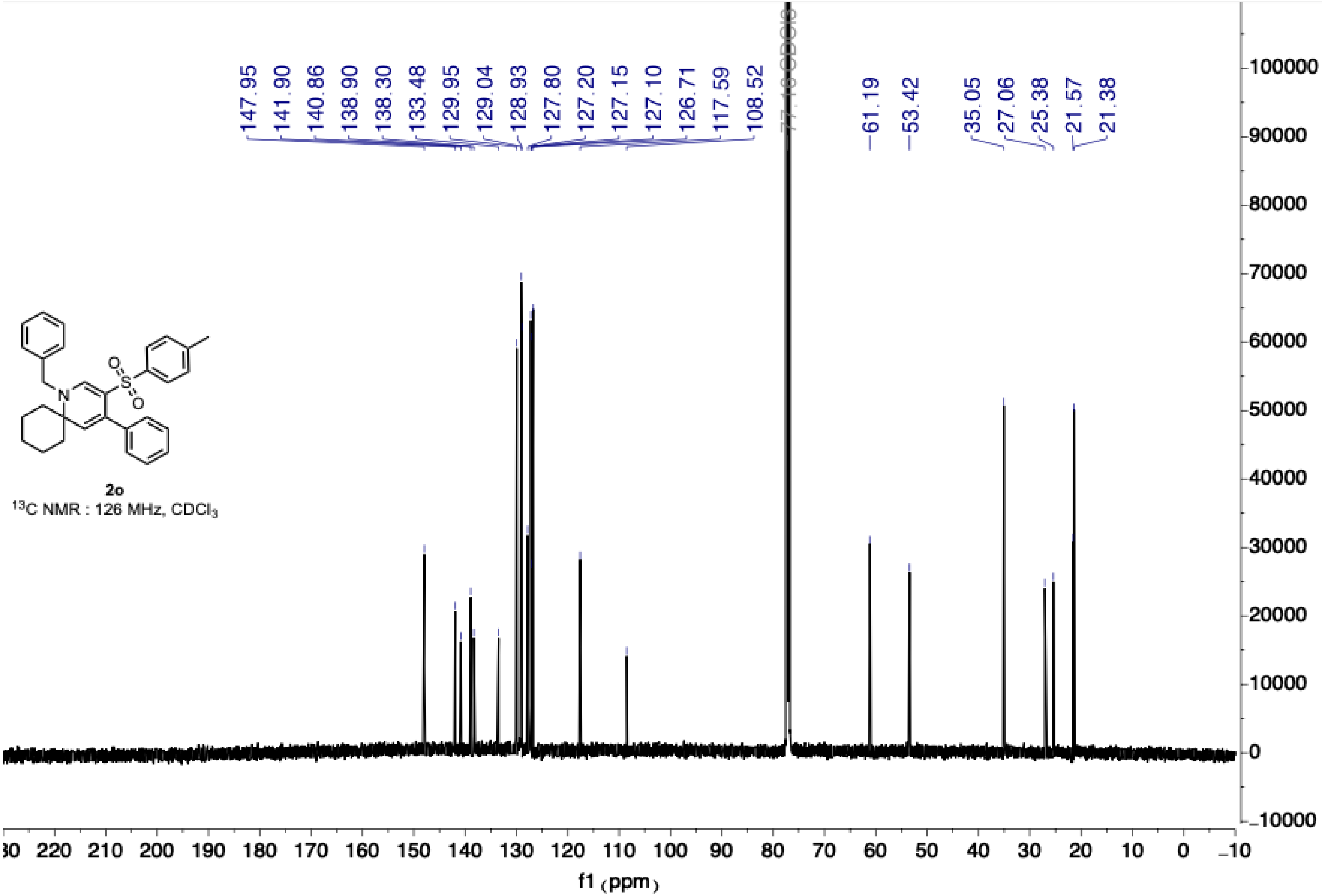
147.95
141.90
140.86
138.90
138.30
133.48
129.95
129.04
128.93
127.80
127.20
127.15
127.10
126.71
117.59
108.52
77.16 CDCl3
61.19
53.42
35.05
27.06
25.38
21.57
21.38
2o
^{13}C NMR : 126 MHz, CDCl$_3$
f1 (ppm)

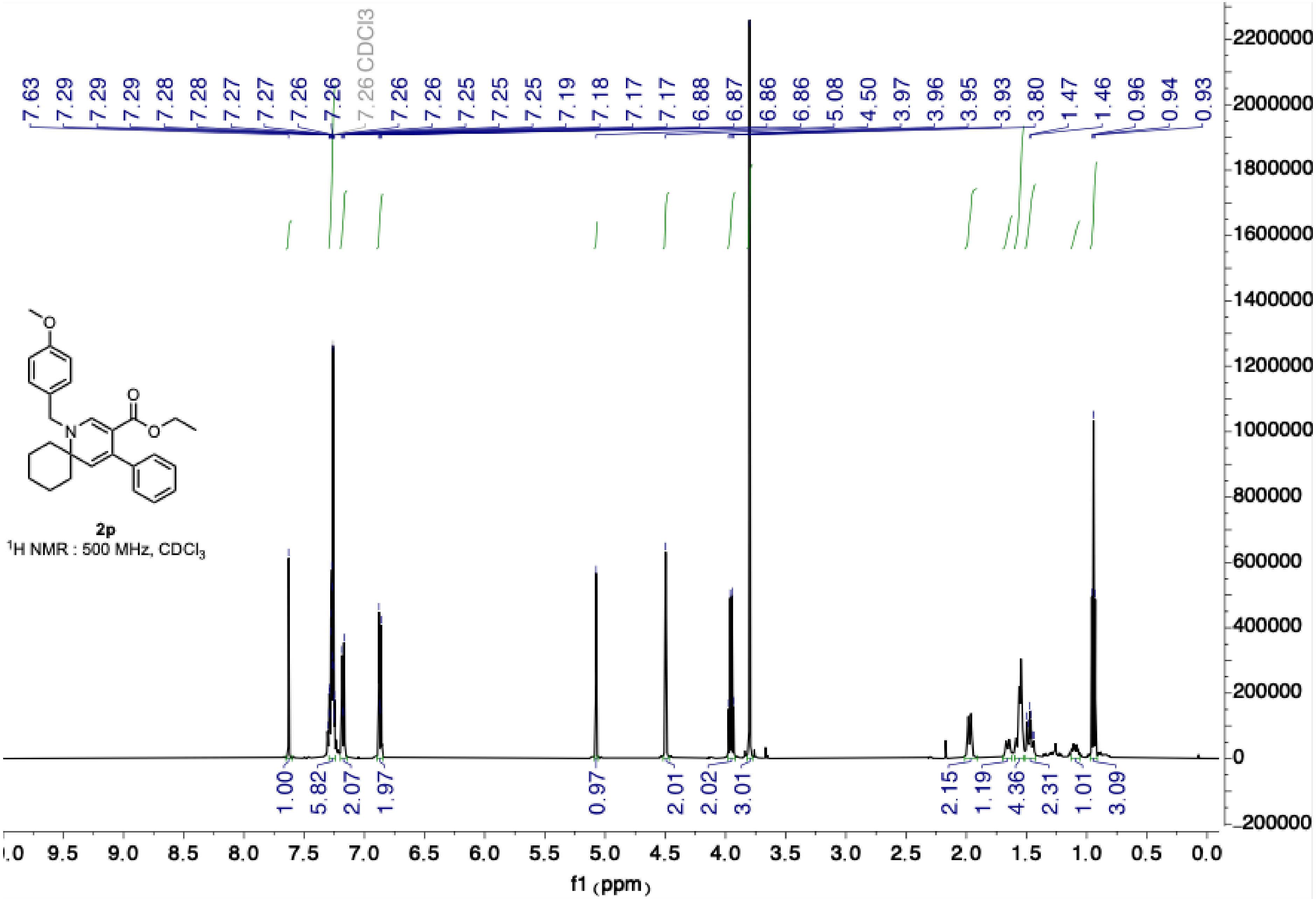
2p
1H NMR : 500 MHz, CDCl3
7.63 7.29 7.29 7.29 7.28 7.28 7.27 7.27 7.26 7.26 7.26 CDCl3 7.26 7.26 7.25 7.25 7.25 7.19 7.18 7.17 7.17 6.88 6.87 6.86 6.86 5.08 4.50 3.97 3.96 3.95 3.93 3.80 1.47 1.46 0.96 0.94 0.93
1.00 5.82 2.07 1.97 0.97 2.01 2.02 3.01 2.15 1.19 4.36 2.31 1.01 3.09
f1 (ppm)

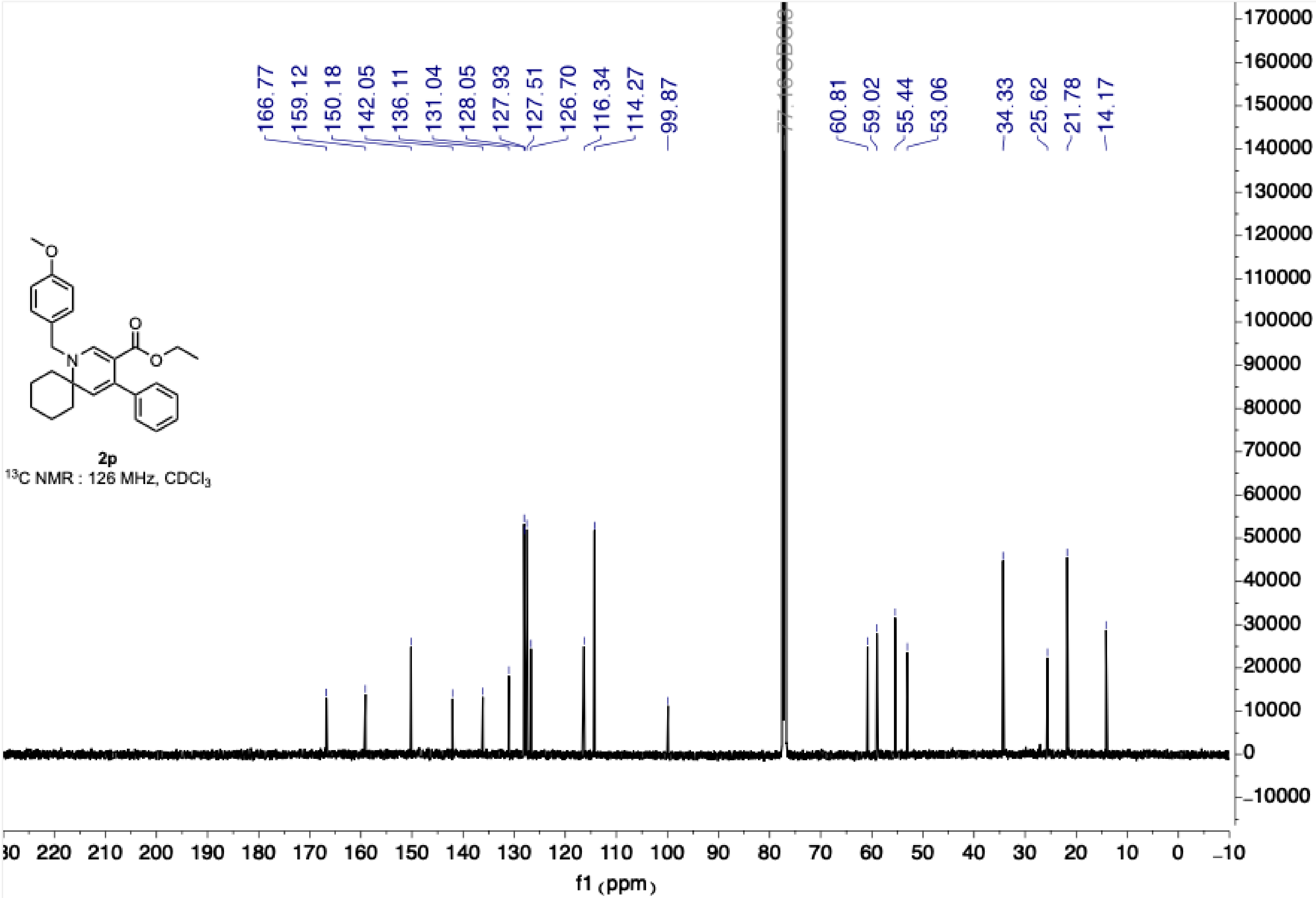
166.77
159.12
150.18
142.05
136.11
131.04
128.05
127.93
127.51
126.70
116.34
114.27
99.87
77.16 CDCl3
60.81
59.02
55.44
53.06
34.33
25.62
21.78
14.17
2p
13C NMR : 126 MHz, CDCl3
f1 (ppm)

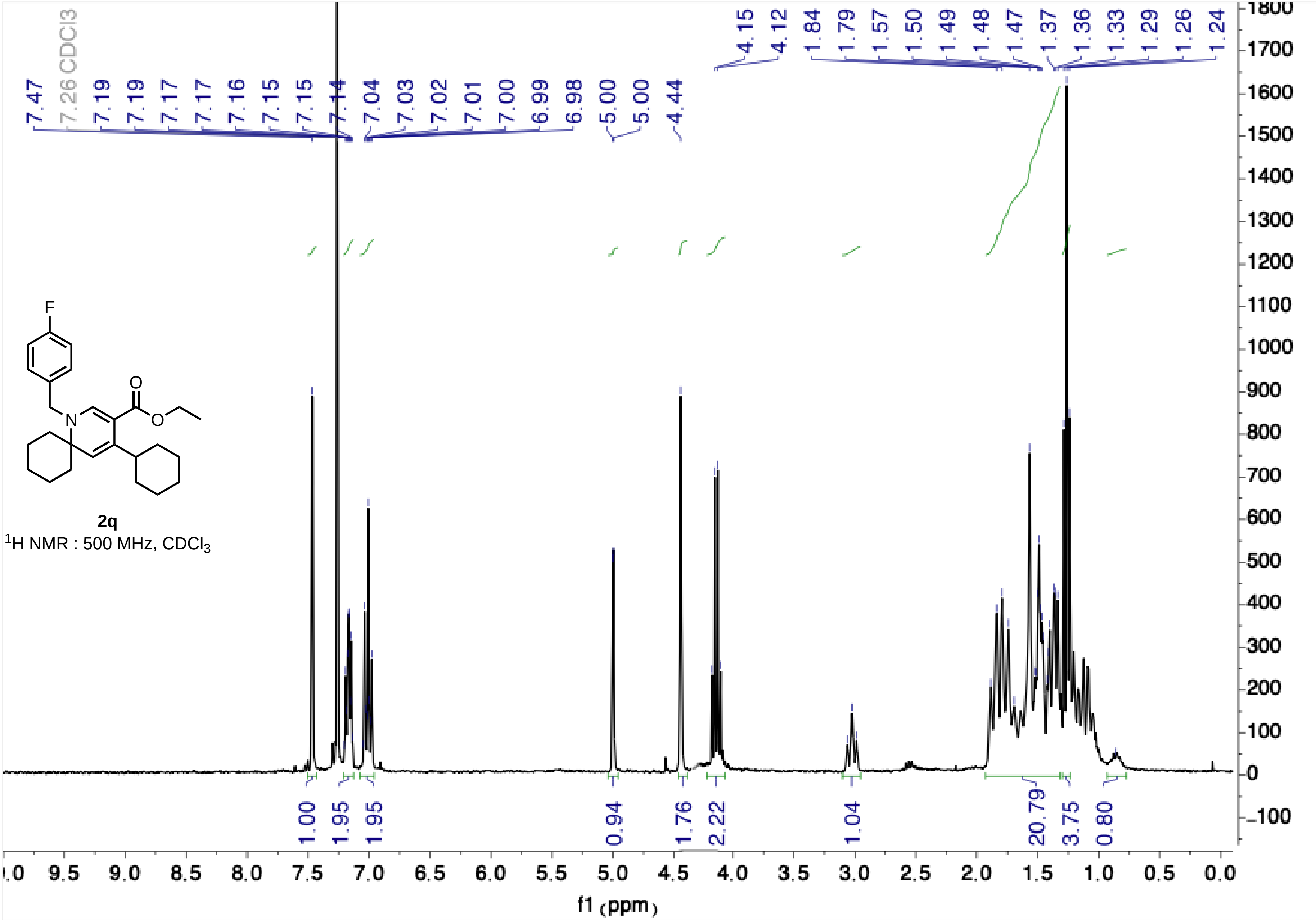

2q

^{1}H NMR : 500 MHz, $CDCl_3$

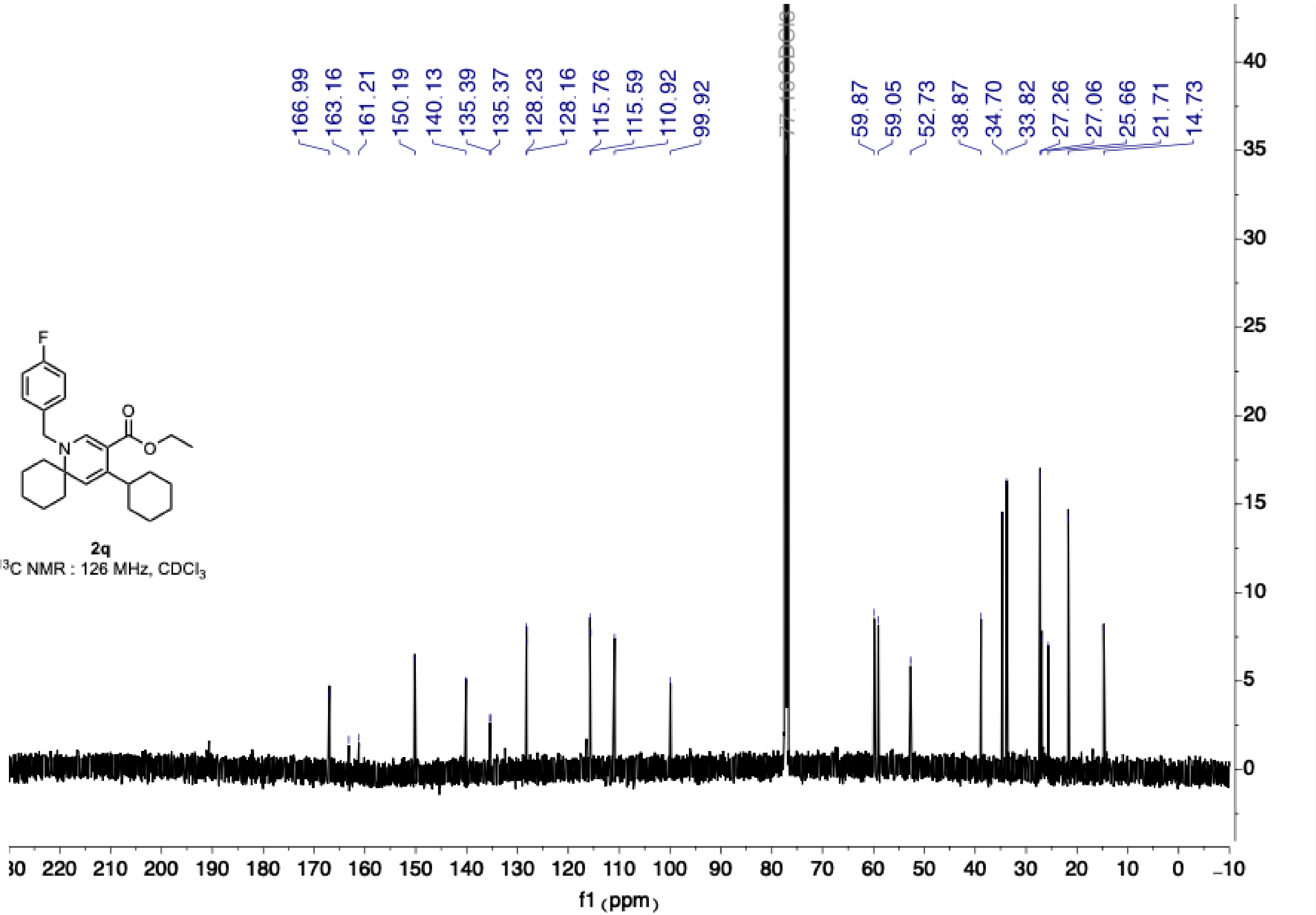

2q

^{13}C NMR : 126 MHz, $CDCl_3$

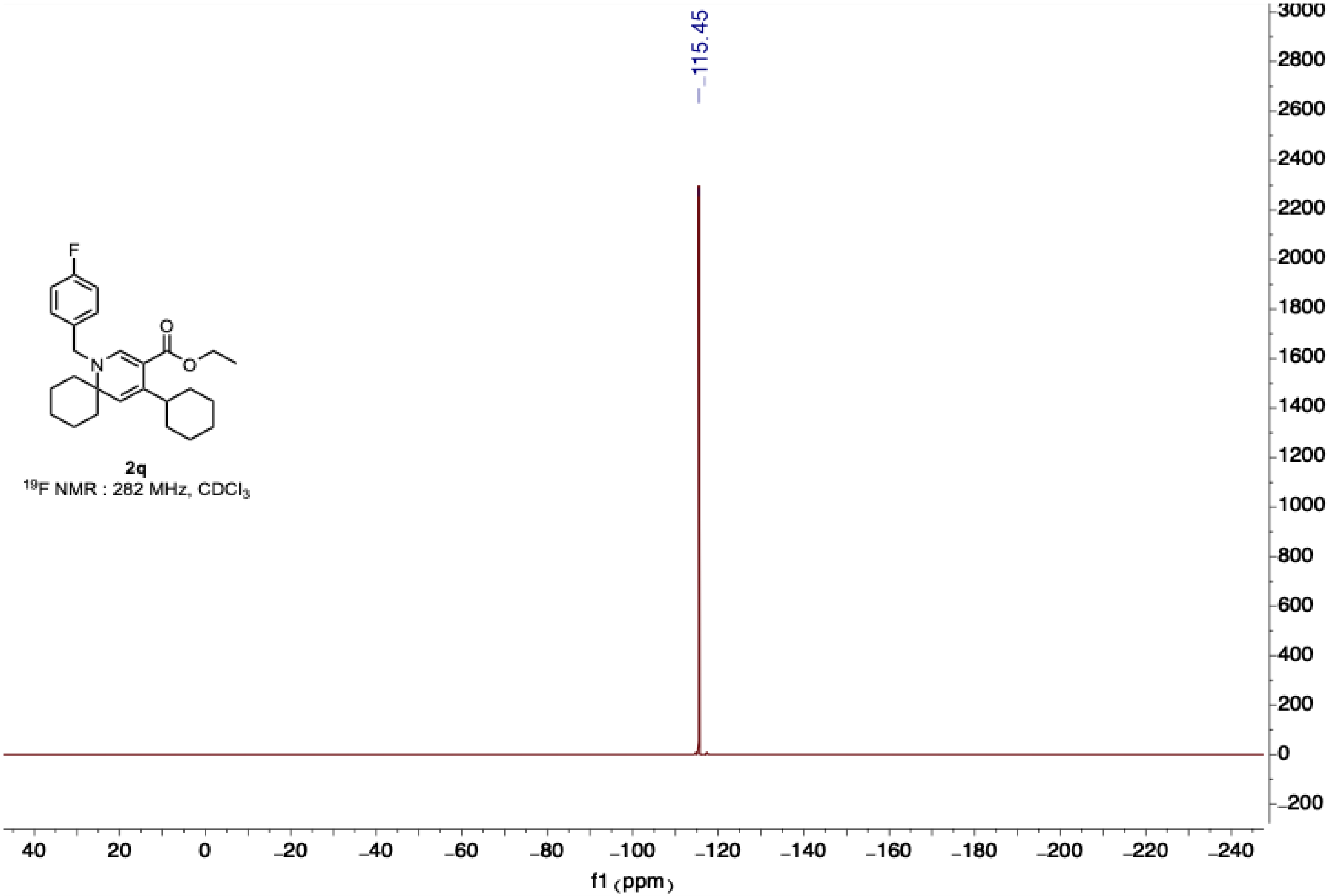

2q
^{19}F NMR : 282 MHz, $CDCl_3$

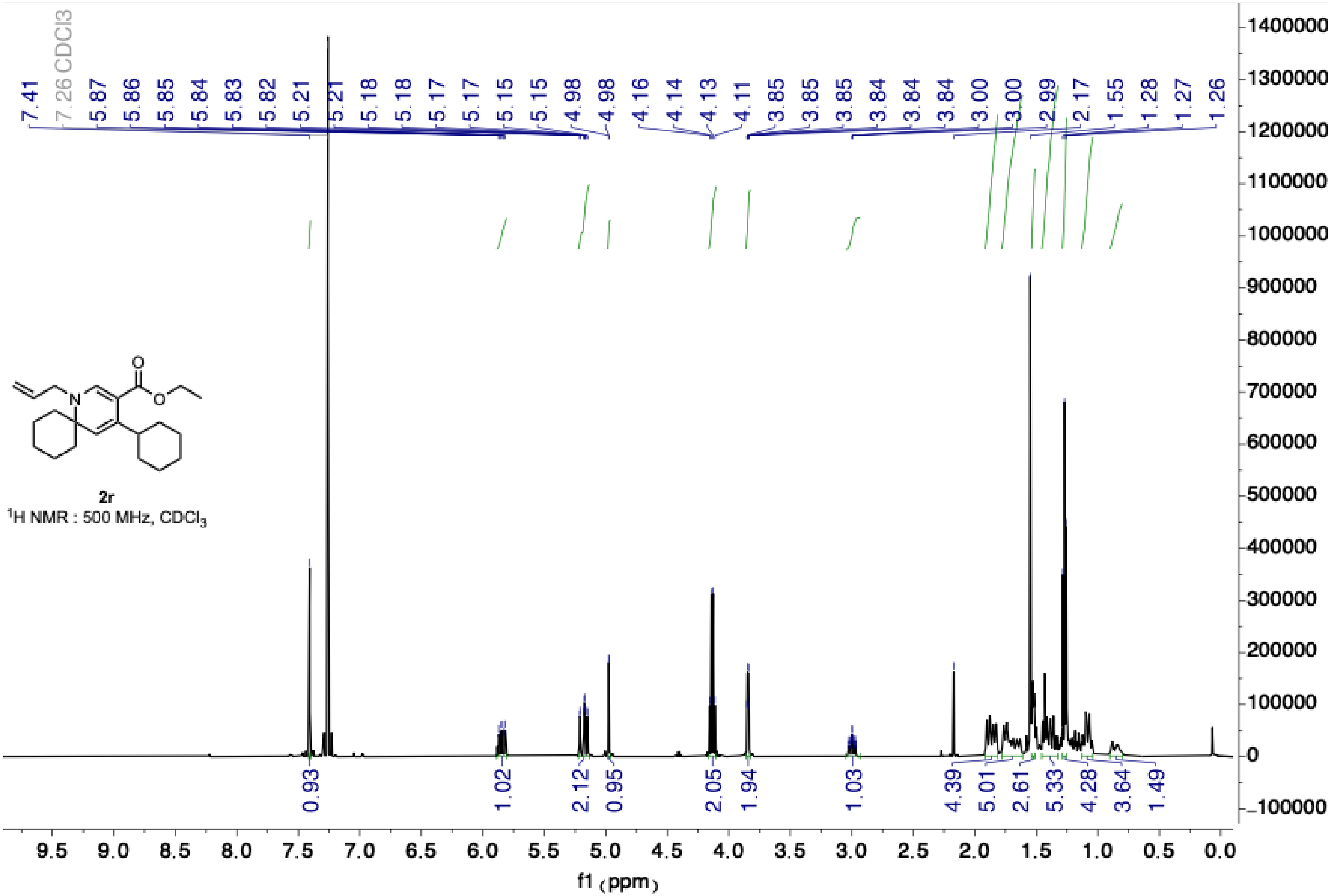

2r

^{1}H NMR : 500 MHz, $CDCl_3$

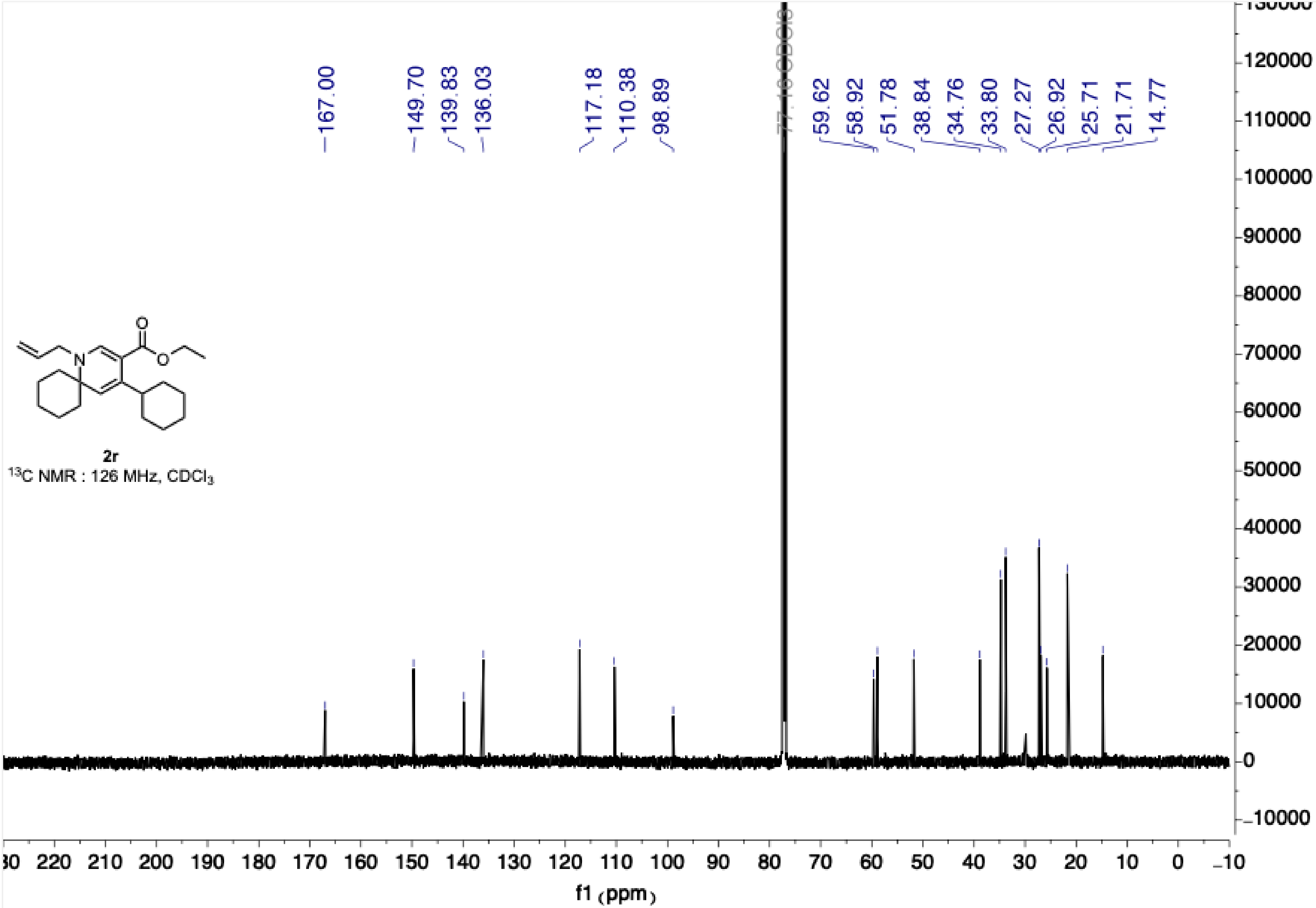
167.00
149.70
139.83
136.03
117.18
110.38
98.89
77.16 CDCl3
59.62
58.92
51.78
38.84
34.76
33.80
27.27
26.92
25.71
21.71
14.77
2r
13C NMR : 126 MHz, CDCl3
f1 (ppm)

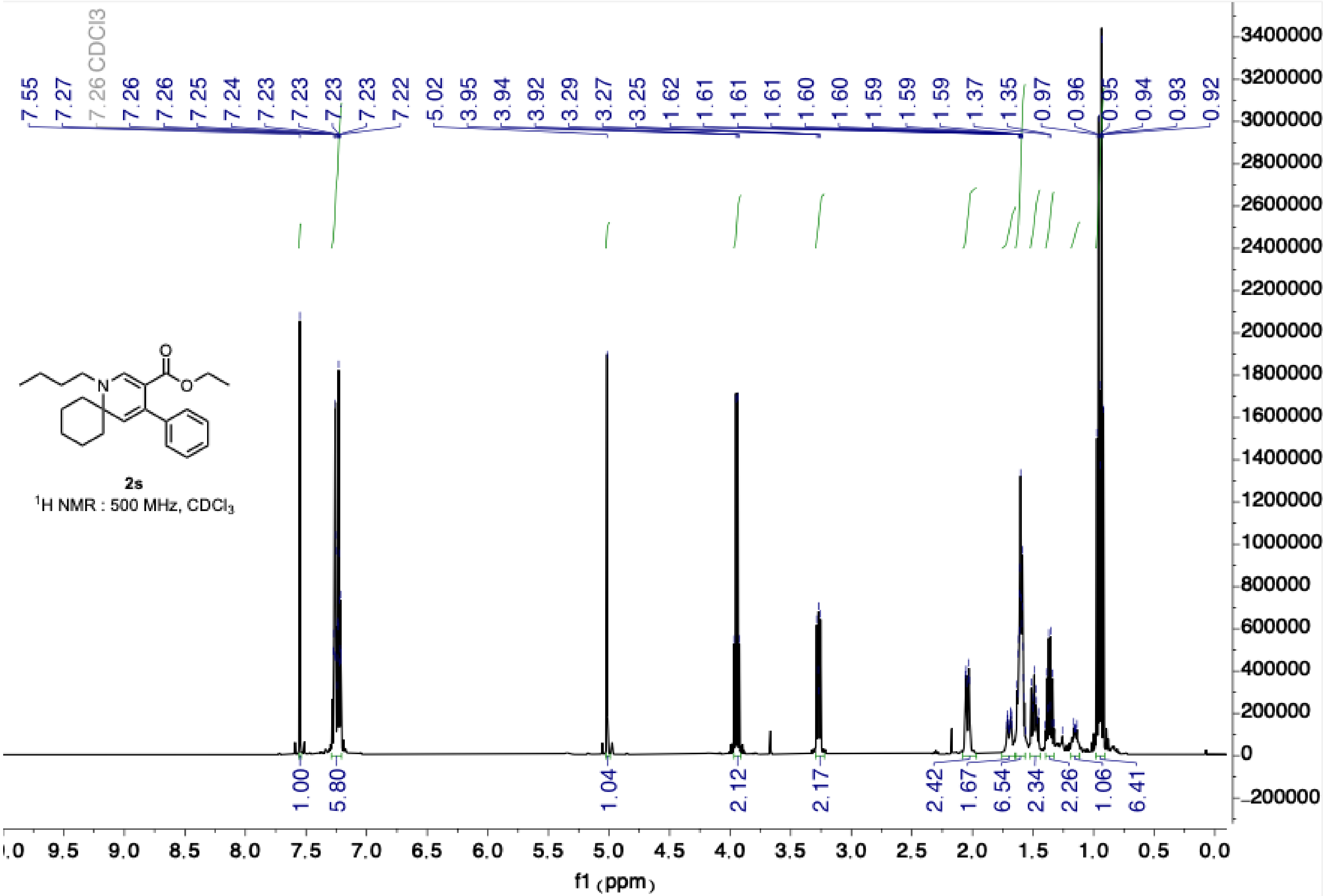

2s
^{1}H NMR : 500 MHz, $CDCl_3$
f1 (ppm)

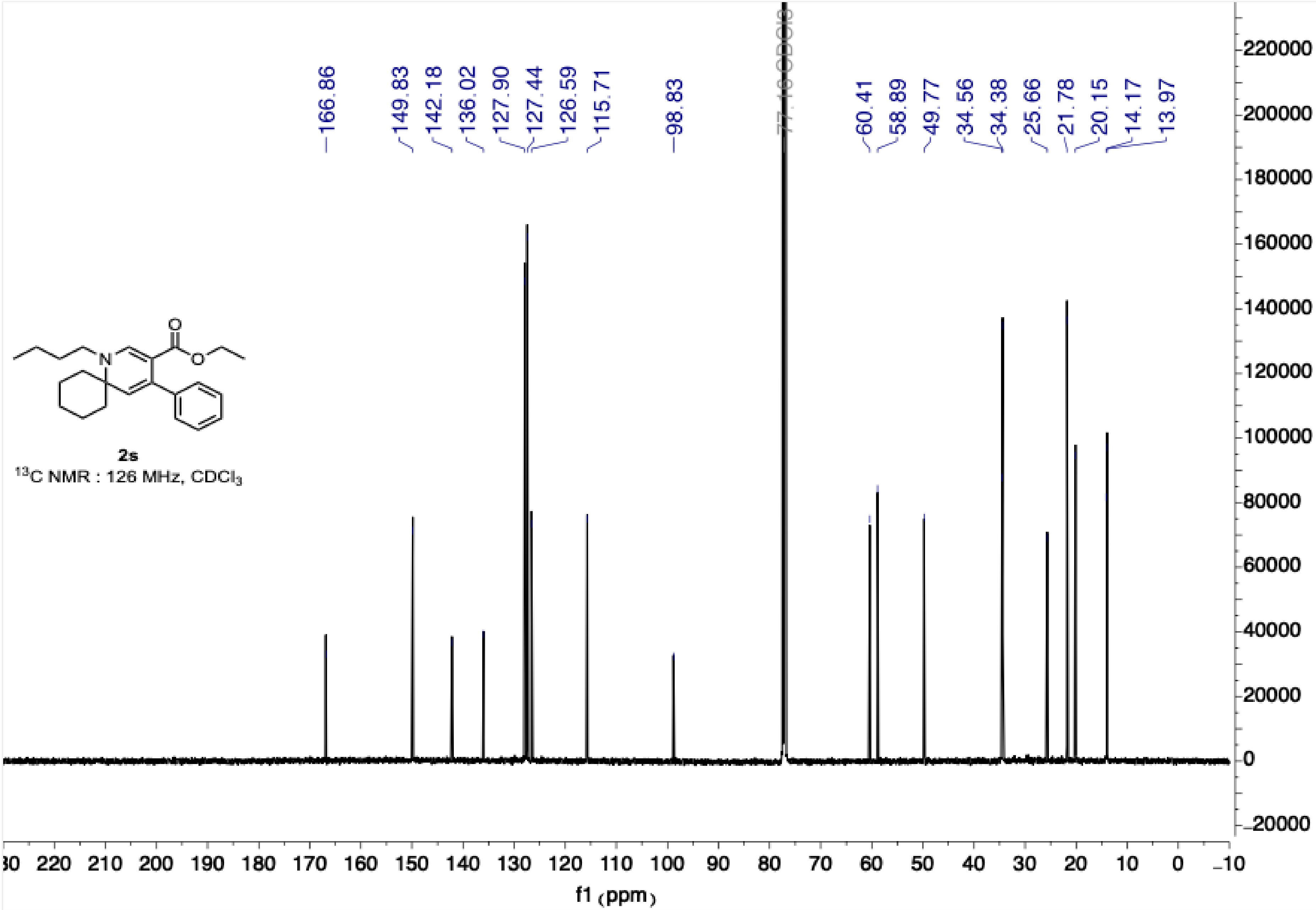
166.86
149.83
142.18
136.02
127.90
127.44
126.59
115.71
98.83
77.16 CDCl3
60.41
58.89
49.77
34.56
34.38
25.66
21.78
20.15
14.17
13.97
2s
^{13}C NMR : 126 MHz, CDCl$_3$
f1 (ppm)
